# WTAP tetramer ensures m^6^A writer assembly and faithful mitosis

**DOI:** 10.1038/s44319-026-00815-3

**Published:** 2026-06-02

**Authors:** Shan Zhang, Jin Ye, Manjuan Zhang, Jie Cao, Yujuan Li, Ranran Hu, Da Chen, Ting Li, Jiasheng Chen, Wen Zhu, Wenli Jiang, Jianchao Li, Wei Hu, Jianzhao Liu, Xing Liu, Chao Wang

**Affiliations:** 1 Department of Neurology, the First Affiliated Hospital of USTC, Center for Advanced Interdisciplinary Science and Biomedicine of IHM, Biomedical Sciences and Health Laboratory of Anhui Province, Division of Life Sciences and Medicine, University of Science and Technology of China, Hefei, China; 2 Ministry of Education Key Laboratory for Membrane-less Organelles and Cellular Dynamics, Hefei National Research Center for Physical Sciences at the Microscale, School of Life Sciences, University of Science and Technology of China, Hefei, China; 3 Ministry of Education Key Laboratory of Macromolecular Synthesis and Functionalization, Department of Polymer Science and Engineering, Zhejiang University, Hangzhou, China; 4 Division of Cell, Developmental and Integrative Biology, School of Medicine, South China University of Technology, Guangzhou, China; 5 Present Address: School of Life Sciences, East China Normal University, Shanghai, China

**Keywords:** Cell Cycle, RNA Biology, Structural Biology

## Abstract

The m^6^A methyltransferase complex (“writer”) regulates mRNA stability and translation, but how its assembly is orchestrated remains incompletely understood. Wilms’ tumor 1-associating protein (WTAP) is a conserved regulatory subunit essential for m^6^A deposition and cell proliferation, yet its structural organization and mechanistic contributions remain elusive. Here, we report that WTAP dimerizes and further assembles into a stable tetramer through its middle coiled-coil domain, as revealed by high-resolution crystal structures. Disruption of this tetrameric interface abolishes WTAP’s interaction with METTL3, METTL14, and ZC3H13, impairs m^6^A deposition, and fails to rescue proliferation defects in WTAP-depleted cells. Live-cell imaging demonstrates that WTAP is required for accurate chromosome segregation, and MeRIP-seq analysis identifies WTAP-dependent m^6^A regulation as a critical determinant sustaining the expression of mitotic regulators, including KIF20A. Together, our study defines a tetrameric scaffold function for WTAP that is essential for writer complex integrity and highlights its pivotal role in linking m^6^A methylation to cell cycle progression.

## Synopsis

WTAP forms a stable tetramer through its middle coiled-coil domain, which is essential for m^6^A writer complex assembly. Disruption of WTAP tetramerization impairs m^6^A deposition and compromises mitotic fidelity by altering the expression of key mitotic regulators.

- WTAP assembles into a stable tetramer through its middle coiled-coil domain.
- WTAP tetramerization is required for m^6^A writer complex assembly and efficient m^6^A deposition.
- WTAP-dependent m^6^A regulation sustains expression of mitotic regulators to ensure chromosome segregation fidelity.

## Abstract (web summary)

WTAP forms a stable tetramer through its middle coiled-coil domain, which is essential for m^6^A writer complex assembly. Disruption of WTAP tetramerization impairs m^6^A deposition and compromises mitotic fidelity by altering the expression of key mitotic regulators.

## Graphical abstract

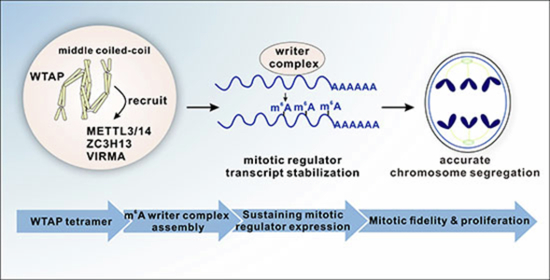

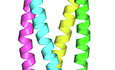

## Introduction

N^6^-methyladenosine (m^6^A) is the most prevalent internal modification in eukaryotic mRNA, predominantly enriched around stop codons and within 3’ untranslated regions (UTRs) (Dominissini et al, 2012; Meyer et al, 2013). This dynamic and reversible mark plays critical roles in nearly every stage of mRNA metabolism, including splicing (Horiuchi et al, 2013; Haussmann et al, 2016), export (Wickramasinghe and Laskey, 2015), translation (Meyer et al, 2015), and decay (Wang et al, 2014a, 2014b). Consequently, m^6^A has emerged as a key regulatory layer in numerous biological processes such as cell cycle progression (Batista et al, 2014), differentiation (Geula et al, 2015), DNA repair (Xiang et al, 2017), circadian rhythm (Wang et al, 2015; Fustin et al, 2013), dosage compensation (Patil et al, 2016), and sex determination (Lence et al, 2016; Yan and Perrimon, 2015; Kan et al, 2017). Advances in transcriptome-wide m^6^A profiling and functional studies have begun to reveal the mechanistic basis of these diverse outcomes (Wang et al, 2019; Meyer and Jaffrey, 2015). However, despite increasing insights into m^6^A-mediated regulation, our understanding of the molecular architecture and functional organization of the m^6^A methyltransferase (“writer”) complex remains incomplete.

The m^6^A writer complex consists of a core heterodimer formed by the catalytic subunits METTL3 and METTL14 (Liu et al, 2014; Ping et al, 2014; Schwartz et al, 2014), which require additional regulatory partners for full activity and specificity. Among these, WTAP (Wilms’ tumor 1-associating protein) is indispensable for recruiting the core enzyme to nuclear speckles and for guiding deposition onto specific transcripts (Ping et al, 2014). WTAP also serves as a scaffold protein that bridges METTL3/METTL14 with additional subunits such as VIRMA (KIAA1429) (Schwartz et al, 2014; Yue et al, 2018), ZC3H13 (Knuckles et al, 2018; Wen et al, 2018), RBM15 and its paralog RBM15B (Moindrot et al, 2015; Patil et al, 2016), and HAKAI (CBLL1) (Růžička et al, 2017). Cryo-EM structures of partial writer assemblies have shown that WTAP and VIRMA form the structural core of the regulatory module (Su et al, 2022; Yan et al, 2022). Despite these advances, the detailed quaternary architecture of WTAP and how it organizes the m^6^A machinery at the molecular level remain largely unknown.

WTAP is a nuclear protein containing a predicted N-terminal coiled-coil domain and a largely unstructured C-terminal region (Schöller et al, 2018). Initially identified as a WT1-interacting protein (Little et al, 2000), it has since been implicated in cell proliferation, splicing regulation, and embryonic development (Horiuchi et al, 2006; Small et al, 2006; Fukusumi et al, 2008). Prior studies suggest that WTAP promotes G2/M progression by stabilizing cyclin A2 transcripts (Horiuchi et al, 2006), and that its loss leads to G2/M arrest and reduced proliferation (Horiuchi et al, 2013). However, whether these functions are mechanistically linked to WTAP’s role in the m^6^A pathway, and whether its molecular assembly state is essential for executing such functions, remains unclear. In particular, it is unknown whether WTAP functions as a monomer, dimer, or higher-order oligomer in vivo, and whether this structural organization underlies its scaffolding role within the writer complex.

Here, we demonstrate that WTAP exists as a dimeric building block, which further assembles into a stable tetramer via its middle coiled-coil domain. Structure-guided mutagenesis uncovered a layered hydrophobic and electrostatic interface critical for tetramerization. Disruption of this assembly not only impairs WTAP’s interactions with METTL3, METTL14, and ZC3H13, but also abolishes its ability to rescue m^6^A deposition and cell proliferation in WTAP-depleted cells. Mechanistically, MeRIP-seq analysis identifies that WTAP-dependent m^6^A methylation sustains KIF20A expression, which in turn ensures accurate chromosome segregation and mitotic progression. Together, our findings establish the structural basis of WTAP oligomerization, define its essential role in m^6^A writer complex assembly, and uncover a mechanistic link between m^6^A deposition, mitotic fidelity, and cell cycle regulation.

## Results

### WTAP assembles into a tetramer through its middle coiled-coil domain

**Figure 1:**
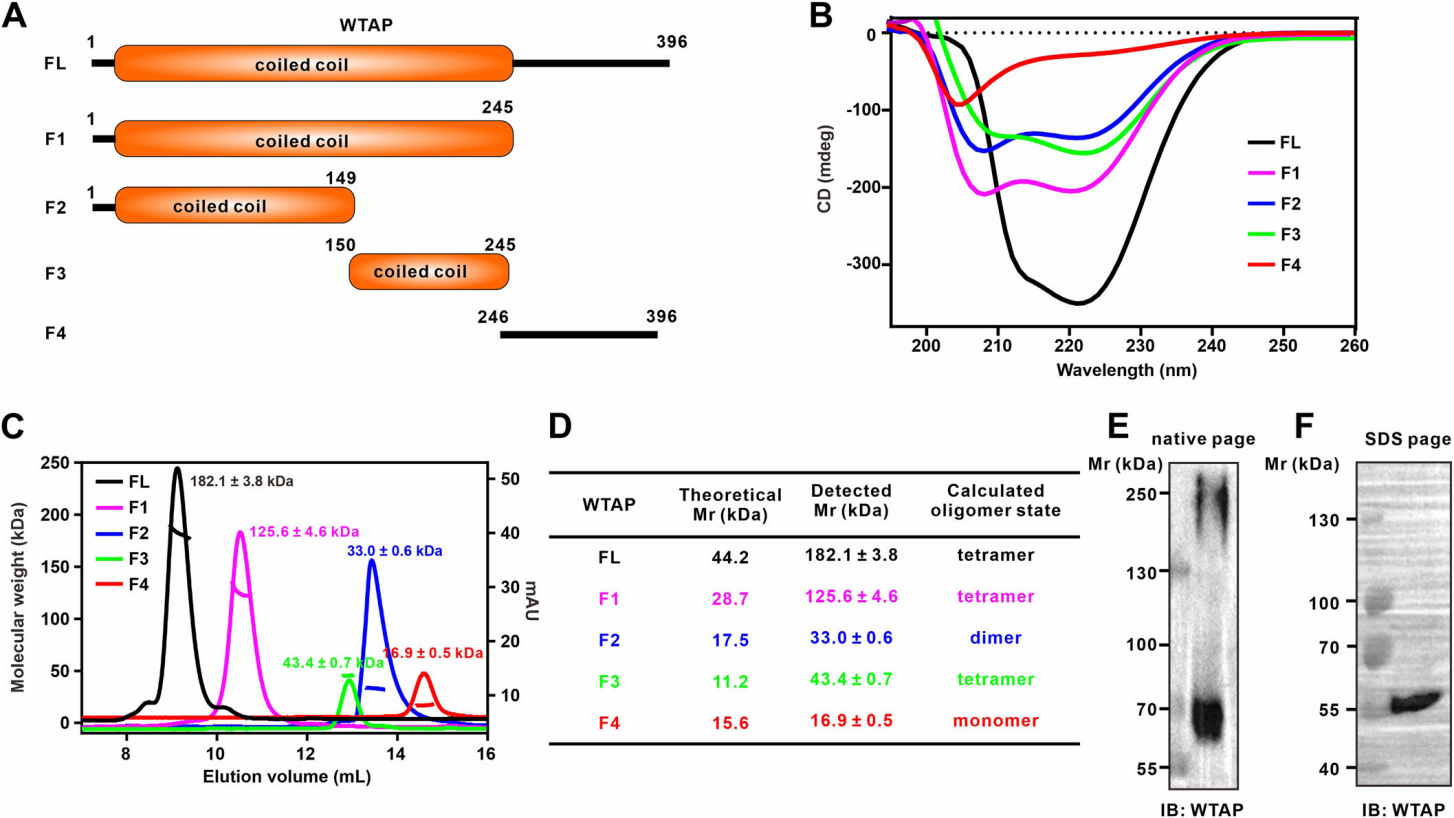
WTAP assembles into a stable tetramer through its middle coiled-coil domain. (**A**) Domain organization of full‑length human WTAP and truncation constructs used in this study (FL: residues 1–396; F1: residues 1–245; F2: residues 1–149; F3: residues 150–245; F4: residues 246–396). (**B**) Circular dichroism (CD) spectra showing that fragments containing the coiled‑coil region (F1, F2, F3) adopt predominantly α‑helical secondary structures, whereas F4 lacks ordered secondary structure. (**C**) FPLC‑MALS analysis showing that FL WTAP and coiled‑coil containing fragments (F1 and F3) elute predominantly as tetramers, whereas F2 forms a dimer and F4 behaves as a monomer. (**D**) Calculated molecular weights from FPLC‑MALS (C), confirming the oligomeric state of each fragment. (**E**, **F**) Native PAGE (**E**) and SDS-PAGE (**F**) analysis of endogenous WTAP from HEK293T lysates, showing that WTAP forms higher‑order oligomers under physiological conditions. Source data are available online for this figure.

**Figure EV1:**
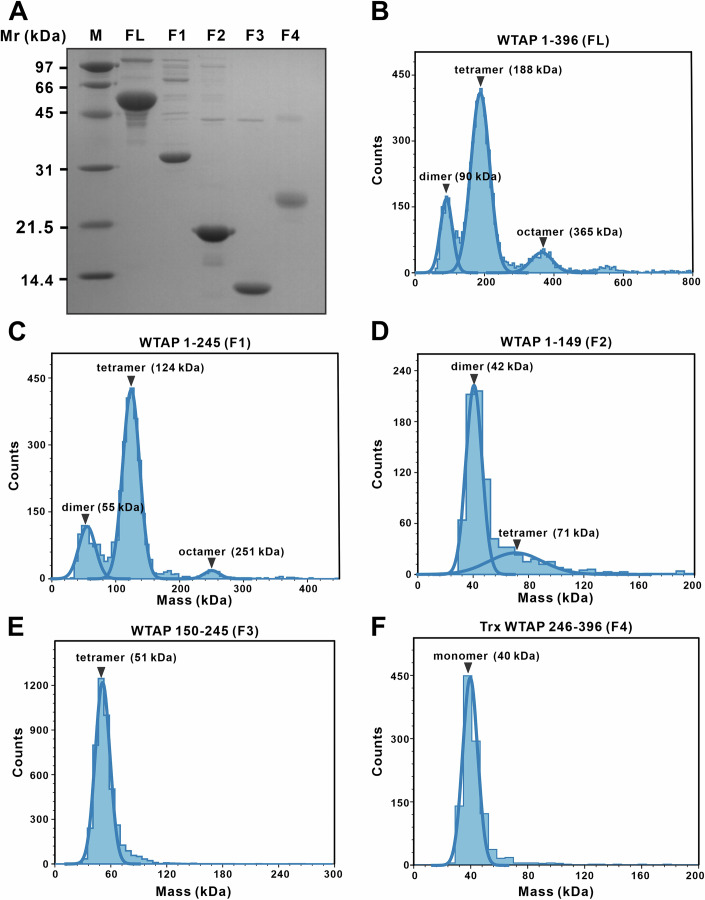
Biochemical analysis of full-length and truncated WTAP constructs. (**A**) SDS-PAGE analysis of purified full-length WTAP and truncated fragments (F1-F4), followed by Coomassie blue staining. (**B**–**F**) Mass photometry analysis of purified full-length WTAP (**B**) and truncated fragments F1 (**C**), F2 (**D**), F3 (**E**), and F4 (**F**). All measurements were performed at a protein concentration of 0.2 μM. The molecular masses of the detected species are indicated. Source data are available online for this figure.

Previous studies have shown that WTAP forms a homodimer with VIRMA, contributing to the structural core of the m^6^A “writer” complex (Yan et al, 2022; Su et al, 2022). To further investigate the biochemical properties of WTAP, we purified a series of truncated fragments (Figs. 1A and EV1A). Circular dichroism (CD) spectroscopy revealed that fragments F1 (residues 1–245), F2 (residues 1–149), and F3 (residues 150–245) adopt α-helical conformations, whereas F4 (residues 246–396) is largely unstructured (Fig. 1B). Fast protein liquid chromatography coupled with multi-angle light scattering (FPLC-MALS) demonstrated that full-length (FL) WTAP (residues 1–396) exists as a tetramer in solution (Fig. 1C,D). Interestingly, the N-terminal fragment F1 also formed tetramers, while the C-terminal F4 was monomeric (Fig. 1C,D). Further dissection of the coiled-coil domain showed that the F2 fragment dimerized, whereas the F3 segment (hereafter termed “WTAP M-CC”) retained a tetrameric state.

To further validate the oligomeric state of WTAP under dilute conditions, we performed single-molecule mass photometry to directly measure the molecular masses of WTAP fragments. These measurements are fully consistent with the FPLC-MALS results, showing that full-length WTAP and the F1 fragment predominantly exist as tetramers, with the F3 segment responsible for tetramer formation, whereas F2 forms dimers and F4 remains monomeric (Fig. EV1B–F). Together, these results identify the middle portion of the coiled-coil region as the key determinant of WTAP tetramerization.

To confirm the physiological relevance of this oligomeric state, we analyzed endogenous WTAP in HEK293 cell lysates using both denaturing (SDS-PAGE) and native gel electrophoresis. Western blot analysis under native conditions revealed a higher-order WTAP species consistent with a tetrameric assembly in cells (Fig. 1E,F). Together, these results demonstrate that WTAP forms a stable tetramer both in vitro and under cellular conditions, and that tetramerization is mediated by its middle coiled-coil domain.

### Structure of the WTAP N-terminal region reveals a dimeric architecture

**Figure 2:**
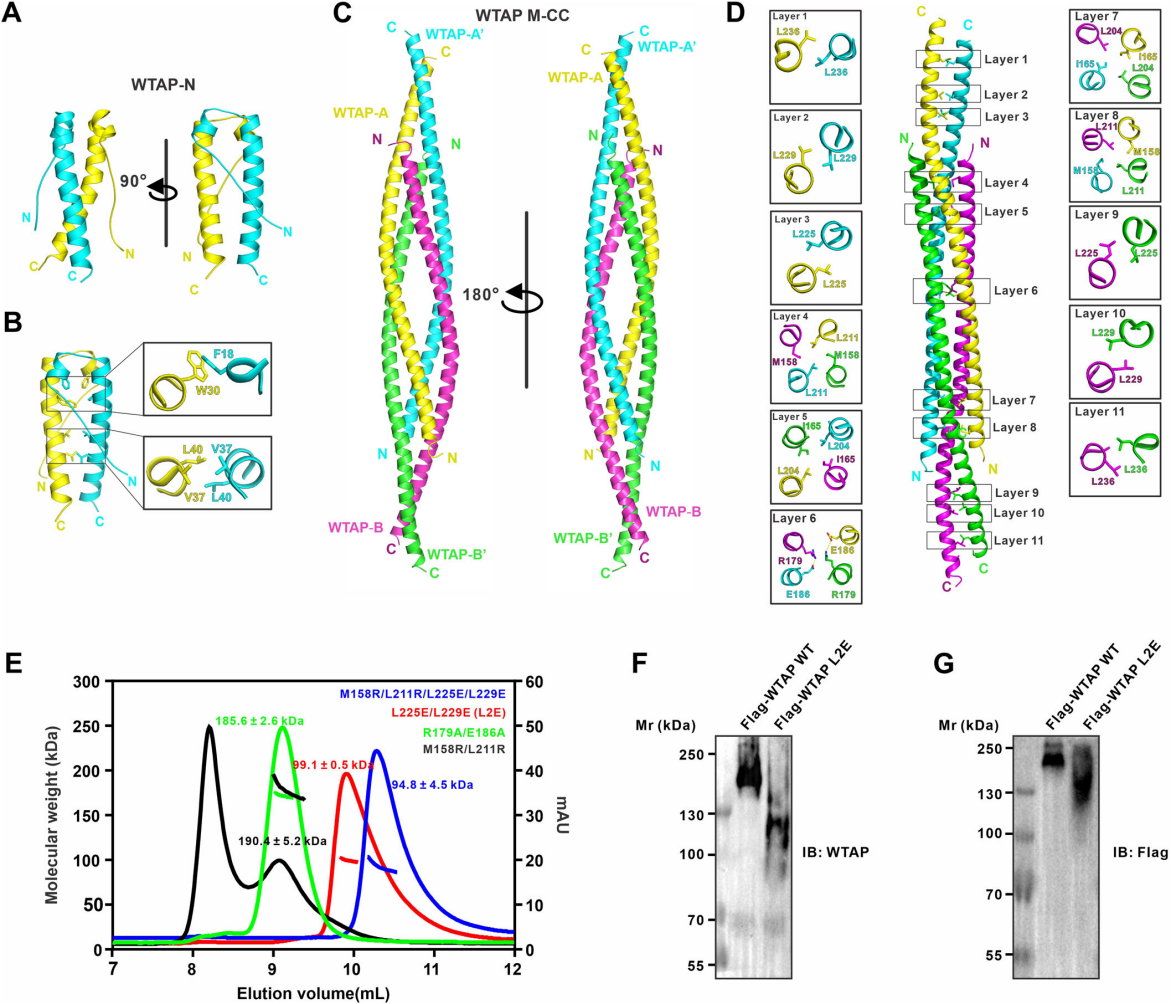
Crystal structures reveal that WTAP tetramerization is mediated by a layered coiled‑coil architecture. (**A**) Crystal structure of the WTAP N‑terminal region (WTAP‑N, residues 1–50), showing a parallel dimer formed by two α‑helices. (**B**) Close‑up view of the hydrophobic core stabilizing WTAP‑N dimerization. Mutation W30R disrupts this packing and monomerizes the fragment. (**C**) Crystal structure of the middle coiled‑coil region (WTAP M‑CC, residues 150–245), revealing a tetrameric four‑helix bundle formed by two parallel dimers in a head‑to‑head arrangement. (**D**) Structural basis of WTAP tetramerization. Layered hydrophobic and electrostatic interactions organize the tetrameric interface: layers 3/2/1 and 9/10/11 stabilize intra‑dimer packing (Leu225, Leu229, L236), whereas layers 4/8 and layers 5/7 mediate dimer-dimer interactions (M158, L211, I165, and L204). A salt bridge (Arg179-Glu186) further contributes to tetramer stabilization. (**E**) FPLC‑MALS analysis showing the oligomeric states of WTAP variants. The core‑disrupting mutant L225E/L229E (L2E) shifts WTAP from tetramer to dimer, while combining L2E with M158R/L211R eliminates higher‑order species. (**F**, **G**) Native PAGE analysis of Flag‑WTAP expressed in HEK293T cells confirms that WTAP L2E predominantly exists as a dimer in cells, whereas WTAP WT forms higher-order oligomers. Detection was performed using anti-WTAP (**F**) and anti-Flag (**G**) antibodies. Source data are available online for this figure.

**Figure EV2:**
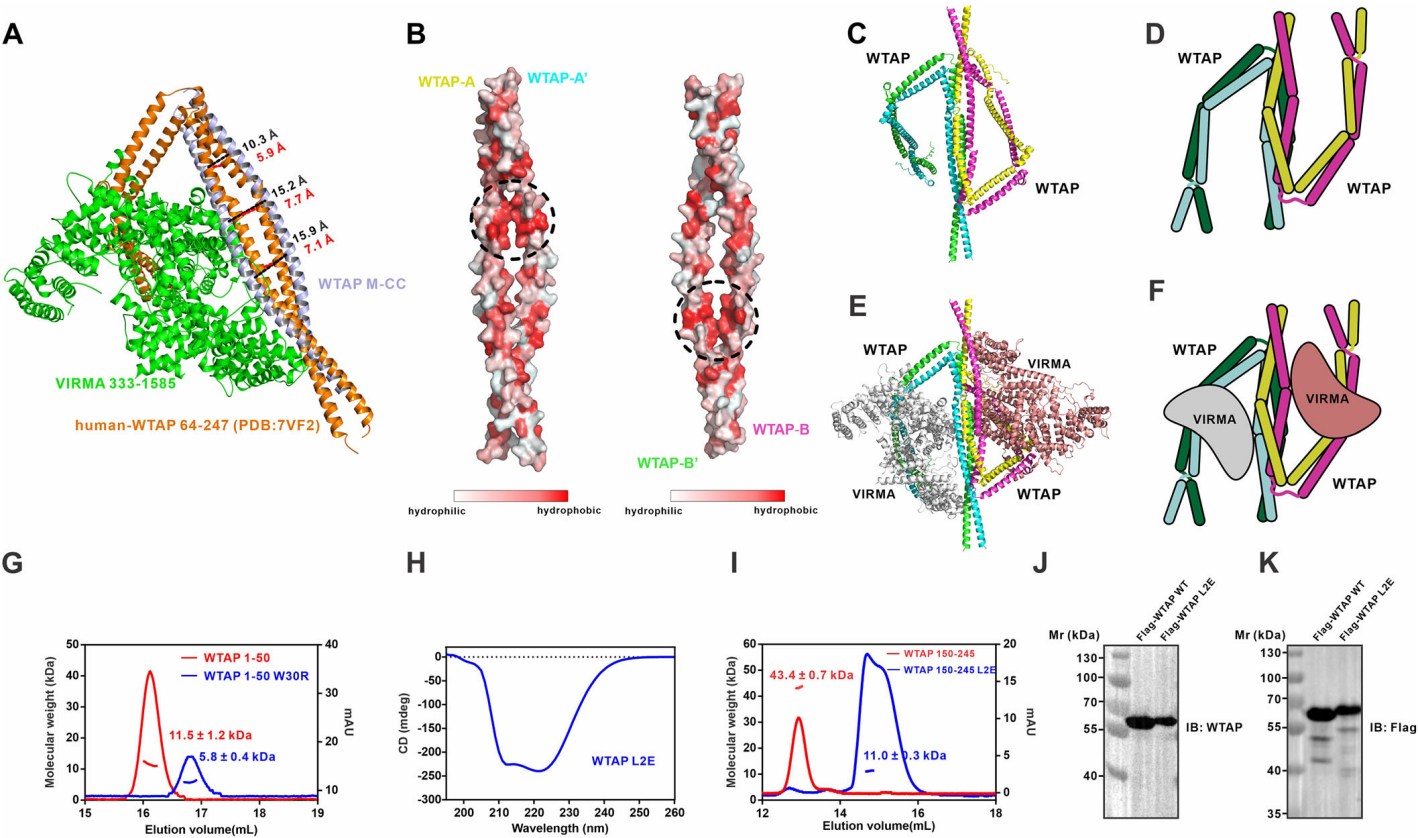
Structure-guided mutagenesis of WTAP disrupts oligomeric assembly while preserving secondary structure. (**A**) Superimposition of the WTAP M-CC crystal structure from this study (cyan) with the previously reported WTAP cryo-EM structure (PDB: 7VF2, orange) (Su et al, 2022). Distances between helices within the coiled-coil domain are indicated. (**B**) Hydrophobic interfaces between α-helices: left, WTAP-A and WTAP-A’; right, WTAP-B and WTAP-B’. Hydrophobic regions are highlighted in red. (**C**, **D**) Cartoon representation (**C**) and schematic diagram (**D**) of WTAP (residues 1–245), showing hierarchical assembly from dimer to tetramer via the central coiled-coil domain. (**E**, **F**) Cartoon representation (**E**) and schematic diagram (**F**) of WTAP (residues 1–245) in complex with VIRMA. (**G**) FPLC-MALS analysis of WTAP-N W30R mutant showing loss of dimerization, confirming disruption of the N-terminal hydrophobic interface. (**H**) Circular dichroism (CD) spectrum of the WTAP L2E mutant, showing retention of α-helical secondary structure despite loss of tetramerization. (**I**) FPLC-MALS analysis of WTAP M-CC (residues 150–245) L2E mutant, showing monomerization of the middle coiled-coil domain. (**J**, **K**) Immunoblot analysis of HEK293T cells expressing Flag-WTAP WT or L2E mutant. WTAP was detected using anti-WTAP (**J**) and anti-Flag (**K**) antibodies. Source data are available online for this figure.

To elucidate the structural basis of WTAP tetramerization, we performed X-ray crystallography on individual domains of the protein. While full-length WTAP failed to crystallize, likely due to conformational flexibility in its C-terminal region, we successfully determined the structure of the N-terminal region (WTAP-N, residues 1–50) at 1.65 Å resolution (Table EV1). This domain forms a stable dimer, consisting of a pair of α-helices engaging in hydrophobic packing (Fig. 2A,B). The dimer interface is stabilized by a hydrophobic core, and a W30R substitution at this site abolished dimerization, converting the construct into a monomer (Figs. 2B and EV2G).

### Structure of the WTAP middle coiled-coil domain reveals a tetrameric bundle

As a complement to the N-terminal dimer structure, we next solved the crystal structure of the middle coiled-coil domain (WTAP M-CC, residues 150–245) at 2.8 Å resolution using selenomethionine-based single-wavelength anomalous dispersion (SeMet-SAD) phasing (Table EV1). The structure reveals a homotetrameric assembly composed of two parallel α-helical monomers (A and A’) forming a supercoiled dimer, which interacts head-to-head with an identical dimer (B and B’) in an antiparallel orientation to form a four-stranded helical bundle (Fig. 2C).

We next performed a detailed comparison between our tetrameric WTAP structure and the previously reported cryo-EM structure of WTAP bound to VIRMA (Su et al, 2022; PDB: 7VF2). Structural superposition reveals a notable difference in the spacing between the helices within the coiled-coil region. In the VIRMA-bound structure, the inter-helical distances are 5.9 Å, 7.7 Å, and 7.1 Å, whereas in the WTAP M-CC structure the corresponding distances are significantly larger (10.3 Å, 15.2 Å, and 15.9 Å) (Fig. EV2A). These observations suggest that VIRMA binding may induce a conformational rearrangement that brings the helices closer together and stabilizes the complex.

### A layered interface drives WTAP tetramerization

Detailed structural analysis identified a layered interaction interface mediating tetramer formation. This interface comprises alternating hydrophobic layers formed by residues such as Leu225, Leu229, and Leu236 (layers 3/2/1 and 9/10/11), while the dimer-dimer interface involves Met158, Leu211, Ile165, and Leu204 (layers 4/8 and 5/7) (Fig. 2D). These residues form a prominent hydrophobic patch between adjacent α-helices that plays a key role in stabilizing the tetrameric assembly (Figs. 2D and EV2B). In addition to these hydrophobic contacts, electrostatic interactions further contribute to stabilization, with Arg179 and Glu186 forming forming a salt bridge at the center of layer 6 between antiparallel helices (Fig. 2D). Together, these hydrophobic and electrostatic interactions define a robust interface that stabilizes the WTAP tetramer.

Importantly, these structural observations support a hierarchical assembly model in which the fundamental building block of WTAP is a dimer. Within this dimer, each monomer contributes two α-helices, forming a saddle-shaped architecture (Fig. EV2C–F). These dimeric units can further assemble into higher-order tetramers via their middle coiled-coil domains (residues 150–245). This model reconciles the distinct oligomerization properties of the N-terminal and middle regions and provides a structural framework for WTAP assembly.

### Targeted disruption of WTAP tetramerization converts it into a stable dimer

Guided by the crystallographic structures of WTAP, we designed a series of mutations to perturb its oligomeric state. FPLC-MALS analysis showed that charge-disrupting mutations at the electrostatic interface (R179A/E186A) preserved tetrameric assembly (Fig. 2E). In contrast, the M158R/L211R double mutant caused a pronounced shift toward aberrant higher-order species, indicating disruption of the native oligomeric organization (Fig. 2E). Most notably, introducing charged residues into the hydrophobic core (L225E/L229E, hereafter termed “L2E”) converted WTAP from a tetramer into a stable dimer, while maintaining high α-helical content as confirmed by CD spectroscopy (Figs. 2E and EV2H). This mutation also fully monomerized the isolated M-CC domain (Fig. EV2I).

Based on previous structural studies (Su et al, 2022; Yan et al, 2022), we infer that L2E mutation disrupts the central hydrophobic packing critical for tetramerization, while preserving the N-terminal dimerization interface. Furthermore, combining L2E with M158R/L211R abolished all higher-order oligomers, producing exclusively dimeric species (Fig. 2E). To assess these effects in cells, we expressed Flag-tagged WTAP and WTAP L2E in HEK293T cells. Native PAGE analysis revealed a predominant dimeric species for WTAP L2E, consistent with disruption of tetramerization (Fig. 2F,G), while SDS-PAGE showed monomeric migration at 55–70 kDa (Fig. EV2J,K). Together, these structure-guided biochemical analyses demonstrate that WTAP tetramerization critically depends on hydrophobic core residues within the middle coiled-coil region, and that targeted disruption yields a stable dimer while preserving secondary structure.

### Tetramerization of WTAP is essential for m^6^A writer complex assembly

**Figure 3:**
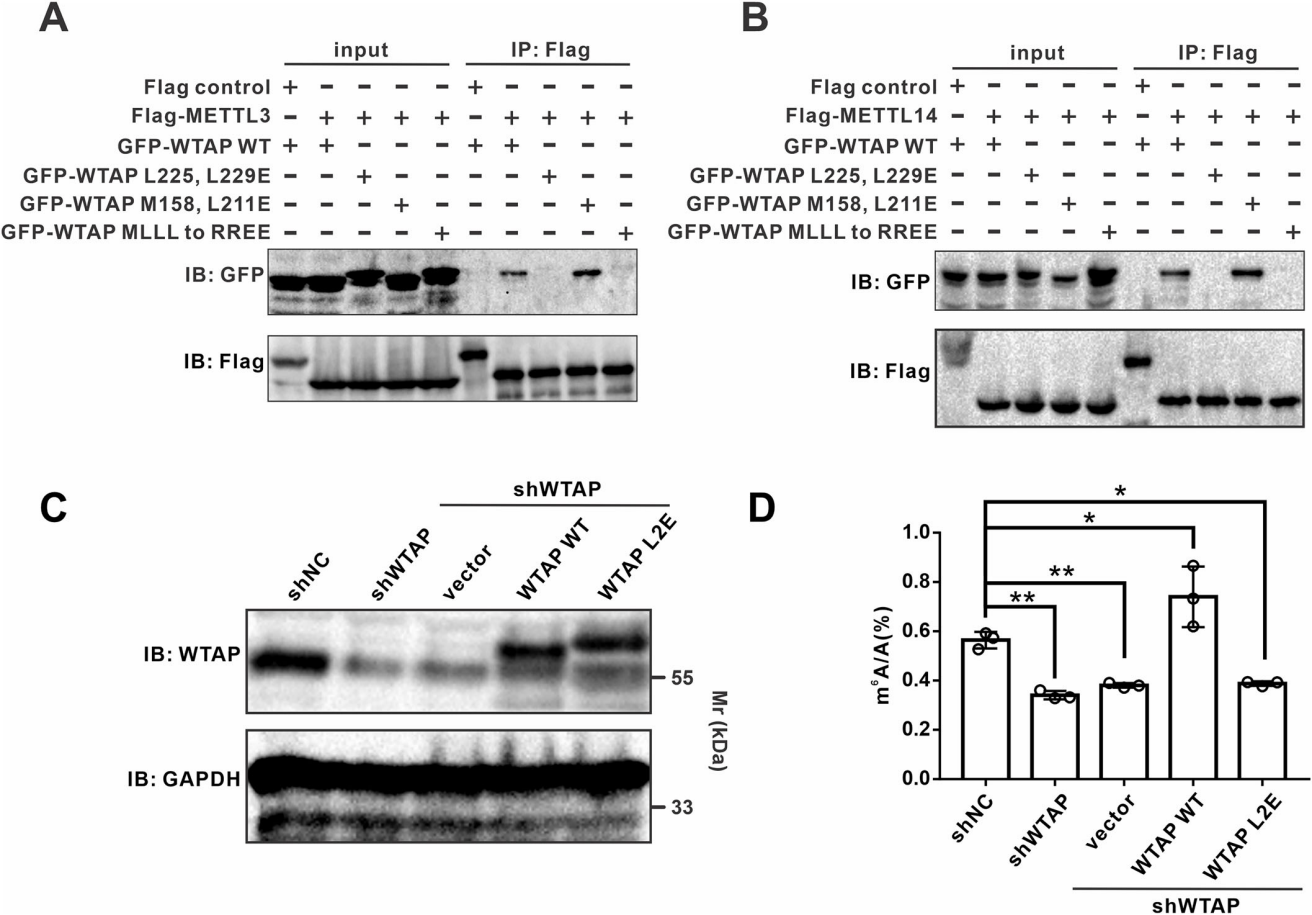
Tetrameric WTAP is required for assembly of the m^6^A writer complex and m^6^A deposition. (**A**, **B**) Co‑immunoprecipitation of Flag‑METTL3 (**A**) or Flag‑METTL14 (**B**) with GFP‑WTAP variants. WTAP WT binds METTL3/METTL14, whereas tetramer‑disrupting mutants (L2E and M158R/L211R/L225E/L229E) lose binding. (**C**) Western blot of WTAP knockdown and rescue lines. shRNA‑resistant WTAP WT restores protein expression; WTAP L2E expresses normally but fails to rescue function. (**D**) LC‑MS/MS quantification of the m^6^A/A ratio from purified mRNA. WTAP knockdown reduces m^6^A levels; WTAP WT but not L2E rescues methylation. Data are presented as mean ± SD (*n* = 3). Statistical significance was determined by one-way ANOVA followed by Dunnett’s multiple comparisons test, comparing each condition to the shNC control; **P < 0.01; *P < 0.05. Exact adjusted *P* values: shNC vs. shWTAP, *P* = 0.0027; shNC vs. shWTAP+vector, *P* = 0.0099; shNC vs. shWTAP+WTAP WT, *P* = 0.0132; shNC vs. shWTAP+WTAP L2E, *P* = 0.0128. Source data are available online for this figure.

Previous work has shown that WTAP interacts with both the m⁶A methyltransferase METTL3/METTL14 and the regulatory protein ZC3H13 (Little et al, 2000; Wen et al, 2018; Ping et al, 2014). To examine whether the tetrameric conformation of WTAP is required for writer complex assembly, we performed co-immunoprecipitation (Co-IP) assays in HEK293T cells co-expressing GFP-WTAP with Flag-tagged METTL3 or METTL14. Wild-type WTAP robustly co-precipitated with both METTL3 and METTL14 (Fig. 3A,B). In striking contrast, the L2E mutation—which converts WTAP into a stable dimer—completely abolished its association with both METTL3 and METTL14 (Fig. 3A,B), indicating that tetramerization is critical for complex formation. We next tested whether other interface mutations similarly affected WTAP–METTL3/14 interactions. The M158R/L211R double mutant retained binding to METTL3 and METTL14, whereas the quadruple mutant (M158R/L211R/L225E/L229E) nearly eliminated these interactions (Fig. 3A,B). These findings suggest that the hydrophobic layers forming the tetrameric core are essential for writer complex assembly.

**Figure EV3:**
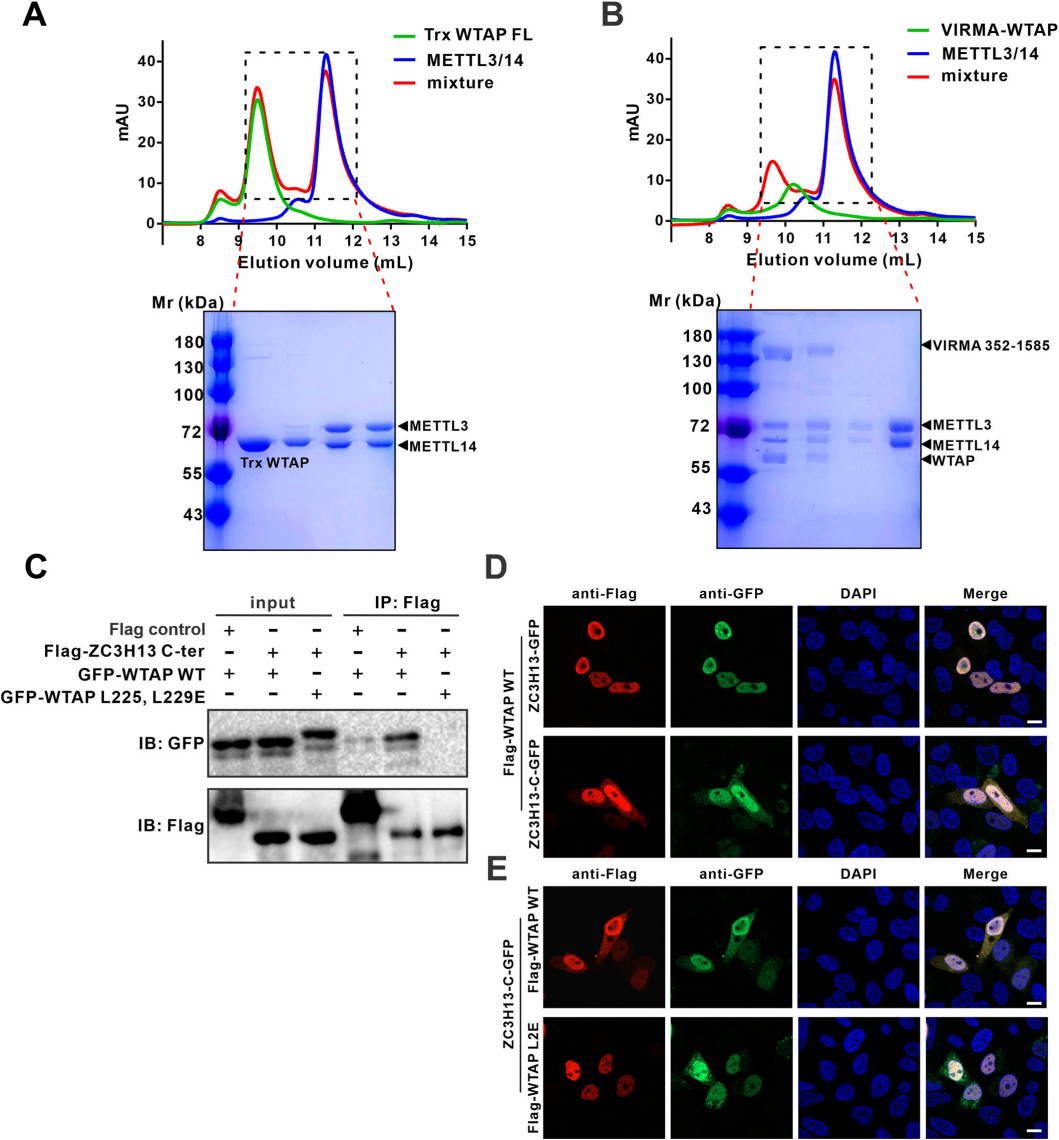
Coiled-coil tetramerization of WTAP is required for interaction with ZC3H13. (**A**) FPLC analysis (top) and SDS-PAGE analysis (bottom) of WTAP and METTL3/14. Distinct elution profiles and absence of co-elution indicate that WTAP does not directly interact with METTL3/14. (**B**) FPLC analysis (top) and SDS-PAGE analysis (bottom) of the WTAP-VIRMA complex and METTL3/14. Co-elution of WTAP, VIRMA, and METTL3/14 indicates that WTAP associates with METTL3/14 in a VIRMA-dependent manner. (**C**) Co-immunoprecipitation assays in HEK293T cells co-expressing Flag-tagged ZC3H13 C-terminal domain (residues 1460–1669) and GFP-tagged WTAP variants (WT or L2E). WTAP L2E fails to interact with ZC3H13. (**D**) Immunofluorescence imaging of HeLa cells co-expressing Flag-WTAP (red, Alexa Fluor 568) and ZC3H13-GFP (full-length or C-terminal). Full-length ZC3H13 co-localizes with WTAP in nuclear speckles, whereas the C-terminal fragment partially relocalizes WTAP to the cytoplasm. Nuclei were stained with DAPI. Scale bar: 10 μm. (**E**) HeLa cells co-expressing Flag-WTAP (WT or L2E) with ZC3H13-C-GFP. Compared to WTAP WT, the L2E mutant fails to colocalize with the ZC3H13 C-terminal fragment. Scale bar: 10 μm. Source data are available online for this figure.

However, size-exclusion chromatography using purified recombinant proteins showed that WTAP alone does not directly bind METTL3/14, and complex formation required the presence of VIRMA (Fig. EV3A,B), suggesting that VIRMA bridges the interaction between WTAP and the methyltransferases.

### ZC3H13 C-terminal interaction with WTAP depends on tetramerization

Previous studies have shown that WTAP interacts with ZC3H13 via its C-terminal region (Wen et al, 2018). Consistent with this, our Co-IP experiments confirmed that wild-type WTAP binds specifically to the C-terminal fragment of ZC3H13 (residues 1460–1669; Fig. EV3C), whereas the tetramer-disrupting L2E mutant showed no detectable interaction. Immunofluorescence analysis revealed that full-length ZC3H13 co-localizes with WTAP in nuclear speckles, consistent with previous reports. In contrast, the isolated C-terminal fragment of ZC3H13 exhibited both nuclear and cytoplasmic distribution and was sufficient to recruit WTAP to the cytoplasm (Fig. EV3D). Notably, WTAP L2E failed to be efficiently relocalized by the ZC3H13 C-terminal fragment and remained predominantly nuclear (Fig. EV3E), consistent with its impaired interaction observed in Co-IP assays. Together, these results indicate that WTAP tetramerization is required for efficient interaction with the ZC3H13 C-terminal region. Combined with our results on METTL3/METTL14 binding, these findings support a model in which the tetrameric architecture of WTAP provides a structural platform for assembly of the m^6^A writer complex.

### Tetramerization of WTAP is required for m^6^A deposition on mRNA

As a core component of the m^6^A writer complex, WTAP plays a central role in mediating methylation of target transcripts. To assess the functional significance of its tetrameric assembly, we examined m^6^A levels in WTAP-depleted cells. Stable knockdown of WTAP in HeLa cells using specific shRNAs resulted in a marked decrease in m^6^A levels, as measured by LC-MS/MS analysis of purified mRNA (Fig. 3C,D). Re-expression of shRNA-resistant wild-type WTAP largely rescued m^6^A methylation, whereas neither the L2E mutant nor empty vector restored m^6^A levels (Fig. 3C,D). These findings demonstrate that proper oligomerization via the coiled-coil domain is essential for WTAP’s role in m^6^A deposition.

### WTAP tetramer ensures mitotic fidelity by regulating chromosome segregation

**Figure 4:**
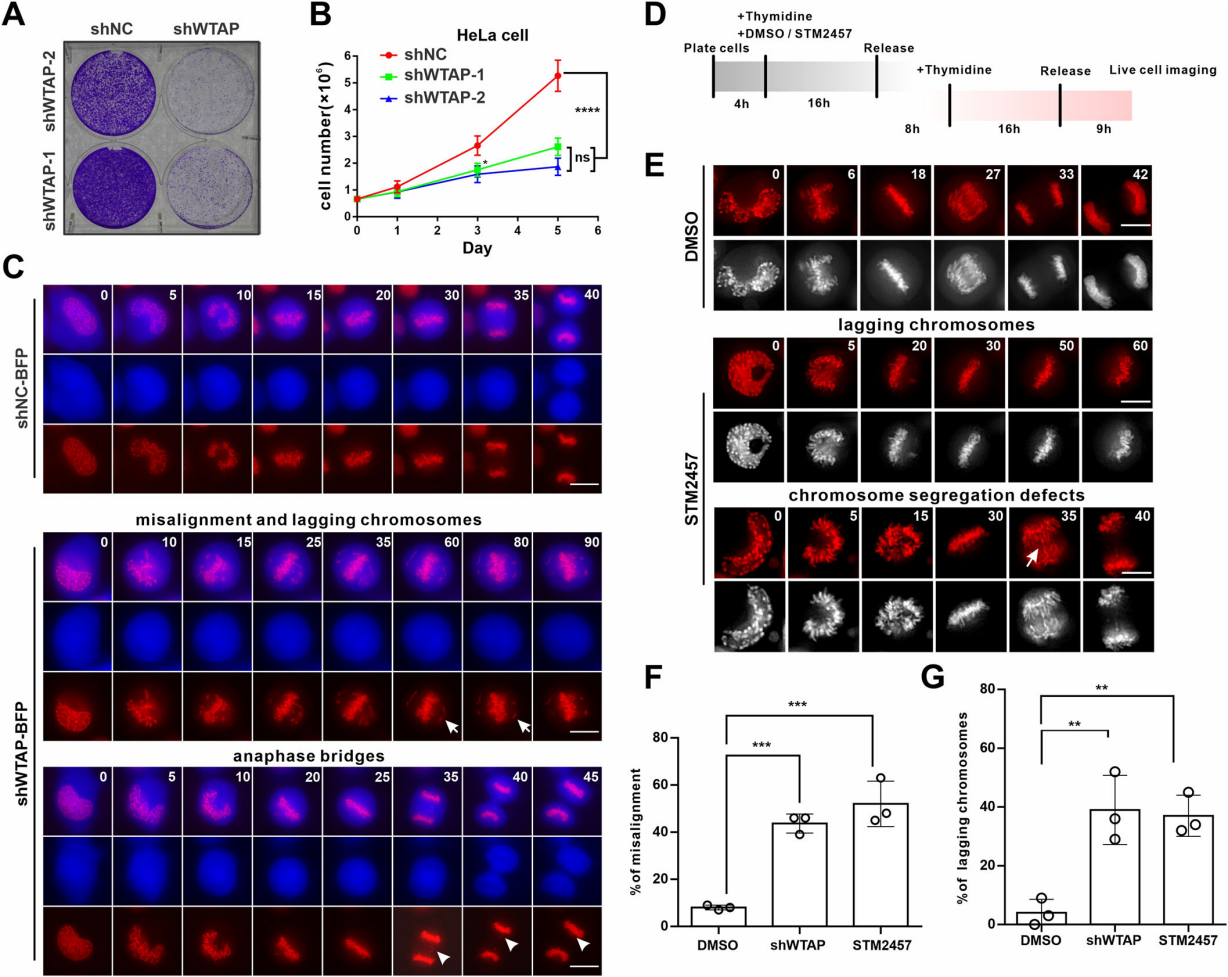
WTAP tetramer ensures accurate chromosome segregation during mitosis. (**A**) Crystal violet staining showing reduced colony formation upon WTAP knockdown. (**B**) Cell growth curves of WTAP-knockdown and control HeLa cells measured daily. Data are presented as mean ± SD (*n* = 3). Data were analyzed using two-way ANOVA followed by Sidak’s multiple comparisons test; ns, not significant; ***P < 0.001. Exact adjusted *P* values: shNC vs. shWTAP-1 and vs. shWTAP-2, *P* < 0.0001; shWTAP-1 vs. shWTAP-2, *P* = 0.1847 (not significant). (**C**) Time-lapse microscopy of HeLa cells expressing shWTAP or control shRNA, with chromosomes labeled by mCherry-H2B. Bold arrows indicate chromosome misalignment, and arrowhead indicate anaphase bridges. Time (min) is indicated in the top right corner. BFP marks the WTAP shRNA-transfected cells. Scale bars: 10 μm. (**D**) Schematic diagram of the experimental design. (**E**) Representative time-lapse images of HeLa cells treated with DMSO (top) or STM2457 (bottom), with chromosomes labeled by mCherry-H2B. Arrows indicate chromosome segregation defects. Time (min) is indicated in the top right corner. Scale bar: 10  μm. (**F**) Quantification of chromosome misalignment defects in live HeLa cells. Data are presented as mean ± SEM (*n* = 3). Statistical significance was determined by one-way ANOVA followed by Dunnett’s multiple comparisons test; ***P < 0.001. Exact adjusted *P* values: DMSO vs. shWTAP, *P* = 0.0007; DMSO vs. STM2457, *P* = 0.0002. (**G**) Quantification of lagging chromosomes in live HeLa cells. Data are presented as mean ± SEM (*n* = 3). Statistical significance was determined by one-way ANOVA followed by Dunnett’s multiple comparisons test; **P < 0.01. Exact adjusted *P* values: DMSO vs. shWTAP, *P* = 0.0039; DMSO vs. STM2457, *P* = 0.0052. Source data are available online for this figure.

**Figure EV4:**
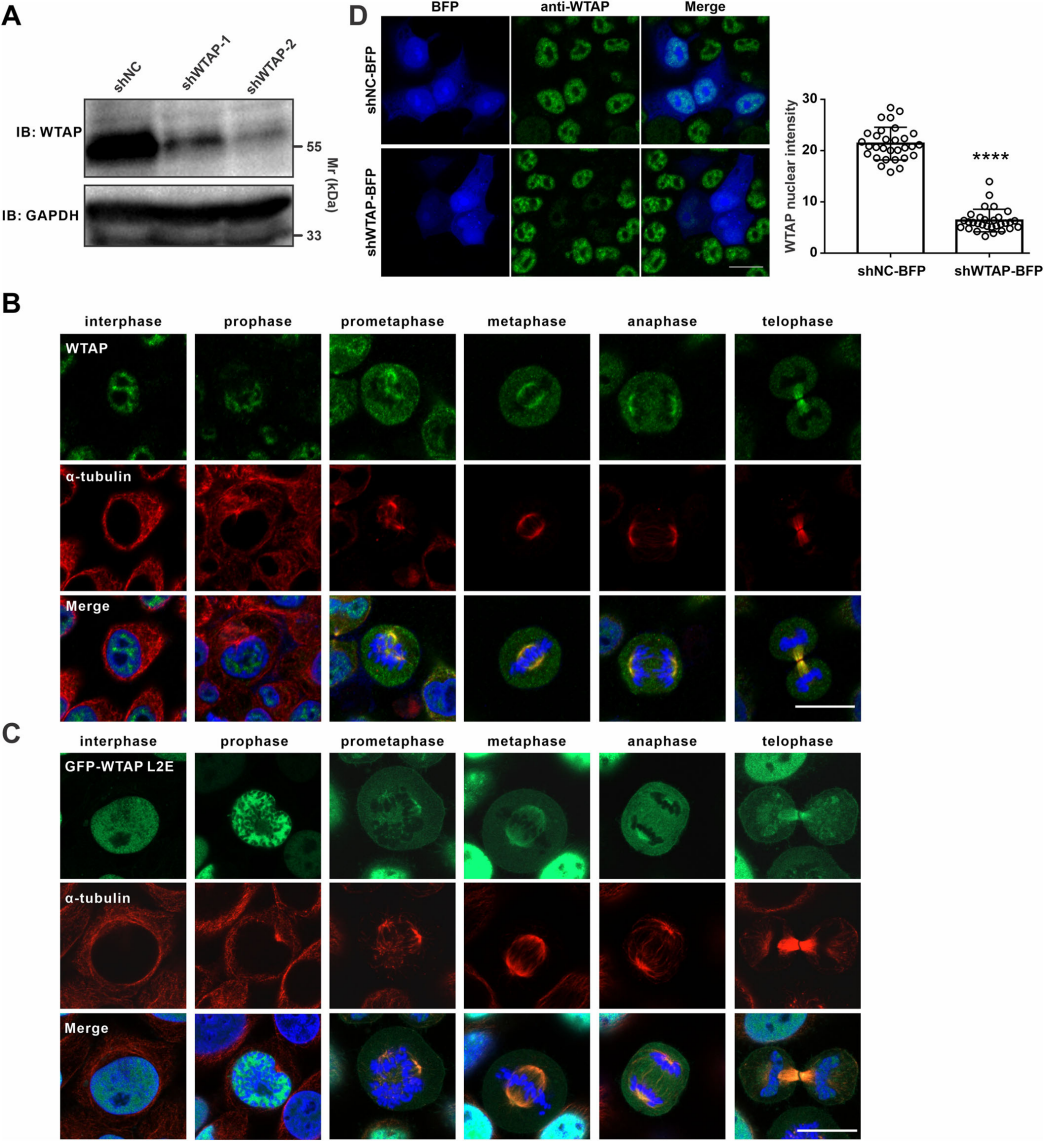
WTAP knockdown efficiency and dynamic mitotic localization. (**A**) Immunoblot analysis of WTAP knockdown in HeLa cells using two independent shRNAs. GAPDH serves as a loading control. (**B**) Immunofluorescence imaging of endogenous WTAP (green) and α-tubulin (red) across mitotic stages. WTAP redistributes from the nucleus to mitotic spindles upon mitotic entry. Scale bar: 20 μm. (**C**) Immunofluorescence imaging of HeLa cells stably expressing GFP-tagged WTAP L2E at different mitotic stages. Cells were stained with α-tubulin to visualize the spindle. Scale bar: 20 μm. (**D**) Fluorescence imaging of HeLa cells transfected with BFP-marked shWTAP or shNC (left). The right panel shows quantification of WTAP fluorescence intensity. Data are presented as mean ± SD (*n* = 30). Statistical significance was determined using a two-tailed unpaired Student’s *t* test: ****P < 0.0001. Scale bar: 20 μm. Source data are available online for this figure.

WTAP knockdown has previously been reported to induce G2/M arrest and impair cell proliferation (Horiuchi et al, 2006). Consistent with these findings, we observed reduced proliferation in WTAP-depleted HeLa cells compared to controls (Figs. 4A,B and EV4A). To investigate WTAP’s role during mitosis, we examined its subcellular localization throughout the cell cycle. During interphase, WTAP localized predominantly to the nucleus; however, upon mitotic entry, it dispersed into the cytoplasm and accumulated at microtubule (MT) asters (Fig. EV4B). Notably, the tetramer-disrupting L2E mutant exhibited a similar localization pattern (Fig. EV4C), indicating that WTAP tetramerization is not required for its mitotic redistribution or spindle association. This observation suggests that the mitotic defects caused by the L2E mutation are unlikely to arise from altered subcellular localization.

To further probe WTAP function during mitosis, we performed time-lapse imaging of WTAP-depleted cells. Knockdown of WTAP significantly delayed anaphase onset—from 32 min in control cells to over 90 min (Fig. 4C)—and resulted in a high frequency of mitotic defects, including chromosome misalignment and anaphase bridges (Figs. 4C,F,G and  EV4D). To determine whether these defects are linked to loss of m^6^A methyltransferase activity, rather than m^6^A-independent functions of WTAP, we treated cells with STM2457 (Yankova et al, 2021), a selective catalytic inhibitor of METTL3. Acute inhibition of METTL3 activity led to a marked increase in mitotic defects, including lagging chromosomes and chromosome mis-segregation (Fig. 4D–G), closely phenocopying the effects observed upon WTAP depletion. These results support the idea that m^6^A methylation activity is required for proper mitotic progression.

**Figure 5:**
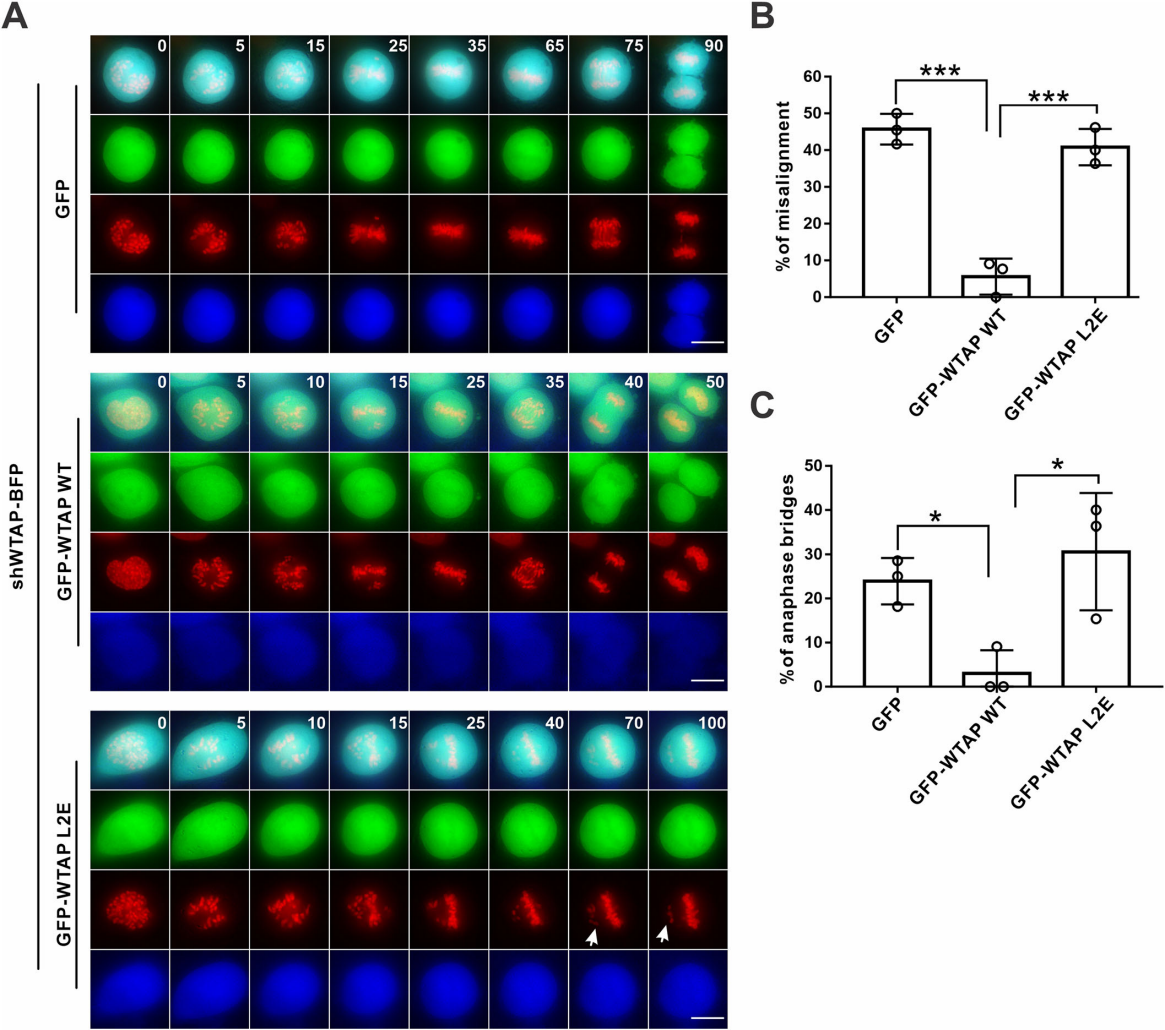
Tetrameric WTAP rescues chromosome segregation defects. (**A**) Time-lapse microscopy of WTAP-knockdown HeLa cells re-expressing GFP, GFP-tagged WTAP WT, or GFP-tagged WTAP L2E. Chromosomes were visualized using mCherry-H2B (red). Arrows indicate chromosome misalignment. BFP marks cells transfected with WTAP shRNA. Time (min) is indicated on each frame. Scale bar: 10 μm. (**B**) Quantification of chromosome misalignment defects in shWTAP-BFP HeLa cells following re-expression of GFP, GFP-tagged WTAP WT, or GFP-tagged WTAP L2E. Data are presented as mean ± SEM (*n* = 3). Statistical significance was determined by one-way ANOVA with Dunnett’s test; ***P < 0.001. Exact adjusted *P* values: GFP vs. WT, *P* = 0.0004; WT vs. L2E, *P* = 0.0002. (**C**) Quantification of anaphase bridge defects in shWTAP-BFP HeLa cells following re-expression of GFP, GFP-tagged WTAP WT, or GFP-tagged WTAP L2E. Data are presented as mean ± SEM (*n* = 3). Statistical significance was determined by one-way ANOVA followed by Dunnett’s multiple comparisons test; *P < 0.05. Exact adjusted *P* values: GFP vs. WT, *P* = 0.0471; WT vs. L2E, *P* = 0.0152. Source data are available online for this figure.

Importantly, these phenotypes were specifically rescued by re-expression of shRNA-resistant wild-type WTAP, but not by empty vector or the L2E mutant (Fig. 5A–C), indicating that tetramerization is required for this function. Collectively, these data demonstrate that WTAP tetramerization is essential for maintaining mitotic fidelity, likely through its role in supporting m^6^A-dependent regulation of mitotic gene expression.

### WTAP tetramer maintains mitotic gene expression by stabilizing m^6^A-modified transcripts

**Figure EV5:**
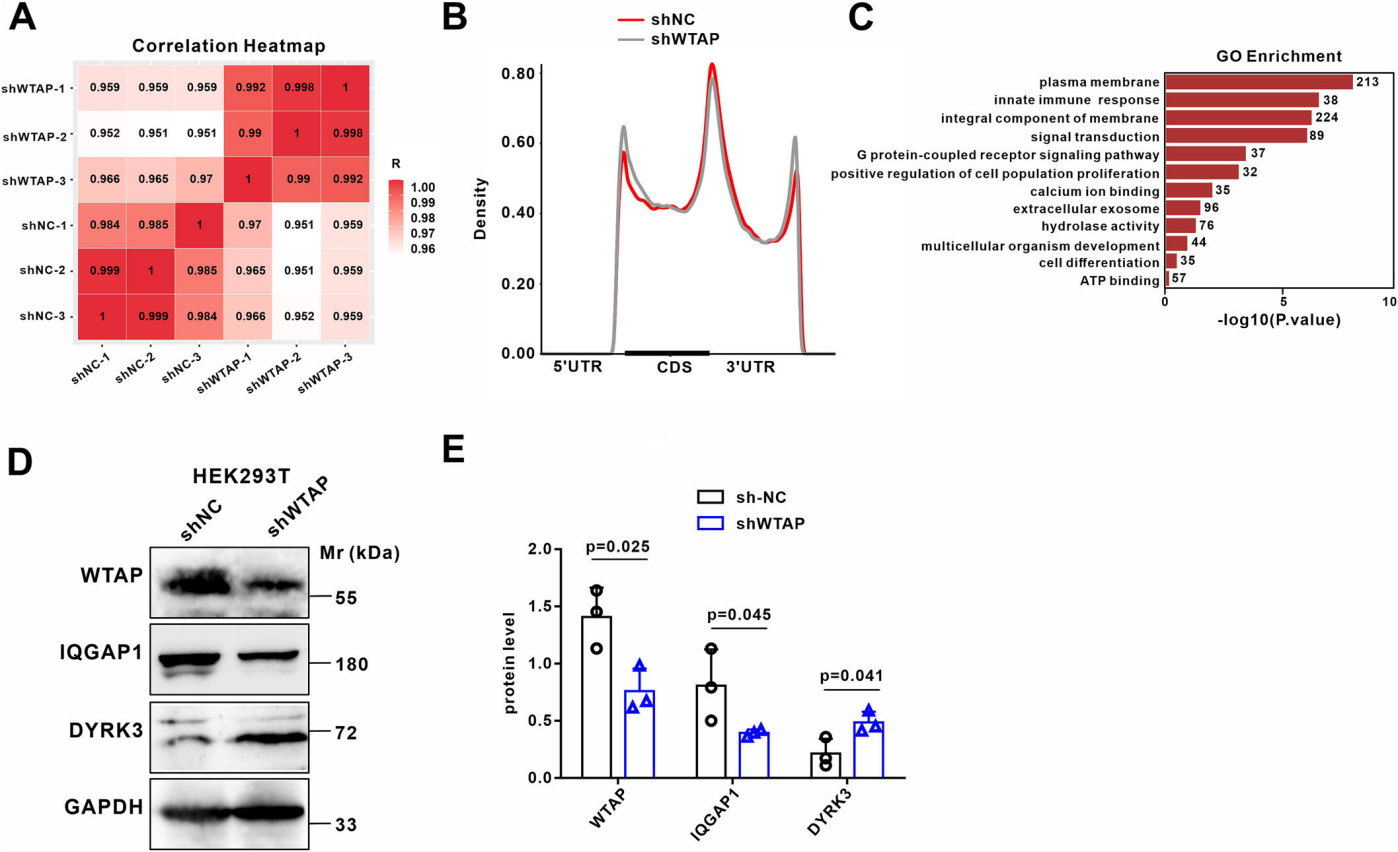
WTAP-dependent m^6^A regulation of mitotic transcripts. (**A**) Heatmap showing Pearson correlation coefficients (R) between samples, indicating reproducibility of biological replicates. (**B**) Metagene analysis showing distribution of m^6^A peaks across mRNA regions in WTAP knockdown (gray) and control (red) cells based on MeRIP-seq. (**C**) Gene Ontology (GO) enrichment analysis of differentially expressed genes from RNA-seq, highlighting pathways related to cell signaling and mitotic progression. Statistical significance was assessed using a one‑sided hypergeometric test with Benjamini–Hochberg FDR correction. (**D**) Immunoblot validation of protein expression changes (IQGAP1and DYRK3) upon WTAP knockdown in HEK293T cells. (**E**) Quantification of protein expression changes from (**D**). Data are presented as mean ± SD from three biological replicates. Band intensities were normalized to GAPDH. Statistical significance was determined using a two-tailed unpaired Student’s *t* test. Source data are available online for this figure.

**Figure 6:**
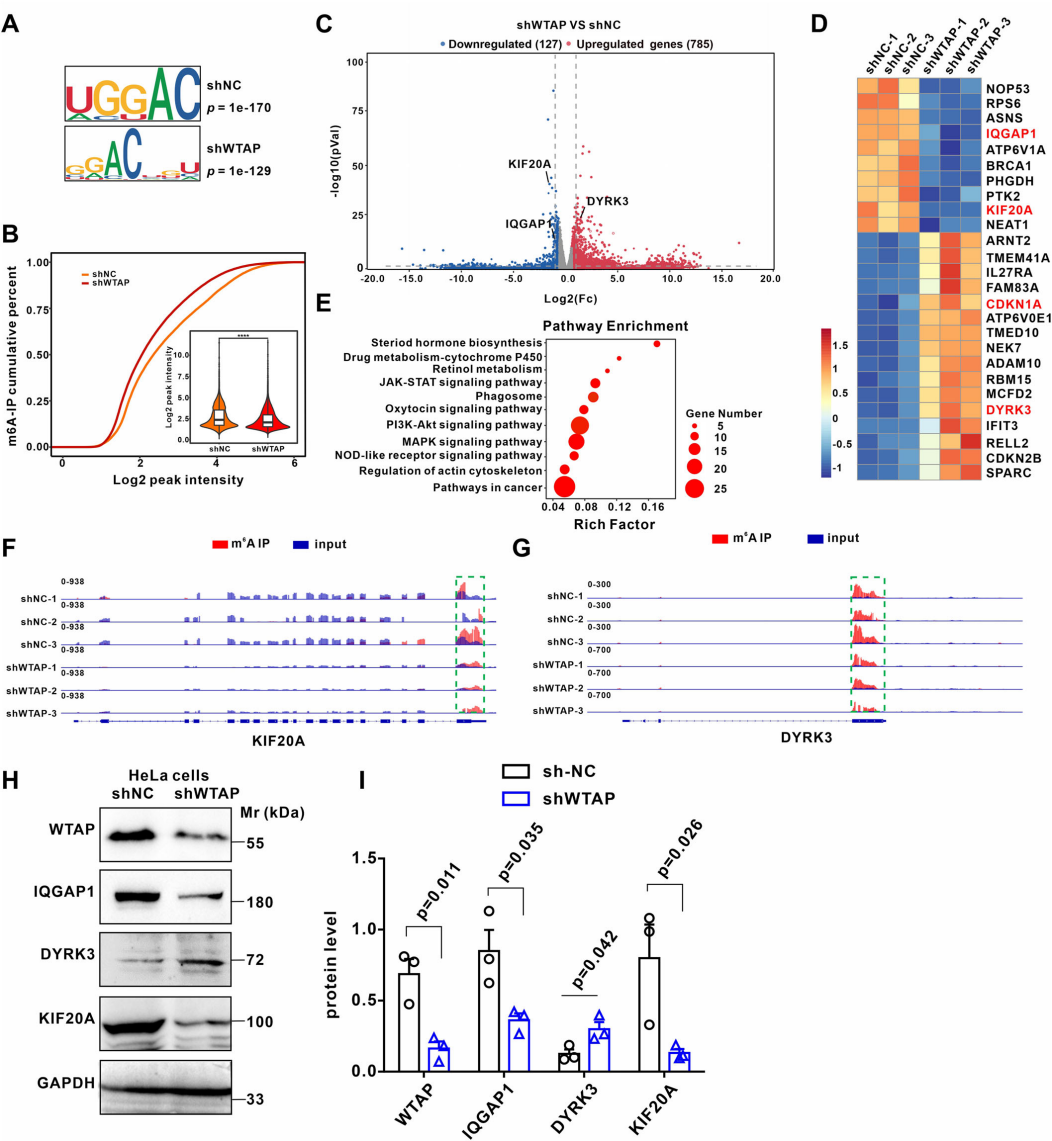
WTAP tetramer maintains m^6^A‑dependent expression of mitotic regulators. (**A**) Sequence motif analysis of m^6^A peaks from MeRIP-seq reveals enrichment of the canonical RRACH motif (R = A/G; H = A/C/U) in both control and WTAP-knockdown HeLa cells, confirming specificity of m^6^A deposition. Motif enrichment was performed using HOMER software. *P* values were calculated based on hypergeometric enrichment statistics. (**B**) Cumulative distribution of m^6^A enrichment in shNC and shWTAP cells. m^6^A-IP signals were normalized to input to calculate enrichment (shNC, *n* = 37,377; shWTAP, *n* = 42,769). For each box plot, the center line represents the median, the box bounds represent the first and third quartiles (Q1 and Q3), and the whiskers extend to the minimum and maximum values (excluding outliers). Statistical significance was assessed using a two-sided Wilcoxon rank-sum test, ****P < 0.0001. (**C**) Volcano plot showing differentially expressed genes upon WTAP knockdown compared to control cells, including 785 upregulated (red) and 127 downregulated (blue) genes. RNA-seq data were analyzed using DESeq2. Statistical significance was assessed using a negative binomial distribution model with Wald test. (**D**) Heatmap of selected cell cycle–associated genes significantly downregulated in WTAP-knockdown cells relative to controls. Three biological replicates are shown. (**E**) KEGG pathway enrichment analysis of differentially expressed genes in WTAP-knockdown versus control cells. (**F**, **G**) Genome browser views of m^6^A-IP and input sequencing reads at the KIF20A (**F**) and DYRK3 (**G**) loci. Tracks include input (control), three shNC replicates, and three shWTAP replicates. m^6^A-IP peaks (red) present in shNC samples are markedly reduced in shWTAP samples, indicating site-specific loss of m⁶A modification upon WTAP knockdown. (**H**) Immunoblot analysis showing that WTAP knockdown reduces the expression of key mitotic regulators IQGAP1 and KIF20A, with modest upregulation of DYRK3 in HeLa cells. GAPDH serves as a loading control. (**I**) Quantification of normalized band intensities from (**H**) based on three independent biological replicates. Data are presented as mean ± SD. Statistical significance was determined using a two-tailed unpaired Student’s *t* test. Source data are available online for this figure.

To elucidate the molecular basis of WTAP-dependent proliferation defects, we performed MeRIP-seq in HeLa cells with and without WTAP knockdown. High-quality sequencing data were confirmed by mapping statistics and reproducibility analysis (Table EV2 and Fig. EV5A). We identified 24,942 common m^6^A peaks consistently detected across replicates (Dataset EV1), with strong enrichment of the canonical “RRACH” motif (R = A/G, H = A/C/U) (Fig. 6A). These peaks were predominantly distributed within coding sequences and 3’ UTRs (Figure EV5B), consistent with previous reports. Global analysis revealed a systematic reduction in m^6^A peak intensity upon WTAP depletion (Fig. 6B). This trend was further supported by representative gene tracks, which showed a marked loss of m^6^A enrichment in WTAP knockdown samples compared to controls (Fig. 6F,G).

To assess the transcriptional consequences of WTAP depletion, we performed RNA-seq analysis, identifying 785 upregulated and 127 downregulated genes (Fig. 6C). Gene Ontology (GO) analysis of these transcripts showed enrichment in biological processes such as signal transduction and cell differentiation (Fig. EV5C). KEGG pathway enrichment further revealed that WTAP-regulated genes are involved in proliferation-associated signaling pathways (Zhang and Liu, 2002; Whitaker and Cook, 2021), including MAPK and PI3K-Akt cascades, and cytoskeletal regulation (Fig. 6E).

Notably, a subset of cell cycle-related genes was significantly downregulated upon WTAP depletion, as shown by heatmap clustering (Fig. 6D). Among these, IQGAP1, a key regulator of cell growth and division (Wang et al, 2009; Johnson et al, 2011), showed reduced protein expression following WTAP knockdown (Figs. 6H,I and  EV5D,E). Similarly, KIF20A, a mitotic regulator involved in chromosome segregation (Wang et al, 2017; Fontijn et al, 2001), exhibited both reduced m^6^A enrichment on its transcript and decreased protein levels (Fig. 6F,H,I), supporting a link between m^6^A modification and gene expression output. In contrast, some transcripts with reduced m^6^A levels, such as DYRK3, displayed moderate upregulation (Figs. 6G–I and  EV5D,E), indicating that m^6^A-dependent regulation may exert transcript-specific effects.

Taken together, these results support a model in which WTAP tetramerization promotes m^6^A deposition on a subset of mitotic regulators, thereby contributing to their proper expression. This mechanism likely underlies, at least in part, the defects in chromosome segregation and cell proliferation observed upon disruption of WTAP tetramerization.

## Discussion

In this study, we identify tetramerization as a key organizational principle underlying WTAP function within the m^6^A writer complex. Our structural and biochemical analyses reveal that WTAP forms a dimeric building block that further assembles into a higher-order tetramer via its middle coiled-coil domain. Moreover, we show that this oligomeric state is essential for the functional role of WTAP within the m^6^A writer complex. Through biochemical and crystallographic analyses, we resolved the structural basis of WTAP tetramerization, identifying a layered interaction interface composed of hydrophobic and electrostatic contacts. Disruption of this tetrameric interface abolishes WTAP interactions with METTL3, METTL14, and ZC3H13, and impaired its ability to restore m^6^A deposition and cell proliferation in knockdown cells. These results establish that tetramerization is not a merely structural feature, but a functional requirement for WTAP to scaffold and organize the writer complex.

WTAP has long been considered as a critical component of the m^6^A machinery, yet its precise molecular role remained incompletely understood. Our findings provide a structural framework in which WTAP tetramerization generates a multivalent platform that enables coordinated engagement of catalytic and regulatory components. This model is consistent with previous observations that WTAP promotes METTL3 localization to nuclear speckles and facilitates efficient m^6^A deposition (Horiuchi et al, 2013; Ping et al, 2014). Importantly, the inability of tetramer-disrupting mutants to support these interactions highlights the functional importance of higher-order assembly in maintaining writer complex integrity.

Functionally, we show that WTAP tetramerization contributes to global m^6^A deposition on mRNA. Integrated MeRIP-seq and RNA-seq analyses revealed that WTAP depletion leads to reduced m^6^A modification on a subset of transcripts, accompanied by altered gene expression. Among these, KIF20A—a key regulator of chromosome segregation and cytokinesis (Wu et al, 2019; Wang et al, 2017)—exhibited reduced m^6^A enrichment together with decreased protein levels, supporting a link between m^6^A modification and gene expression output. At the same time, we note that m^6^A-dependent regulation is likely transcript-specific, as some targets display distinct expression responses despite reduced methylation. These findings support a model in which WTAP tetramerization promotes m^6^A-dependent expression of mitotic regulators, thereby contributing to proper cell cycle progression.

In addition to its role in m^6^A deposition, we observed that WTAP dynamically redistributes during mitosis and associates with spindle structures. Disruption of WTAP tetramerization does not affect this localization but leads to pronounced defects in chromosome segregation, including delayed anaphase onset and increased segregation errors. Notably, pharmacological inhibition of METTL3 recapitulates these phenotypes, supporting the idea that m^6^A methyltransferase activity contributes to mitotic fidelity. However, given that WTAP has also been reported to localize to mitotic structures, we cannot exclude the possibility that m^6^A-independent mechanisms may additionally contribute to this process. Thus, WTAP likely integrates both structural scaffolding and regulatory functions to ensure accurate cell division.

In addition to defining the structural and functional importance of WTAP tetramerization, an important open question is whether this oligomeric state is dynamically regulated in cells. Our biochemical data indicate that WTAP predominantly exists as a tetramer under dilute conditions, while a minor dimeric population can also be detected, suggesting a potential equilibrium between different oligomeric states. Although our current study does not directly address regulatory mechanisms governing this equilibrium, it is conceivable that cellular signals, such as post-translational modifications, cell cycle–dependent cues, or interactions with binding partners including VIRMA, may modulate WTAP assembly and thereby influence writer complex activity. Consistent with this idea, our structural comparison with the VIRMA-bound WTAP complex suggests that partner binding can induce conformational changes within the coiled-coil domain. Future studies will be required to determine whether regulated transitions between dimeric and tetrameric states of WTAP contribute to dynamic control of m^6^A deposition in response to cellular signals.

WTAP overexpression has been reported in a variety of cancers, including hepatocellular carcinoma, acute myeloid leukemia, and osteosarcoma (Frye et al, 2018; Huang et al, 2022), where it correlates with enhanced proliferation and poor prognosis. Our structural and functional analyses provide a mechanistic basis for this observation, suggesting that tetrameric assembly of WTAP supports efficient writer complex formation and sustained m^6^A-dependent gene expression programs that promote cell cycle progression. These findings raise the possibility that targeting WTAP tetramerization could represent a strategy to modulate m^6^A deposition in disease contexts. More broadly, our study highlights how higher-order assembly of scaffold proteins can regulate the organization and activity of epitranscriptomic machinery. By linking quaternary structure to RNA modification and cellular function, our work provides a framework for understanding how structural features of writer complex components contribute to gene regulatory control.

## Methods

**Table:** Reagents and Tools Table

Reagent/resource	Reference or source	Identifier or catalog number
**Experimental models**		
HEK-293 cells (*H. sapiens*)	ATCC	CBP60439
Hela	ATCC	CRM-CCL-2
**Recombinant DNA**		
pET32m3c-WTAP-FL	This study	N/A
pET32m3c-WTAP-FL L2E	This study	N/A
pET32m3c-WTAP-FL M158R/L211R	This study	N/A
pET32m3c-WTAP-FL R179A/E186A	This study	N/A
pET32m3c-WTAP-FL L225/L229/M158R/L211R	This study	N/A
pET32m3c-WTAP-F1	This study	N/A
pET32m3c-WTAP-F2	This study	N/A
pET32m3c-WTAP-F3	This study	N/A
pET32m3c-WTAP-F4	This study	N/A
pET MBP-WTAP-1–50	This study	N/A
pET MBP-WTAP-1–50 W30R	This study	N/A
pCMV-Flag-WTAP	This study	N/A
pCMV-Flag-WTAP L2E	This study	N/A
pEGFP-WTAP	This study	N/A
pEGFP-WTAP L2E	This study	N/A
pEGFP-WTAP M158R/L211R	This study	N/A
pEGFP-WTAP L225/L229/M158R/L211R	This study	N/A
pCMV-Flag-METTL3	Liu et al, 2014	N/A
pCMV-Flag-METTL14	Liu et al, 2014	N/A
pCMV-Flag-ZC3H13	This study	N/A
pEGFPN1-ZC3H13	This study	N/A
pEGFPN1-ZC3H13-C	This study	N/A
BFP-plko3.7	This study	N/A
BFP-WTAP-shRNA	This study	N/A
plko-WTAP	This study	N/A
**Antibodies**		
Mouse anti-DYKDDDDK	Proteintech	66008-4-Ig
Rabbit anti-GFP	Proteintech	50430-2-AP
Mouse anti-WTAP	Proteintech	60188-1-Ig
Rabbit anti-GAPDH	Proteintech	10494-1-AP
Mouse anti-α-tubulin	Sigma-Aldrich	T9026
Mouse anti-KIF20A	Santa Cruz	sc-374508
Mouse anti-DYRK3	Invitrogen	PA5-40341
Rabbit anti-IQGAP1	Huaan Biotechnology	HA721972
Goat anti-rabbit IgG-HRP	Absin	J0609A01
Goat anti-mouse IgG-HRP	Absin	D1230A01
568 goat anti-rabbit IgG	Invitrogen	2277758
488 goat anti-mouse IgG	Invitrogen	2415945
**Oligonucleotides and other sequence-based reagents**		
PCR primers	This study	Table EV3
**Chemicals, enzymes and other reagents**		
DMEM F-12	ThermoFisher	11320033
Trypsin	ThermoFisher	15090046
Fetal Bovine Serum	Excell Bio	FSP500
Penicillin–streptomycin	HyClone	SV30010
Opti-MEM (1×)	ThermoFisher	31985.70
4% Paraformaldehyde Fix Solution	Sangon Biotech	E672002
Crystal violet solution	Biosharp	BL802A
thymidine	Sigma-Aldrich	T9250
Triton X-100	Sangon Biotech	A600198
Tween-20	Sangon Biotech	A600560
NP-40 Lysis Buffer	Beyotime	P0013F
TRIzol	Invitrogen	15596026CN
polyethyleneimine	Polyscience	23966-1
Lipofectamine 2000	Invitrogen	11668019
STM2457	Sigma-Aldrich	SML3360-5MG
NON-FAT Powdered Milk	Sangon Biotech	A600669-0250
2×Phanta Master Mix	Vazyme	P525-01
ClonExpress Ⅱ One Step Cloning Kit	Vazyme	C112-02
10×DNA loading buffer	Vazyme	P022-01
DNA marker	Vazyme	MD102-02
BamHI-HF	NEB	R3136L
EcoRI-HF	NEB	R3101L
Xhol	NEB	R0146L
CutSmart	NEB	B7204S
IPTG	Sangon Biotech	A600168-0100
DTT	Sangon Biotech	A620058-0100
**Software**		
GraphPad Prism	https://www.graphpad.com/	
ImageJ	https://imagej.nih.gov/ij/index.html	
PyMOL	https://pymol.org/2/	
**Other**		
ZEISS LSM980 with Airyscan	ZEISS	
VP-ITC	Malvern	
Tanon 5200 Multi	Tanon	
NanoDrop ND-1000	Thermo Fisher Scientific	

### Cloning

Full-length human WTAP cDNA (UniProt ID: Q15007), along with various truncated fragments, was subcloned into a modified pET32a expression vector containing an N-terminal thioredoxin (Trx) and hexahistidine (His_6_) affinity tag for bacterial protein expression and purified protein studies. Site-directed mutagenesis was performed to generate WTAP point mutants. All constructs were verified by DNA sequencing. Primer sequences used in this study are listed in Table EV3. For mammalian expression and cell-based assays, cDNAs encoding human WTAP, ZC3H13 (UniProt ID: Q5T200), VIRMA (UniProt ID: Q69YN4), METTL3 (UniProt ID: Q86U44), and METTL14 (UniProt ID: Q9HCE5), were cloned into the pCDNA3.1 vector containing an N-terminal Flag tag, or into the pEGFP-C3 vector containing an N-terminal GFP tag. For recombinant complex expression in Expi293F cells, VIRMA (residues 352–1585) and WTAP were subcloned into a modified vector containing an N-terminal Twin-Strep tag and an HRV 3 C protease cleavage site. All constructs were verified by sequencing prior to use.

### Protein expression and purification

Recombinant WTAP full-length and truncated proteins were expressed in *Escherichia coli* BL21(DE3) cells. Cultures were grown at 37 °C until reaching an OD_600_ of 0.6, then induced with 0.2 mM isopropyl-β-D-thiogalactopyranoside (IPTG) and incubated at 16 °C for 18 h. Cells were harvested by centrifugation and resuspended in lysis buffer containing 500 mM NaCl, 20 mM Tris-HCl (pH 7.8), and 5 mM imidazole. Cell lysis was performed using high-pressure homogenization. The resulting lysate was clarified by centrifugation and loaded onto a Ni-NTA agarose affinity column. Eluted proteins were further purified by size-exclusion chromatography (Superdex 200 Increase 10/300 GL, GE Healthcare) in buffer containing 100 mM NaCl, 50 mM Tris-HCl (pH 7.8), 1 mM EDTA, and 1 mM DTT.

For selenomethionine (SeMet)-labeled WTAP (residues 150–245), expression was performed in E. coli B834(DE3) cells cultured in M9 minimal medium supplemented with 4 g/L glucose, 5 mg/L vitamin B1, 2 mM MgSO_4_, 0.1 mM CaCl_2_, 100 mg/L each of lysine, threonine, phenylalanine, and selenomethionine, and 50 mg/L each of leucine, isoleucine, and valine. Cultures were induced with 0.2 mM IPTG and incubated at 16 °C for 18 h. SeMet-labeled proteins were purified using the same Ni-NTA and gel filtration protocol as above, using a Superdex 200 26/600 column (GE Healthcare). For crystallization, the N-terminal Trx-His_6_ tag was cleaved by incubation with HRV 3 C protease at 4 °C overnight, followed by a second round of size-exclusion chromatography to isolate the tag-free protein.

For mammalian expression, the VIRMA-WTAP complex and the METTL3/METTL14 heterodimer (with Strep-tagged METTL14) were expressed in Expi293F cells. For a 100 mL culture, 100 μg of plasmid DNA was mixed with 400 μg linear polyethyleneimine in 10 mL Opti-MEM, incubated for 20 min at room temperature, and then added to cells at a density of ~2 × 10^6^ cells/mL. Cells were cultured at 37 °C for 72 h and harvested for purification. The complex was purified by Strep-Tactin affinity chromatography followed by size-exclusion chromatography.

### FPLC-MALS analysis

Oligomeric states and absolute molecular weights of WTAP and its truncated constructs were analyzed using fast protein liquid chromatography coupled with multi-angle light scattering (FPLC-MALS). The system consisted of an AKTA Pure chromatography unit (GE Healthcare), a miniDAWN TREOS MALS detector (Wyatt Technology), and an Optilab T-rEX differential refractive index (dRI) detector (Wyatt Technology). Protein samples (typically at 100 μM) were filtered through 0.22 μm syringe filters prior to injection and loaded onto a Superdex 200 Increase 10/300 GL column (GE Healthcare) pre-equilibrated with buffer containing 100 mM NaCl, 50 mM Tris-HCl (pH 7.8), 1 mM EDTA, and 1 mM DTT. The column was operated at a flow rate of 0.5 mL/min at room temperature. Data acquisition and molecular weight calculations were performed using ASTRA 7 software (Wyatt Technology).

### Circular dichroism measurements

Circular dichroism (CD) spectra were recorded at room temperature using a Chirascan^TM^-plus CD spectrometer. Full-length WTAP and its truncated fragments (WTAP 1–245, 1–150, 150–245, and 246–396) were purified, cleaved to remove the affinity tag, and diluted to 20 μM in CD buffer containing 25 mM Tris-HCl (pH 7.8), 50 mM NaCl, 0.5 mM EDTA, and 0.5 mM DTT. Samples were transferred to a quartz cuvette with a 1 mm path length, and spectra were collected over 190–260 nm range.

### Single-molecule mass photometry

Mass photometry measurements were conducted on a Refeyn TwoMP instrument (Refeyn Ltd). The system was calibrated with a bovine serum albumin (BSA) standard prior to data acquisition. WTAP fragments were diluted to 1 μM in their respective storage buffers immediately before measurement. For each measurement, 12 μL of buffer was first applied to a clean coverslip to establish a focus lock, followed by the addition of 3 μL of the protein sample to a final volume of 15 μL. Movies were recorded and analyzed using Refeyn AcquireMP software, and mass calibration was performed based on the contrast-to-mass ratio derived from the BSA standard.

### Crystallography

Crystals of the WTAP 1–50 fragment were obtained by sitting-drop vapor diffusion at 16 °C. Purified protein was mixed with reservoir solution containing 0.1 M HEPES (pH 7.5) and 40% (v/v) polyethylene glycol (PEG) 400. Native and selenomethionine-labeled WTAP 150–245 crystals were also obtained by sitting-drop vapor diffusion at 16 °C using reservoir solution containing 45% (w/v) pentaerythritol propoxylate (17/8 PO/OH) and 100 mM Tris (pH 8.5). Crystals were cryoprotected by brief soaking in reservoir solution supplemented with 25% glycerol and flash-frozen in liquid nitrogen.

X-ray diffraction data were collected at beamline BL19U1 of the Shanghai Synchrotron Radiation Facility (SSRF) and processed with HKL2000 (Otwinowski and Minor, 1997). The structure was solved by single-wavelength anomalous dispersion (SAD) using CRANK2 in the CCP4 suite (Skubák and Pannu, 2013). Initial model building was performed in COOT (Emsley et al, 2010), followed by iterative refinement using PHENIX. The final model was validated using MolProbity (Chen et al, 2010), and refinement statistics are summarized in Table EV1. Structural figures were generated with PyMOL.

### Cell lines, cell culture and synchronization

HeLa and HEK293T cell lines were obtained from the American Type Culture Collection (ATCC) and maintained at 37 °C in a humidified incubator with 5% CO_2_. Cells were cultured in Dulbecco’s Modified Eagle Medium (DMEM), supplemented with 10% fetal bovine serum and 1% penicillin–streptomycin. For cell cycle synchronization, HeLa cells were arrested at the G1/S phase by treatment with 2.5 mM thymidine (Sigma) for 16 h. For METTL3 inhibition, cells were treated with 5 μM STM2457 or an equivalent volume of DMSO as a vehicle control during the thymidine block. Cells were then released into fresh culture medium for 8 h prior to enrich for the mitotic population.

### Lentiviral shRNAs

Short hairpin RNAs (shRNAs) targeting human WTAP were designed, synthesized, and subcloned into the PLKO.1-puro lentiviral vector (Addgene). The target sequences were: shRNA-1: 5’-TCGAATGCTTATCCAGGAGAA-3’, and shRNA-2: 5’-GAGAAAGCAGTGAGTGGGAAA-3’. Lentiviral particles were produced by co-transfecting HEK293T cells with the shRNA construct, packaging plasmid psPAX2, and envelope plasmid pMD2.G at a 4:3:1 ratio using Lipofectamine 2000 (Thermo Fisher Scientific). 48 h post-transfection, viral supernatants were harvested, filtered through 0.45 μm syringe filters, and used immediately for infection.

For lentiviral infection, HeLa cells at ~60% confluency were cultured in DMEM supplemented with 10% FBS and incubated with viral supernatant in the presence of 5 μg/mL polybrene (Sigma) for 12 h. The medium was then replaced with fresh DMEM containing 10% FBS. After 24 h, cells were selected with puromycin for 5 consecutive days, after which surviving cells were pooled to establish stable knockdown lines.

### Protein immunoprecipitation

Cells were harvested and lysed on ice in buffer containing 25 mM Tris-HCl (pH 7.5), 150 mM NaCl, 5% glycerol, 1% NP-40, and 2 mM EDTA, supplemented with protease inhibitor cocktail. The lysates were incubated on ice for 30 min, followed by centrifugation at 13,000 rpm for 10 min at 4 °C to remove cell debris. The supernatants were collected and incubated with Anti-Flag M2 agarose beads (Sigma-Aldrich) for 2 h at 4 °C with gentle rotation.

After incubation, the beads were washed and bound proteins were eluted by boiling in SDS loading buffer at 95 °C for 10 min. Samples were resolved by SDS-PAGE (10%–15%) and transferred to polyvinylidene difluoride (PVDF) membranes (Sigma-Aldrich). Membranes were blocked with 5% nonfat milk in PBST (PBS + 0.1% Tween-20) for 1 h at room temperature, then incubated with primary antibodies overnight at 4 °C. After three washes with PBST, membranes were incubated with horseradish peroxidase (HRP)-conjugated secondary antibodies (1:10,000) in 5% milk PBST for 1 h at room temperature. Signals were developed using BeyoECL Plus (Beyotime). The following antibodies were used for Western blotting: anti-Flag (Proteintech, #66008-4-Ig) and anti-GFP (Proteintech, #50430-2-AP).

For immunoblotting of endogenous proteins, cell lysates were boiled at 95 °C and resolved by SDS-PAGE prior to transfer to PVDF membranes. The following antibodies were used: anti-WTAP (Proteintech, #60188-1-Ig), anti-p21 Waf1/Cip1 (Santa Cruz, #sc-6246), anti-KIF20A (Santa Cruz, #sc-374508), anti-IQGAP1 (Huaan Biotechnology, #HA721972), anti-DYRK3 (Thermo Fisher Scientific, #PA5-40341), and anti-GAPDH (Proteintech, #10494-1-AP). All immunoblot experiments were independently repeated at least three times.

### Fluorescence microscopy

Cells were grown on coverslips, washed once with PBS, and fixed with 4% paraformaldehyde in PBS for 10 min at room temperature. After fixation, cells were permeabilized with 0.2% Triton X-100 in PBS for 10 min, followed by blocking in PBST (PBS + 0.1% Tween-20) containing 1% bovine serum albumin (BSA) for 30 min at room temperature. Cells were then incubated overnight at 4 °C with primary antibodies diluted in PBST. The next day, cells were washed three times with PBST and incubated with fluorophore-conjugated secondary antibodies for 1 h at room temperature. Nuclei were counterstained with 0.5 μg/mL DAPI for 10 min prior to mounting. Images were acquired using a Zeiss LSM 980 laser-scanning confocal microscope. Primary antibodies used were: anti-Flag (1:1000; Proteintech, #66008-4-Ig), anti-GFP (1:1000; Proteintech, #50430-2-AP), and anti-α-tubulin (1:500; Sigma-Aldrich, #T6199). Secondary antibodies conjugated to Alexa Fluor 488, 568, or 647 (Thermo Fisher) were used at 1:1000 dilution.

For live-cell time-lapse imaging, HeLa cells were cultured in glass-bottom dishes (MatTek) and maintained in CO_2_-independent medium (Gibco) supplemented with 10% FBS and 1% penicillin–streptomycin. Imaging was performed at 37 °C in a sealed environmental chamber using a DeltaVision fluorescence imaging system. Time-lapse images were analyzed with ImageJ.

### Crystal violet staining

Cells were fixed with 4% methanol-free formaldehyde (Polysciences) for 10 min at room temperature. Following fixation, cells were stained with 0.1% (w/v) crystal violet solution (Biosharp) for 10 min at room temperature. Excess dye was removed by rinsing the plates five times with PBS. Plates were then air-dried and scanned for image documentation and further analysis.

### Cell proliferation assay

HeLa cells were seeded in 12-well plates at equal density in 500 μL complete growth medium per well on day 0. At the indicated time points, cells were trypsinized, resuspended in culture medium, and counted using an automated cell counter (Countstar) with disposable counting chamber slides. Each time point was measured in triplicate from independent wells, and the experiment was independently repeated at least three times.

### RNA extraction and m^6^A-seq library construction

Total RNA was extracted using TRIzol reagent (Invitrogen, Carlsbad, CA, USA) according to the manufacturer’s instructions. RNA concentration and purity were assessed using a NanoDrop ND-1000 spectrophotometer (Thermo Fisher Scientific, USA). Polyadenylated mRNA was subsequently purified from total RNA using the GenElute mRNA Miniprep Kit (Sigma, cat. no. MRN10-1KT) through two rounds of enrichment.

For m^6^A-seq, 5% of the fragmented RNA lysate was reserved as input. The remaining mRNA was fragmented at 86 °C for 7 min using the Magnesium RNA Fragmentation Module (NEB, cat. no. E6150), then incubated for 2 h at 4 °C with 5 μg of m^6^A-specific antibody (Synaptic Systems, cat. no. 202003) in immunoprecipitation buffer (50 mM Tris-HCl, 750 mM NaCl, 0.5% Igepal CA-630). Immunoprecipitated RNA and input controls were extracted, purified, and subjected to reverse transcription to generate cDNA. Second-strand synthesis with U-labeling was performed, followed by A-tailing and ligation of single- or dual-indexed sequencing adapters. AMPure XP beads (Beckman Coulter) were used to select the appropriate fragment sizes.

The U-labeled second-stranded DNA was treated with a heat-labile UDG enzyme (NEB, USA) to remove uracil residues prior to PCR amplification. The resulting cDNA libraries were sequenced on an Illumina NovaSeq^TM^ 6000 platform using paired-end 150 bp (PE150) reads. Raw reads containing sequencing adapters or low-quality bases were filtered using fastp (version 0.19.4, https://github.com/OpenGene/fastp) to generate clean, high-quality data for downstream analysis. Libraries were sequenced on the Illumina sequencing platform by LC-Bio Technology Co., Ltd (Hangzhou, China).

### Quantitative analysis of the m^6^A levels using LC-MS/MS

To quantify global m⁶A levels, 300–500 ng of total RNA from each sample was enzymatically digested in a 30 μL reaction containing 1 unit of nuclease P1 (Wako) and 20 mM ammonium acetate (NH₄OAc, pH 5.3) at 42 °C for 2 h. Following digestion, 1 μL of shrimp alkaline phosphatase (NEB) and 3.5 μL of 10× CutSmart buffer (NEB) were added to the reaction, which was then incubated at 37 °C for an additional 2 h to complete dephosphorylation. The resulting nucleoside solution was centrifuged at 13,000 rpm for 10 min at 4 °C to remove debris, diluted to 60–70 μL with DEPC-treated water, and filtered through a 0.22 μm membrane (Millipore). A 10 μL aliquot was injected into a liquid chromatography–tandem mass spectrometry (LC-MS/MS) system (AB Sciex QTRAP 6500 + ) for analysis.

m^6^A and adenosine (A) were quantified using multiple reaction monitoring (MRM) with the following mass transitions: m^6^A, m/z 282.1 → 150.1; A, m/z 268.2 → 136.1. Absolute concentrations were calculated by comparison with calibration curves generated from synthetic nucleoside standards. The m^6^A-to-A ratio was determined by dividing the molar concentration of m^6^A by that of A, and used as a readout of relative methylation levels.

## Supplementary information

Table EV1

Table EV2

Table EV3

Peer Review File

Dataset EV1

Source data Fig. 1

Source data Fig. 2

Source data Fig. 3

Source data Fig. 4

Source data Fig. 5

Source data Fig. 6

Figure EV1 Source Data

Figure EV2 Source Data

Figure EV3 Source Data

Figure EV4 Source Data

Figure EV5 Source Data

Expanded View Figures

## Data Availability

The atomic coordinates of the WTAP-N and WTAP M-CC have been deposited to the Protein Data Bank (https://www.rcsb.org/) under the accession code 9XOE and 9XOF, respectively. The raw sequencing data generated in this study have been deposited in the Gene Expression Omnibus (GEO) database under the accession code GSE324500. The source data of this paper are collected in the following database record: biostudies:S-SCDT-10_1038-S44319-026-00815-3.
