# Supplementary material for: WTAP tetramer ensures m6A writer assembly and faithful mitosis
Source: EMBO Rep. 2026 Jun 2;27(13):3842–62. doi: 10.1038/s44319-026-00815-3 (PMC13354555; doi:10.1038/s44319-026-00815-3)
Supplement: Supplementary file 1 — Table EV1 [file 44319_2026_815_MOESM1_ESM.docx]

**Table EV1 Statistics of X-ray Crystallographic Data Collection and Model refinement**

| \| **Data collection** \| \| \| \| --- \| --- \| --- \| \| Data sets \| WTAP-N \| WTAP M-CC \| \| Space group \| *P4_1_2_1_2* \| *P2* \| \| Wavelength (Å) \| 0.97915 \| 0.97918 \| \| Unit Cell Parameters (Å) \| a=b=76.173, c=75.414  α=β=γ=90° \| a=47.621, b=104.618, c=101.601  α=γ=90°, β=90.220° \| \| Resolution range (Å) \| 50-1.65 (1.68-1.65) \| 50-2.80 (2.85-2.80) \| \| No. of unique reflections \| 27412 (1329) \| 24493 (1163) \| \| Redundancy \| 24.9 (25.1) \| 13.6 (12.3) \| \| I/σ \| 65.6 (2.7) \| 24.1 (2.5) \| \| Completeness (%) \| 100 (100) \| 98.4 (94.6) \| \| R_merge_ ^a^(%) \| 6.8 (101.7) \| 14.5 (74.8) \| \| CC_1/2_ ^b^ \| 0.996 (0.890) \| 0.971 (0.888) \| \| **Structure refinement** \|  \|  \| \| Resolution (Å) \| 50-1.65 (1.71-1.65) \| 50-2.80 (2.90-2.80) \| \| R_cryst_ ^c^/R_free_ ^d^ (%) \| 23.01/25.59 (29.05/33.77) \| 21.86/27.04 (27.77/37.31) \| \| rmsd bonds (Å)/angles (°) \| 0.009/1.091 \| 0.004/0.739 \| \| Average B factor (Å^2^) ^e^ \| 36.1 \| 52.7 \| \| No. of atoms \|  \|  \| \| Protein atoms \| 1538 \| 5858 \| \| Water \| 56 \| 5 \| \| Ligands \| 0 \| 0 \| \| Working set \| 26100 (2819) \| 23152 (2402) \| \| Test set \| 1256 (120) \| 1297 (135) \| \| Ramachandran plot regions ^e^ \|  \|  \| \| Favored (%) \| 98.86 \| 99.31 \| \| Allowed (%) \| 1.14 \| 0.69 \| \| Outliers (%) \| 0 \| 0 \| |
| --- | --- | --- | --- | --- | --- | --- | --- | --- | --- | --- | --- | --- | --- | --- | --- | --- | --- | --- | --- | --- | --- | --- | --- | --- | --- | --- | --- | --- | --- | --- | --- | --- | --- | --- | --- | --- | --- | --- | --- | --- | --- | --- | --- | --- | --- | --- | --- | --- | --- | --- | --- | --- | --- | --- | --- | --- | --- | --- | --- | --- | --- | --- | --- | --- | --- | --- | --- | --- | --- | --- | --- | --- | --- | --- | --- | --- | --- | --- | --- | --- | --- |
|  |

Numbers in parentheses represent the value for the highest resolution shell.

a. R_merge_ = Σ |*I_i_* - <*I*>| / Σ*I_i_*, where *I_i_* is the intensity of measured reflection and <*I*> is the mean intensity of all symmetry-related reflections.

b. CC_1/2_ were defined by Karplus and Diederichs(Karplus & Diederichs, 2012).

c. R_cryst_=Σ||*F*_calc_| – |*F*_obs_||/Σ*F*_obs_, where *F*_obs_ and *F*_calc_ are observed and calculated structure factors.

d. R_free_= Σ_T_||*F*_calc_| – |*F*_obs_||/Σ*F*_obs_, where T is a test data set of about 5% of the total unique reflections randomly chosen and set aside prior to refinement.

e. B factors and Ramachandran plot statistics are calculated using MOLPROBITY(Chen *et al*, 2010).
