# Supplementary figures and images for: WTAP tetramer ensures m6A writer assembly and faithful mitosis (part 1 of 4)
Source: EMBO Rep. 2026 Jun 2;27(13):3842–62. doi: 10.1038/s44319-026-00815-3 (PMC13354555; doi:10.1038/s44319-026-00815-3)

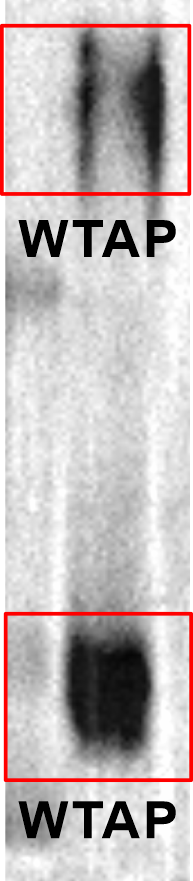

Supplement: Supplementary file 6 — Source data Fig. 1 [file 44319_2026_815_MOESM6_ESM.zip › Figure 1/1E/WTAP native-PAGE.tif]

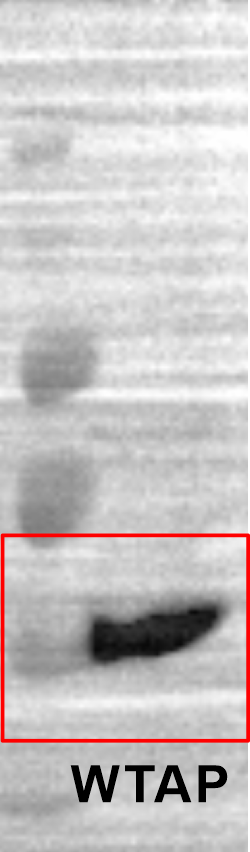

Supplement: Supplementary file 6 — Source data Fig. 1 [file 44319_2026_815_MOESM6_ESM.zip › Figure 1/1F/WTAP SDS-PAGE.tif]

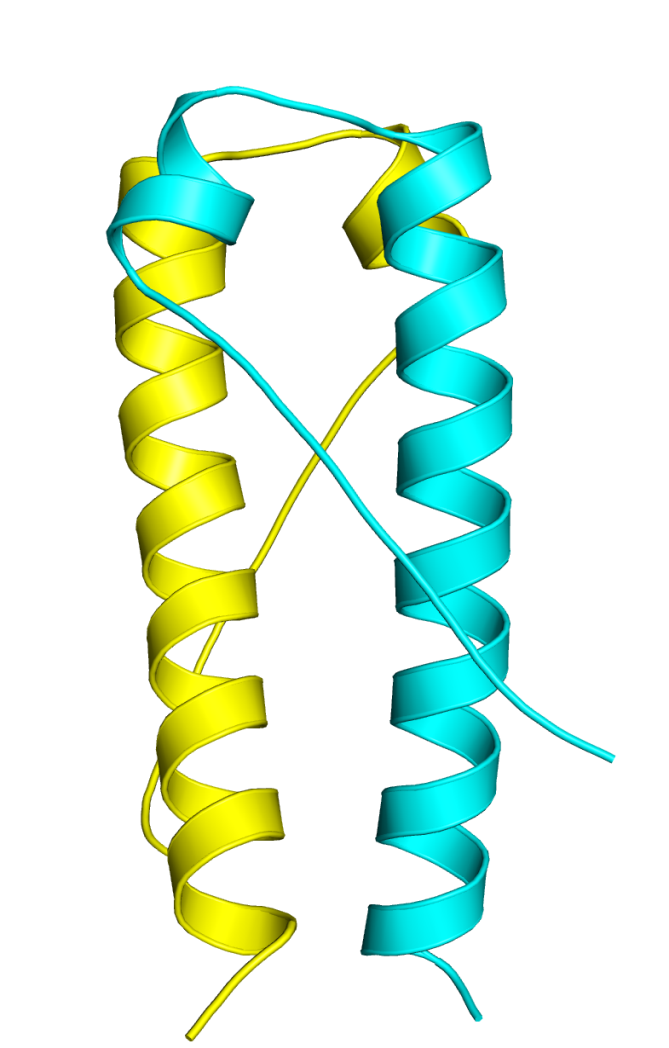

Supplement: Supplementary file 7 — Source data Fig. 2 [file 44319_2026_815_MOESM7_ESM.zip › Figure 2/2A/WTAP-N-2.tif]

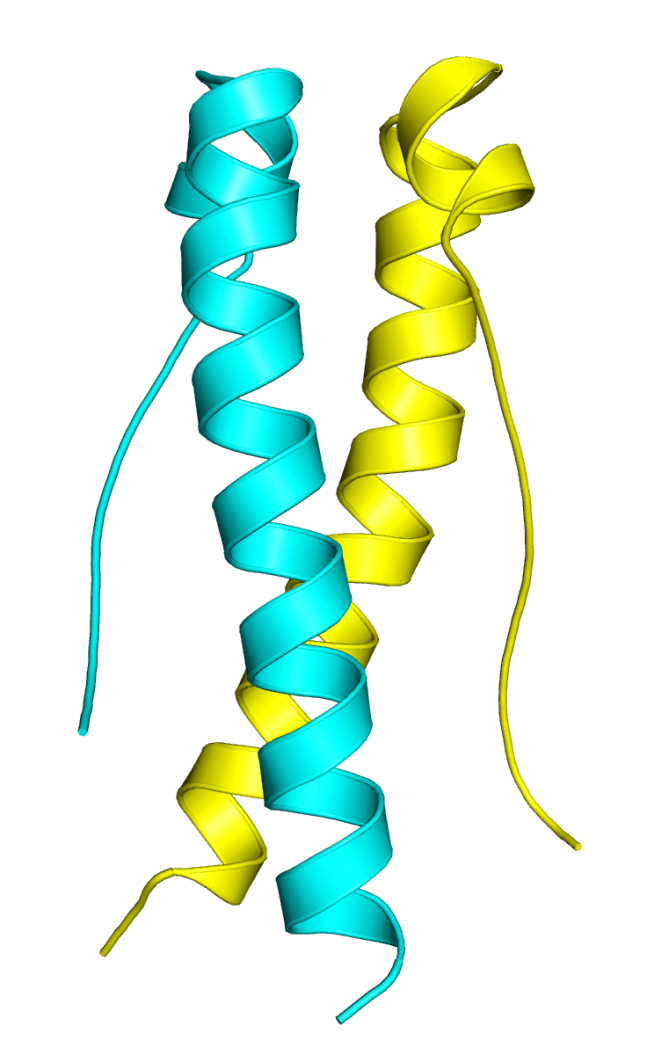

Supplement: Supplementary file 7 — Source data Fig. 2 [file 44319_2026_815_MOESM7_ESM.zip › Figure 2/2A/WTAP-N.tif]

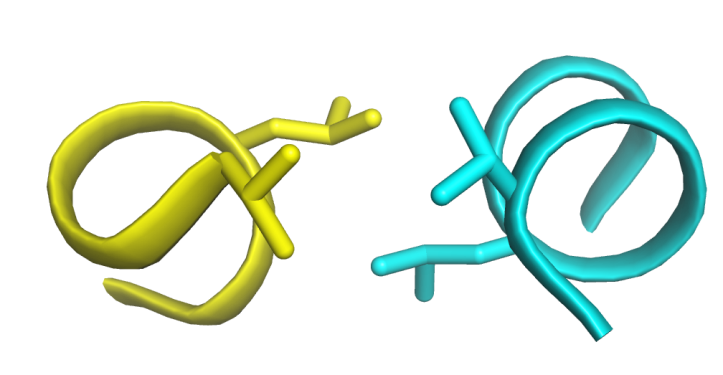

Supplement: Supplementary file 7 — Source data Fig. 2 [file 44319_2026_815_MOESM7_ESM.zip › Figure 2/2B/WTAP-N interaction-2.tif]

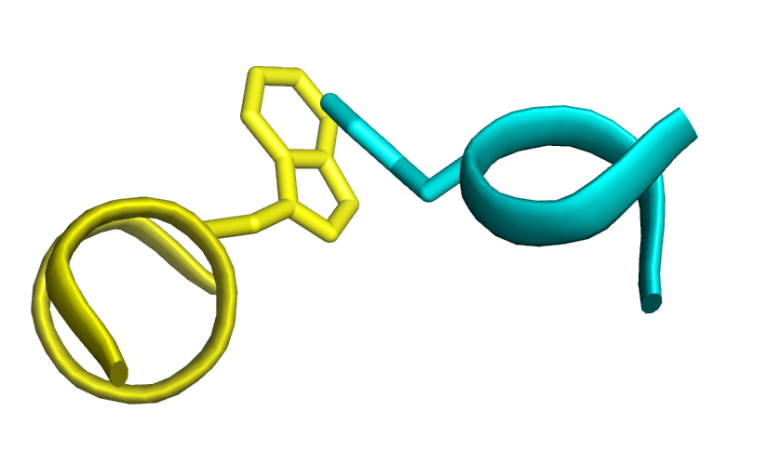

Supplement: Supplementary file 7 — Source data Fig. 2 [file 44319_2026_815_MOESM7_ESM.zip › Figure 2/2B/WTAP-N interaction.tif]

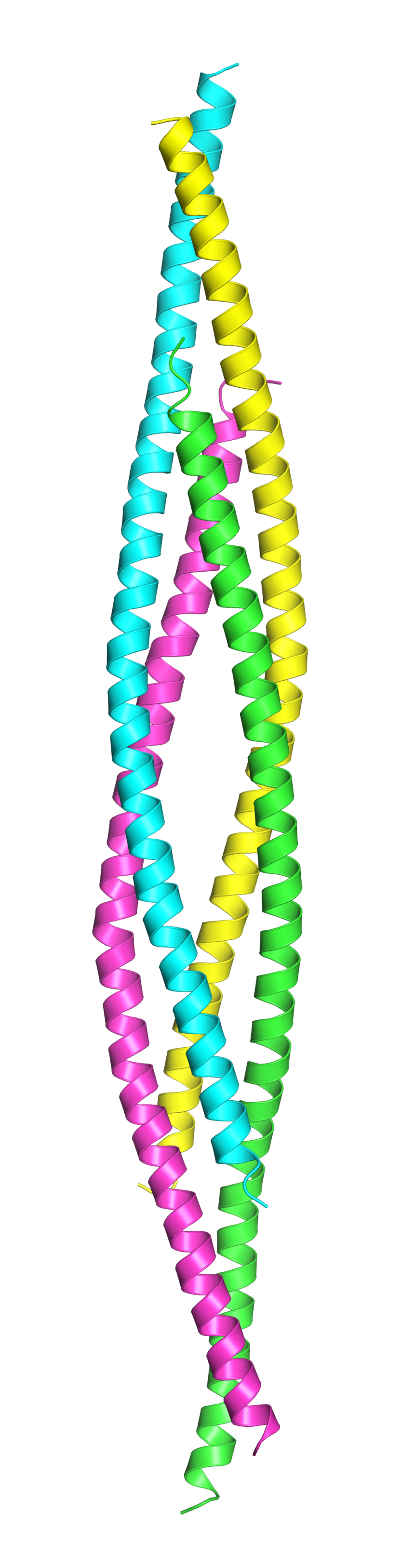

Supplement: Supplementary file 7 — Source data Fig. 2 [file 44319_2026_815_MOESM7_ESM.zip › Figure 2/2C/WTAP M-CC-2.tif]

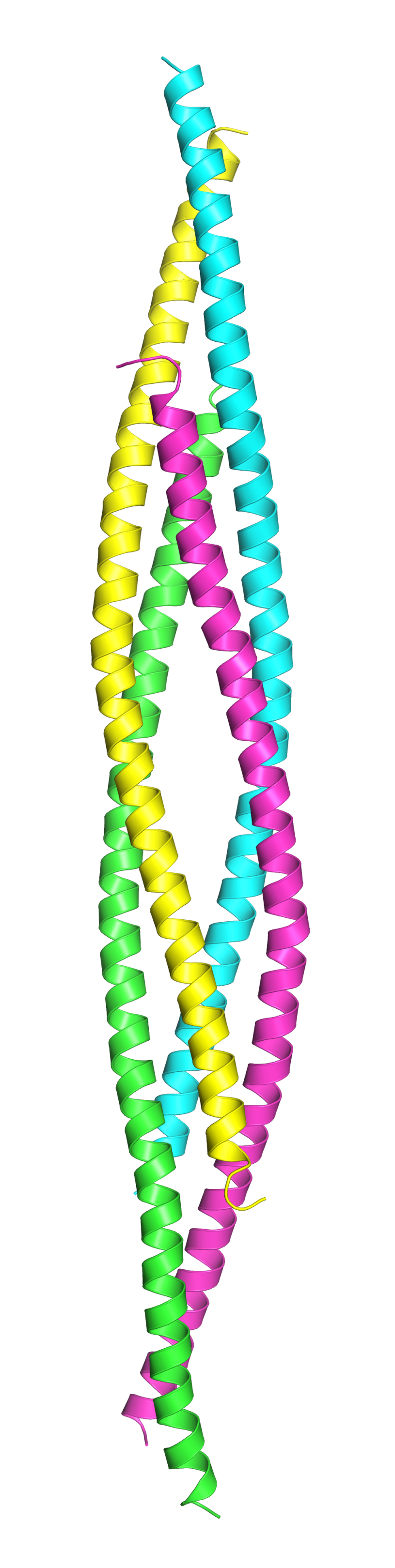

Supplement: Supplementary file 7 — Source data Fig. 2 [file 44319_2026_815_MOESM7_ESM.zip › Figure 2/2C/WTAP M-CC.tif]

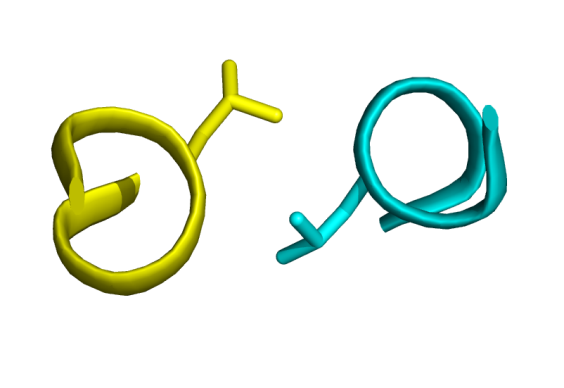

Supplement: Supplementary file 7 — Source data Fig. 2 [file 44319_2026_815_MOESM7_ESM.zip › Figure 2/2D/layer1.tif]

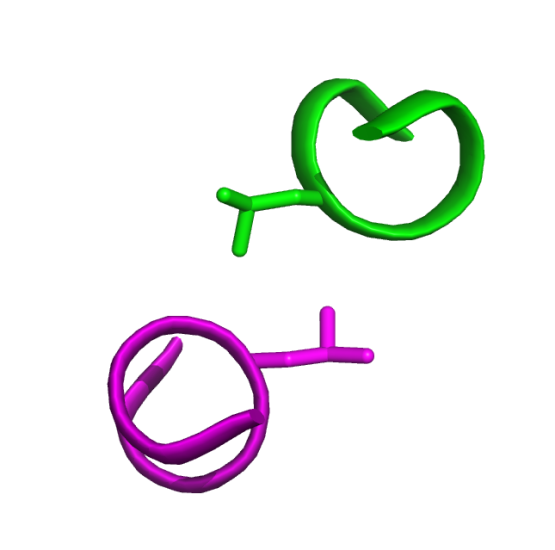

Supplement: Supplementary file 7 — Source data Fig. 2 [file 44319_2026_815_MOESM7_ESM.zip › Figure 2/2D/layer10.tif]

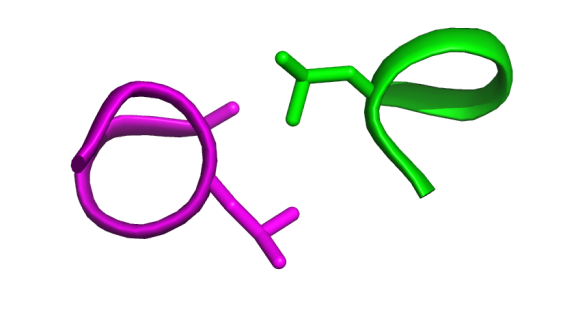

Supplement: Supplementary file 7 — Source data Fig. 2 [file 44319_2026_815_MOESM7_ESM.zip › Figure 2/2D/layer11.tif]

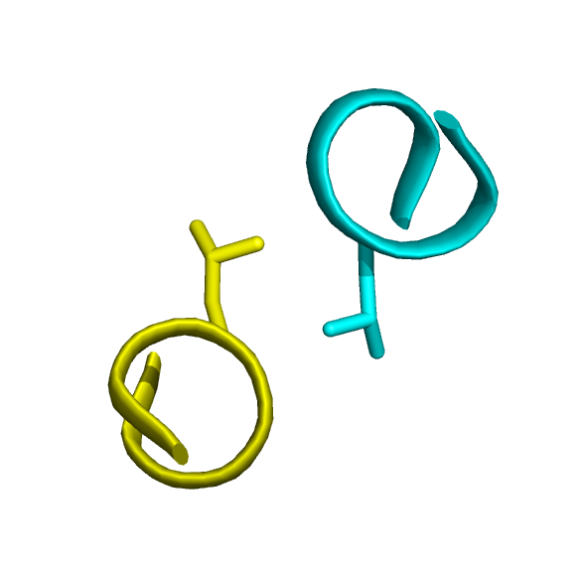

Supplement: Supplementary file 7 — Source data Fig. 2 [file 44319_2026_815_MOESM7_ESM.zip › Figure 2/2D/layer2.tif]

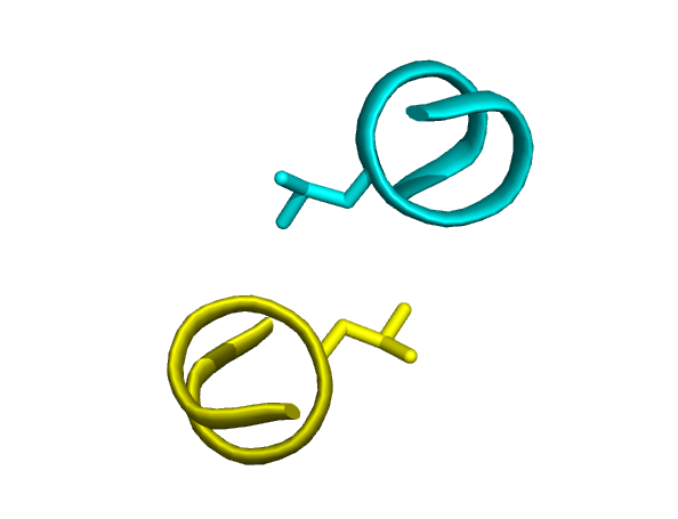

Supplement: Supplementary file 7 — Source data Fig. 2 [file 44319_2026_815_MOESM7_ESM.zip › Figure 2/2D/layer3.tif]

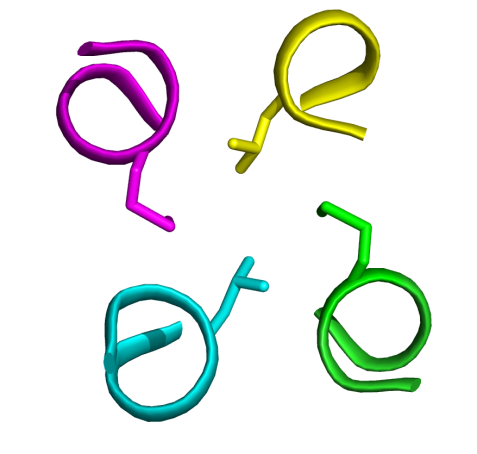

Supplement: Supplementary file 7 — Source data Fig. 2 [file 44319_2026_815_MOESM7_ESM.zip › Figure 2/2D/layer4.tif]

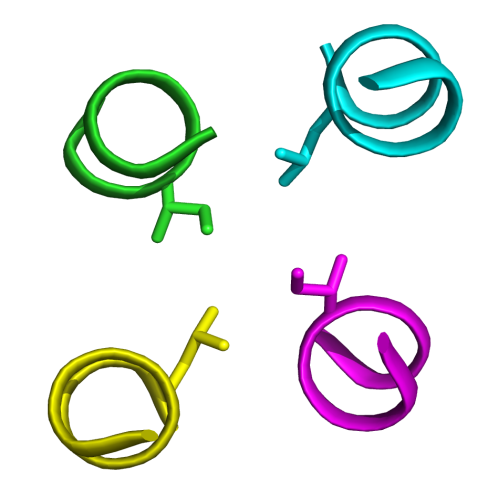

Supplement: Supplementary file 7 — Source data Fig. 2 [file 44319_2026_815_MOESM7_ESM.zip › Figure 2/2D/layer5.tif]

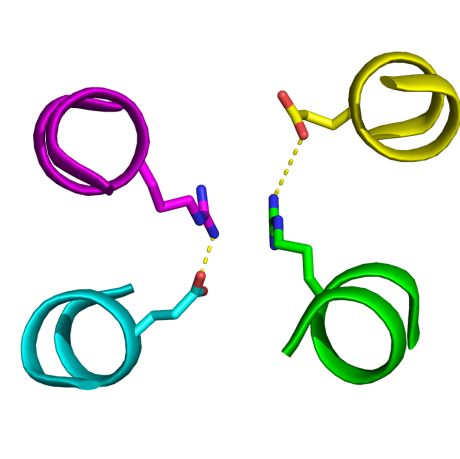

Supplement: Supplementary file 7 — Source data Fig. 2 [file 44319_2026_815_MOESM7_ESM.zip › Figure 2/2D/layer6.tif]

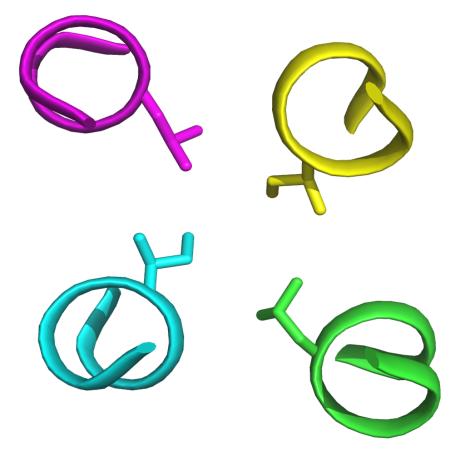

Supplement: Supplementary file 7 — Source data Fig. 2 [file 44319_2026_815_MOESM7_ESM.zip › Figure 2/2D/layer7.tif]

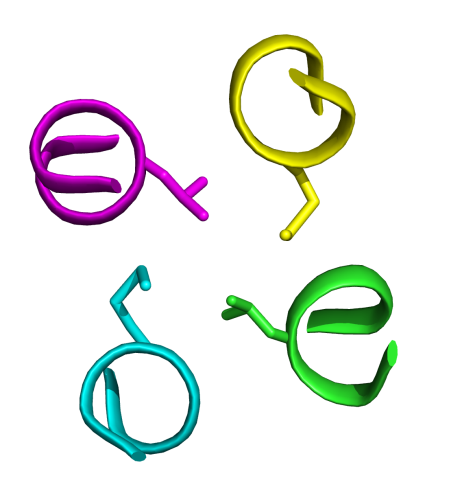

Supplement: Supplementary file 7 — Source data Fig. 2 [file 44319_2026_815_MOESM7_ESM.zip › Figure 2/2D/layer8.tif]

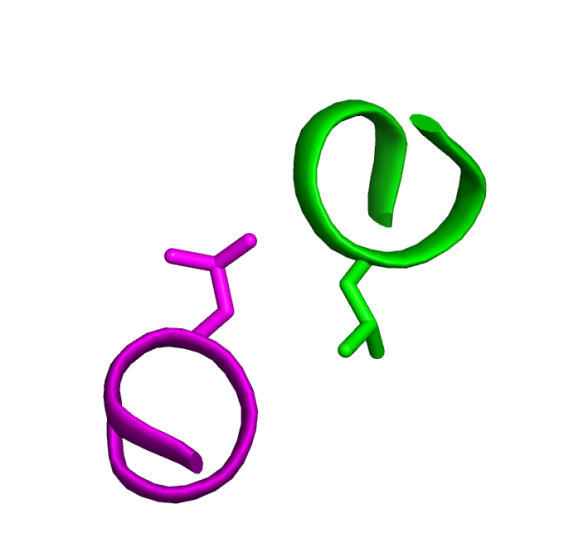

Supplement: Supplementary file 7 — Source data Fig. 2 [file 44319_2026_815_MOESM7_ESM.zip › Figure 2/2D/layer9.tif]

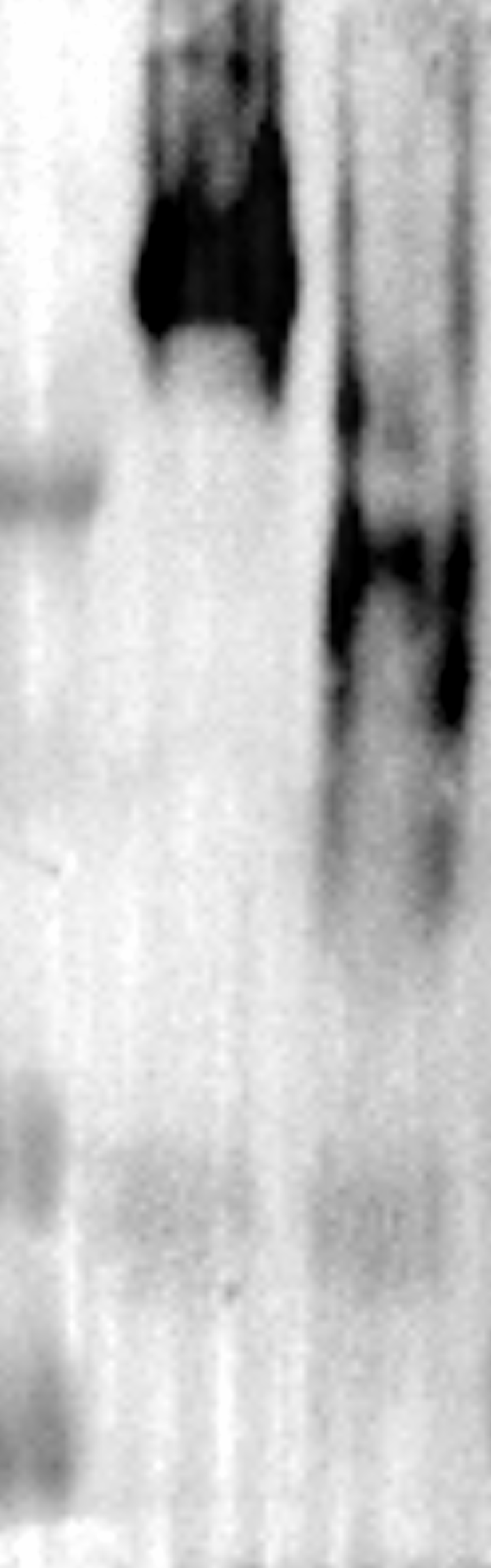

Supplement: Supplementary file 7 — Source data Fig. 2 [file 44319_2026_815_MOESM7_ESM.zip › Figure 2/2F/western blot-anti-WTAP.tif]

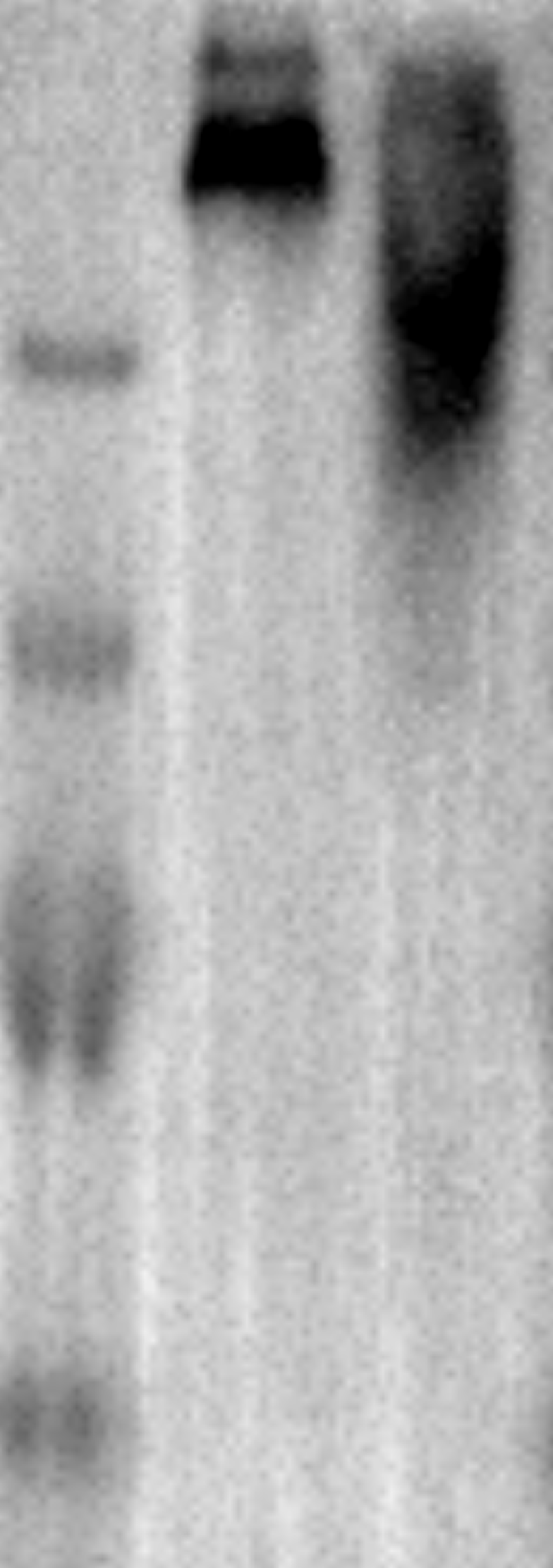

Supplement: Supplementary file 7 — Source data Fig. 2 [file 44319_2026_815_MOESM7_ESM.zip › Figure 2/2G/western blot-anti-flag.tif]

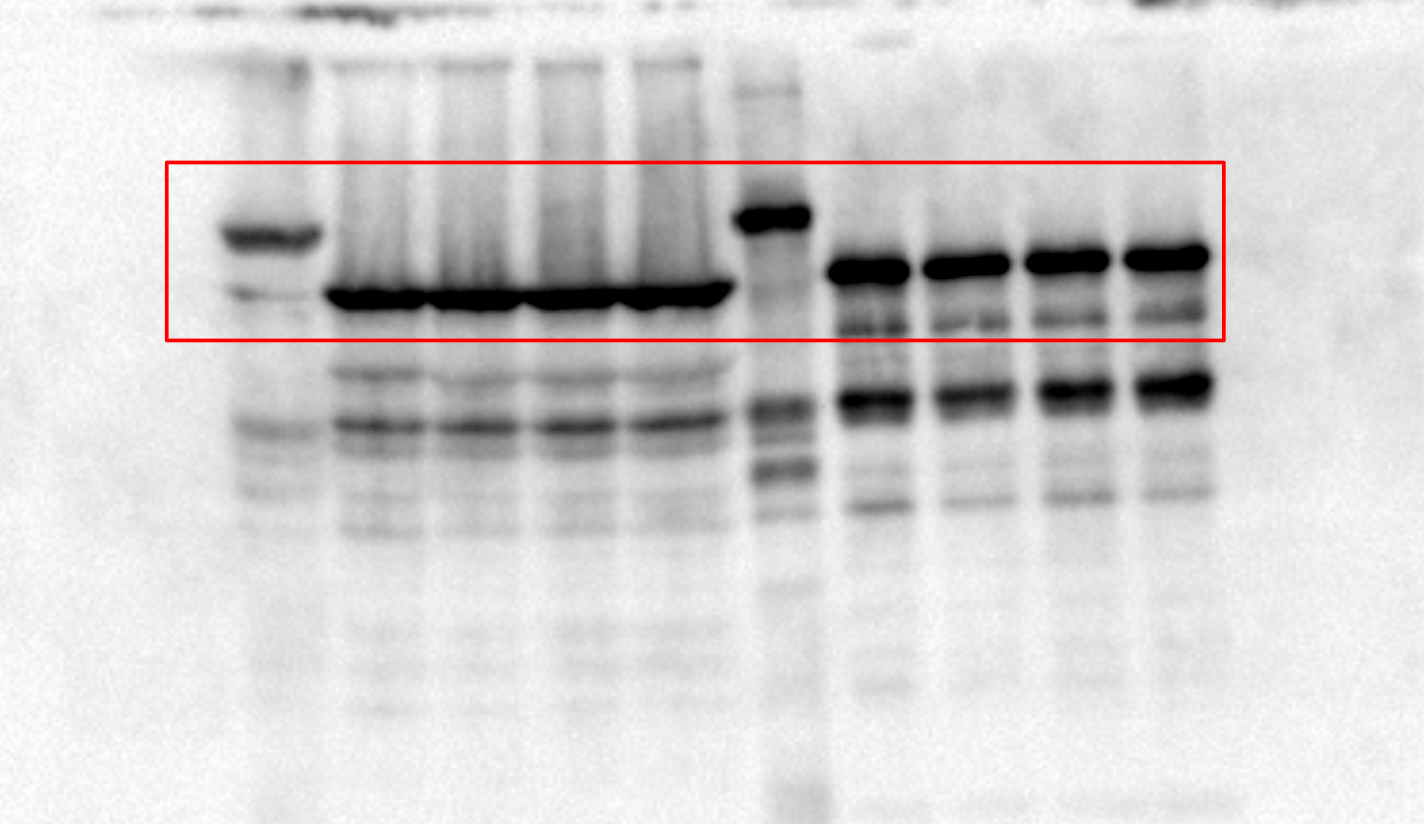

Supplement: Supplementary file 8 — Source data Fig. 3 [file 44319_2026_815_MOESM8_ESM.zip › Figure 3/3A/anti Flag.tif]

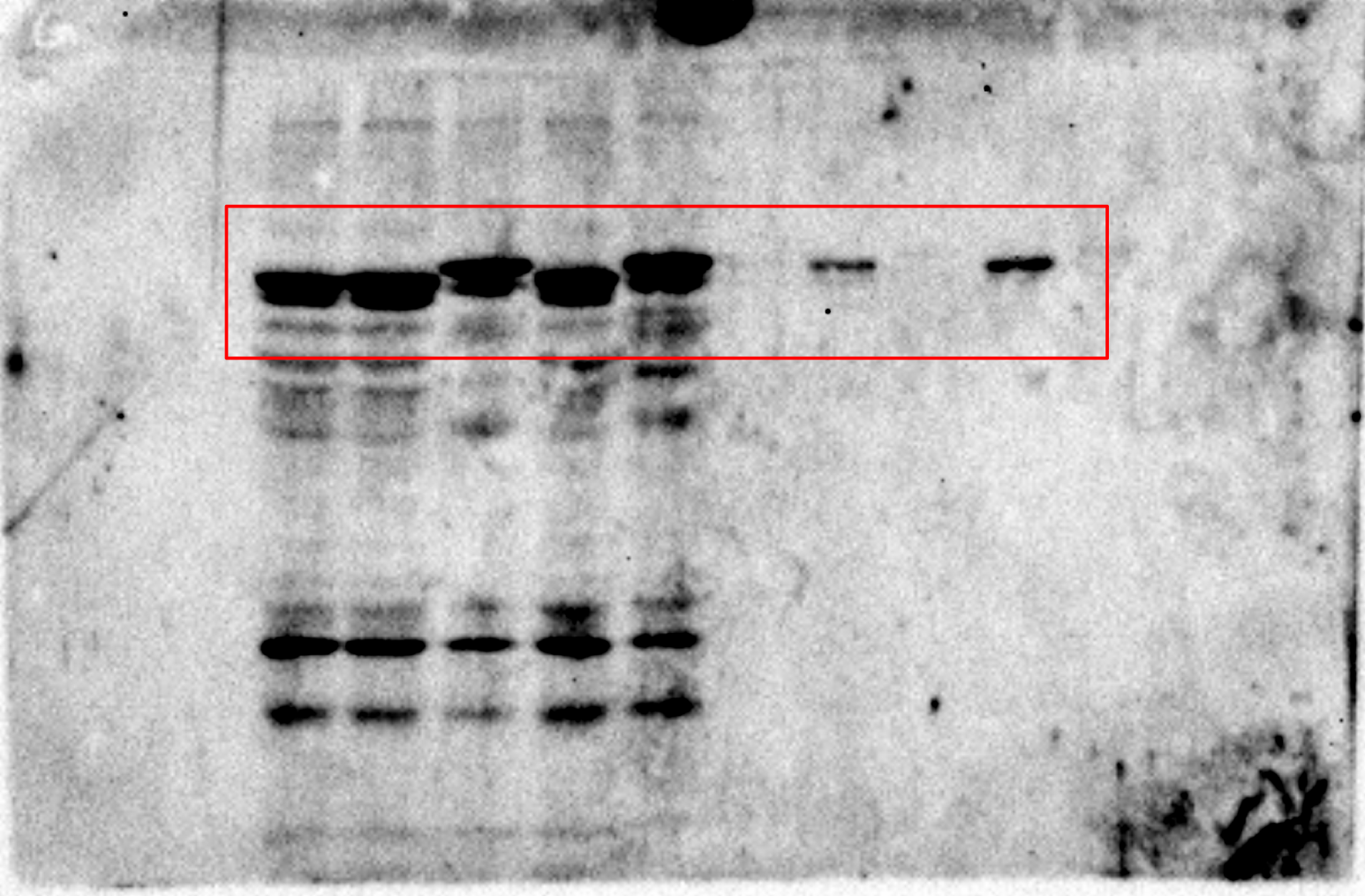

Supplement: Supplementary file 8 — Source data Fig. 3 [file 44319_2026_815_MOESM8_ESM.zip › Figure 3/3A/anti GFP.tif]

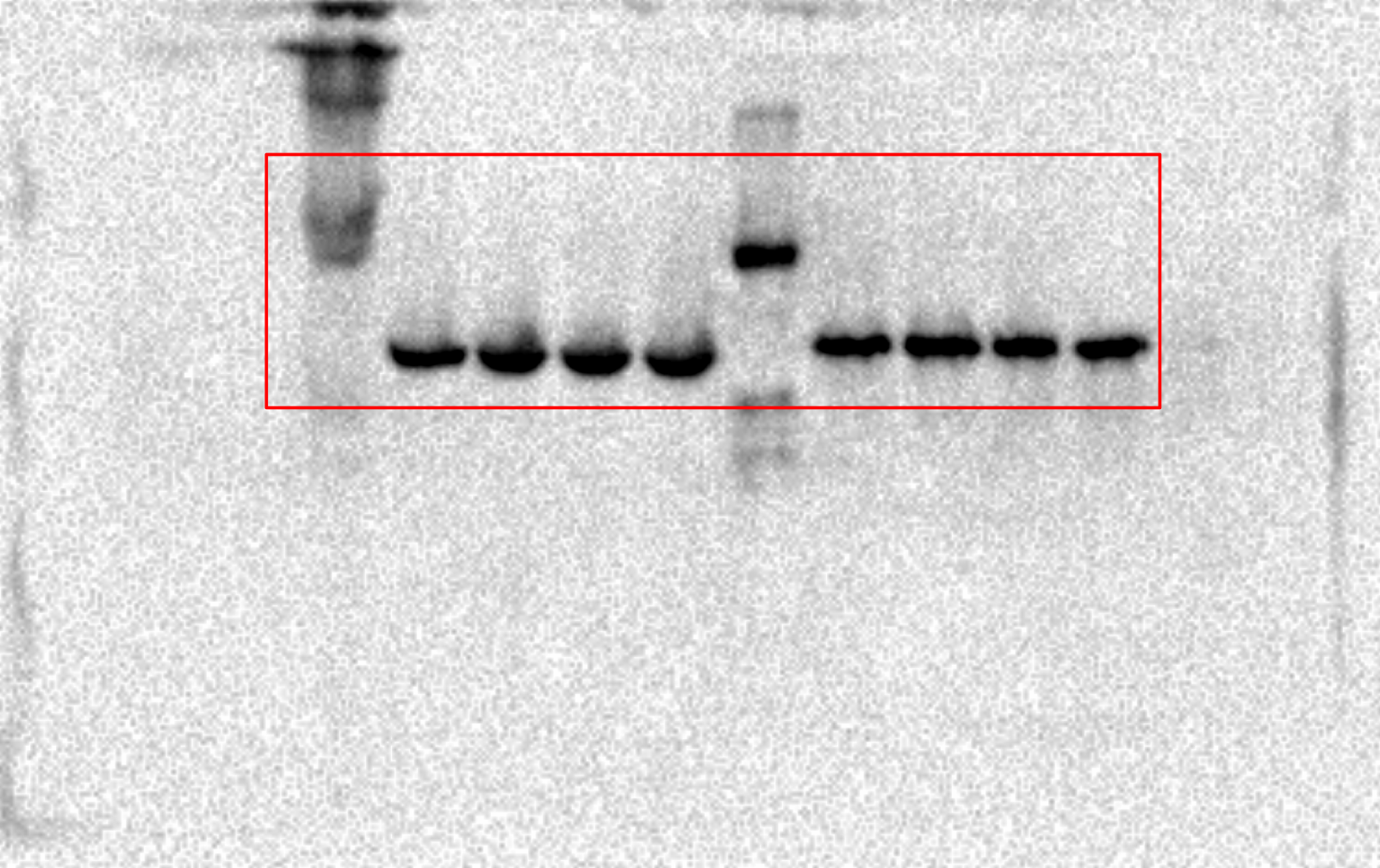

Supplement: Supplementary file 8 — Source data Fig. 3 [file 44319_2026_815_MOESM8_ESM.zip › Figure 3/3B/anti Flag.tif]

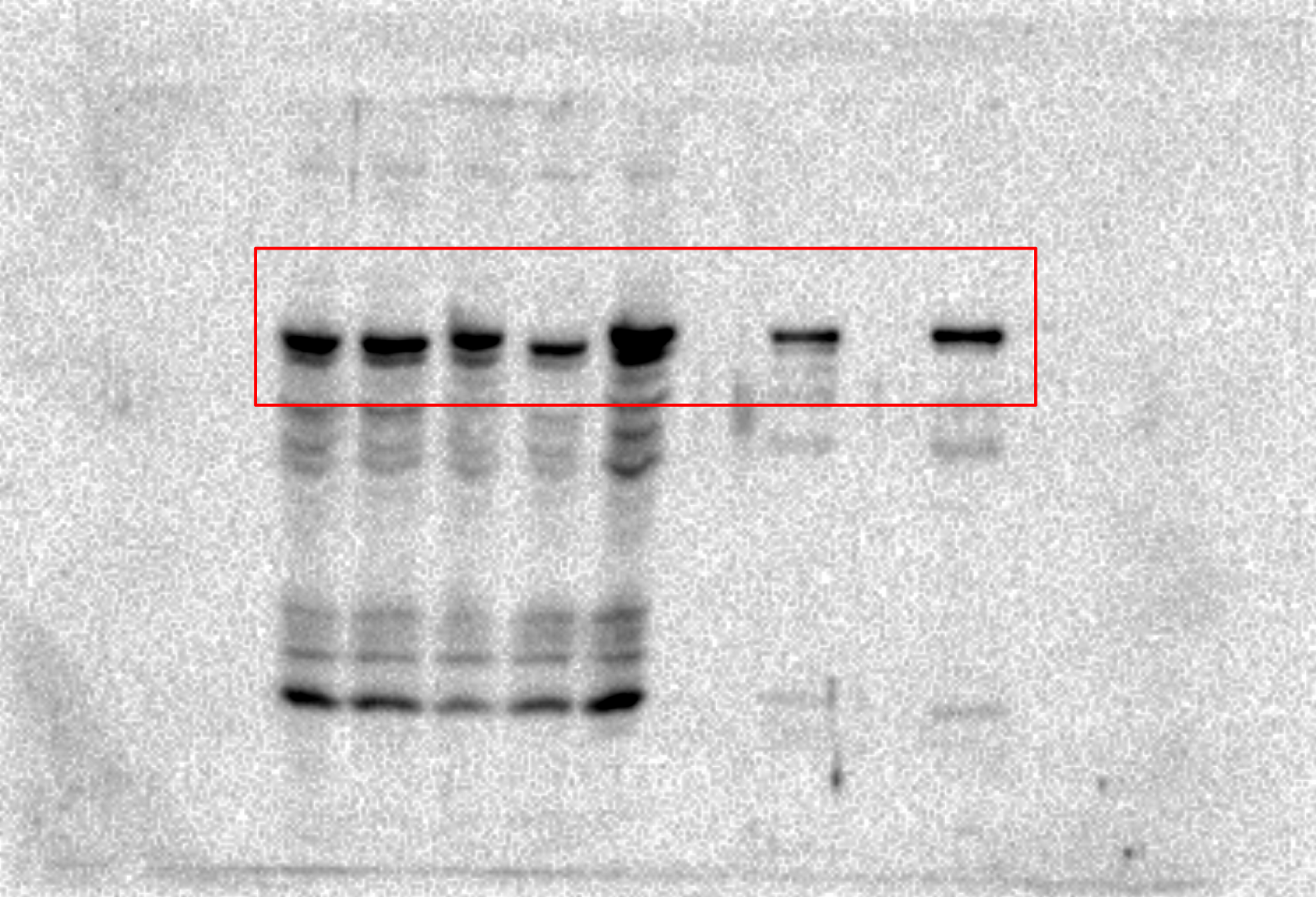

Supplement: Supplementary file 8 — Source data Fig. 3 [file 44319_2026_815_MOESM8_ESM.zip › Figure 3/3B/anti GFP.tif]

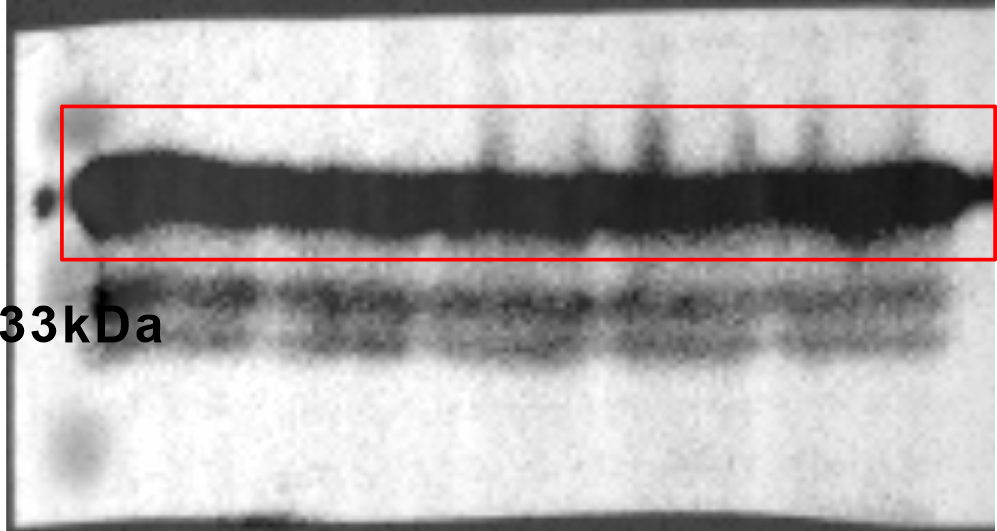

Supplement: Supplementary file 8 — Source data Fig. 3 [file 44319_2026_815_MOESM8_ESM.zip › Figure 3/3C/anti GAPDH.tif]

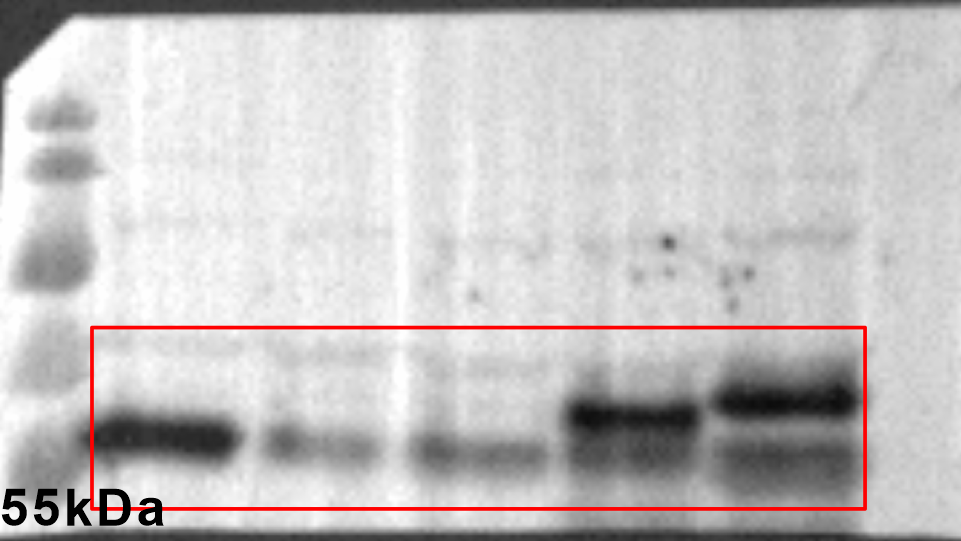

Supplement: Supplementary file 8 — Source data Fig. 3 [file 44319_2026_815_MOESM8_ESM.zip › Figure 3/3C/anti WTAP.tif]

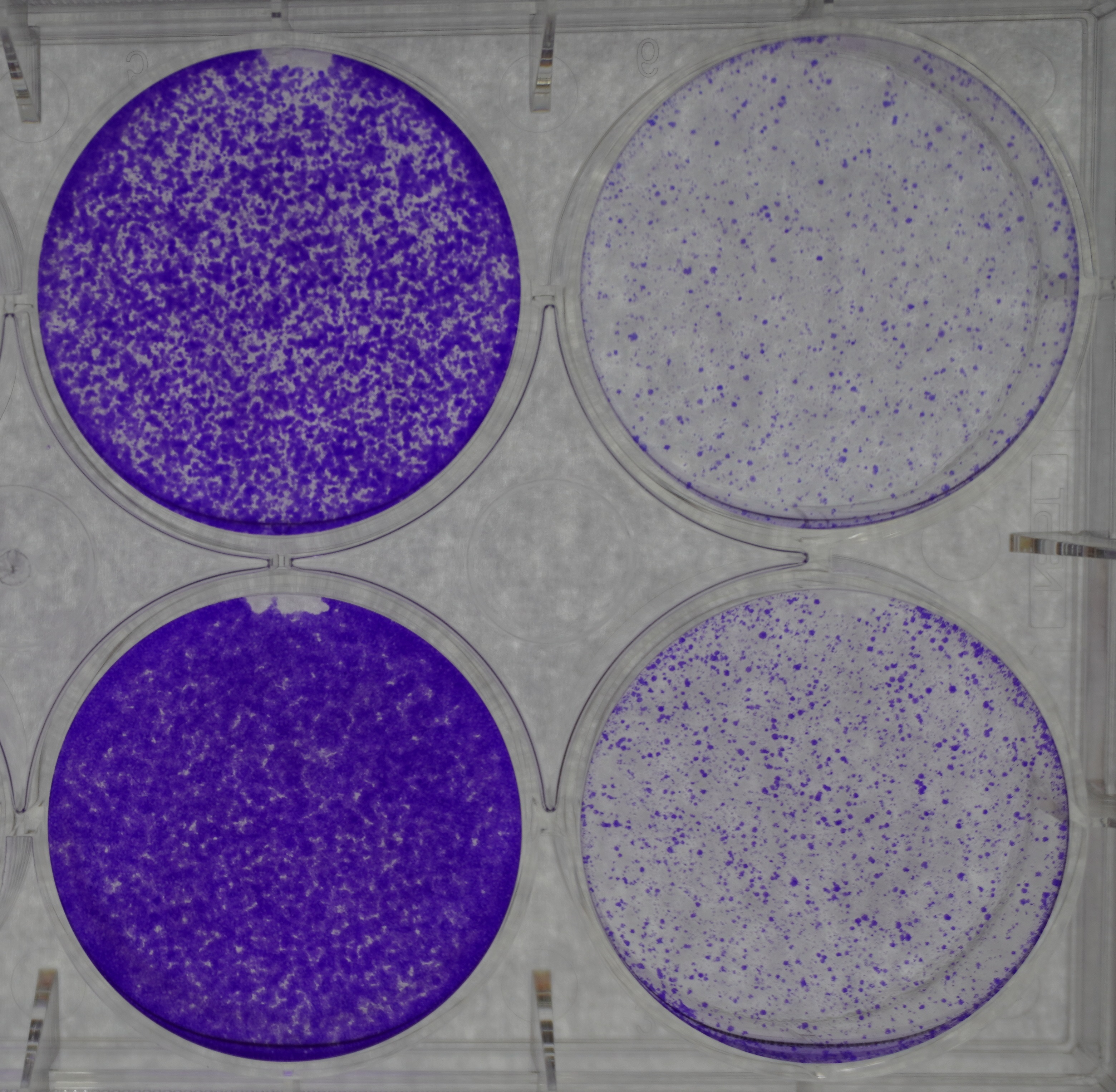

Supplement: Supplementary file 9 — Source data Fig. 4 [file 44319_2026_815_MOESM9_ESM.zip › Figure 4/4A/Crystal violet staining.jpg]

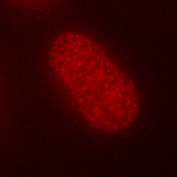

Supplement: Supplementary file 9 — Source data Fig. 4 [file 44319_2026_815_MOESM9_ESM.zip › Figure 4/4C/shNTC-BFP/0-1.tif]

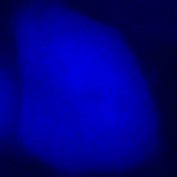

Supplement: Supplementary file 9 — Source data Fig. 4 [file 44319_2026_815_MOESM9_ESM.zip › Figure 4/4C/shNTC-BFP/0-2.tif]

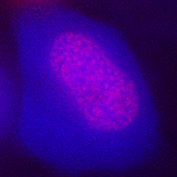

Supplement: Supplementary file 9 — Source data Fig. 4 [file 44319_2026_815_MOESM9_ESM.zip › Figure 4/4C/shNTC-BFP/0-3.tif]

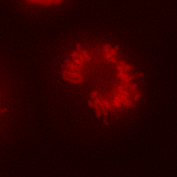

Supplement: Supplementary file 9 — Source data Fig. 4 [file 44319_2026_815_MOESM9_ESM.zip › Figure 4/4C/shNTC-BFP/10-1.tif]

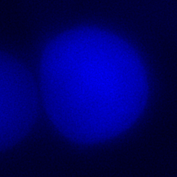

Supplement: Supplementary file 9 — Source data Fig. 4 [file 44319_2026_815_MOESM9_ESM.zip › Figure 4/4C/shNTC-BFP/10-2.tif]

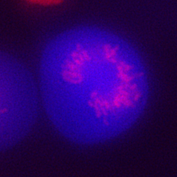

Supplement: Supplementary file 9 — Source data Fig. 4 [file 44319_2026_815_MOESM9_ESM.zip › Figure 4/4C/shNTC-BFP/10-3.tif]

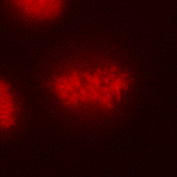

Supplement: Supplementary file 9 — Source data Fig. 4 [file 44319_2026_815_MOESM9_ESM.zip › Figure 4/4C/shNTC-BFP/15-1.tif]

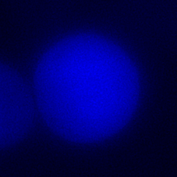

Supplement: Supplementary file 9 — Source data Fig. 4 [file 44319_2026_815_MOESM9_ESM.zip › Figure 4/4C/shNTC-BFP/15-2.tif]

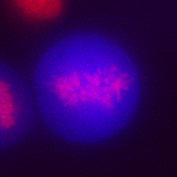

Supplement: Supplementary file 9 — Source data Fig. 4 [file 44319_2026_815_MOESM9_ESM.zip › Figure 4/4C/shNTC-BFP/15-3.tif]

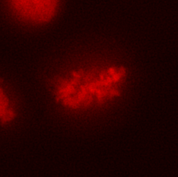

Supplement: Supplementary file 9 — Source data Fig. 4 [file 44319_2026_815_MOESM9_ESM.zip › Figure 4/4C/shNTC-BFP/20-1.tif]

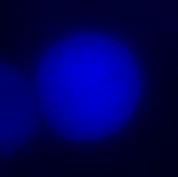

Supplement: Supplementary file 9 — Source data Fig. 4 [file 44319_2026_815_MOESM9_ESM.zip › Figure 4/4C/shNTC-BFP/20-2.tif]

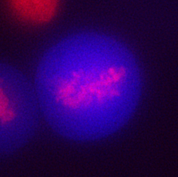

Supplement: Supplementary file 9 — Source data Fig. 4 [file 44319_2026_815_MOESM9_ESM.zip › Figure 4/4C/shNTC-BFP/20-3.tif]

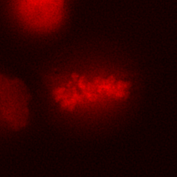

Supplement: Supplementary file 9 — Source data Fig. 4 [file 44319_2026_815_MOESM9_ESM.zip › Figure 4/4C/shNTC-BFP/30-1.tif]

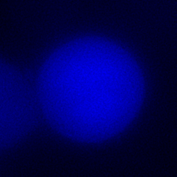

Supplement: Supplementary file 9 — Source data Fig. 4 [file 44319_2026_815_MOESM9_ESM.zip › Figure 4/4C/shNTC-BFP/30-2.tif]

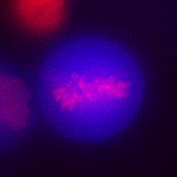

Supplement: Supplementary file 9 — Source data Fig. 4 [file 44319_2026_815_MOESM9_ESM.zip › Figure 4/4C/shNTC-BFP/30-3.tif]

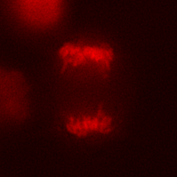

Supplement: Supplementary file 9 — Source data Fig. 4 [file 44319_2026_815_MOESM9_ESM.zip › Figure 4/4C/shNTC-BFP/35-1.tif]

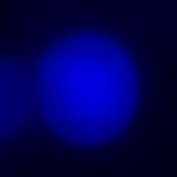

Supplement: Supplementary file 9 — Source data Fig. 4 [file 44319_2026_815_MOESM9_ESM.zip › Figure 4/4C/shNTC-BFP/35-2.tif]

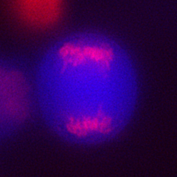

Supplement: Supplementary file 9 — Source data Fig. 4 [file 44319_2026_815_MOESM9_ESM.zip › Figure 4/4C/shNTC-BFP/35-3.tif]

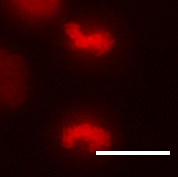

Supplement: Supplementary file 9 — Source data Fig. 4 [file 44319_2026_815_MOESM9_ESM.zip › Figure 4/4C/shNTC-BFP/40-1 bar.tif]

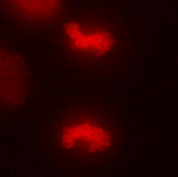

Supplement: Supplementary file 9 — Source data Fig. 4 [file 44319_2026_815_MOESM9_ESM.zip › Figure 4/4C/shNTC-BFP/40-1.tif]

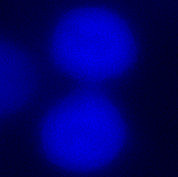

Supplement: Supplementary file 9 — Source data Fig. 4 [file 44319_2026_815_MOESM9_ESM.zip › Figure 4/4C/shNTC-BFP/40-2.tif]

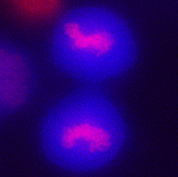

Supplement: Supplementary file 9 — Source data Fig. 4 [file 44319_2026_815_MOESM9_ESM.zip › Figure 4/4C/shNTC-BFP/40-3.tif]

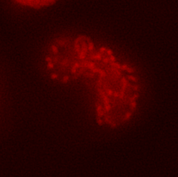

Supplement: Supplementary file 9 — Source data Fig. 4 [file 44319_2026_815_MOESM9_ESM.zip › Figure 4/4C/shNTC-BFP/5-1.tif]

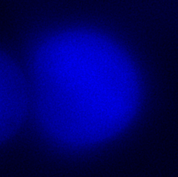

Supplement: Supplementary file 9 — Source data Fig. 4 [file 44319_2026_815_MOESM9_ESM.zip › Figure 4/4C/shNTC-BFP/5-2.tif]

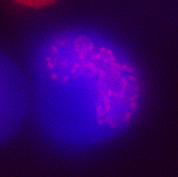

Supplement: Supplementary file 9 — Source data Fig. 4 [file 44319_2026_815_MOESM9_ESM.zip › Figure 4/4C/shNTC-BFP/5-3.tif]

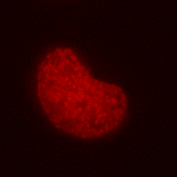

Supplement: Supplementary file 9 — Source data Fig. 4 [file 44319_2026_815_MOESM9_ESM.zip › Figure 4/4C/shWTAP chromosome bridge/0-1.tif]

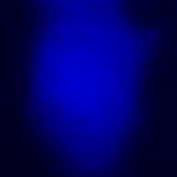

Supplement: Supplementary file 9 — Source data Fig. 4 [file 44319_2026_815_MOESM9_ESM.zip › Figure 4/4C/shWTAP chromosome bridge/0-2.tif]

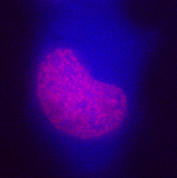

Supplement: Supplementary file 9 — Source data Fig. 4 [file 44319_2026_815_MOESM9_ESM.zip › Figure 4/4C/shWTAP chromosome bridge/0-3.tif]

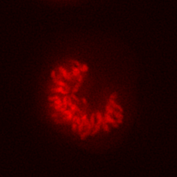

Supplement: Supplementary file 9 — Source data Fig. 4 [file 44319_2026_815_MOESM9_ESM.zip › Figure 4/4C/shWTAP chromosome bridge/10-1.tif]

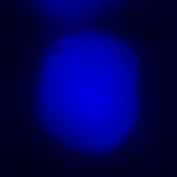

Supplement: Supplementary file 9 — Source data Fig. 4 [file 44319_2026_815_MOESM9_ESM.zip › Figure 4/4C/shWTAP chromosome bridge/10-2.tif]

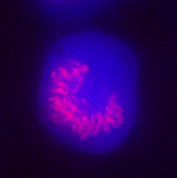

Supplement: Supplementary file 9 — Source data Fig. 4 [file 44319_2026_815_MOESM9_ESM.zip › Figure 4/4C/shWTAP chromosome bridge/10-3.tif]

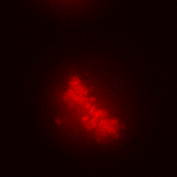

Supplement: Supplementary file 9 — Source data Fig. 4 [file 44319_2026_815_MOESM9_ESM.zip › Figure 4/4C/shWTAP chromosome bridge/20-1.tif]

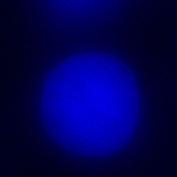

Supplement: Supplementary file 9 — Source data Fig. 4 [file 44319_2026_815_MOESM9_ESM.zip › Figure 4/4C/shWTAP chromosome bridge/20-2.tif]

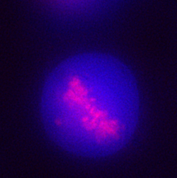

Supplement: Supplementary file 9 — Source data Fig. 4 [file 44319_2026_815_MOESM9_ESM.zip › Figure 4/4C/shWTAP chromosome bridge/20-3.tif]

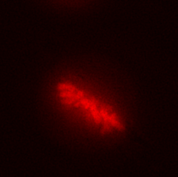

Supplement: Supplementary file 9 — Source data Fig. 4 [file 44319_2026_815_MOESM9_ESM.zip › Figure 4/4C/shWTAP chromosome bridge/25-1.tif]

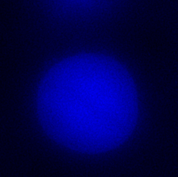

Supplement: Supplementary file 9 — Source data Fig. 4 [file 44319_2026_815_MOESM9_ESM.zip › Figure 4/4C/shWTAP chromosome bridge/25-2.tif]

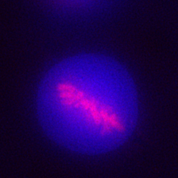

Supplement: Supplementary file 9 — Source data Fig. 4 [file 44319_2026_815_MOESM9_ESM.zip › Figure 4/4C/shWTAP chromosome bridge/25-3.tif]

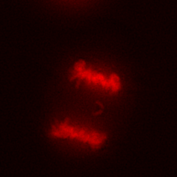

Supplement: Supplementary file 9 — Source data Fig. 4 [file 44319_2026_815_MOESM9_ESM.zip › Figure 4/4C/shWTAP chromosome bridge/35-1.tif]

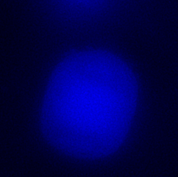

Supplement: Supplementary file 9 — Source data Fig. 4 [file 44319_2026_815_MOESM9_ESM.zip › Figure 4/4C/shWTAP chromosome bridge/35-2.tif]

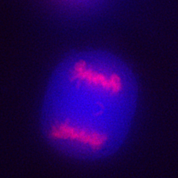

Supplement: Supplementary file 9 — Source data Fig. 4 [file 44319_2026_815_MOESM9_ESM.zip › Figure 4/4C/shWTAP chromosome bridge/35-3.tif]

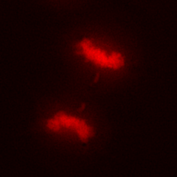

Supplement: Supplementary file 9 — Source data Fig. 4 [file 44319_2026_815_MOESM9_ESM.zip › Figure 4/4C/shWTAP chromosome bridge/40-1.tif]

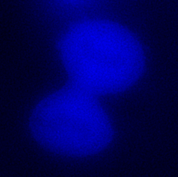

Supplement: Supplementary file 9 — Source data Fig. 4 [file 44319_2026_815_MOESM9_ESM.zip › Figure 4/4C/shWTAP chromosome bridge/40-2.tif]

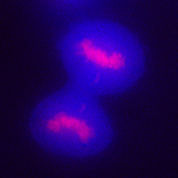

Supplement: Supplementary file 9 — Source data Fig. 4 [file 44319_2026_815_MOESM9_ESM.zip › Figure 4/4C/shWTAP chromosome bridge/40-3.tif]

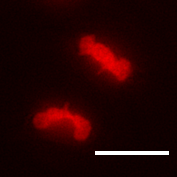

Supplement: Supplementary file 9 — Source data Fig. 4 [file 44319_2026_815_MOESM9_ESM.zip › Figure 4/4C/shWTAP chromosome bridge/45-1 bar.tif]

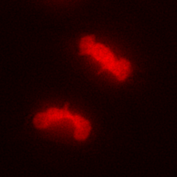

Supplement: Supplementary file 9 — Source data Fig. 4 [file 44319_2026_815_MOESM9_ESM.zip › Figure 4/4C/shWTAP chromosome bridge/45-1.tif]

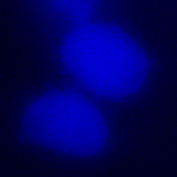

Supplement: Supplementary file 9 — Source data Fig. 4 [file 44319_2026_815_MOESM9_ESM.zip › Figure 4/4C/shWTAP chromosome bridge/45-2.tif]

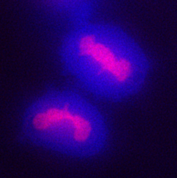

Supplement: Supplementary file 9 — Source data Fig. 4 [file 44319_2026_815_MOESM9_ESM.zip › Figure 4/4C/shWTAP chromosome bridge/45-3.tif]

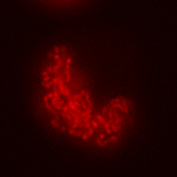

Supplement: Supplementary file 9 — Source data Fig. 4 [file 44319_2026_815_MOESM9_ESM.zip › Figure 4/4C/shWTAP chromosome bridge/5-1.tif]

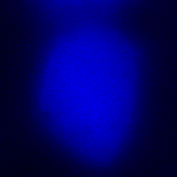

Supplement: Supplementary file 9 — Source data Fig. 4 [file 44319_2026_815_MOESM9_ESM.zip › Figure 4/4C/shWTAP chromosome bridge/5-2.tif]

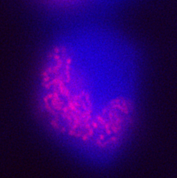

Supplement: Supplementary file 9 — Source data Fig. 4 [file 44319_2026_815_MOESM9_ESM.zip › Figure 4/4C/shWTAP chromosome bridge/5-3.tif]

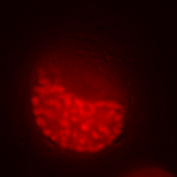

Supplement: Supplementary file 9 — Source data Fig. 4 [file 44319_2026_815_MOESM9_ESM.zip › Figure 4/4C/shWTAP lagging of chromosome/0-1.tif]

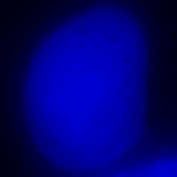

Supplement: Supplementary file 9 — Source data Fig. 4 [file 44319_2026_815_MOESM9_ESM.zip › Figure 4/4C/shWTAP lagging of chromosome/0-2.tif]

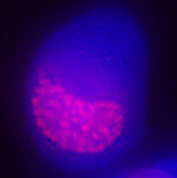

Supplement: Supplementary file 9 — Source data Fig. 4 [file 44319_2026_815_MOESM9_ESM.zip › Figure 4/4C/shWTAP lagging of chromosome/0-3.tif]

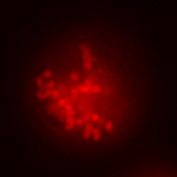

Supplement: Supplementary file 9 — Source data Fig. 4 [file 44319_2026_815_MOESM9_ESM.zip › Figure 4/4C/shWTAP lagging of chromosome/10-1.tif]

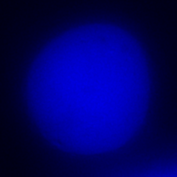

Supplement: Supplementary file 9 — Source data Fig. 4 [file 44319_2026_815_MOESM9_ESM.zip › Figure 4/4C/shWTAP lagging of chromosome/10-2.tif]

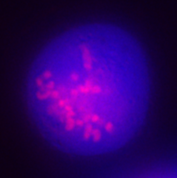

Supplement: Supplementary file 9 — Source data Fig. 4 [file 44319_2026_815_MOESM9_ESM.zip › Figure 4/4C/shWTAP lagging of chromosome/10-3.tif]

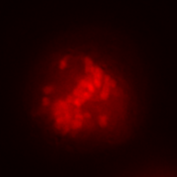

Supplement: Supplementary file 9 — Source data Fig. 4 [file 44319_2026_815_MOESM9_ESM.zip › Figure 4/4C/shWTAP lagging of chromosome/15-1.tif]

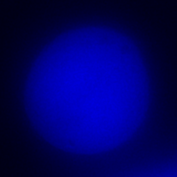

Supplement: Supplementary file 9 — Source data Fig. 4 [file 44319_2026_815_MOESM9_ESM.zip › Figure 4/4C/shWTAP lagging of chromosome/15-2.tif]

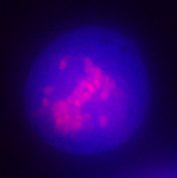

Supplement: Supplementary file 9 — Source data Fig. 4 [file 44319_2026_815_MOESM9_ESM.zip › Figure 4/4C/shWTAP lagging of chromosome/15-3.tif]

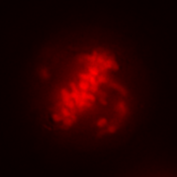

Supplement: Supplementary file 9 — Source data Fig. 4 [file 44319_2026_815_MOESM9_ESM.zip › Figure 4/4C/shWTAP lagging of chromosome/25-1.tif]

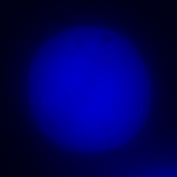

Supplement: Supplementary file 9 — Source data Fig. 4 [file 44319_2026_815_MOESM9_ESM.zip › Figure 4/4C/shWTAP lagging of chromosome/25-2.tif]

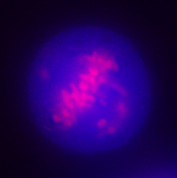

Supplement: Supplementary file 9 — Source data Fig. 4 [file 44319_2026_815_MOESM9_ESM.zip › Figure 4/4C/shWTAP lagging of chromosome/25-3.tif]

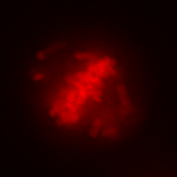

Supplement: Supplementary file 9 — Source data Fig. 4 [file 44319_2026_815_MOESM9_ESM.zip › Figure 4/4C/shWTAP lagging of chromosome/35-1.tif]

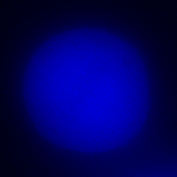

Supplement: Supplementary file 9 — Source data Fig. 4 [file 44319_2026_815_MOESM9_ESM.zip › Figure 4/4C/shWTAP lagging of chromosome/35-2.tif]

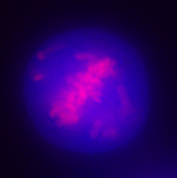

Supplement: Supplementary file 9 — Source data Fig. 4 [file 44319_2026_815_MOESM9_ESM.zip › Figure 4/4C/shWTAP lagging of chromosome/35-3.tif]

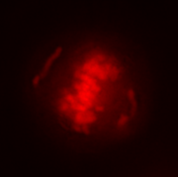

Supplement: Supplementary file 9 — Source data Fig. 4 [file 44319_2026_815_MOESM9_ESM.zip › Figure 4/4C/shWTAP lagging of chromosome/60-1.tif]

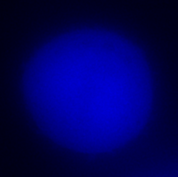

Supplement: Supplementary file 9 — Source data Fig. 4 [file 44319_2026_815_MOESM9_ESM.zip › Figure 4/4C/shWTAP lagging of chromosome/60-2.tif]

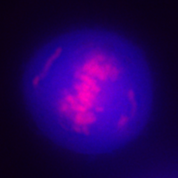

Supplement: Supplementary file 9 — Source data Fig. 4 [file 44319_2026_815_MOESM9_ESM.zip › Figure 4/4C/shWTAP lagging of chromosome/60-3.tif]

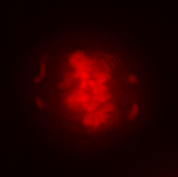

Supplement: Supplementary file 9 — Source data Fig. 4 [file 44319_2026_815_MOESM9_ESM.zip › Figure 4/4C/shWTAP lagging of chromosome/80-1.tif]

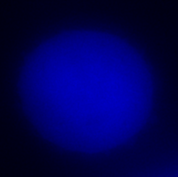

Supplement: Supplementary file 9 — Source data Fig. 4 [file 44319_2026_815_MOESM9_ESM.zip › Figure 4/4C/shWTAP lagging of chromosome/80-2.tif]

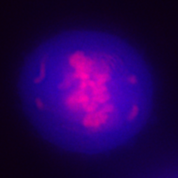

Supplement: Supplementary file 9 — Source data Fig. 4 [file 44319_2026_815_MOESM9_ESM.zip › Figure 4/4C/shWTAP lagging of chromosome/80-3.tif]

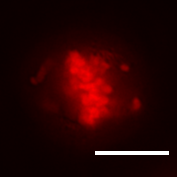

Supplement: Supplementary file 9 — Source data Fig. 4 [file 44319_2026_815_MOESM9_ESM.zip › Figure 4/4C/shWTAP lagging of chromosome/90-1 bar.tif]
