# Supplementary figures and images for: WTAP tetramer ensures m6A writer assembly and faithful mitosis (part 2 of 4)
Source: EMBO Rep. 2026 Jun 2;27(13):3842–62. doi: 10.1038/s44319-026-00815-3 (PMC13354555; doi:10.1038/s44319-026-00815-3)

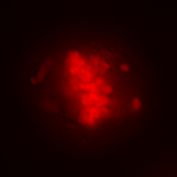

Supplement: Supplementary file 9 — Source data Fig. 4 [file 44319_2026_815_MOESM9_ESM.zip › Figure 4/4C/shWTAP lagging of chromosome/90-1.tif]

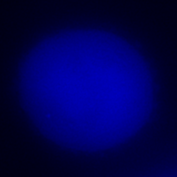

Supplement: Supplementary file 9 — Source data Fig. 4 [file 44319_2026_815_MOESM9_ESM.zip › Figure 4/4C/shWTAP lagging of chromosome/90-2.tif]

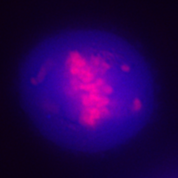

Supplement: Supplementary file 9 — Source data Fig. 4 [file 44319_2026_815_MOESM9_ESM.zip › Figure 4/4C/shWTAP lagging of chromosome/90-3.tif]

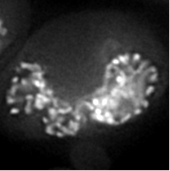

Supplement: Supplementary file 9 — Source data Fig. 4 [file 44319_2026_815_MOESM9_ESM.zip › Figure 4/4E/DMSO/DMSO-0-2.png]

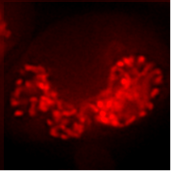

Supplement: Supplementary file 9 — Source data Fig. 4 [file 44319_2026_815_MOESM9_ESM.zip › Figure 4/4E/DMSO/DMSO-0.png]

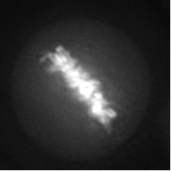

Supplement: Supplementary file 9 — Source data Fig. 4 [file 44319_2026_815_MOESM9_ESM.zip › Figure 4/4E/DMSO/DMSO-18-2.png]

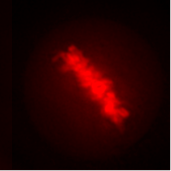

Supplement: Supplementary file 9 — Source data Fig. 4 [file 44319_2026_815_MOESM9_ESM.zip › Figure 4/4E/DMSO/DMSO-18.png]

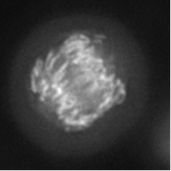

Supplement: Supplementary file 9 — Source data Fig. 4 [file 44319_2026_815_MOESM9_ESM.zip › Figure 4/4E/DMSO/DMSO-27-2.png]

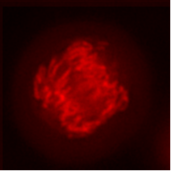

Supplement: Supplementary file 9 — Source data Fig. 4 [file 44319_2026_815_MOESM9_ESM.zip › Figure 4/4E/DMSO/DMSO-27.png]

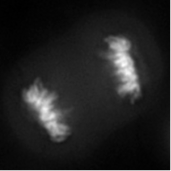

Supplement: Supplementary file 9 — Source data Fig. 4 [file 44319_2026_815_MOESM9_ESM.zip › Figure 4/4E/DMSO/DMSO-33-2.png]

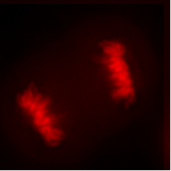

Supplement: Supplementary file 9 — Source data Fig. 4 [file 44319_2026_815_MOESM9_ESM.zip › Figure 4/4E/DMSO/DMSO-33.png]

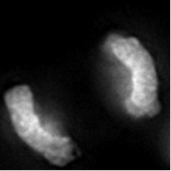

Supplement: Supplementary file 9 — Source data Fig. 4 [file 44319_2026_815_MOESM9_ESM.zip › Figure 4/4E/DMSO/DMSO-42-2.png]

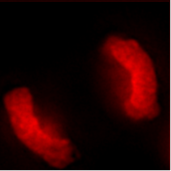

Supplement: Supplementary file 9 — Source data Fig. 4 [file 44319_2026_815_MOESM9_ESM.zip › Figure 4/4E/DMSO/DMSO-42.png]

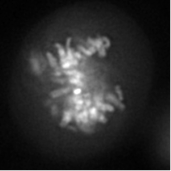

Supplement: Supplementary file 9 — Source data Fig. 4 [file 44319_2026_815_MOESM9_ESM.zip › Figure 4/4E/DMSO/DMSO-6-2.png]

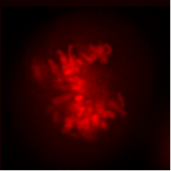

Supplement: Supplementary file 9 — Source data Fig. 4 [file 44319_2026_815_MOESM9_ESM.zip › Figure 4/4E/DMSO/DMSO-6.png]

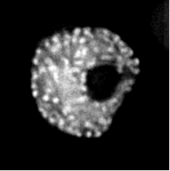

Supplement: Supplementary file 9 — Source data Fig. 4 [file 44319_2026_815_MOESM9_ESM.zip › Figure 4/4E/STM2457 Lagging of chromosome/STM2457-0-2.png]

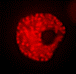

Supplement: Supplementary file 9 — Source data Fig. 4 [file 44319_2026_815_MOESM9_ESM.zip › Figure 4/4E/STM2457 Lagging of chromosome/STM2457-0.png]

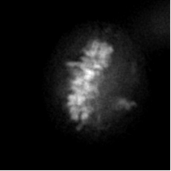

Supplement: Supplementary file 9 — Source data Fig. 4 [file 44319_2026_815_MOESM9_ESM.zip › Figure 4/4E/STM2457 Lagging of chromosome/STM2457-20-2.png]

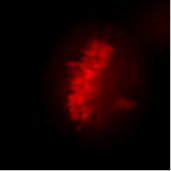

Supplement: Supplementary file 9 — Source data Fig. 4 [file 44319_2026_815_MOESM9_ESM.zip › Figure 4/4E/STM2457 Lagging of chromosome/STM2457-20.png]

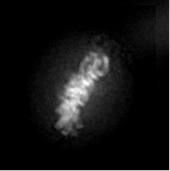

Supplement: Supplementary file 9 — Source data Fig. 4 [file 44319_2026_815_MOESM9_ESM.zip › Figure 4/4E/STM2457 Lagging of chromosome/STM2457-30-2.png]

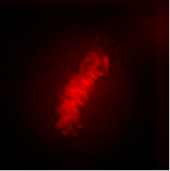

Supplement: Supplementary file 9 — Source data Fig. 4 [file 44319_2026_815_MOESM9_ESM.zip › Figure 4/4E/STM2457 Lagging of chromosome/STM2457-30.png]

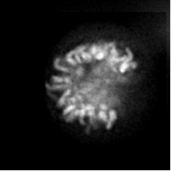

Supplement: Supplementary file 9 — Source data Fig. 4 [file 44319_2026_815_MOESM9_ESM.zip › Figure 4/4E/STM2457 Lagging of chromosome/STM2457-5-2.png]

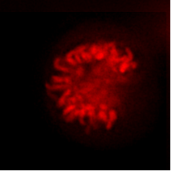

Supplement: Supplementary file 9 — Source data Fig. 4 [file 44319_2026_815_MOESM9_ESM.zip › Figure 4/4E/STM2457 Lagging of chromosome/STM2457-5.png]

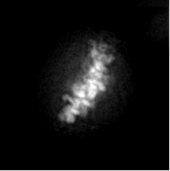

Supplement: Supplementary file 9 — Source data Fig. 4 [file 44319_2026_815_MOESM9_ESM.zip › Figure 4/4E/STM2457 Lagging of chromosome/STM2457-50-2.png]

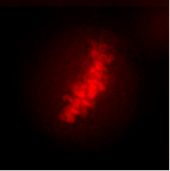

Supplement: Supplementary file 9 — Source data Fig. 4 [file 44319_2026_815_MOESM9_ESM.zip › Figure 4/4E/STM2457 Lagging of chromosome/STM2457-50.png]

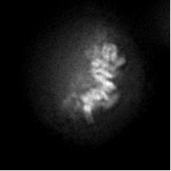

Supplement: Supplementary file 9 — Source data Fig. 4 [file 44319_2026_815_MOESM9_ESM.zip › Figure 4/4E/STM2457 Lagging of chromosome/STM2457-60-2.png]

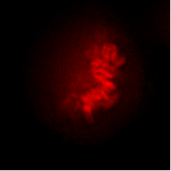

Supplement: Supplementary file 9 — Source data Fig. 4 [file 44319_2026_815_MOESM9_ESM.zip › Figure 4/4E/STM2457 Lagging of chromosome/STM2457-60.png]

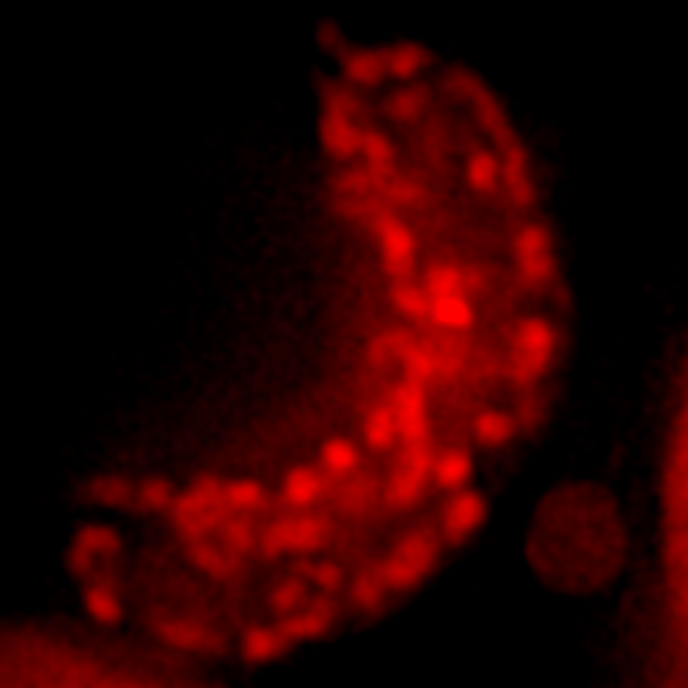

Supplement: Supplementary file 9 — Source data Fig. 4 [file 44319_2026_815_MOESM9_ESM.zip › Figure 4/4E/STM2457 segregation defects/0-1.tif]

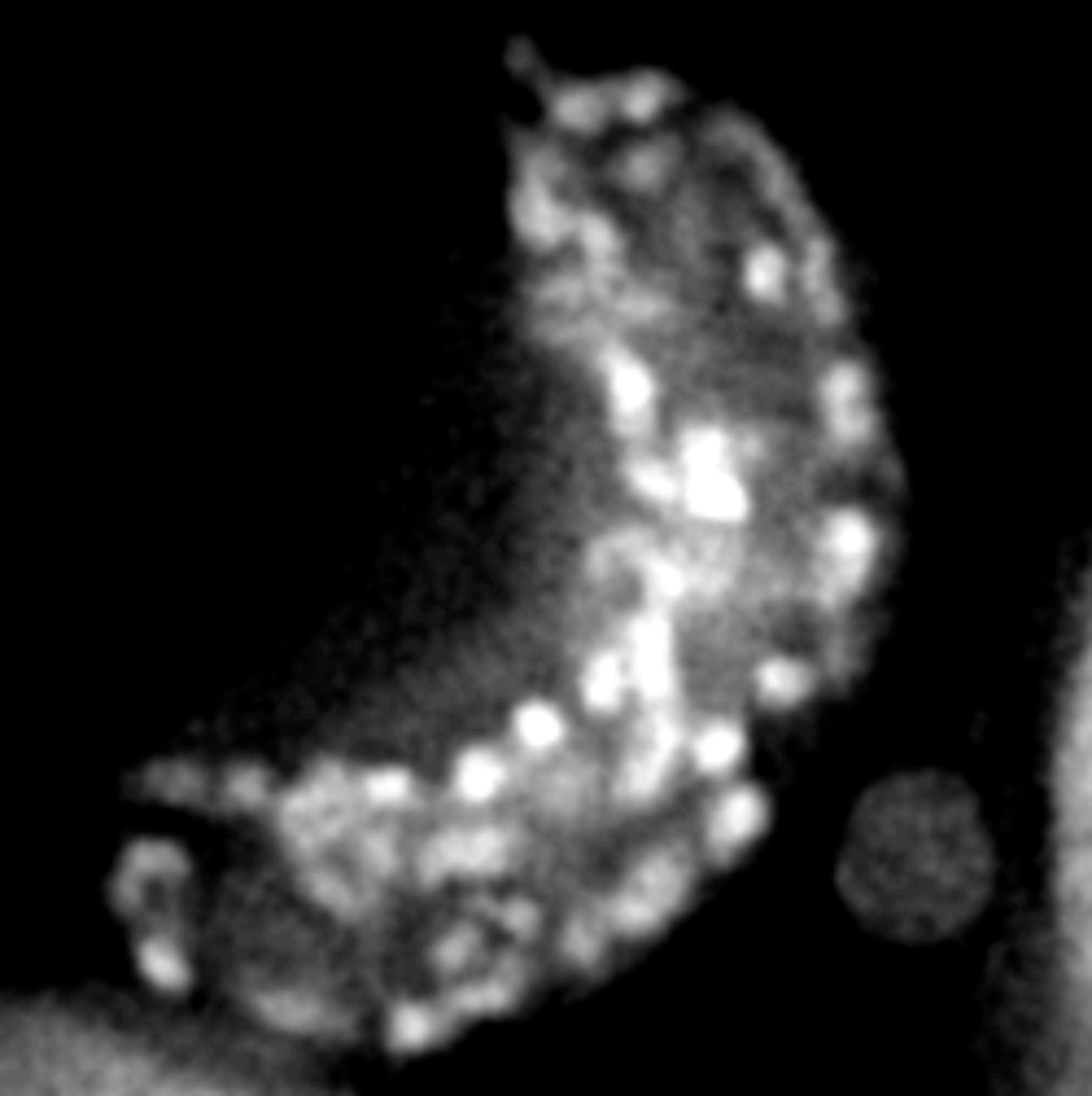

Supplement: Supplementary file 9 — Source data Fig. 4 [file 44319_2026_815_MOESM9_ESM.zip › Figure 4/4E/STM2457 segregation defects/0-2.tif]

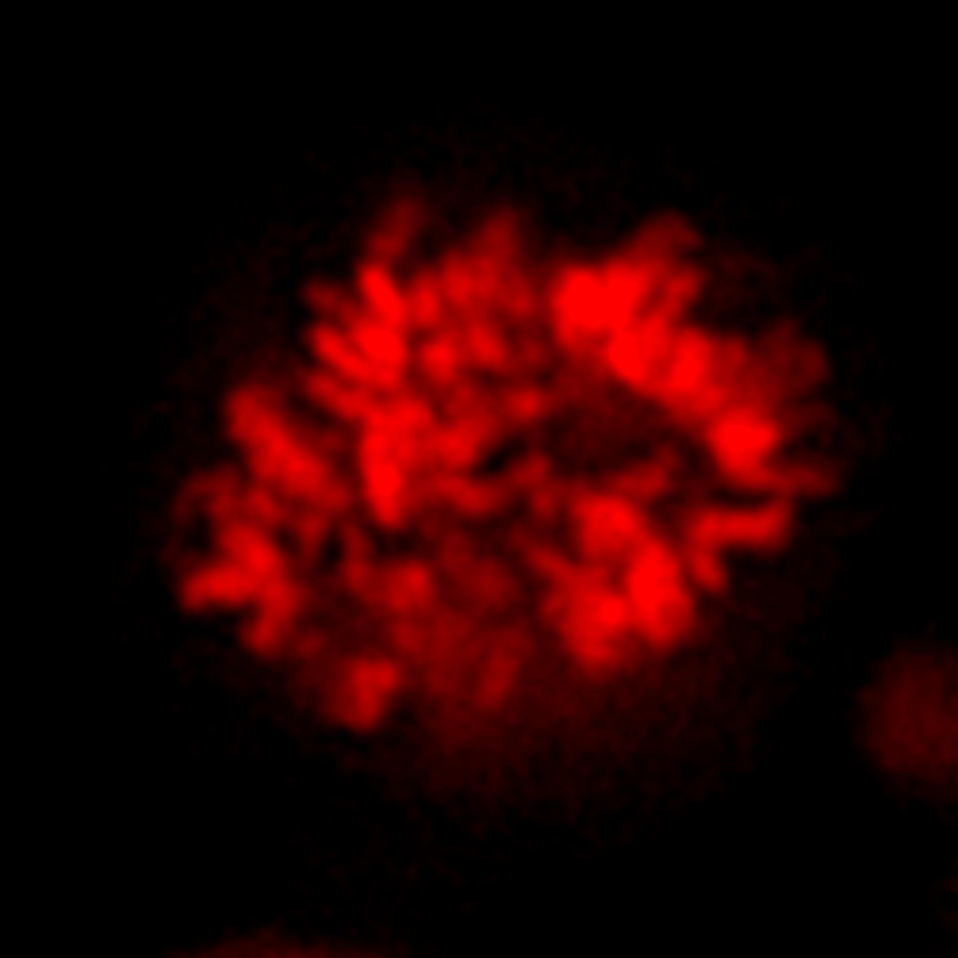

Supplement: Supplementary file 9 — Source data Fig. 4 [file 44319_2026_815_MOESM9_ESM.zip › Figure 4/4E/STM2457 segregation defects/15-1.tif]

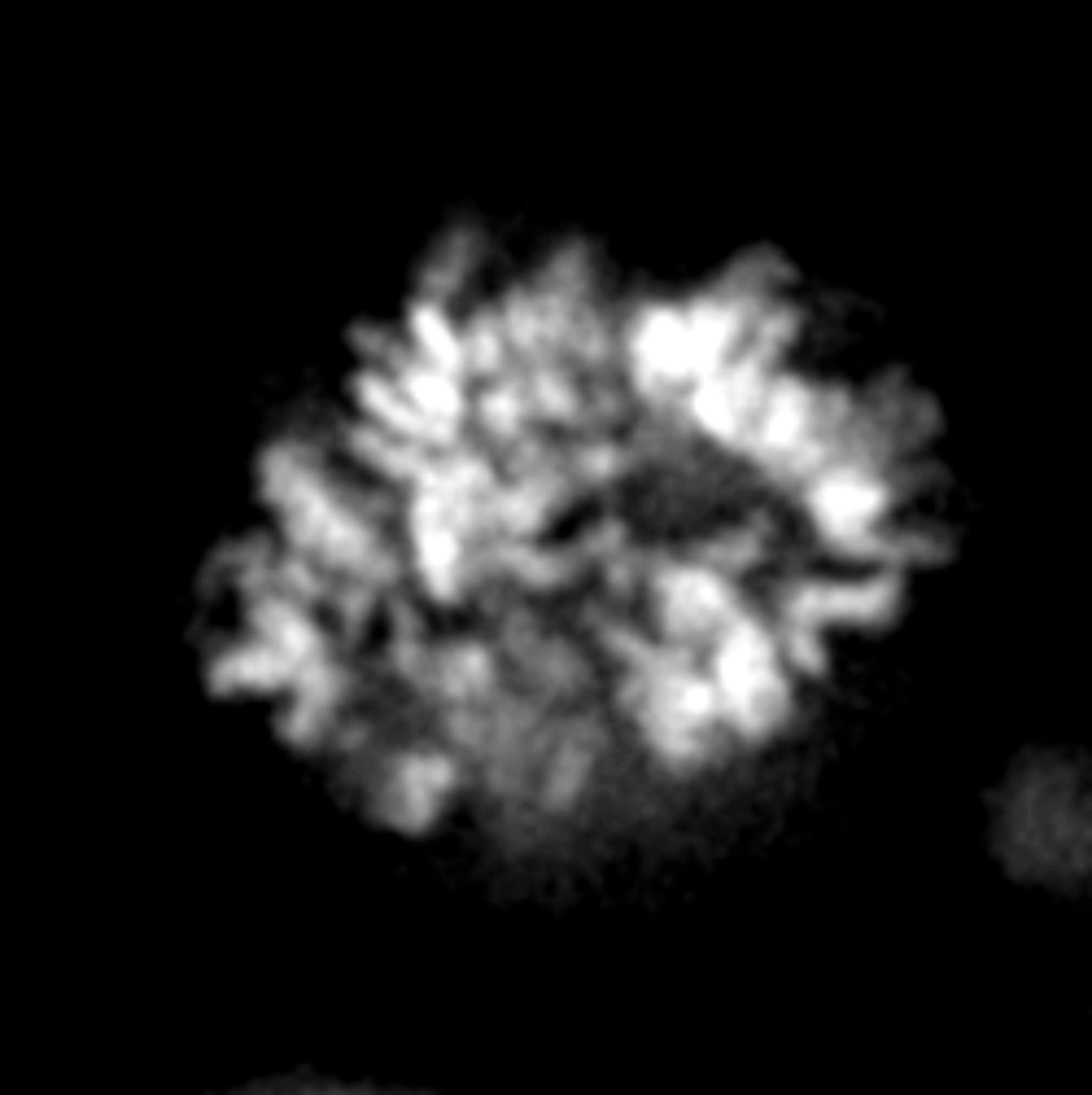

Supplement: Supplementary file 9 — Source data Fig. 4 [file 44319_2026_815_MOESM9_ESM.zip › Figure 4/4E/STM2457 segregation defects/15-2.tif]

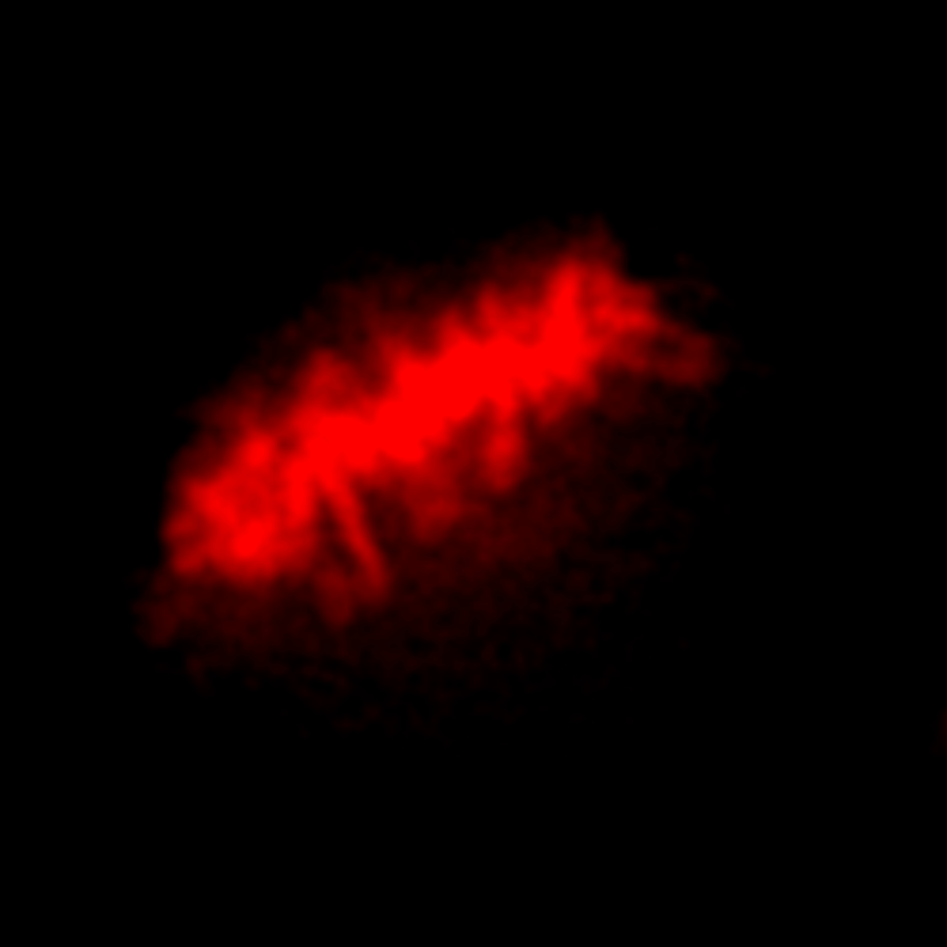

Supplement: Supplementary file 9 — Source data Fig. 4 [file 44319_2026_815_MOESM9_ESM.zip › Figure 4/4E/STM2457 segregation defects/30-1.tif]

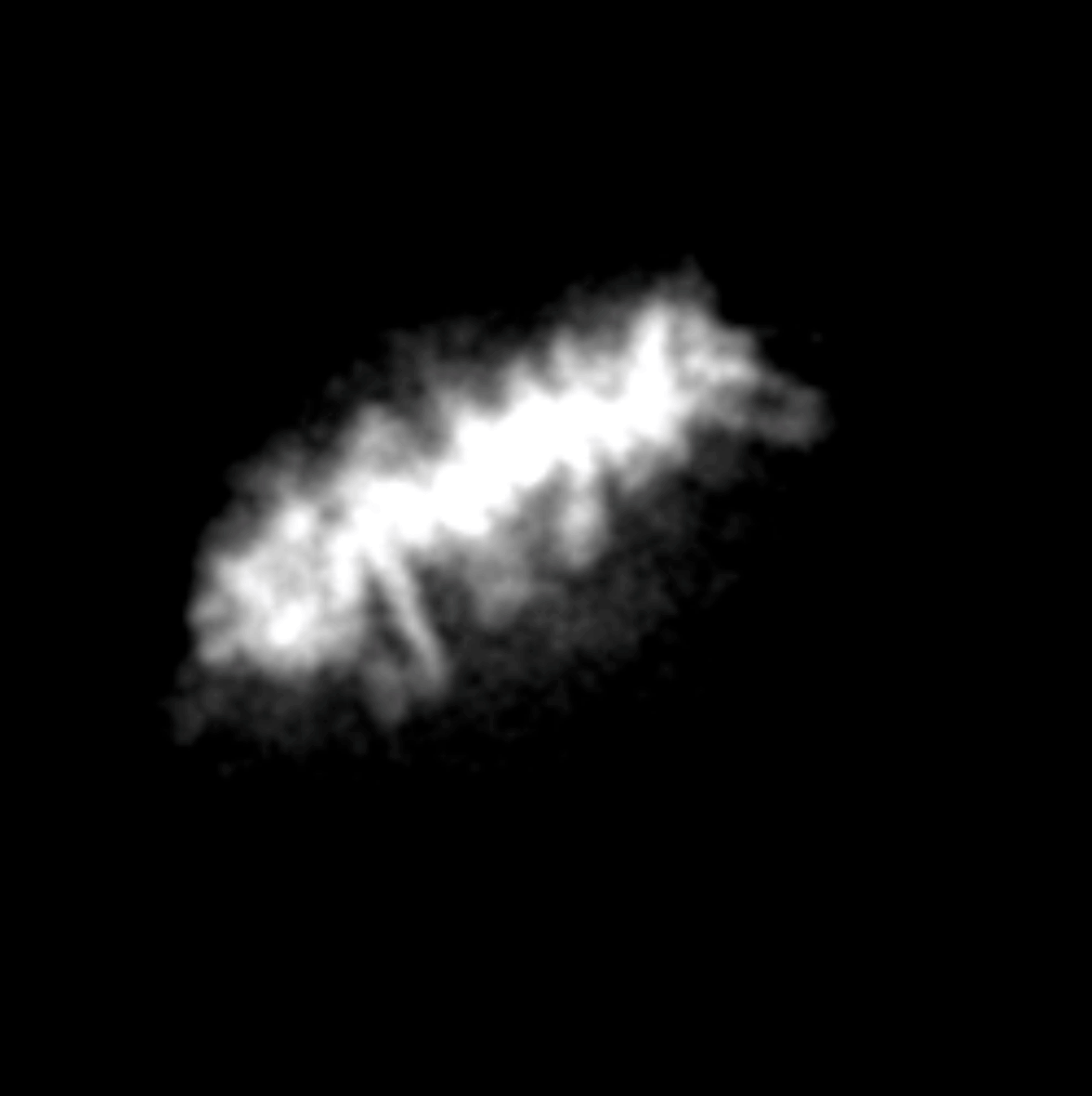

Supplement: Supplementary file 9 — Source data Fig. 4 [file 44319_2026_815_MOESM9_ESM.zip › Figure 4/4E/STM2457 segregation defects/30-2.tif]

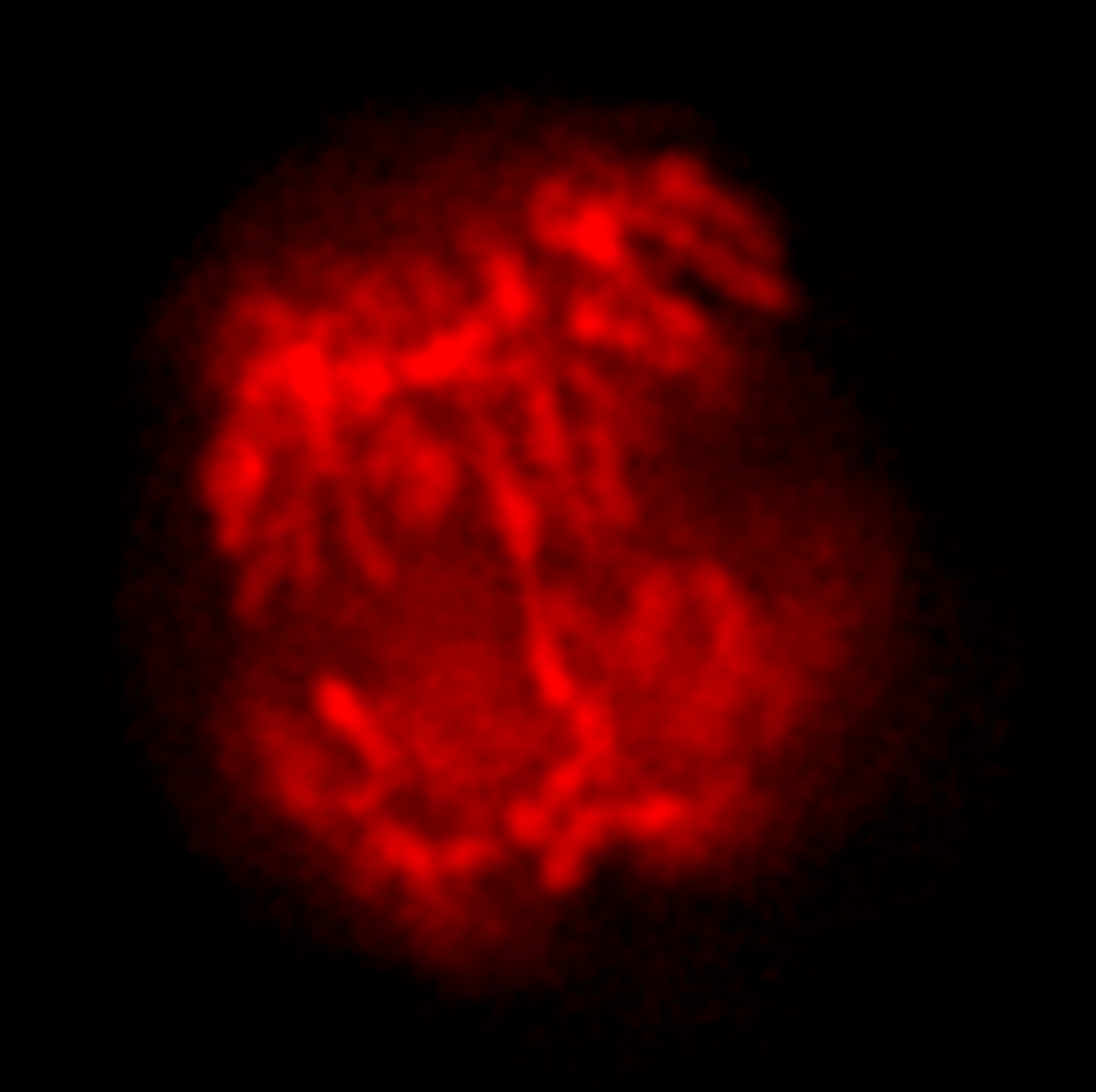

Supplement: Supplementary file 9 — Source data Fig. 4 [file 44319_2026_815_MOESM9_ESM.zip › Figure 4/4E/STM2457 segregation defects/35-1.tif]

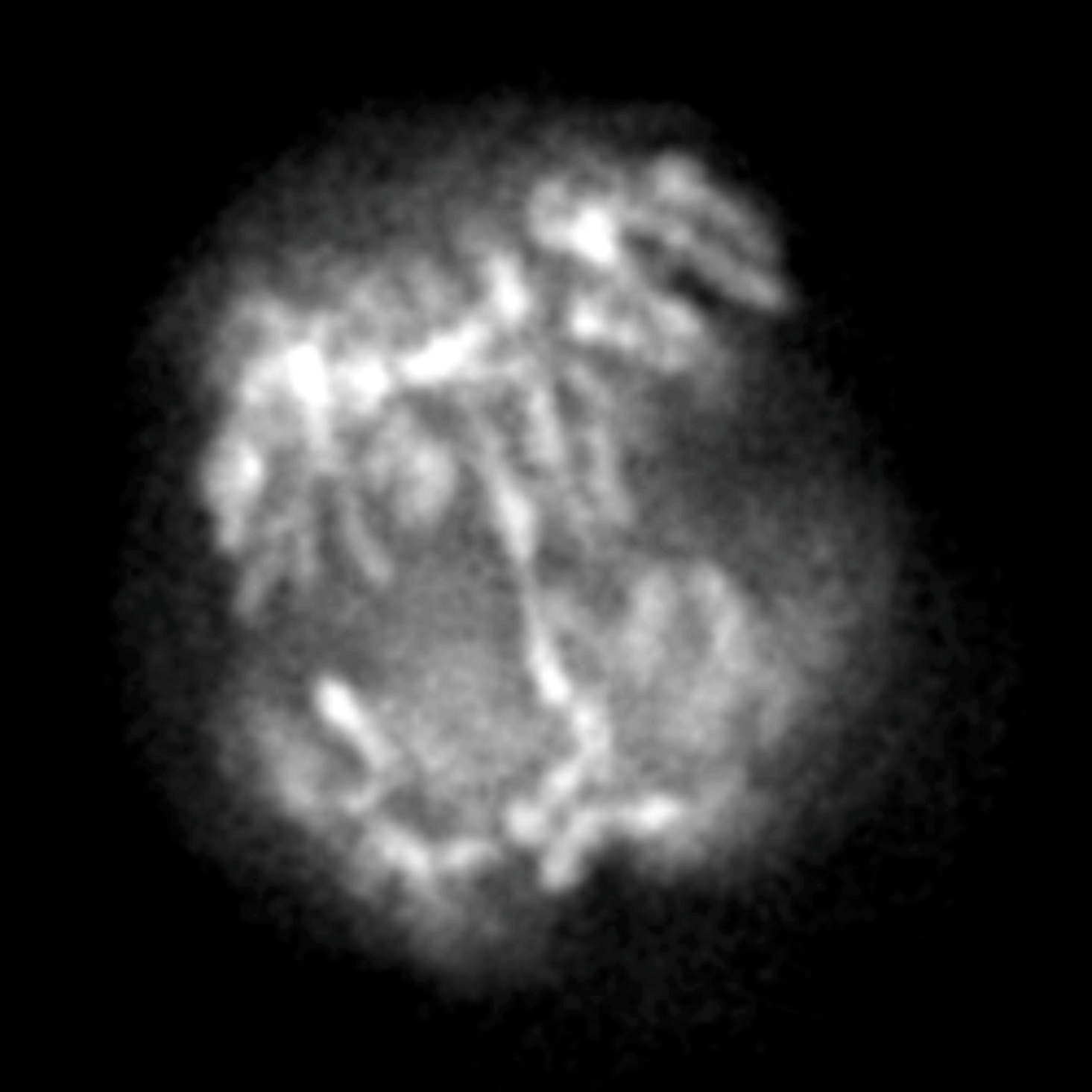

Supplement: Supplementary file 9 — Source data Fig. 4 [file 44319_2026_815_MOESM9_ESM.zip › Figure 4/4E/STM2457 segregation defects/35-2.tif]

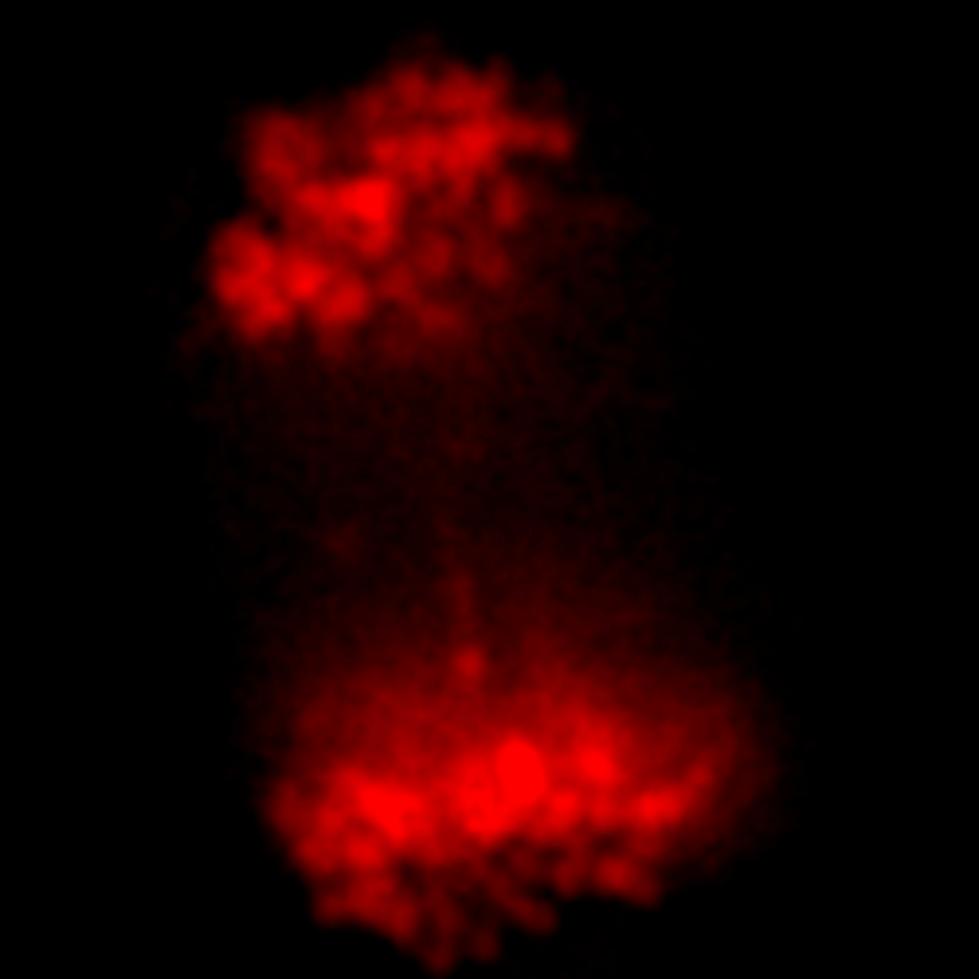

Supplement: Supplementary file 9 — Source data Fig. 4 [file 44319_2026_815_MOESM9_ESM.zip › Figure 4/4E/STM2457 segregation defects/40-1.tif]

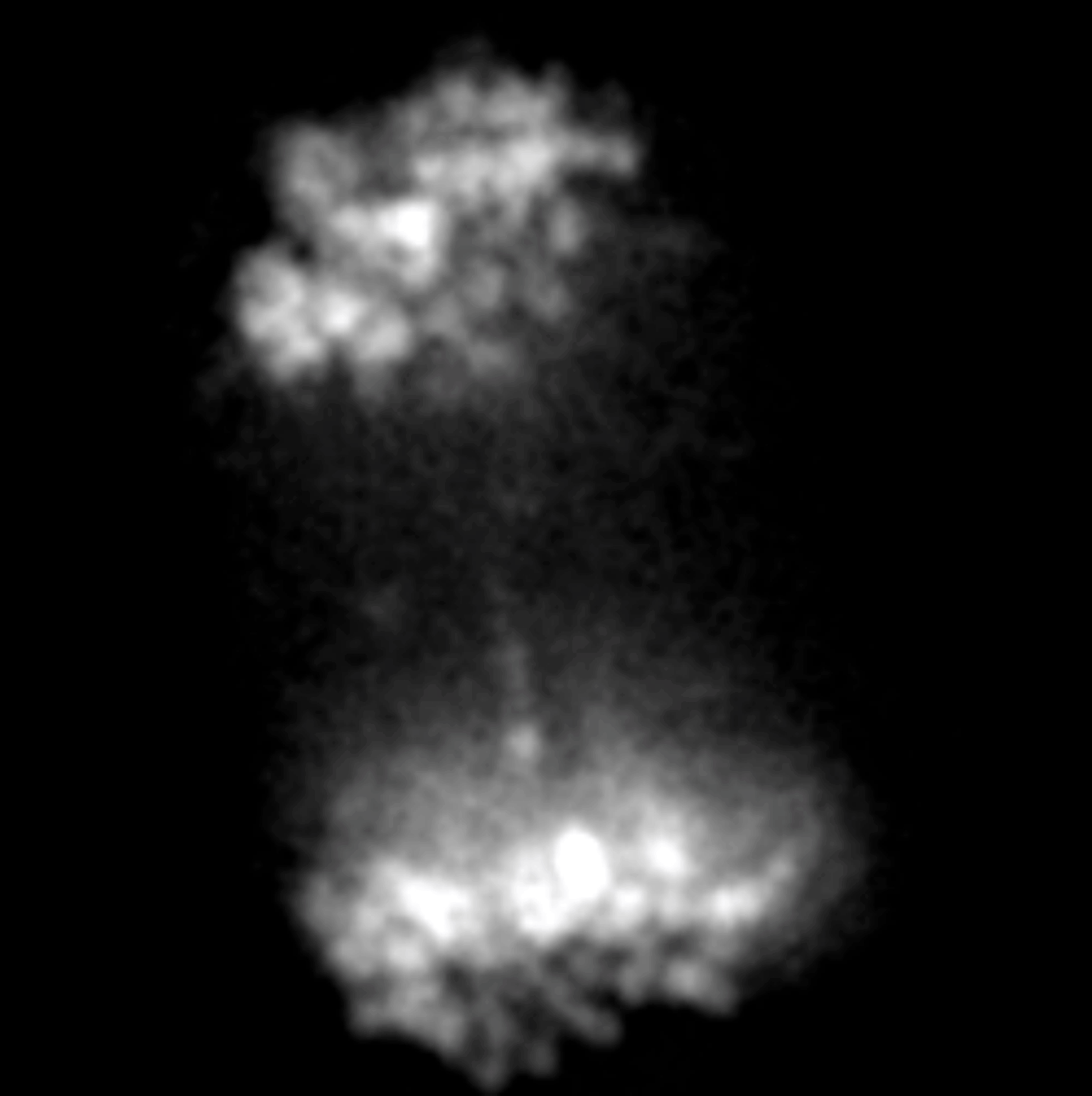

Supplement: Supplementary file 9 — Source data Fig. 4 [file 44319_2026_815_MOESM9_ESM.zip › Figure 4/4E/STM2457 segregation defects/40-2.tif]

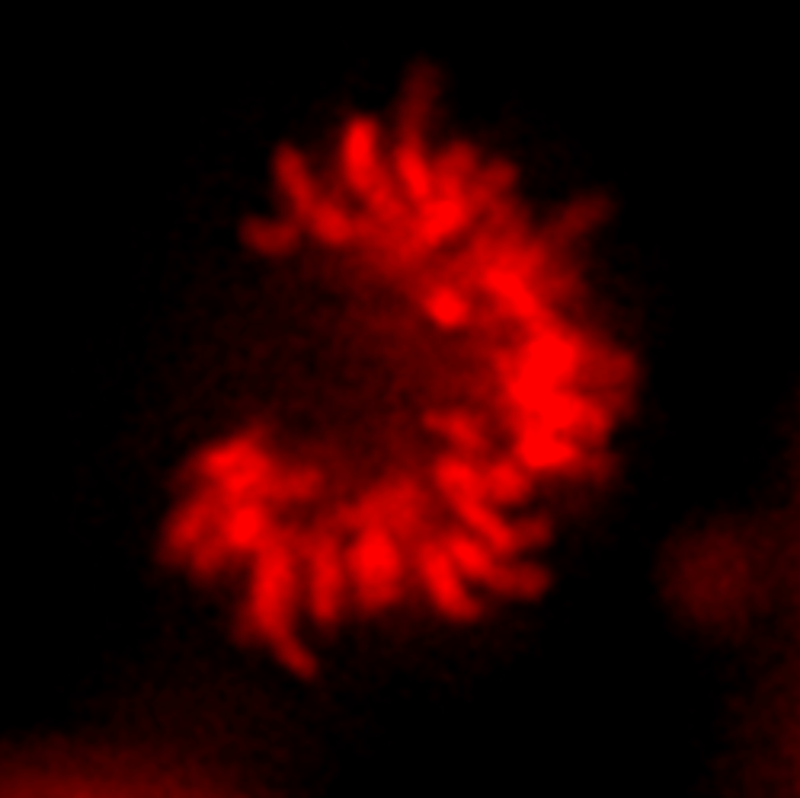

Supplement: Supplementary file 9 — Source data Fig. 4 [file 44319_2026_815_MOESM9_ESM.zip › Figure 4/4E/STM2457 segregation defects/5-1.tif]

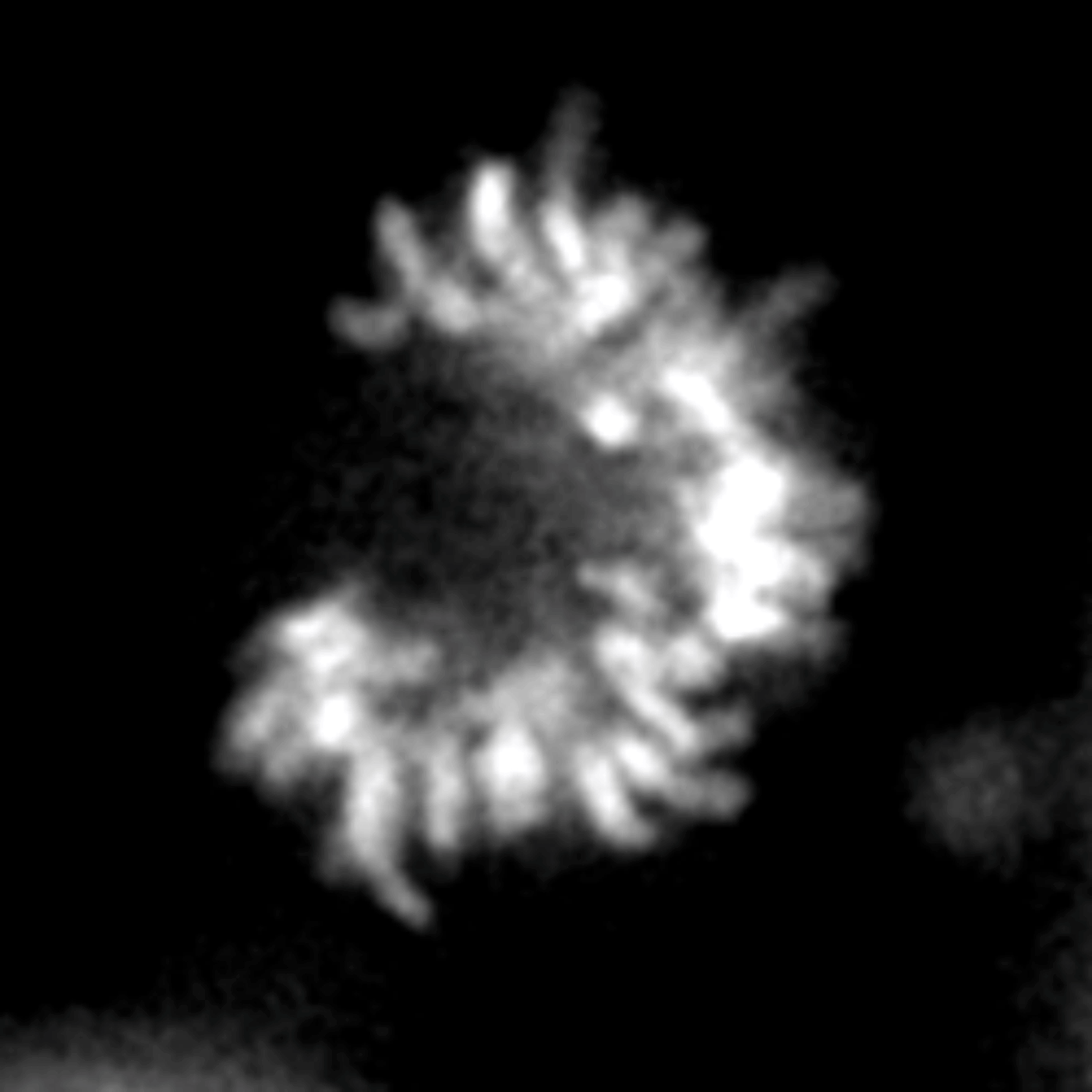

Supplement: Supplementary file 9 — Source data Fig. 4 [file 44319_2026_815_MOESM9_ESM.zip › Figure 4/4E/STM2457 segregation defects/5-2.tif]

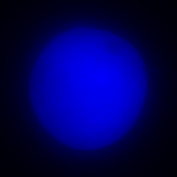

Supplement: Supplementary file 10 — Source data Fig. 5 [file 44319_2026_815_MOESM10_ESM.zip › Figure 5/5A/rescue-GFP/0-1.tif]

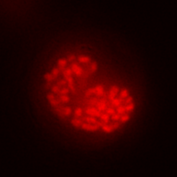

Supplement: Supplementary file 10 — Source data Fig. 5 [file 44319_2026_815_MOESM10_ESM.zip › Figure 5/5A/rescue-GFP/0-2.tif]

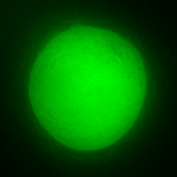

Supplement: Supplementary file 10 — Source data Fig. 5 [file 44319_2026_815_MOESM10_ESM.zip › Figure 5/5A/rescue-GFP/0-3.tif]

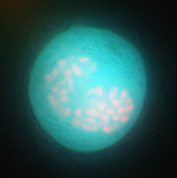

Supplement: Supplementary file 10 — Source data Fig. 5 [file 44319_2026_815_MOESM10_ESM.zip › Figure 5/5A/rescue-GFP/0-4.tif]

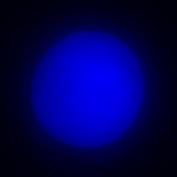

Supplement: Supplementary file 10 — Source data Fig. 5 [file 44319_2026_815_MOESM10_ESM.zip › Figure 5/5A/rescue-GFP/15-1.tif]

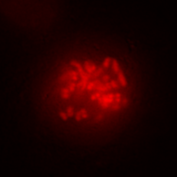

Supplement: Supplementary file 10 — Source data Fig. 5 [file 44319_2026_815_MOESM10_ESM.zip › Figure 5/5A/rescue-GFP/15-2.tif]

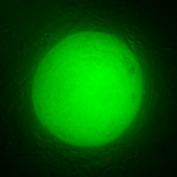

Supplement: Supplementary file 10 — Source data Fig. 5 [file 44319_2026_815_MOESM10_ESM.zip › Figure 5/5A/rescue-GFP/15-3.tif]

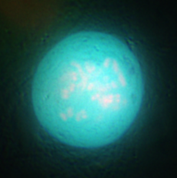

Supplement: Supplementary file 10 — Source data Fig. 5 [file 44319_2026_815_MOESM10_ESM.zip › Figure 5/5A/rescue-GFP/15-4.tif]

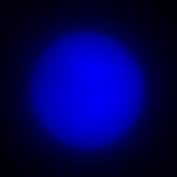

Supplement: Supplementary file 10 — Source data Fig. 5 [file 44319_2026_815_MOESM10_ESM.zip › Figure 5/5A/rescue-GFP/25-1.tif]

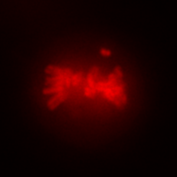

Supplement: Supplementary file 10 — Source data Fig. 5 [file 44319_2026_815_MOESM10_ESM.zip › Figure 5/5A/rescue-GFP/25-2.tif]

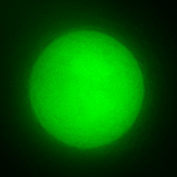

Supplement: Supplementary file 10 — Source data Fig. 5 [file 44319_2026_815_MOESM10_ESM.zip › Figure 5/5A/rescue-GFP/25-3.tif]

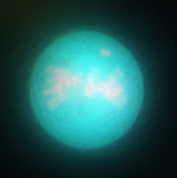

Supplement: Supplementary file 10 — Source data Fig. 5 [file 44319_2026_815_MOESM10_ESM.zip › Figure 5/5A/rescue-GFP/25-4.tif]

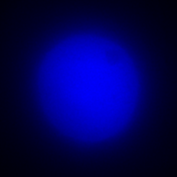

Supplement: Supplementary file 10 — Source data Fig. 5 [file 44319_2026_815_MOESM10_ESM.zip › Figure 5/5A/rescue-GFP/35-1.tif]

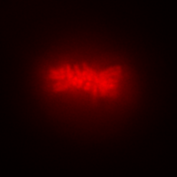

Supplement: Supplementary file 10 — Source data Fig. 5 [file 44319_2026_815_MOESM10_ESM.zip › Figure 5/5A/rescue-GFP/35-2.tif]

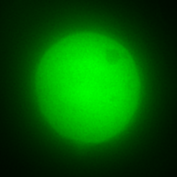

Supplement: Supplementary file 10 — Source data Fig. 5 [file 44319_2026_815_MOESM10_ESM.zip › Figure 5/5A/rescue-GFP/35-3.tif]

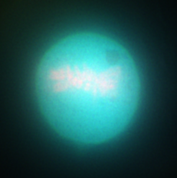

Supplement: Supplementary file 10 — Source data Fig. 5 [file 44319_2026_815_MOESM10_ESM.zip › Figure 5/5A/rescue-GFP/35-4.tif]

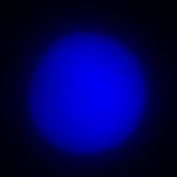

Supplement: Supplementary file 10 — Source data Fig. 5 [file 44319_2026_815_MOESM10_ESM.zip › Figure 5/5A/rescue-GFP/5-1.tif]

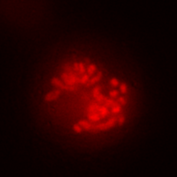

Supplement: Supplementary file 10 — Source data Fig. 5 [file 44319_2026_815_MOESM10_ESM.zip › Figure 5/5A/rescue-GFP/5-2.tif]

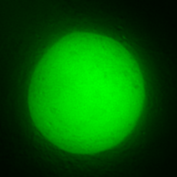

Supplement: Supplementary file 10 — Source data Fig. 5 [file 44319_2026_815_MOESM10_ESM.zip › Figure 5/5A/rescue-GFP/5-3.tif]

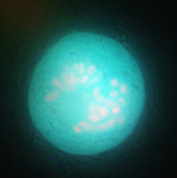

Supplement: Supplementary file 10 — Source data Fig. 5 [file 44319_2026_815_MOESM10_ESM.zip › Figure 5/5A/rescue-GFP/5-4.tif]

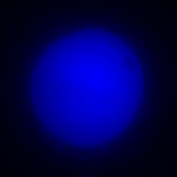

Supplement: Supplementary file 10 — Source data Fig. 5 [file 44319_2026_815_MOESM10_ESM.zip › Figure 5/5A/rescue-GFP/65-1.tif]

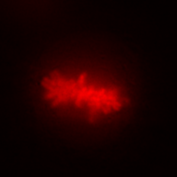

Supplement: Supplementary file 10 — Source data Fig. 5 [file 44319_2026_815_MOESM10_ESM.zip › Figure 5/5A/rescue-GFP/65-2.tif]

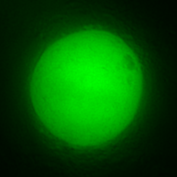

Supplement: Supplementary file 10 — Source data Fig. 5 [file 44319_2026_815_MOESM10_ESM.zip › Figure 5/5A/rescue-GFP/65-3.tif]

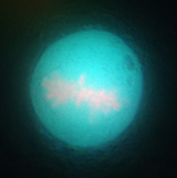

Supplement: Supplementary file 10 — Source data Fig. 5 [file 44319_2026_815_MOESM10_ESM.zip › Figure 5/5A/rescue-GFP/65-4.tif]

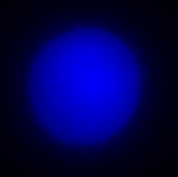

Supplement: Supplementary file 10 — Source data Fig. 5 [file 44319_2026_815_MOESM10_ESM.zip › Figure 5/5A/rescue-GFP/75-1.tif]

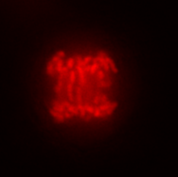

Supplement: Supplementary file 10 — Source data Fig. 5 [file 44319_2026_815_MOESM10_ESM.zip › Figure 5/5A/rescue-GFP/75-2.tif]

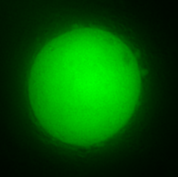

Supplement: Supplementary file 10 — Source data Fig. 5 [file 44319_2026_815_MOESM10_ESM.zip › Figure 5/5A/rescue-GFP/75-3.tif]

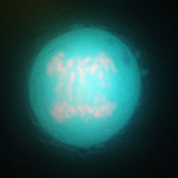

Supplement: Supplementary file 10 — Source data Fig. 5 [file 44319_2026_815_MOESM10_ESM.zip › Figure 5/5A/rescue-GFP/75-4.tif]

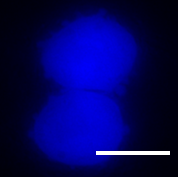

Supplement: Supplementary file 10 — Source data Fig. 5 [file 44319_2026_815_MOESM10_ESM.zip › Figure 5/5A/rescue-GFP/90-1 bar.tif]

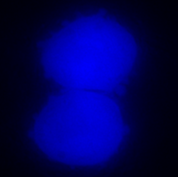

Supplement: Supplementary file 10 — Source data Fig. 5 [file 44319_2026_815_MOESM10_ESM.zip › Figure 5/5A/rescue-GFP/90-1.tif]

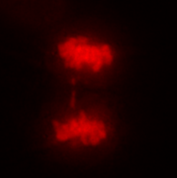

Supplement: Supplementary file 10 — Source data Fig. 5 [file 44319_2026_815_MOESM10_ESM.zip › Figure 5/5A/rescue-GFP/90-2.tif]

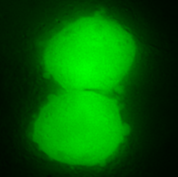

Supplement: Supplementary file 10 — Source data Fig. 5 [file 44319_2026_815_MOESM10_ESM.zip › Figure 5/5A/rescue-GFP/90-3.tif]

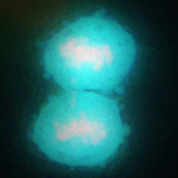

Supplement: Supplementary file 10 — Source data Fig. 5 [file 44319_2026_815_MOESM10_ESM.zip › Figure 5/5A/rescue-GFP/90-4.tif]

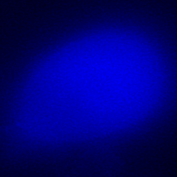

Supplement: Supplementary file 10 — Source data Fig. 5 [file 44319_2026_815_MOESM10_ESM.zip › Figure 5/5A/rescue-L2E/0-1.tif]

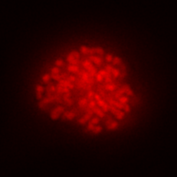

Supplement: Supplementary file 10 — Source data Fig. 5 [file 44319_2026_815_MOESM10_ESM.zip › Figure 5/5A/rescue-L2E/0-2.tif]

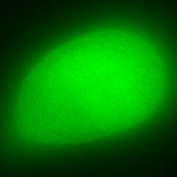

Supplement: Supplementary file 10 — Source data Fig. 5 [file 44319_2026_815_MOESM10_ESM.zip › Figure 5/5A/rescue-L2E/0-3.tif]

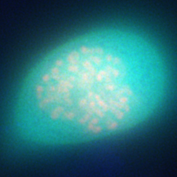

Supplement: Supplementary file 10 — Source data Fig. 5 [file 44319_2026_815_MOESM10_ESM.zip › Figure 5/5A/rescue-L2E/0-4.tif]

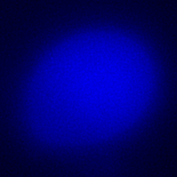

Supplement: Supplementary file 10 — Source data Fig. 5 [file 44319_2026_815_MOESM10_ESM.zip › Figure 5/5A/rescue-L2E/10-1.tif]

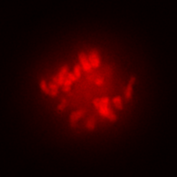

Supplement: Supplementary file 10 — Source data Fig. 5 [file 44319_2026_815_MOESM10_ESM.zip › Figure 5/5A/rescue-L2E/10-2.tif]

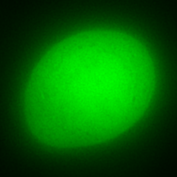

Supplement: Supplementary file 10 — Source data Fig. 5 [file 44319_2026_815_MOESM10_ESM.zip › Figure 5/5A/rescue-L2E/10-3.tif]

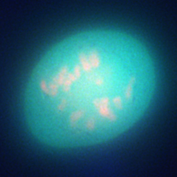

Supplement: Supplementary file 10 — Source data Fig. 5 [file 44319_2026_815_MOESM10_ESM.zip › Figure 5/5A/rescue-L2E/10-4.tif]

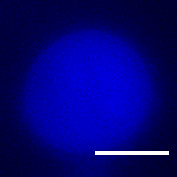

Supplement: Supplementary file 10 — Source data Fig. 5 [file 44319_2026_815_MOESM10_ESM.zip › Figure 5/5A/rescue-L2E/100-1 bar.tif]

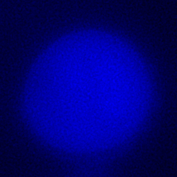

Supplement: Supplementary file 10 — Source data Fig. 5 [file 44319_2026_815_MOESM10_ESM.zip › Figure 5/5A/rescue-L2E/100-1.tif]

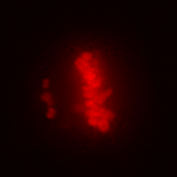

Supplement: Supplementary file 10 — Source data Fig. 5 [file 44319_2026_815_MOESM10_ESM.zip › Figure 5/5A/rescue-L2E/100-2.tif]

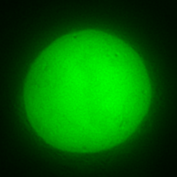

Supplement: Supplementary file 10 — Source data Fig. 5 [file 44319_2026_815_MOESM10_ESM.zip › Figure 5/5A/rescue-L2E/100-3.tif]

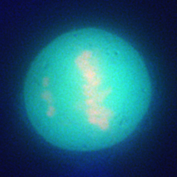

Supplement: Supplementary file 10 — Source data Fig. 5 [file 44319_2026_815_MOESM10_ESM.zip › Figure 5/5A/rescue-L2E/100-4.tif]

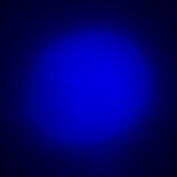

Supplement: Supplementary file 10 — Source data Fig. 5 [file 44319_2026_815_MOESM10_ESM.zip › Figure 5/5A/rescue-L2E/15-1.tif]

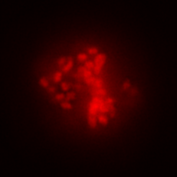

Supplement: Supplementary file 10 — Source data Fig. 5 [file 44319_2026_815_MOESM10_ESM.zip › Figure 5/5A/rescue-L2E/15-2.tif]

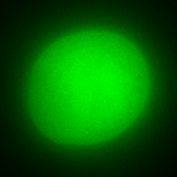

Supplement: Supplementary file 10 — Source data Fig. 5 [file 44319_2026_815_MOESM10_ESM.zip › Figure 5/5A/rescue-L2E/15-3.tif]

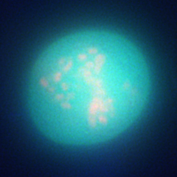

Supplement: Supplementary file 10 — Source data Fig. 5 [file 44319_2026_815_MOESM10_ESM.zip › Figure 5/5A/rescue-L2E/15-4.tif]

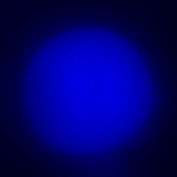

Supplement: Supplementary file 10 — Source data Fig. 5 [file 44319_2026_815_MOESM10_ESM.zip › Figure 5/5A/rescue-L2E/25-1.tif]

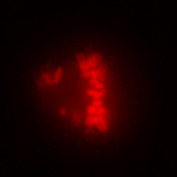

Supplement: Supplementary file 10 — Source data Fig. 5 [file 44319_2026_815_MOESM10_ESM.zip › Figure 5/5A/rescue-L2E/25-2.tif]

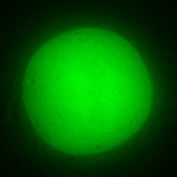

Supplement: Supplementary file 10 — Source data Fig. 5 [file 44319_2026_815_MOESM10_ESM.zip › Figure 5/5A/rescue-L2E/25-3.tif]

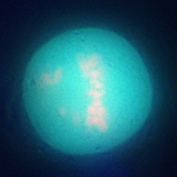

Supplement: Supplementary file 10 — Source data Fig. 5 [file 44319_2026_815_MOESM10_ESM.zip › Figure 5/5A/rescue-L2E/25-4.tif]

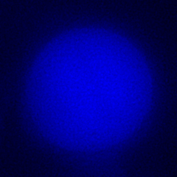

Supplement: Supplementary file 10 — Source data Fig. 5 [file 44319_2026_815_MOESM10_ESM.zip › Figure 5/5A/rescue-L2E/40-1.tif]

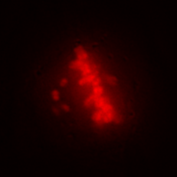

Supplement: Supplementary file 10 — Source data Fig. 5 [file 44319_2026_815_MOESM10_ESM.zip › Figure 5/5A/rescue-L2E/40-2.tif]

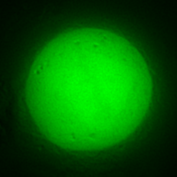

Supplement: Supplementary file 10 — Source data Fig. 5 [file 44319_2026_815_MOESM10_ESM.zip › Figure 5/5A/rescue-L2E/40-3.tif]

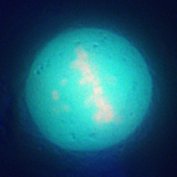

Supplement: Supplementary file 10 — Source data Fig. 5 [file 44319_2026_815_MOESM10_ESM.zip › Figure 5/5A/rescue-L2E/40-4.tif]

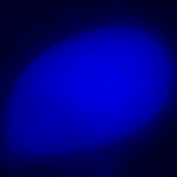

Supplement: Supplementary file 10 — Source data Fig. 5 [file 44319_2026_815_MOESM10_ESM.zip › Figure 5/5A/rescue-L2E/5-1.tif]

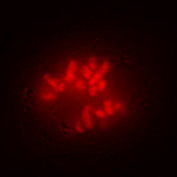

Supplement: Supplementary file 10 — Source data Fig. 5 [file 44319_2026_815_MOESM10_ESM.zip › Figure 5/5A/rescue-L2E/5-2.tif]

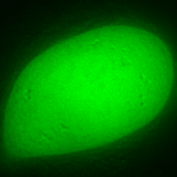

Supplement: Supplementary file 10 — Source data Fig. 5 [file 44319_2026_815_MOESM10_ESM.zip › Figure 5/5A/rescue-L2E/5-3.tif]
