# Supplementary figures and images for: WTAP tetramer ensures m6A writer assembly and faithful mitosis (part 3 of 4)
Source: EMBO Rep. 2026 Jun 2;27(13):3842–62. doi: 10.1038/s44319-026-00815-3 (PMC13354555; doi:10.1038/s44319-026-00815-3)

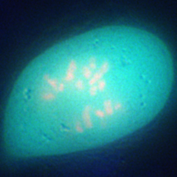

Supplement: Supplementary file 10 — Source data Fig. 5 [file 44319_2026_815_MOESM10_ESM.zip › Figure 5/5A/rescue-L2E/5-4.tif]

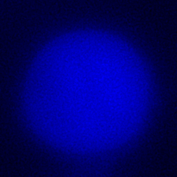

Supplement: Supplementary file 10 — Source data Fig. 5 [file 44319_2026_815_MOESM10_ESM.zip › Figure 5/5A/rescue-L2E/70-1.tif]

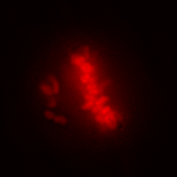

Supplement: Supplementary file 10 — Source data Fig. 5 [file 44319_2026_815_MOESM10_ESM.zip › Figure 5/5A/rescue-L2E/70-2.tif]

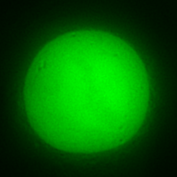

Supplement: Supplementary file 10 — Source data Fig. 5 [file 44319_2026_815_MOESM10_ESM.zip › Figure 5/5A/rescue-L2E/70-3.tif]

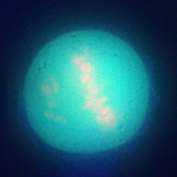

Supplement: Supplementary file 10 — Source data Fig. 5 [file 44319_2026_815_MOESM10_ESM.zip › Figure 5/5A/rescue-L2E/70-4.tif]

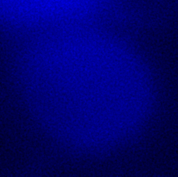

Supplement: Supplementary file 10 — Source data Fig. 5 [file 44319_2026_815_MOESM10_ESM.zip › Figure 5/5A/rescue-WT/0-1.tif]

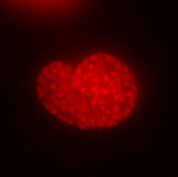

Supplement: Supplementary file 10 — Source data Fig. 5 [file 44319_2026_815_MOESM10_ESM.zip › Figure 5/5A/rescue-WT/0-2.tif]

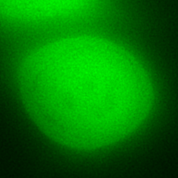

Supplement: Supplementary file 10 — Source data Fig. 5 [file 44319_2026_815_MOESM10_ESM.zip › Figure 5/5A/rescue-WT/0-3.tif]

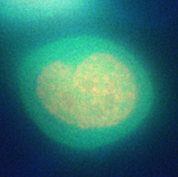

Supplement: Supplementary file 10 — Source data Fig. 5 [file 44319_2026_815_MOESM10_ESM.zip › Figure 5/5A/rescue-WT/0-4.tif]

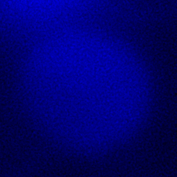

Supplement: Supplementary file 10 — Source data Fig. 5 [file 44319_2026_815_MOESM10_ESM.zip › Figure 5/5A/rescue-WT/10-1.tif]

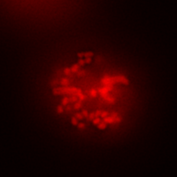

Supplement: Supplementary file 10 — Source data Fig. 5 [file 44319_2026_815_MOESM10_ESM.zip › Figure 5/5A/rescue-WT/10-2.tif]

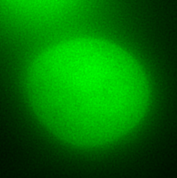

Supplement: Supplementary file 10 — Source data Fig. 5 [file 44319_2026_815_MOESM10_ESM.zip › Figure 5/5A/rescue-WT/10-3.tif]

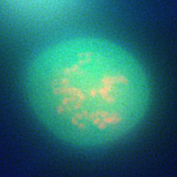

Supplement: Supplementary file 10 — Source data Fig. 5 [file 44319_2026_815_MOESM10_ESM.zip › Figure 5/5A/rescue-WT/10-4.tif]

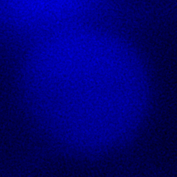

Supplement: Supplementary file 10 — Source data Fig. 5 [file 44319_2026_815_MOESM10_ESM.zip › Figure 5/5A/rescue-WT/15-1.tif]

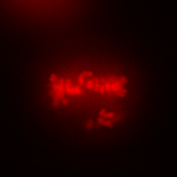

Supplement: Supplementary file 10 — Source data Fig. 5 [file 44319_2026_815_MOESM10_ESM.zip › Figure 5/5A/rescue-WT/15-2.tif]

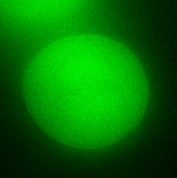

Supplement: Supplementary file 10 — Source data Fig. 5 [file 44319_2026_815_MOESM10_ESM.zip › Figure 5/5A/rescue-WT/15-3.tif]

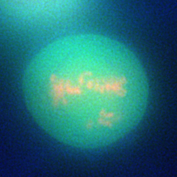

Supplement: Supplementary file 10 — Source data Fig. 5 [file 44319_2026_815_MOESM10_ESM.zip › Figure 5/5A/rescue-WT/15-4.tif]

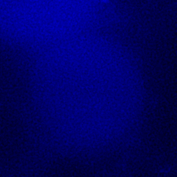

Supplement: Supplementary file 10 — Source data Fig. 5 [file 44319_2026_815_MOESM10_ESM.zip › Figure 5/5A/rescue-WT/25-1.tif]

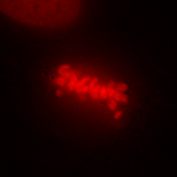

Supplement: Supplementary file 10 — Source data Fig. 5 [file 44319_2026_815_MOESM10_ESM.zip › Figure 5/5A/rescue-WT/25-2.tif]

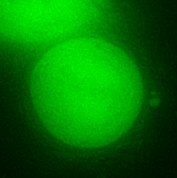

Supplement: Supplementary file 10 — Source data Fig. 5 [file 44319_2026_815_MOESM10_ESM.zip › Figure 5/5A/rescue-WT/25-3.tif]

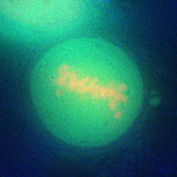

Supplement: Supplementary file 10 — Source data Fig. 5 [file 44319_2026_815_MOESM10_ESM.zip › Figure 5/5A/rescue-WT/25-4.tif]

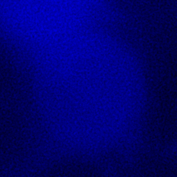

Supplement: Supplementary file 10 — Source data Fig. 5 [file 44319_2026_815_MOESM10_ESM.zip › Figure 5/5A/rescue-WT/35-1.tif]

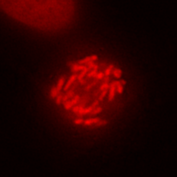

Supplement: Supplementary file 10 — Source data Fig. 5 [file 44319_2026_815_MOESM10_ESM.zip › Figure 5/5A/rescue-WT/35-2.tif]

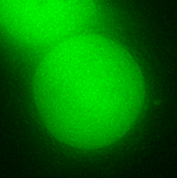

Supplement: Supplementary file 10 — Source data Fig. 5 [file 44319_2026_815_MOESM10_ESM.zip › Figure 5/5A/rescue-WT/35-3.tif]

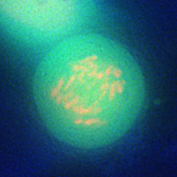

Supplement: Supplementary file 10 — Source data Fig. 5 [file 44319_2026_815_MOESM10_ESM.zip › Figure 5/5A/rescue-WT/35-4.tif]

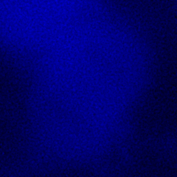

Supplement: Supplementary file 10 — Source data Fig. 5 [file 44319_2026_815_MOESM10_ESM.zip › Figure 5/5A/rescue-WT/40-1.tif]

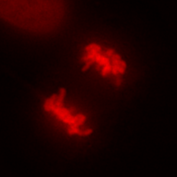

Supplement: Supplementary file 10 — Source data Fig. 5 [file 44319_2026_815_MOESM10_ESM.zip › Figure 5/5A/rescue-WT/40-2.tif]

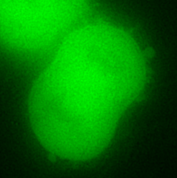

Supplement: Supplementary file 10 — Source data Fig. 5 [file 44319_2026_815_MOESM10_ESM.zip › Figure 5/5A/rescue-WT/40-3.tif]

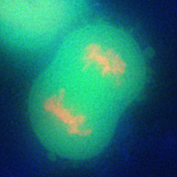

Supplement: Supplementary file 10 — Source data Fig. 5 [file 44319_2026_815_MOESM10_ESM.zip › Figure 5/5A/rescue-WT/40-4.tif]

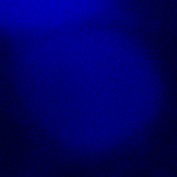

Supplement: Supplementary file 10 — Source data Fig. 5 [file 44319_2026_815_MOESM10_ESM.zip › Figure 5/5A/rescue-WT/5-1.tif]

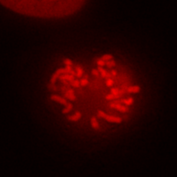

Supplement: Supplementary file 10 — Source data Fig. 5 [file 44319_2026_815_MOESM10_ESM.zip › Figure 5/5A/rescue-WT/5-2.tif]

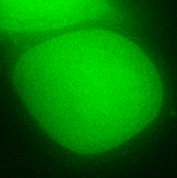

Supplement: Supplementary file 10 — Source data Fig. 5 [file 44319_2026_815_MOESM10_ESM.zip › Figure 5/5A/rescue-WT/5-3.tif]

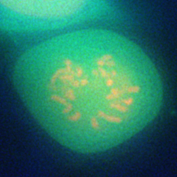

Supplement: Supplementary file 10 — Source data Fig. 5 [file 44319_2026_815_MOESM10_ESM.zip › Figure 5/5A/rescue-WT/5-4.tif]

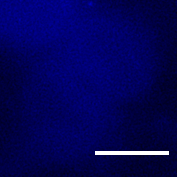

Supplement: Supplementary file 10 — Source data Fig. 5 [file 44319_2026_815_MOESM10_ESM.zip › Figure 5/5A/rescue-WT/50-1 bar.tif]

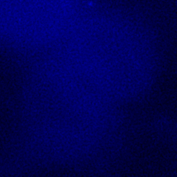

Supplement: Supplementary file 10 — Source data Fig. 5 [file 44319_2026_815_MOESM10_ESM.zip › Figure 5/5A/rescue-WT/50-1.tif]

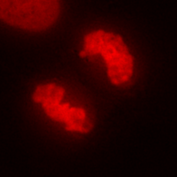

Supplement: Supplementary file 10 — Source data Fig. 5 [file 44319_2026_815_MOESM10_ESM.zip › Figure 5/5A/rescue-WT/50-2.tif]

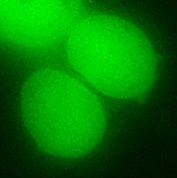

Supplement: Supplementary file 10 — Source data Fig. 5 [file 44319_2026_815_MOESM10_ESM.zip › Figure 5/5A/rescue-WT/50-3.tif]

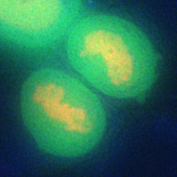

Supplement: Supplementary file 10 — Source data Fig. 5 [file 44319_2026_815_MOESM10_ESM.zip › Figure 5/5A/rescue-WT/50-4.tif]

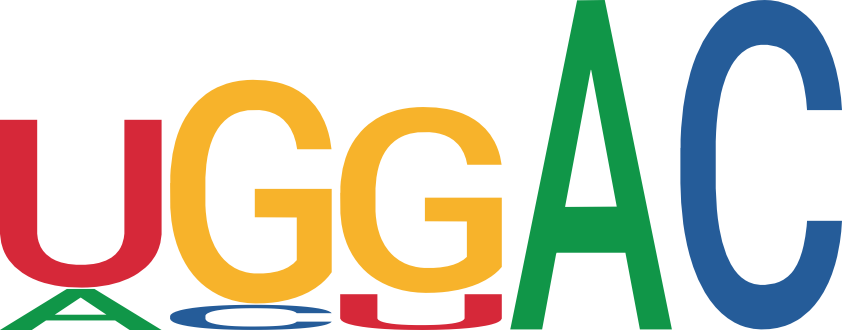

Supplement: Supplementary file 11 — Source data Fig. 6 [file 44319_2026_815_MOESM11_ESM.zip › Figure 6/6A/shNC.tif]

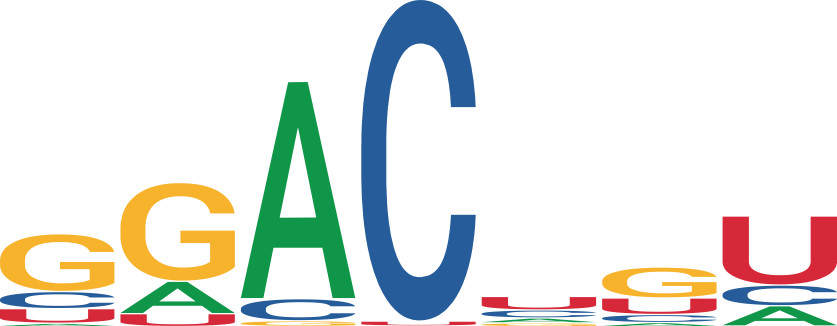

Supplement: Supplementary file 11 — Source data Fig. 6 [file 44319_2026_815_MOESM11_ESM.zip › Figure 6/6A/shWTAP.tif]

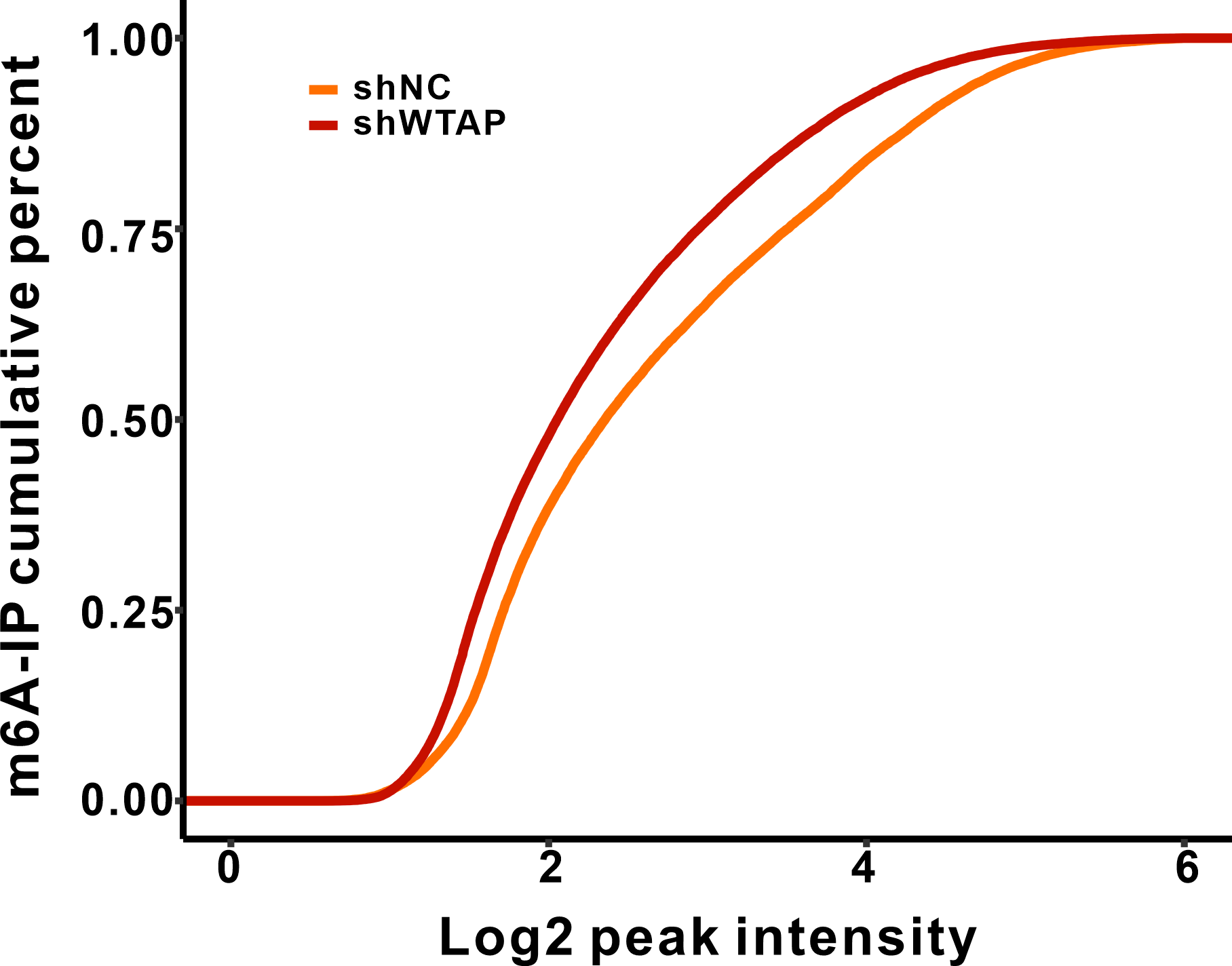

Supplement: Supplementary file 11 — Source data Fig. 6 [file 44319_2026_815_MOESM11_ESM.zip › Figure 6/6B/CDF plot.tif]

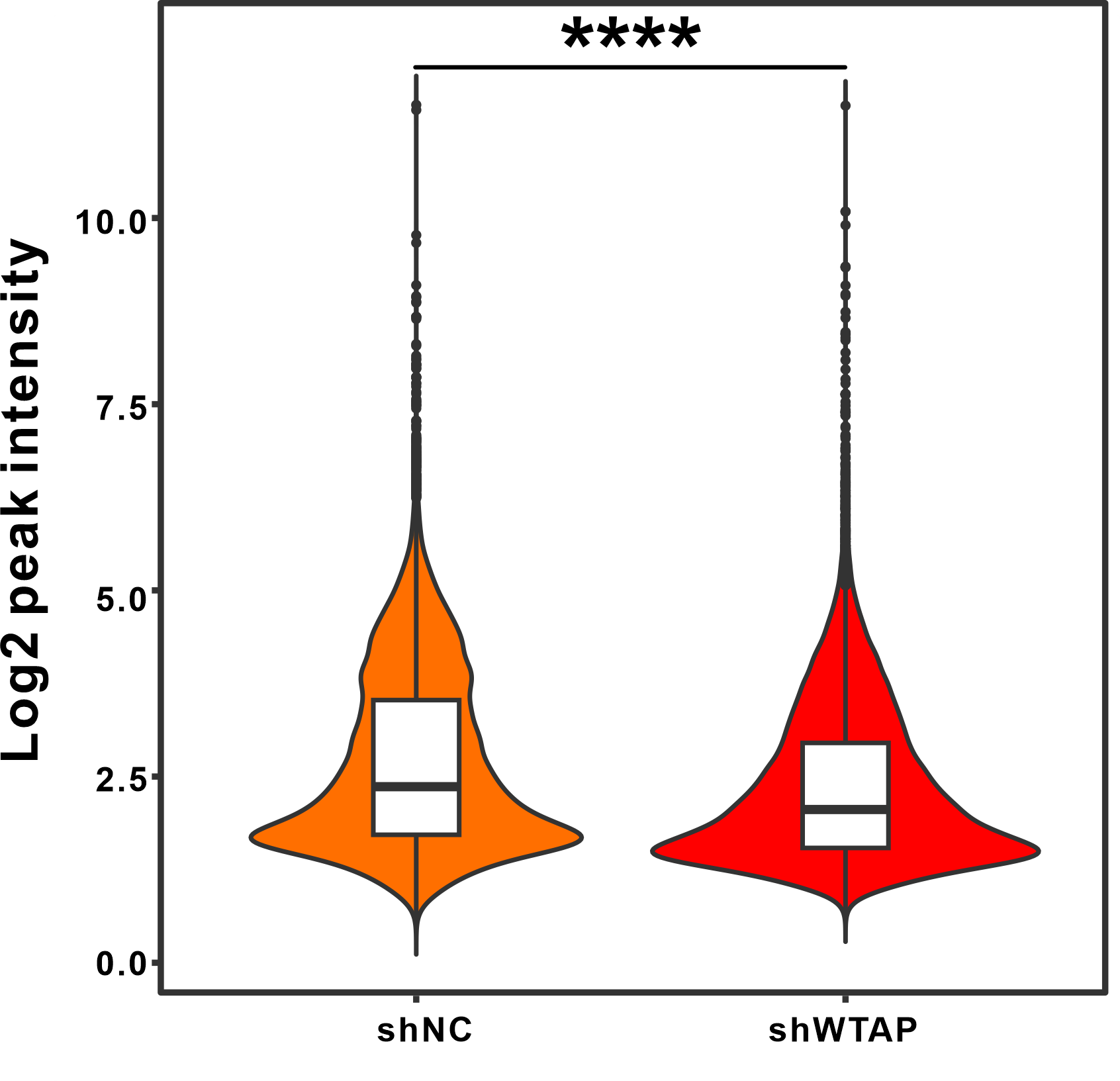

Supplement: Supplementary file 11 — Source data Fig. 6 [file 44319_2026_815_MOESM11_ESM.zip › Figure 6/6B/violin polt.tif]

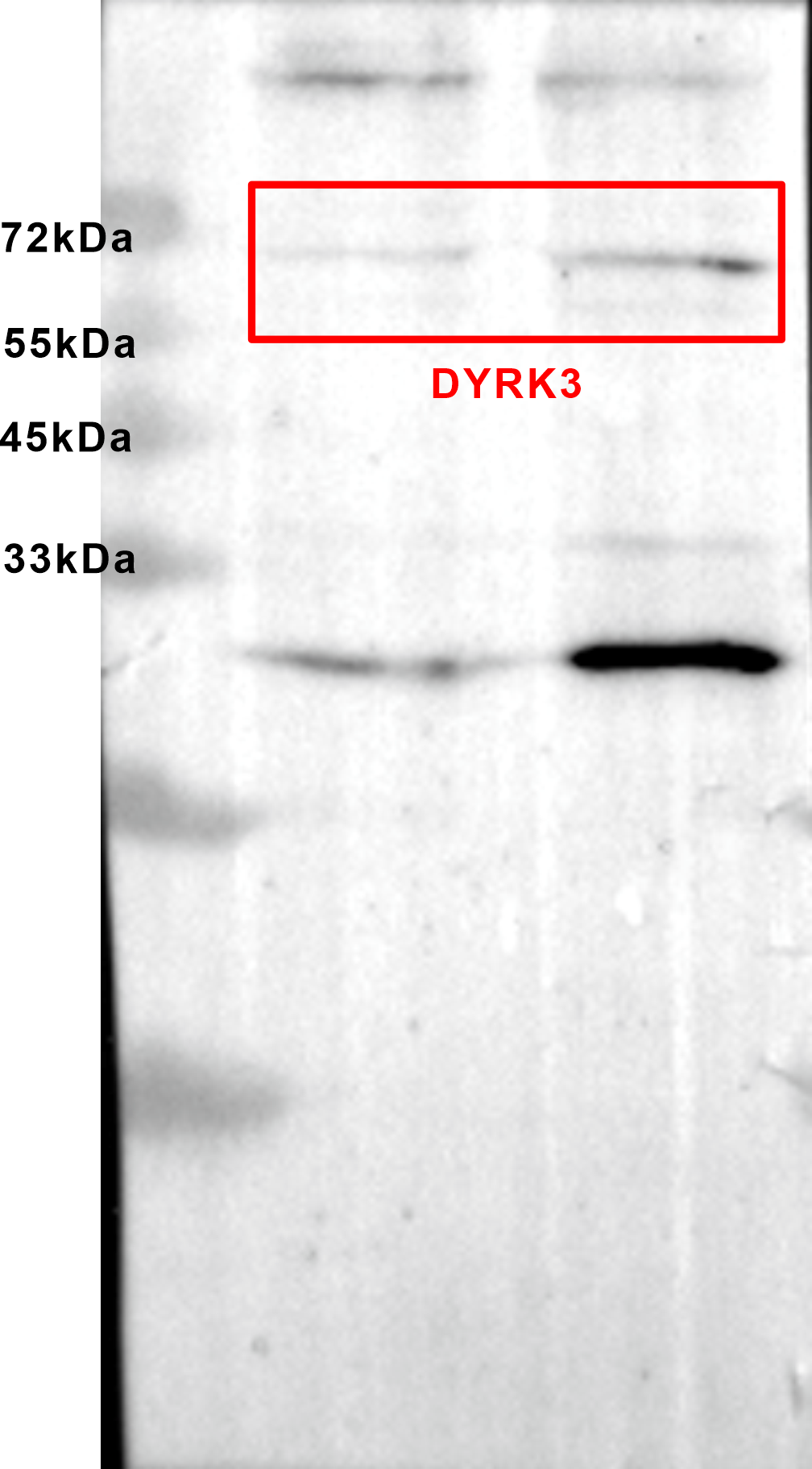

Supplement: Supplementary file 11 — Source data Fig. 6 [file 44319_2026_815_MOESM11_ESM.zip › Figure 6/6H/Western blot-DYRK3.tif]

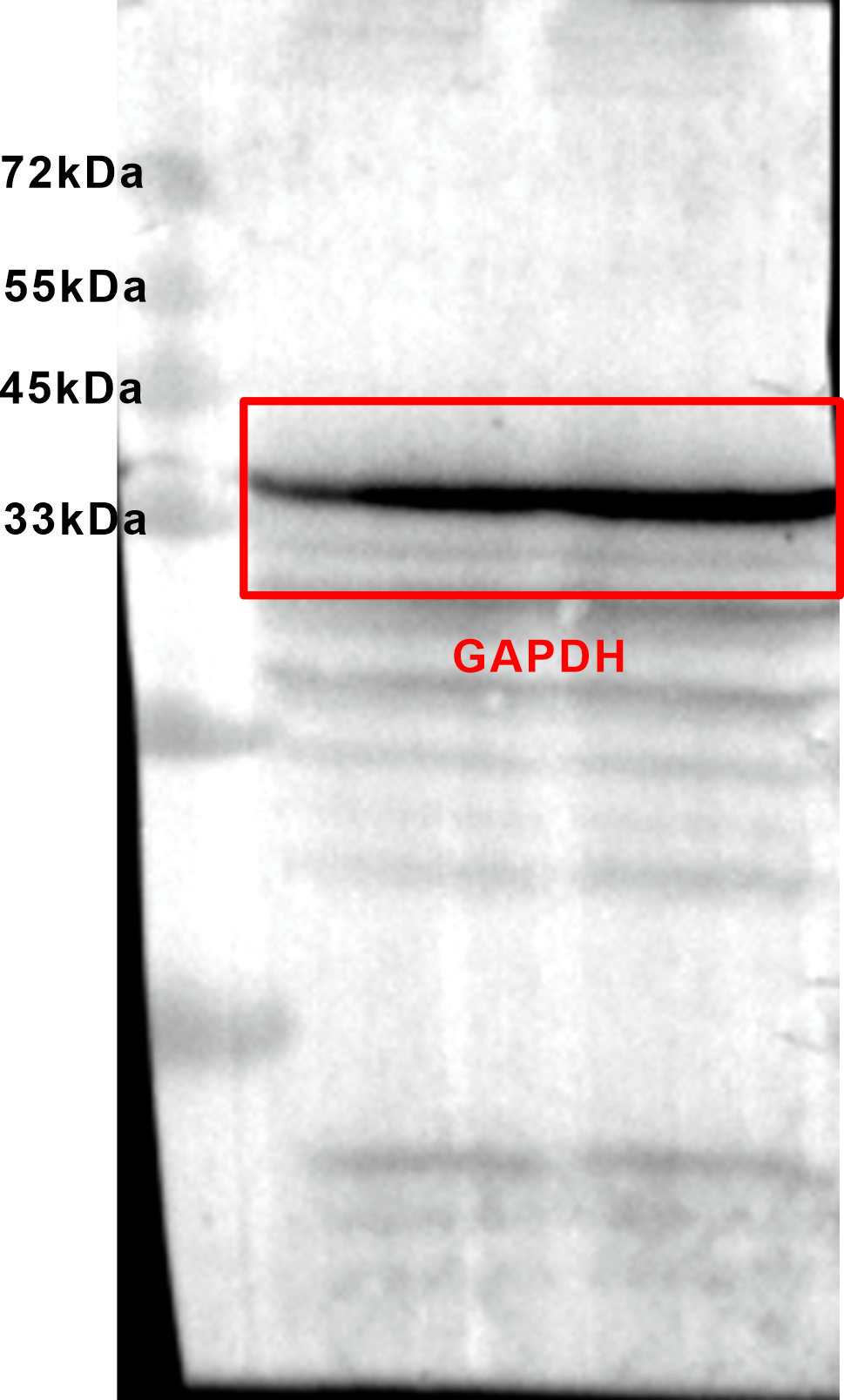

Supplement: Supplementary file 11 — Source data Fig. 6 [file 44319_2026_815_MOESM11_ESM.zip › Figure 6/6H/Western blot-GAPDH.tif]

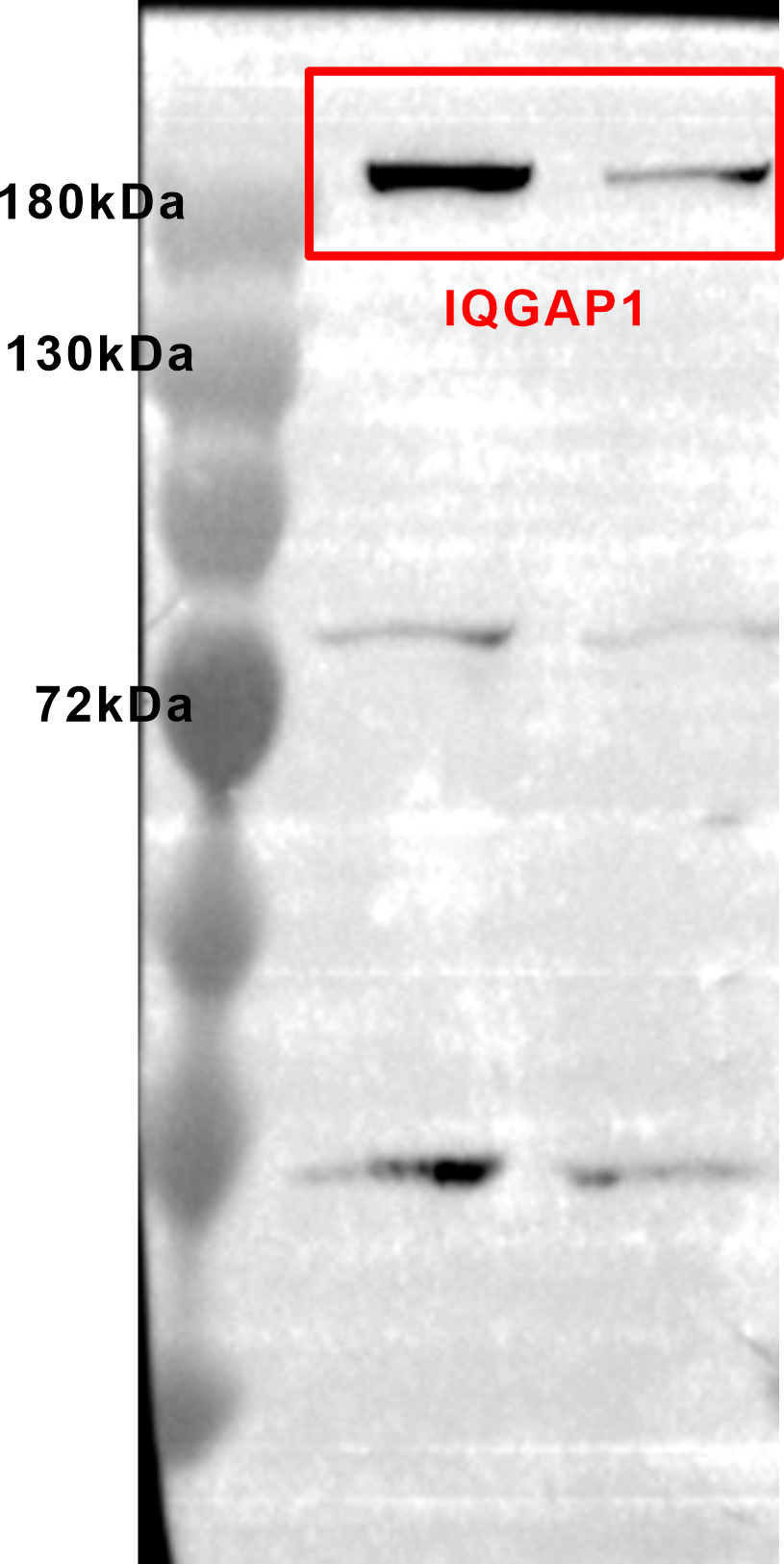

Supplement: Supplementary file 11 — Source data Fig. 6 [file 44319_2026_815_MOESM11_ESM.zip › Figure 6/6H/Western blot-IQGAP1.tif]

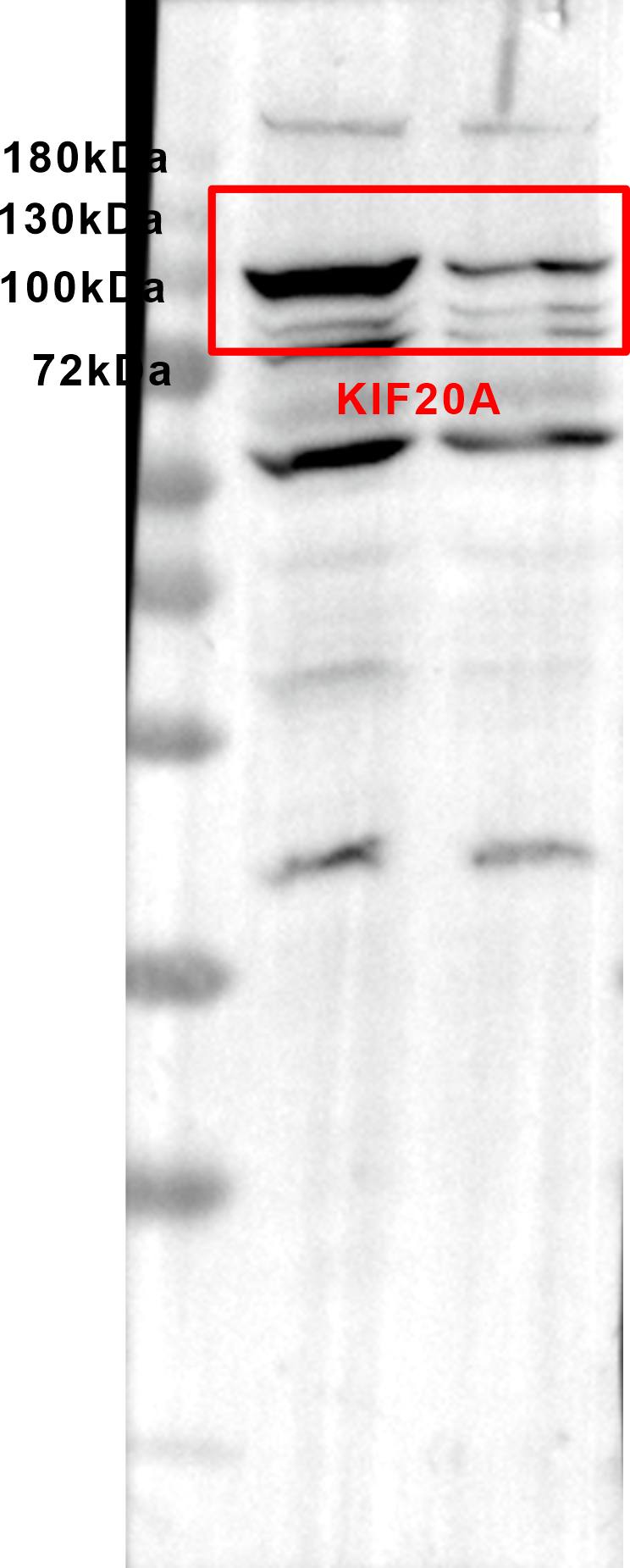

Supplement: Supplementary file 11 — Source data Fig. 6 [file 44319_2026_815_MOESM11_ESM.zip › Figure 6/6H/Western blot-KIF20A.tif]

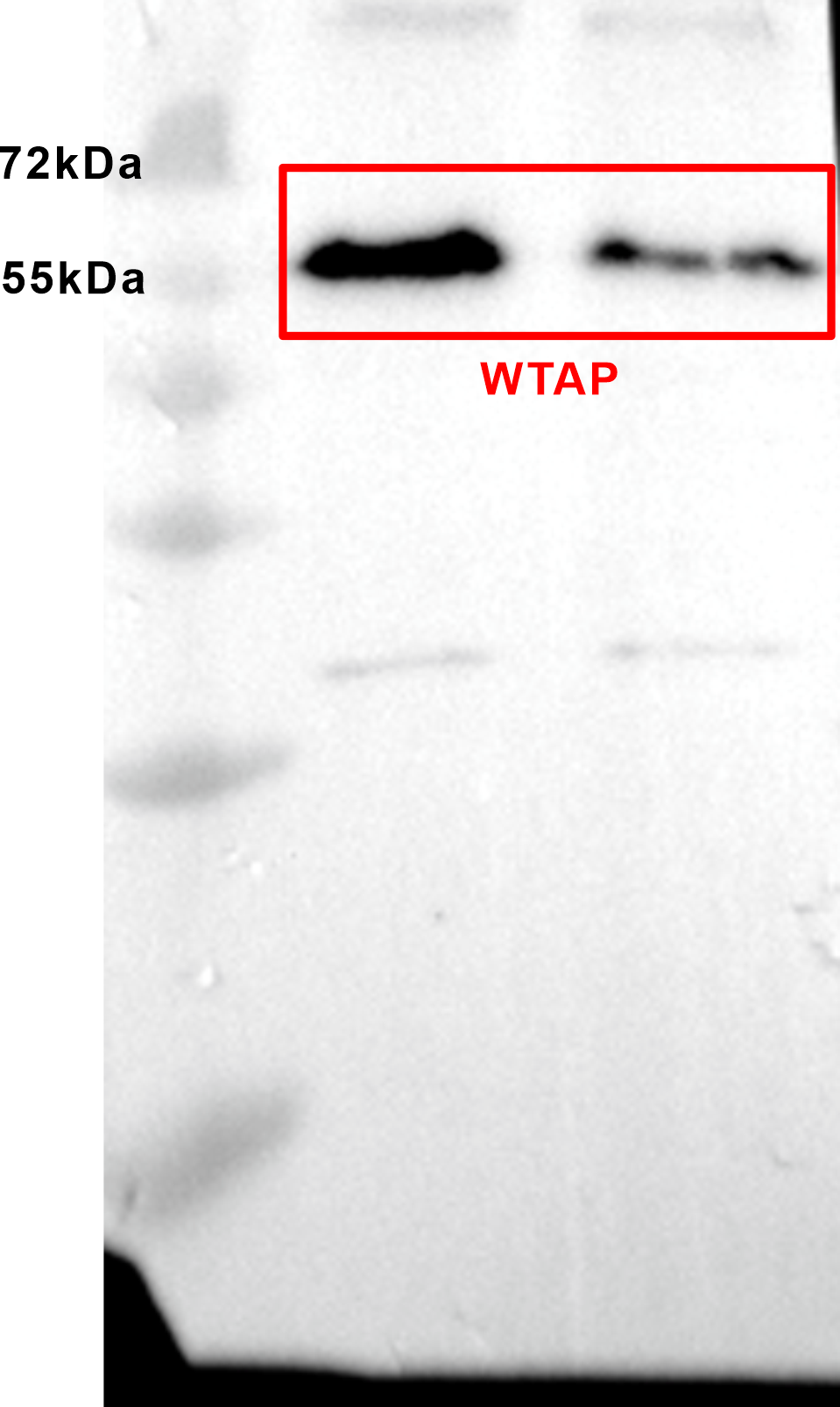

Supplement: Supplementary file 11 — Source data Fig. 6 [file 44319_2026_815_MOESM11_ESM.zip › Figure 6/6H/Western blot-WTAP.tif]

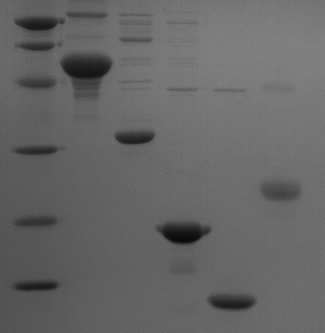

Supplement: Supplementary file 12 — Figure EV1 Source Data [file 44319_2026_815_MOESM12_ESM.zip › Figure EV1/1A/WTAP.tif]

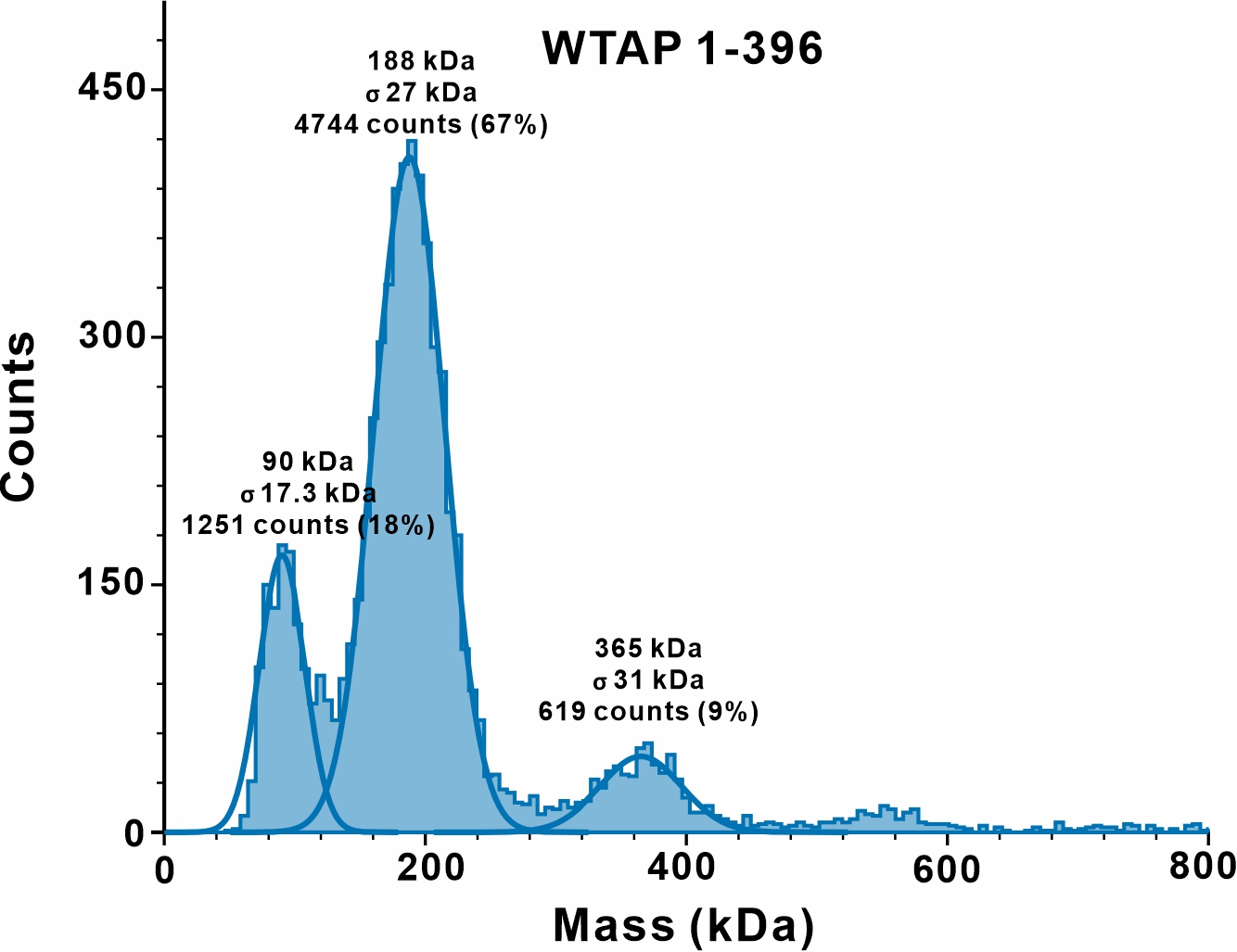

Supplement: Supplementary file 12 — Figure EV1 Source Data [file 44319_2026_815_MOESM12_ESM.zip › Figure EV1/1B/WTAP FL.jpg]

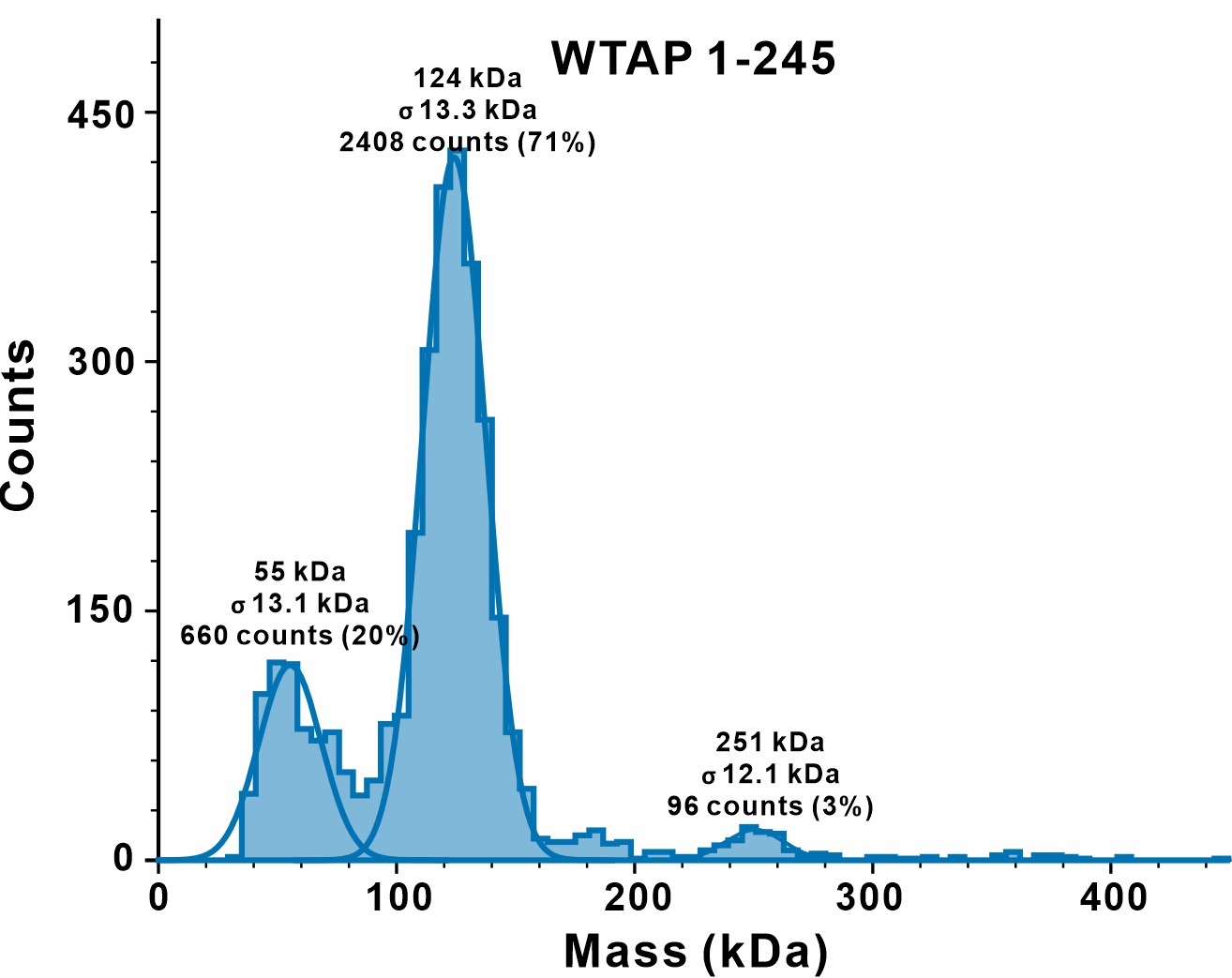

Supplement: Supplementary file 12 — Figure EV1 Source Data [file 44319_2026_815_MOESM12_ESM.zip › Figure EV1/1C/WTAP F1.jpg]

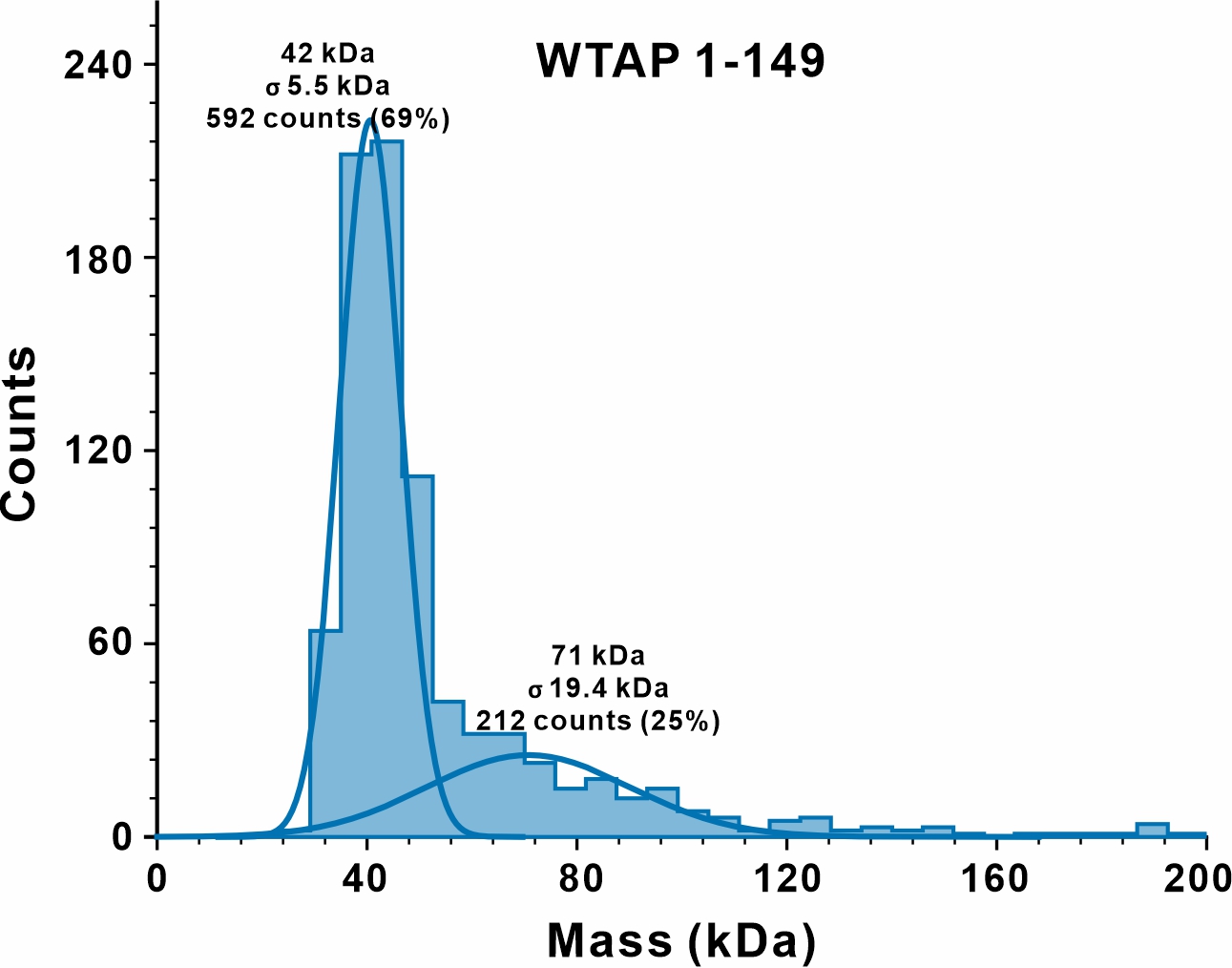

Supplement: Supplementary file 12 — Figure EV1 Source Data [file 44319_2026_815_MOESM12_ESM.zip › Figure EV1/1D/WTAP F2.jpg]

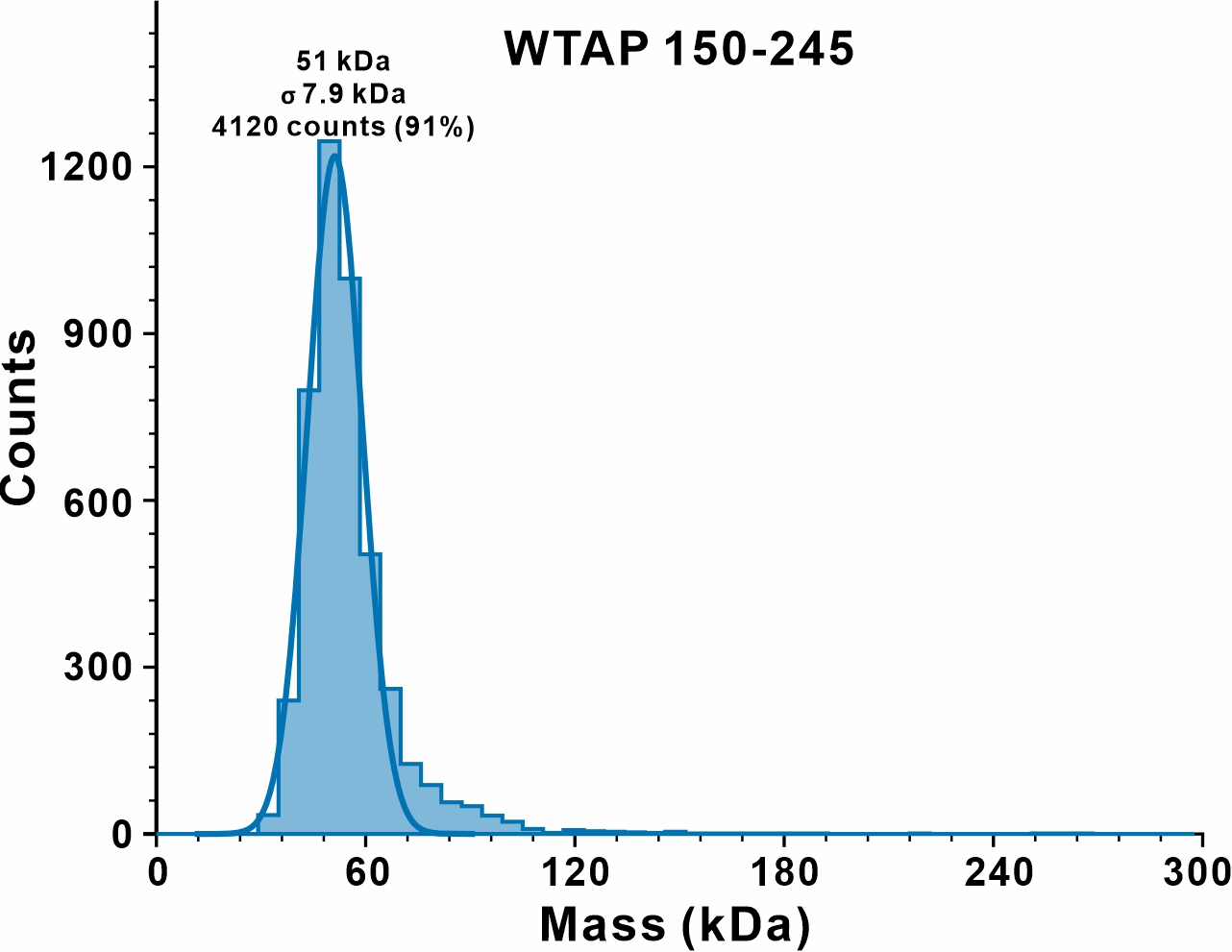

Supplement: Supplementary file 12 — Figure EV1 Source Data [file 44319_2026_815_MOESM12_ESM.zip › Figure EV1/1E/WTAP F3.jpg]

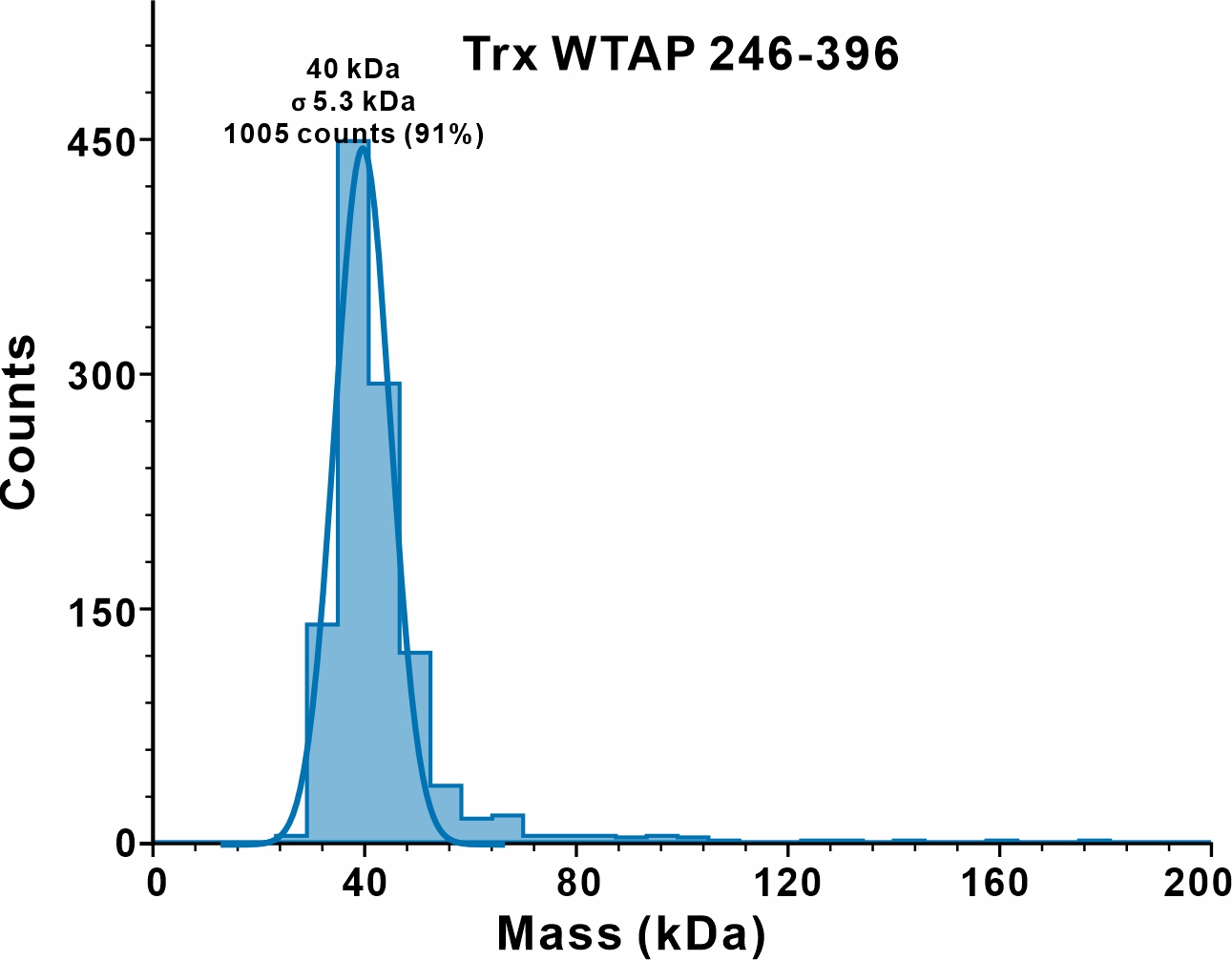

Supplement: Supplementary file 12 — Figure EV1 Source Data [file 44319_2026_815_MOESM12_ESM.zip › Figure EV1/1F/WTAP F4.jpg]

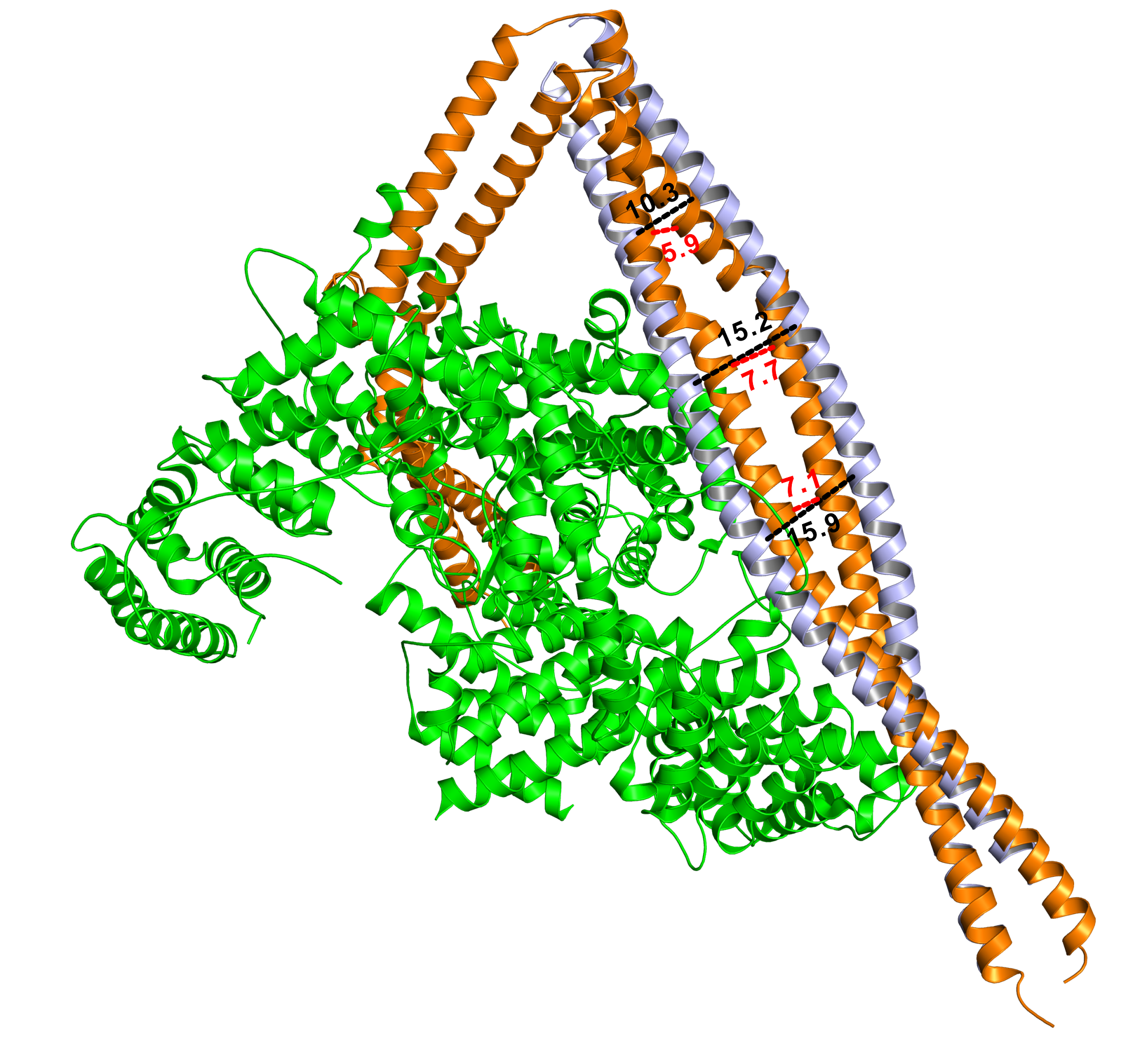

Supplement: Supplementary file 13 — Figure EV2 Source Data [file 44319_2026_815_MOESM13_ESM.zip › Figure EV2/2A/Structure.tif]

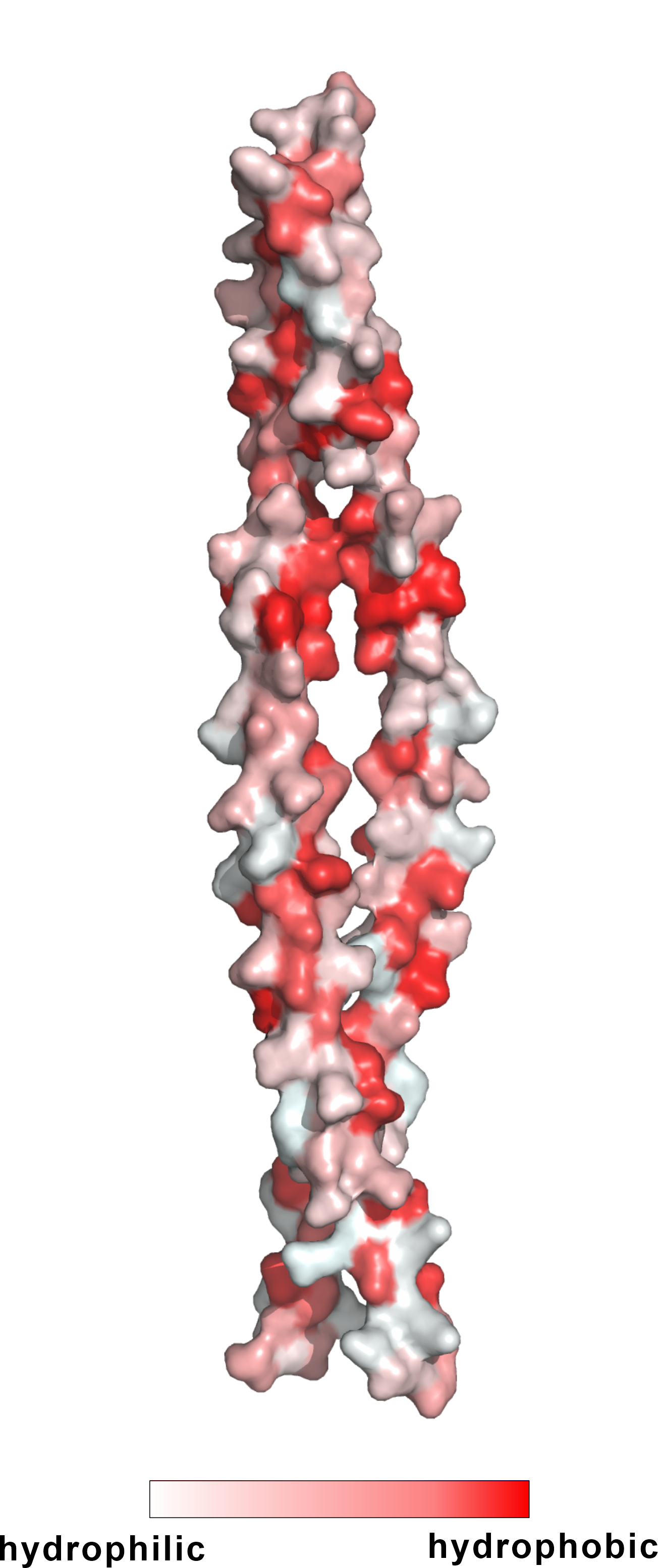

Supplement: Supplementary file 13 — Figure EV2 Source Data [file 44319_2026_815_MOESM13_ESM.zip › Figure EV2/2B/1.tif]

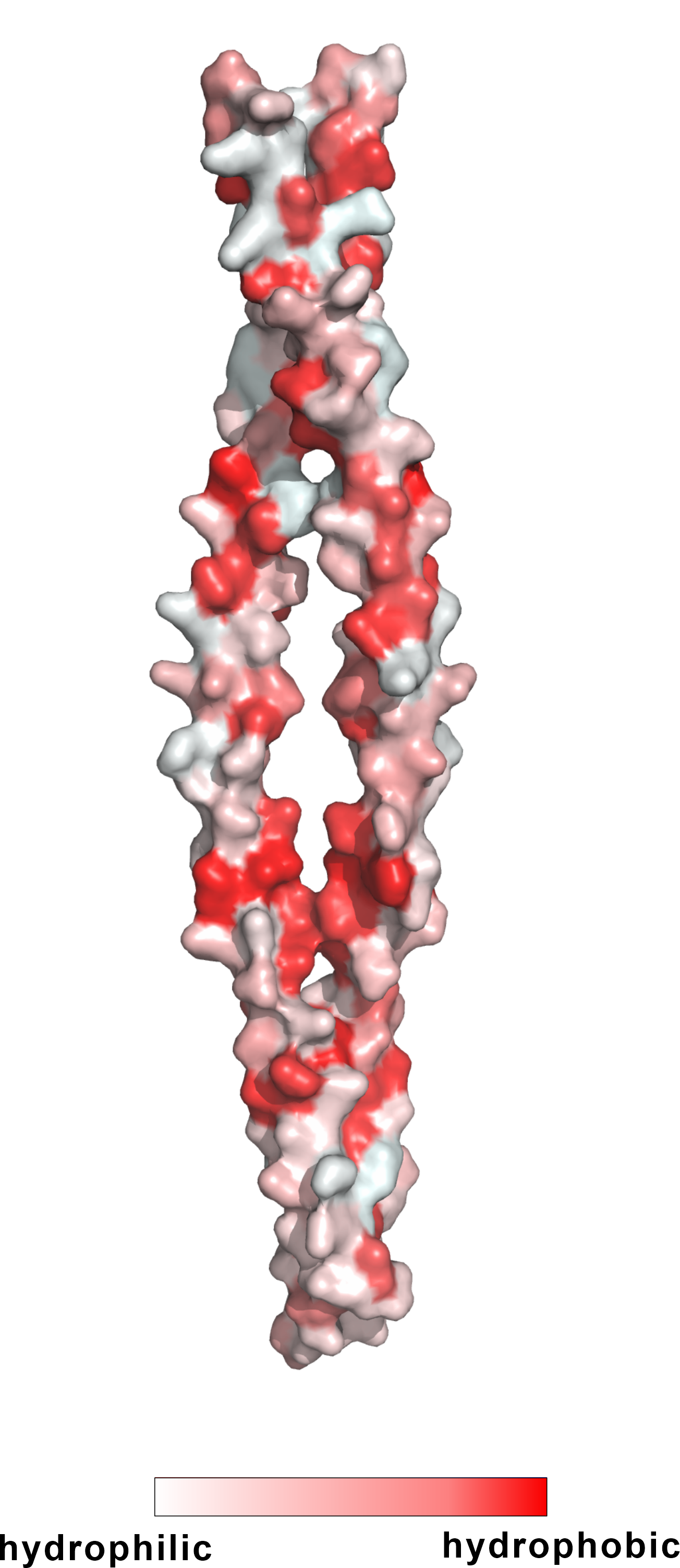

Supplement: Supplementary file 13 — Figure EV2 Source Data [file 44319_2026_815_MOESM13_ESM.zip › Figure EV2/2B/2.tif]

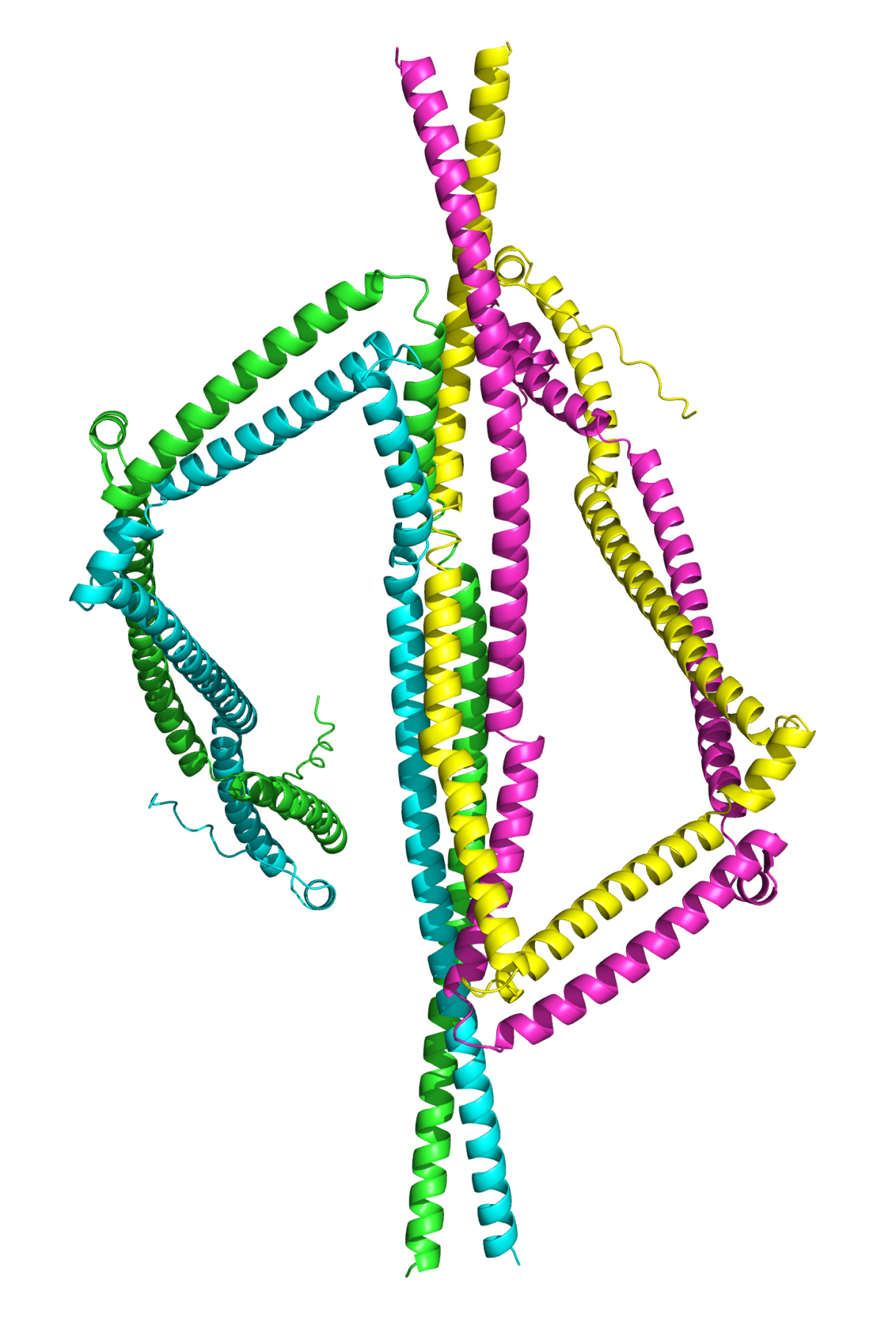

Supplement: Supplementary file 13 — Figure EV2 Source Data [file 44319_2026_815_MOESM13_ESM.zip › Figure EV2/2C/structure model.tif]

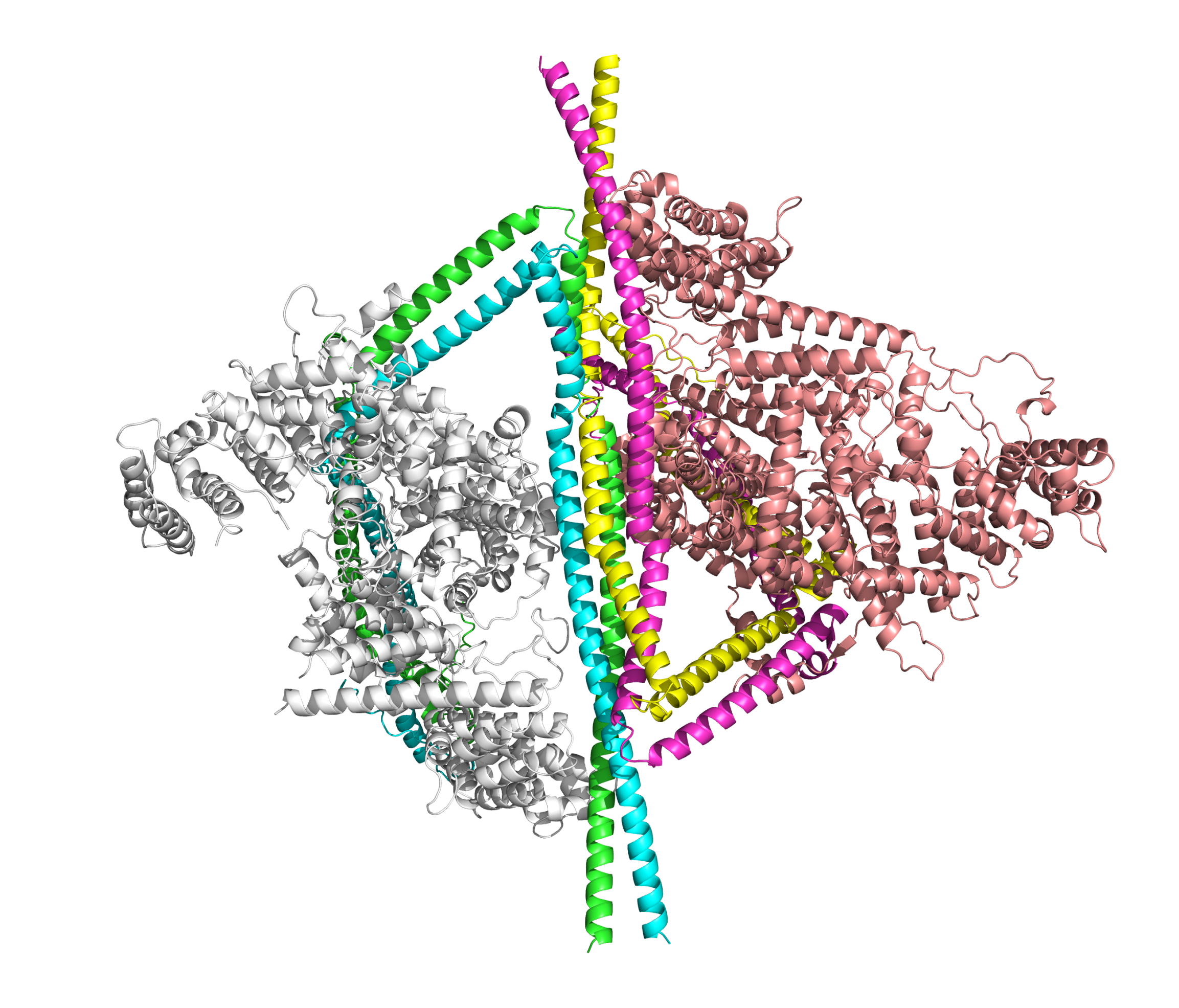

Supplement: Supplementary file 13 — Figure EV2 Source Data [file 44319_2026_815_MOESM13_ESM.zip › Figure EV2/2E/Structure model.tif]

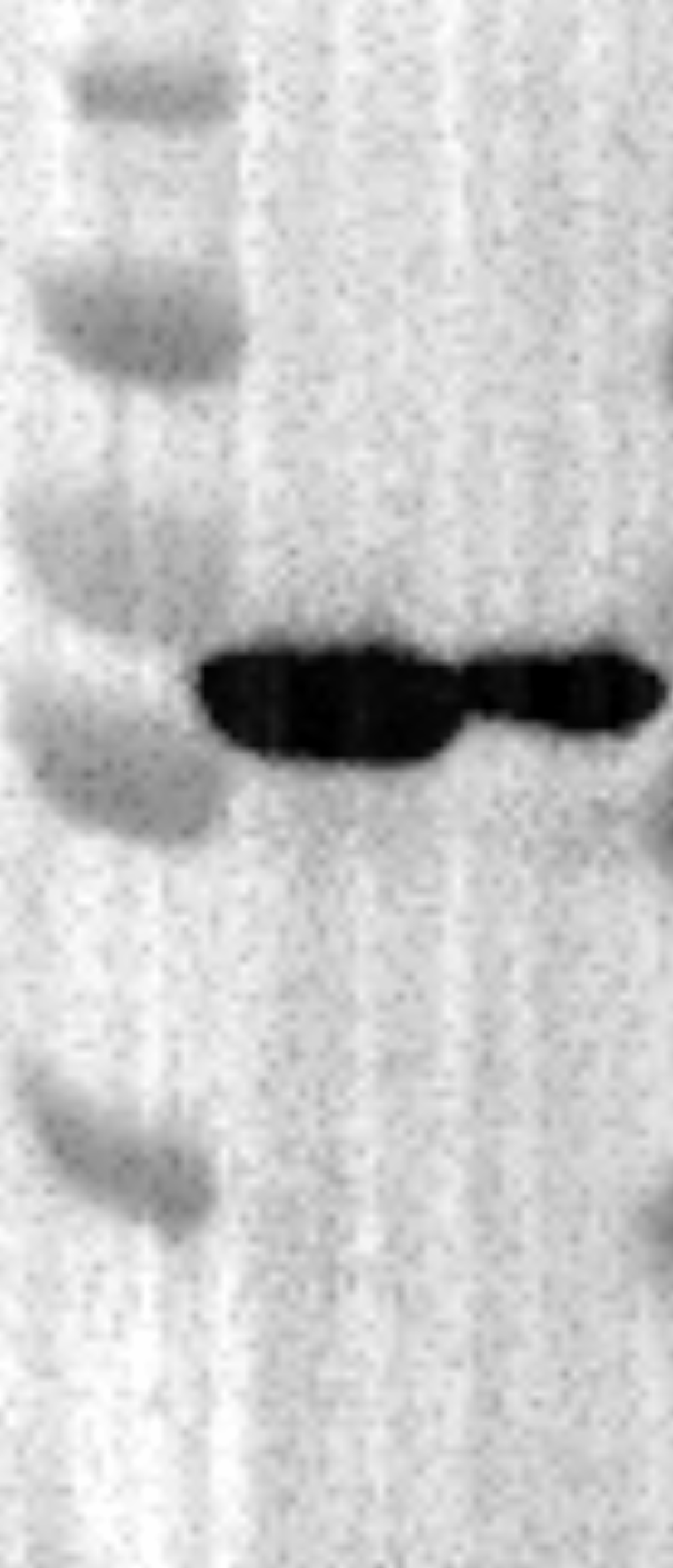

Supplement: Supplementary file 13 — Figure EV2 Source Data [file 44319_2026_815_MOESM13_ESM.zip › Figure EV2/2J/western blot anti-WTAP.tif]

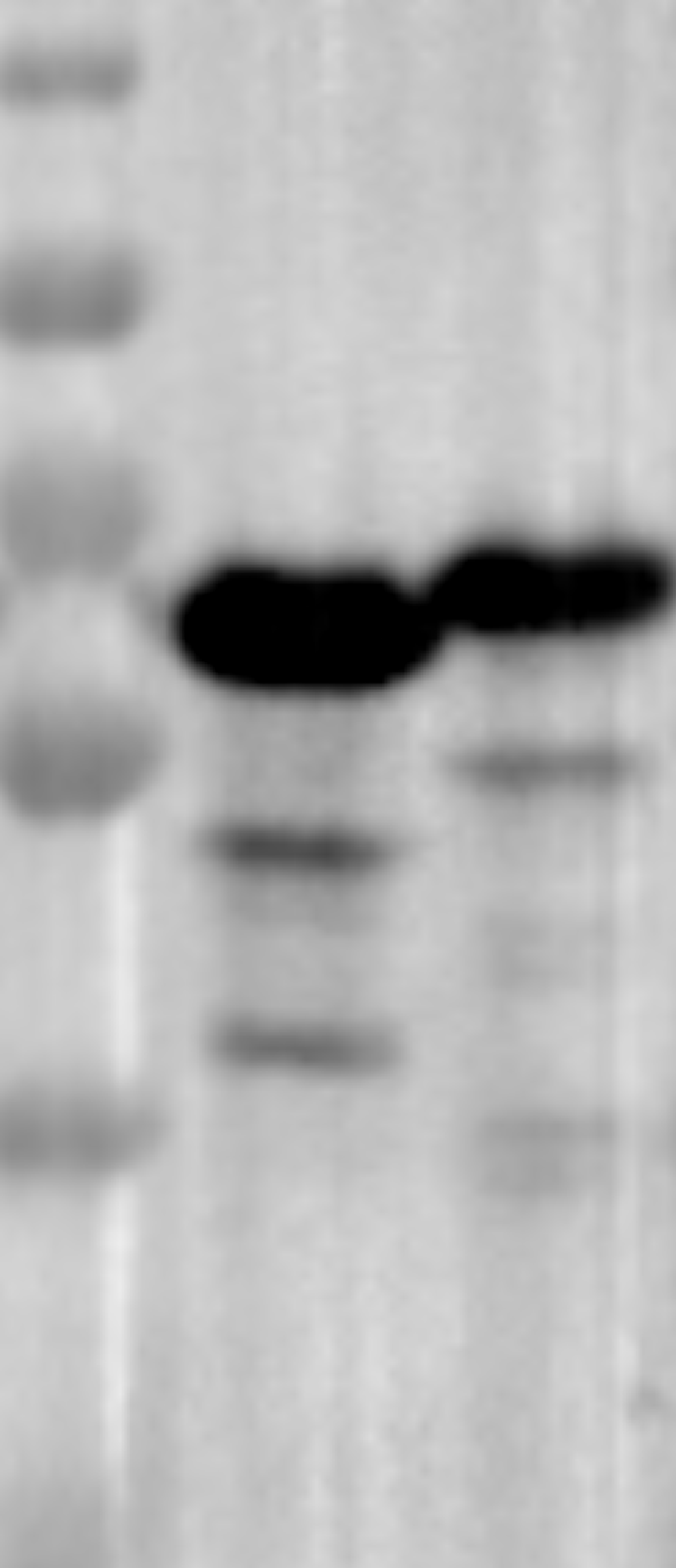

Supplement: Supplementary file 13 — Figure EV2 Source Data [file 44319_2026_815_MOESM13_ESM.zip › Figure EV2/2K/Western blot anti-flag.tif]

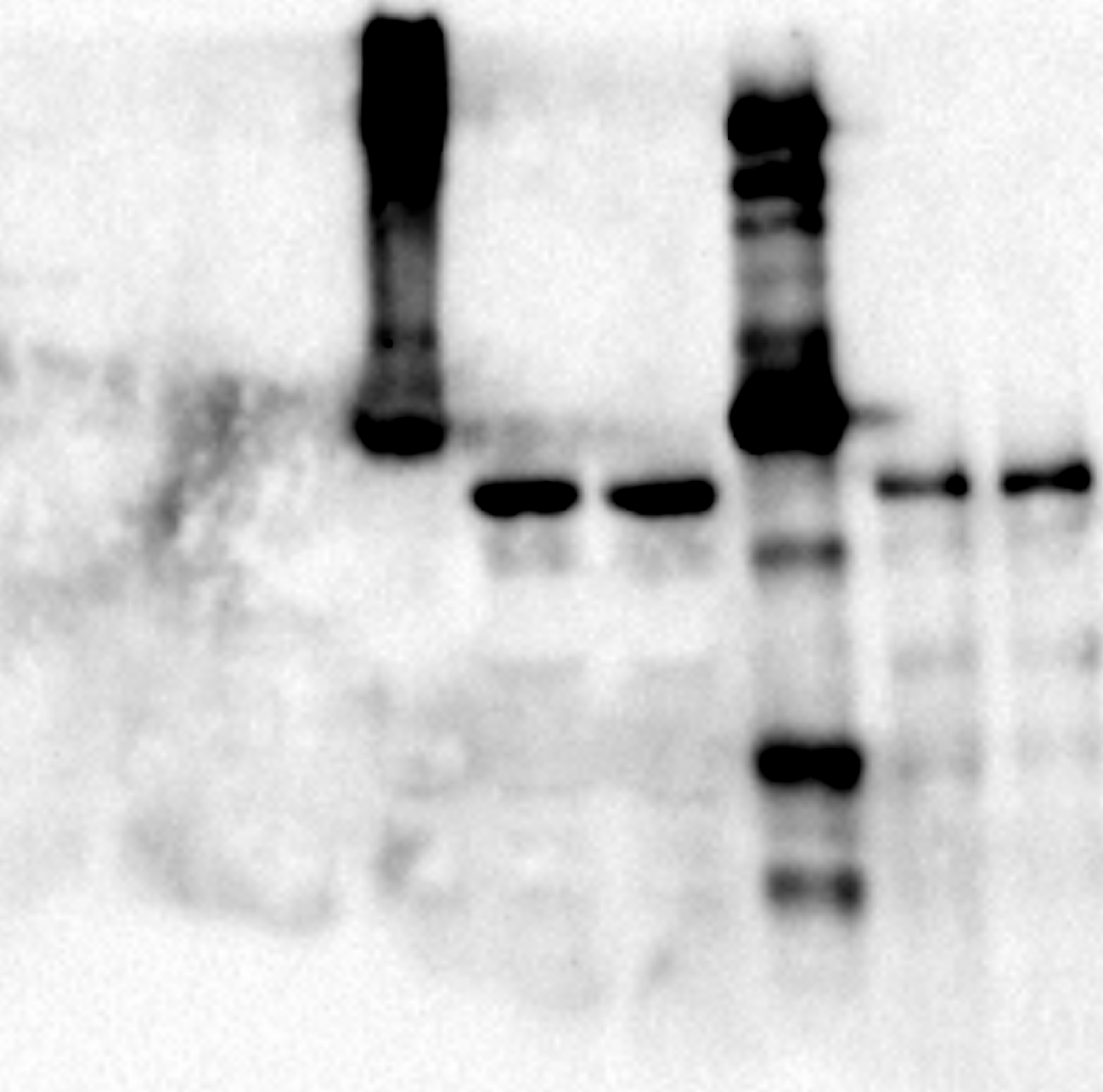

Supplement: Supplementary file 14 — Figure EV3 Source Data [file 44319_2026_815_MOESM14_ESM.zip › Figure EV3/3C/anti Flag.tif]

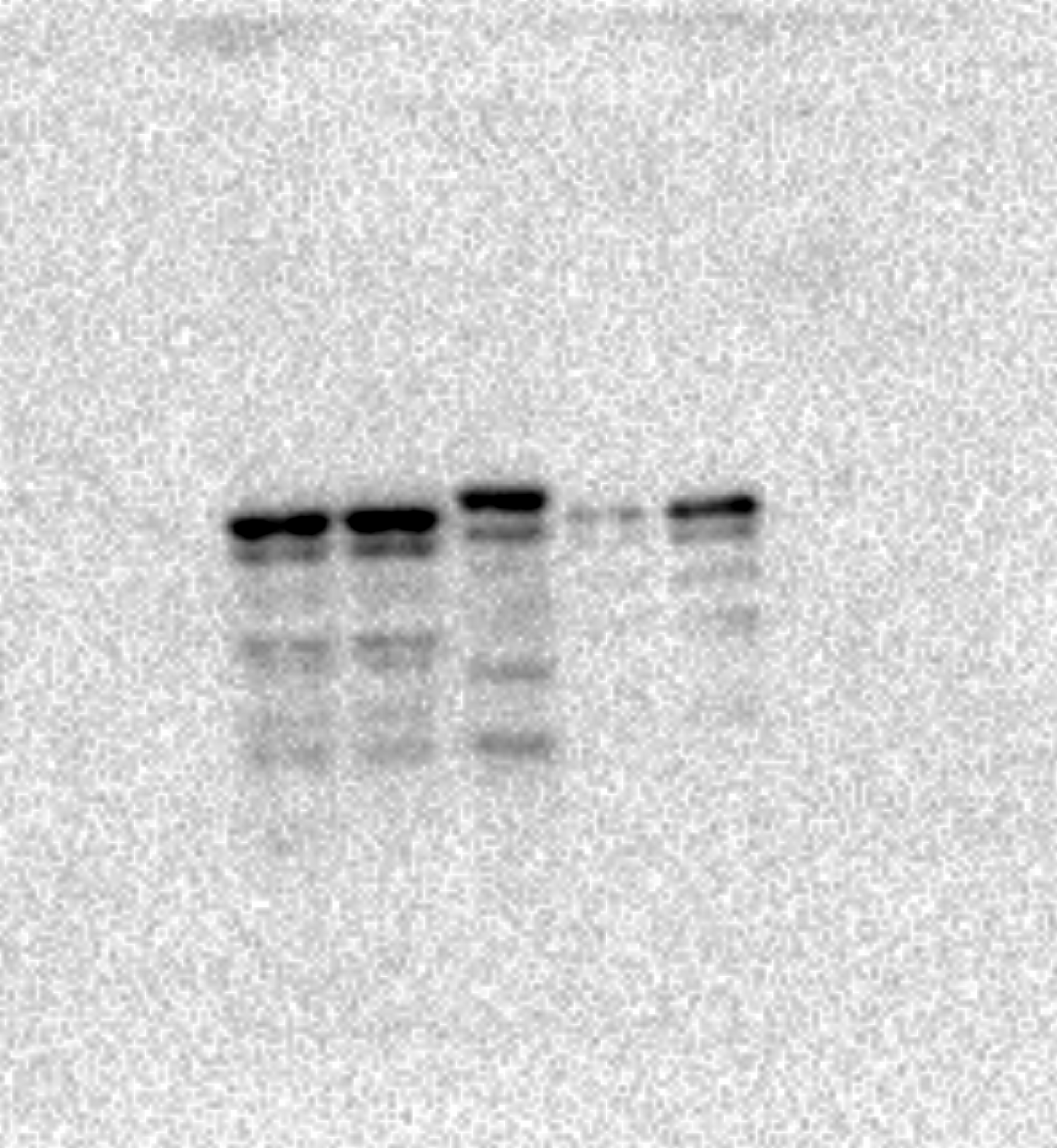

Supplement: Supplementary file 14 — Figure EV3 Source Data [file 44319_2026_815_MOESM14_ESM.zip › Figure EV3/3C/anti GFP.tif]

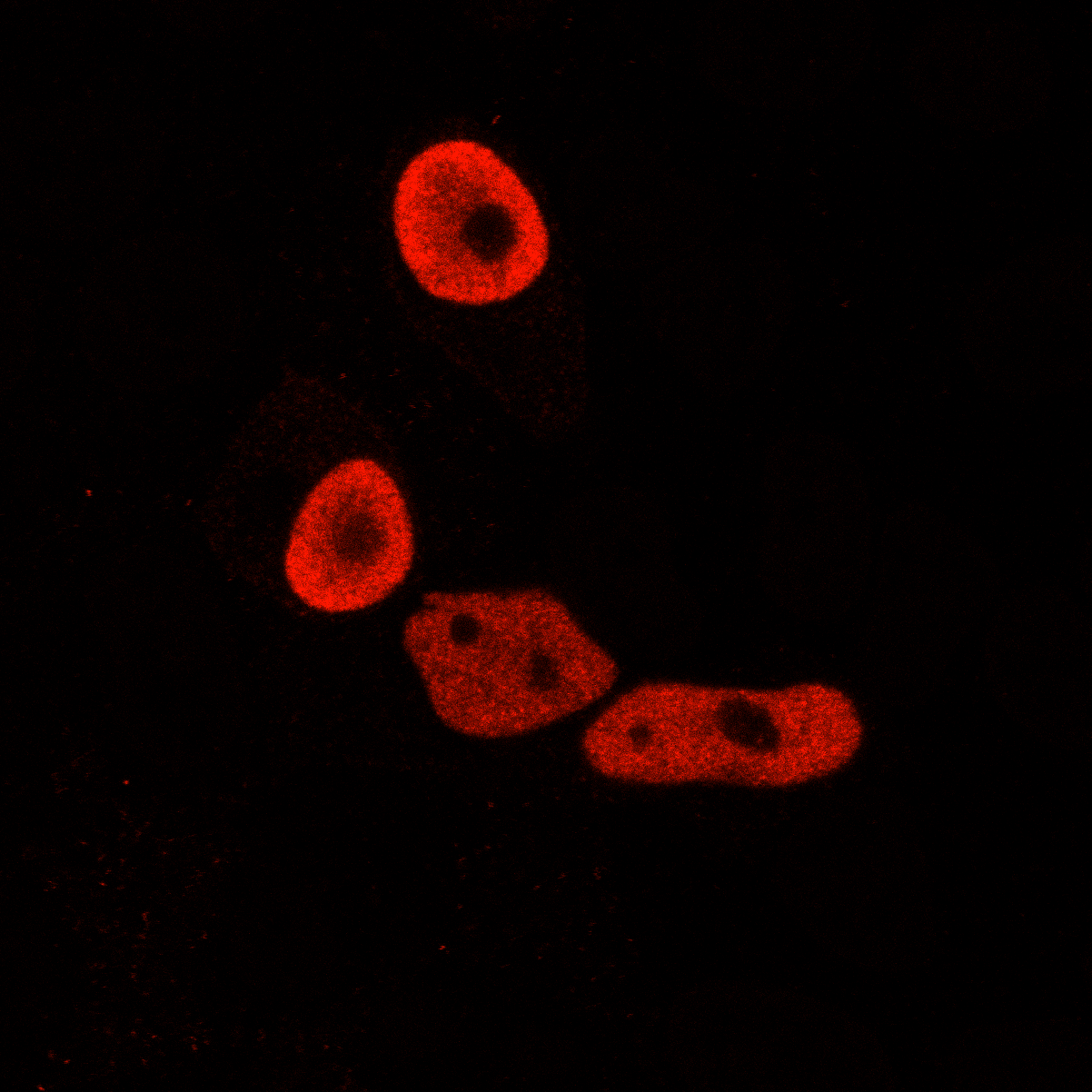

Supplement: Supplementary file 14 — Figure EV3 Source Data [file 44319_2026_815_MOESM14_ESM.zip › Figure EV3/3D/C2-flag WTAP & GFP ZC3H 100X.png]

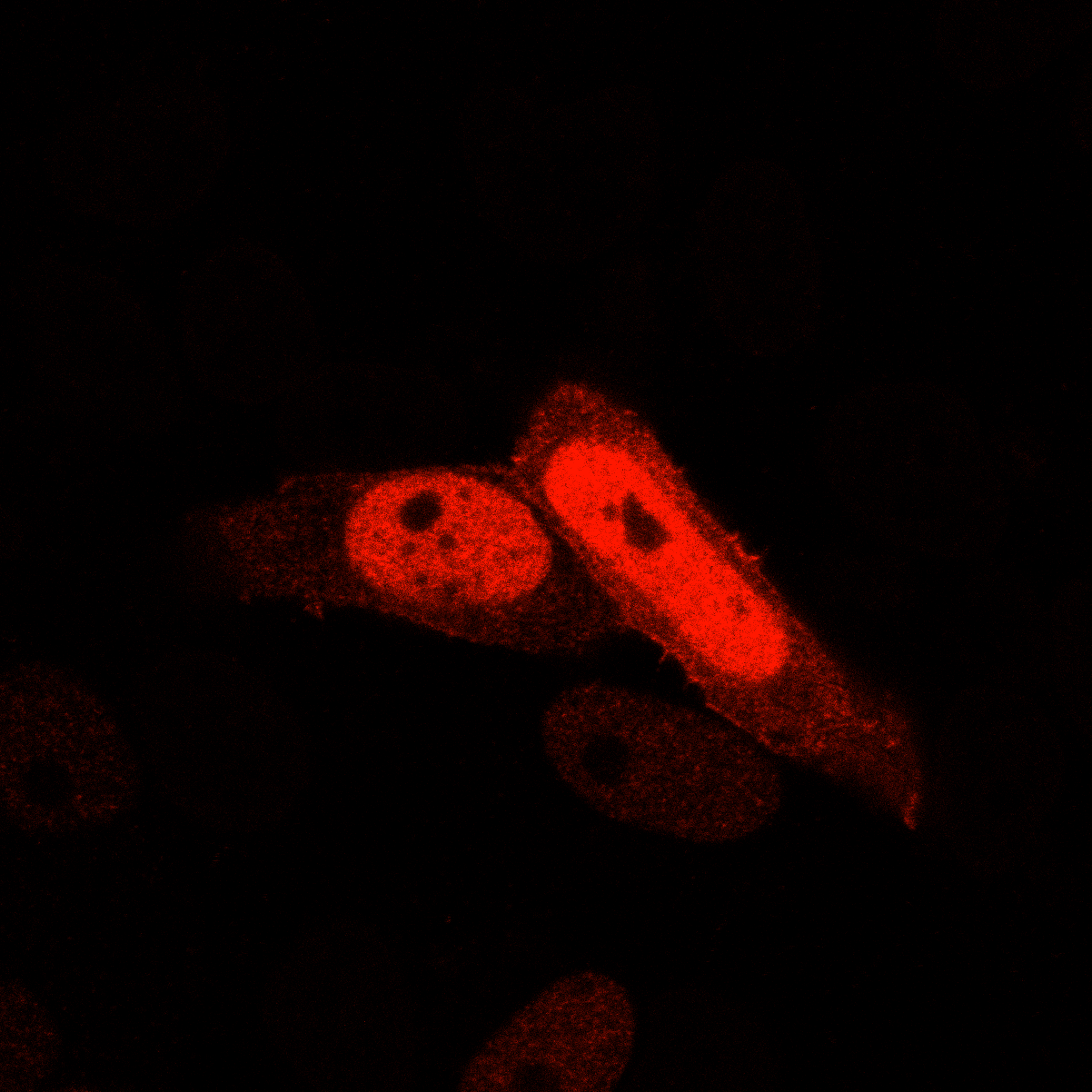

Supplement: Supplementary file 14 — Figure EV3 Source Data [file 44319_2026_815_MOESM14_ESM.zip › Figure EV3/3D/C2-flag WTAP & GFP ZC3H-C 100X-4.png]

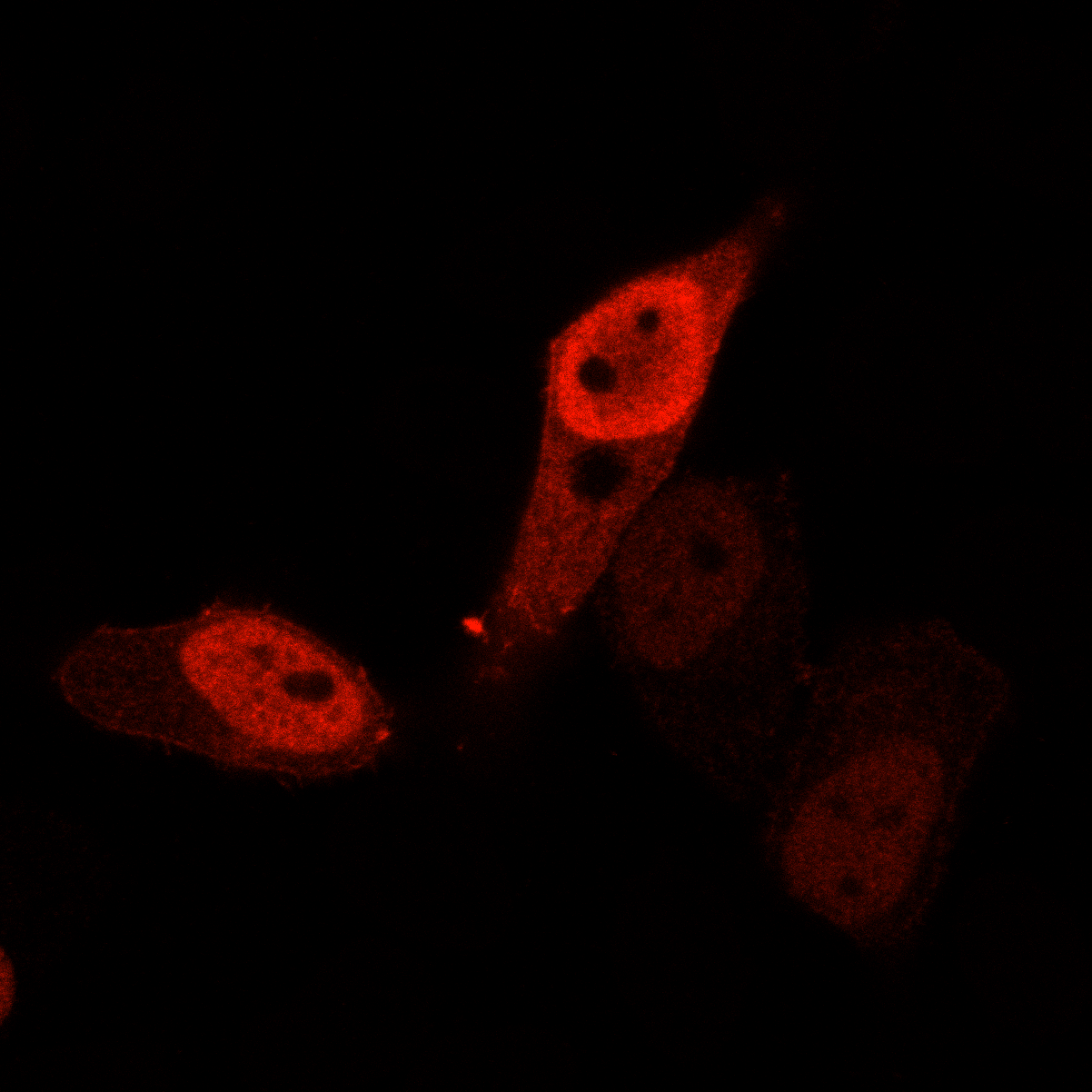

Supplement: Supplementary file 14 — Figure EV3 Source Data [file 44319_2026_815_MOESM14_ESM.zip › Figure EV3/3D/C2-flag WTAP & GFP ZC3H-C 100X-6.png]

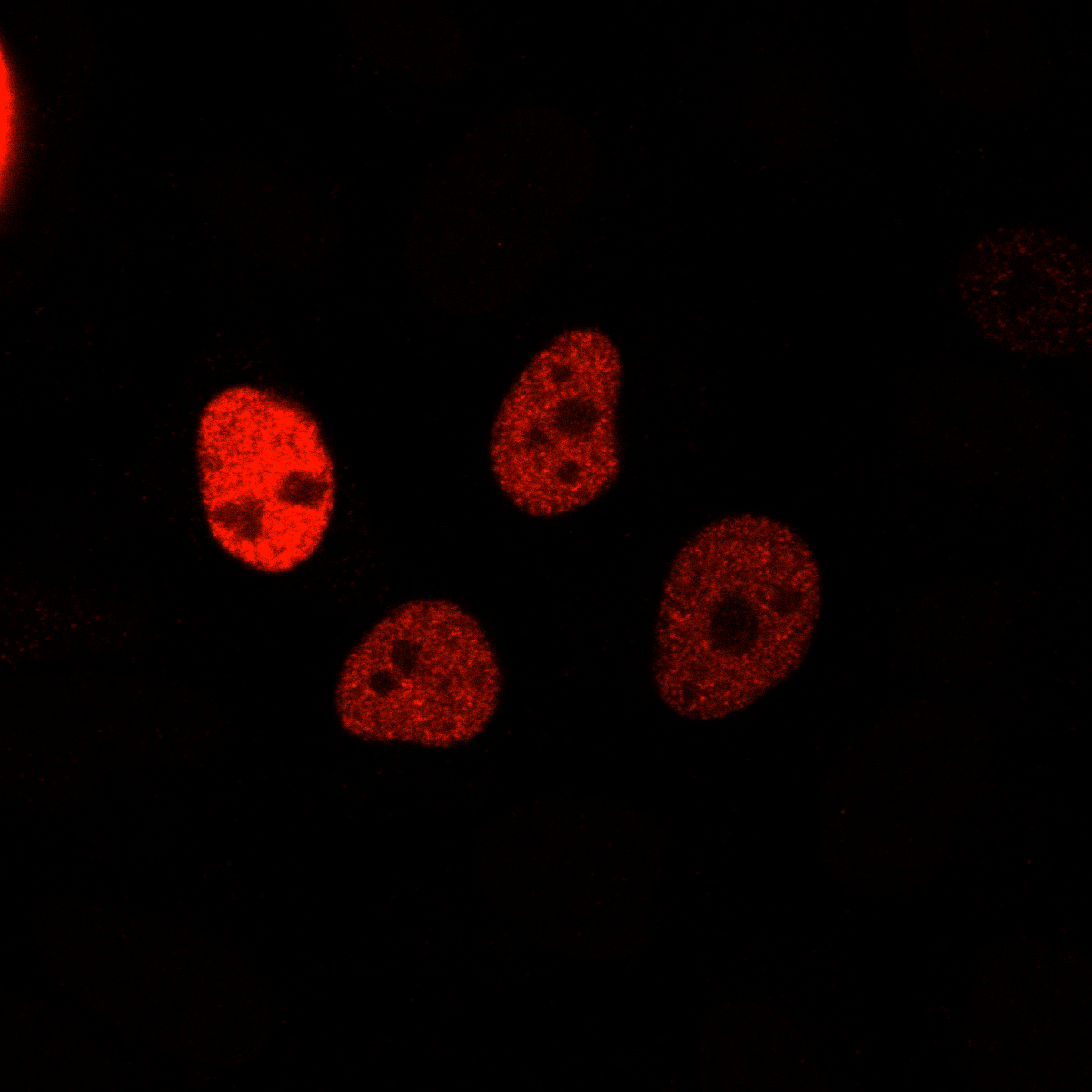

Supplement: Supplementary file 14 — Figure EV3 Source Data [file 44319_2026_815_MOESM14_ESM.zip › Figure EV3/3D/C2-flag WTAP L2E & GFP ZC3H-C 100X-2.png]

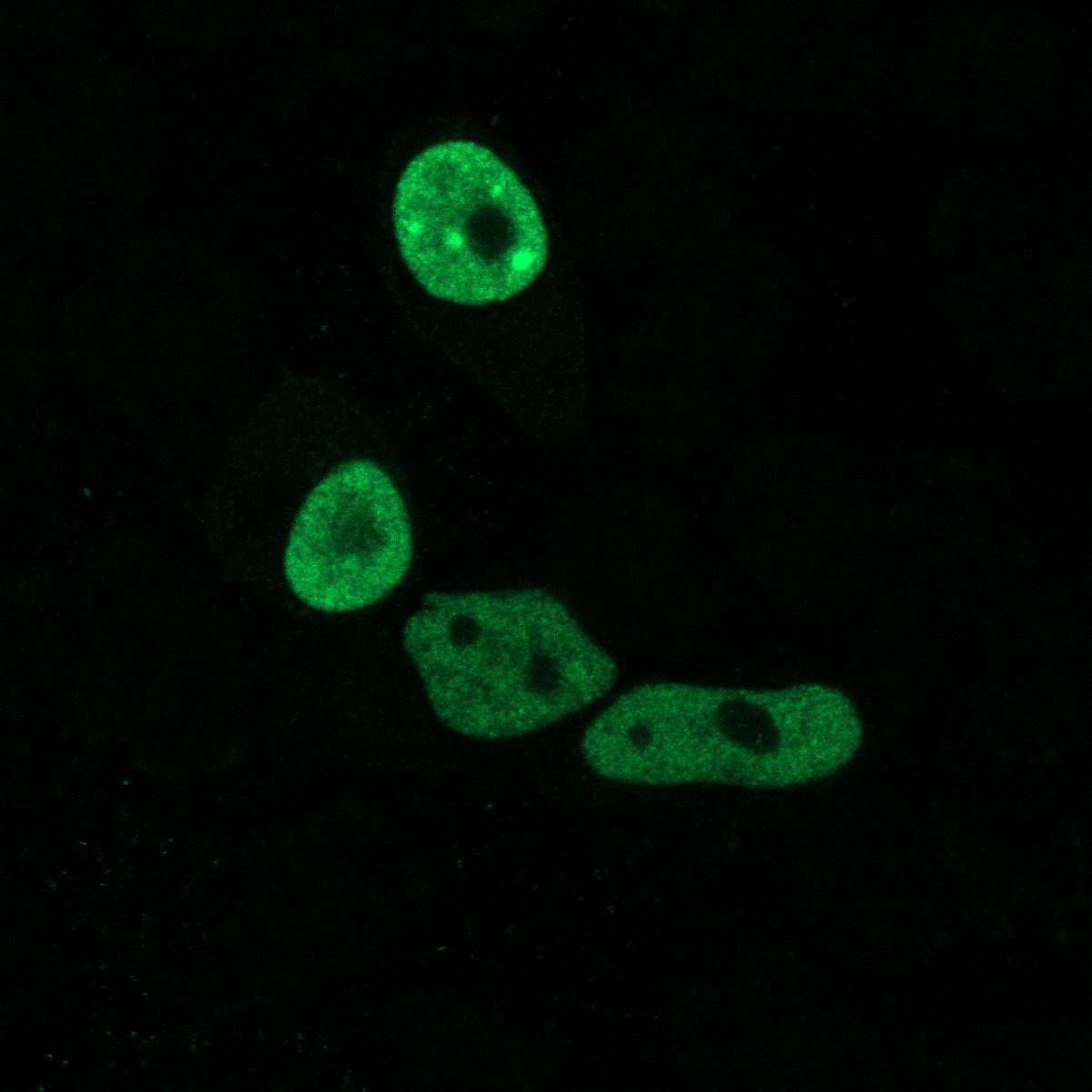

Supplement: Supplementary file 14 — Figure EV3 Source Data [file 44319_2026_815_MOESM14_ESM.zip › Figure EV3/3D/C4-flag WTAP & GFP ZC3H 100X.png]

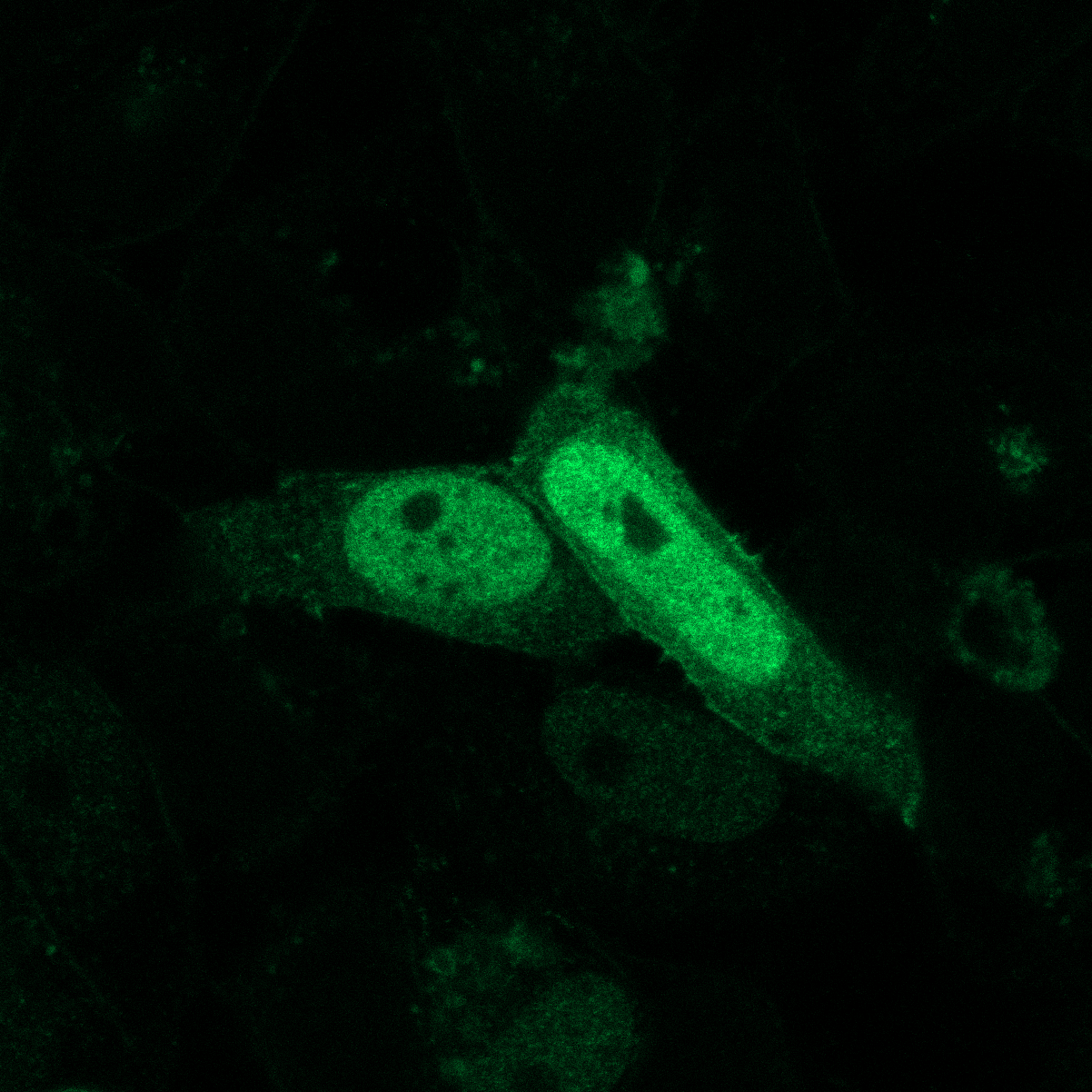

Supplement: Supplementary file 14 — Figure EV3 Source Data [file 44319_2026_815_MOESM14_ESM.zip › Figure EV3/3D/C4-flag WTAP & GFP ZC3H-C 100X-4.png]

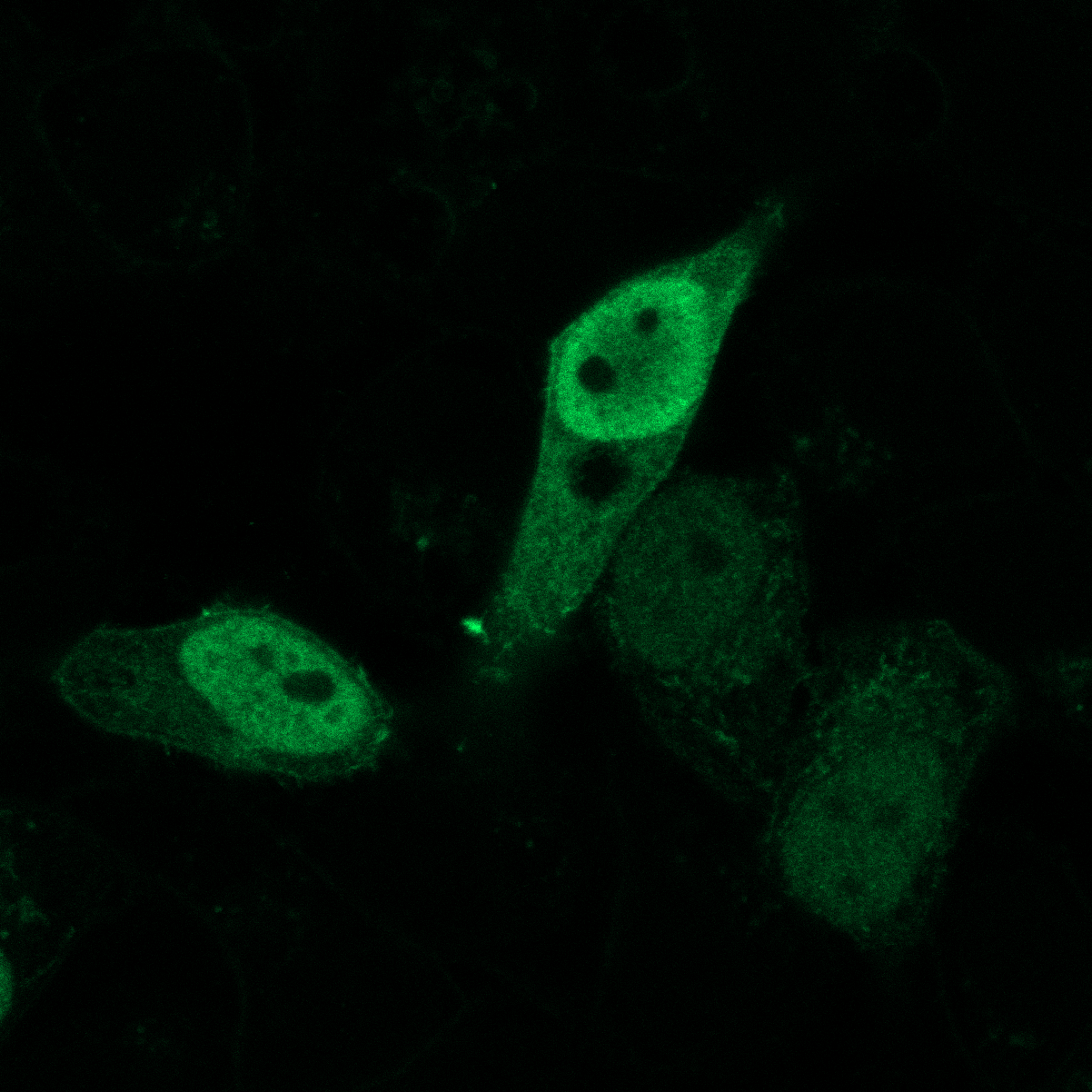

Supplement: Supplementary file 14 — Figure EV3 Source Data [file 44319_2026_815_MOESM14_ESM.zip › Figure EV3/3D/C4-flag WTAP & GFP ZC3H-C 100X-6.png]

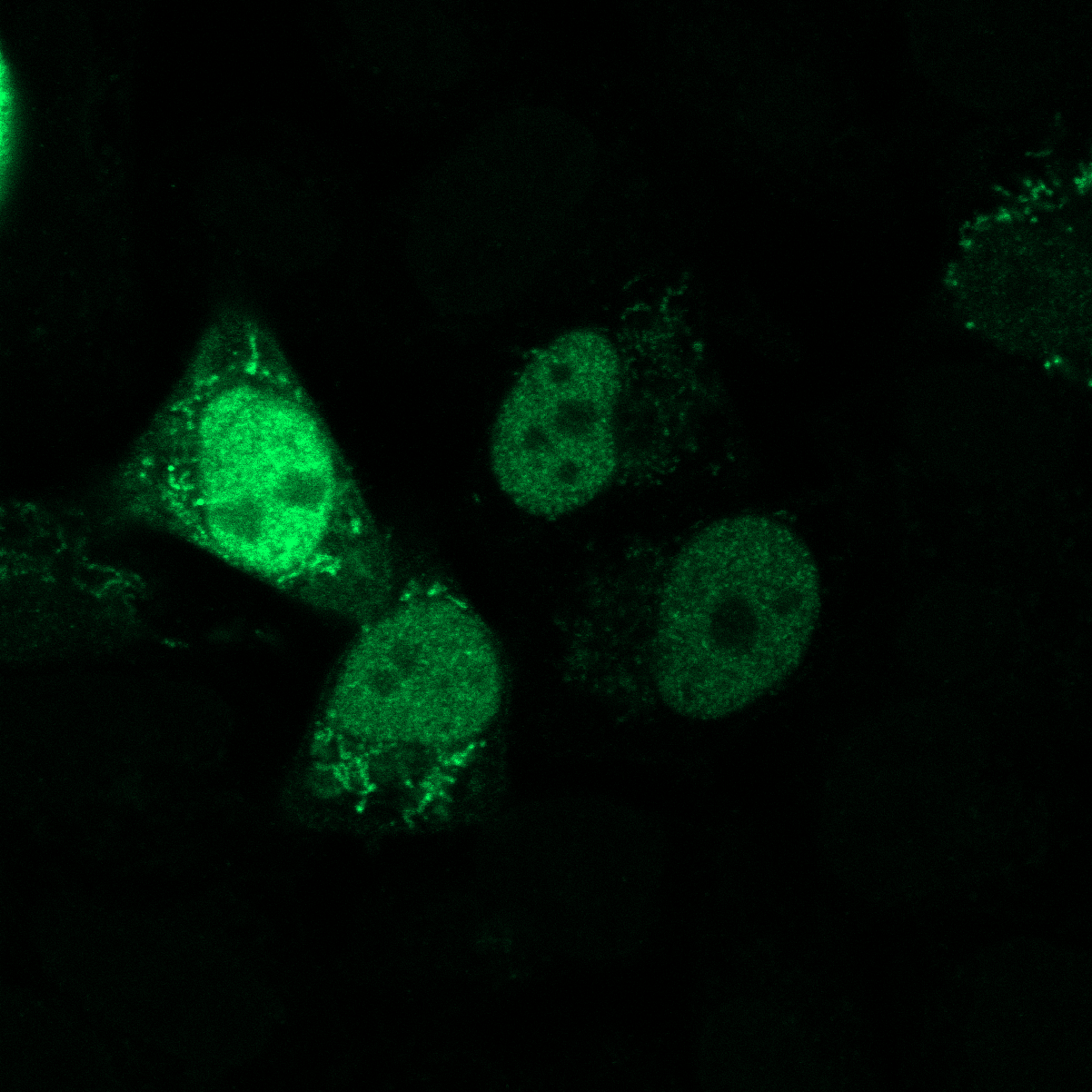

Supplement: Supplementary file 14 — Figure EV3 Source Data [file 44319_2026_815_MOESM14_ESM.zip › Figure EV3/3D/C4-flag WTAP L2E & GFP ZC3H-C 100X-2.png]

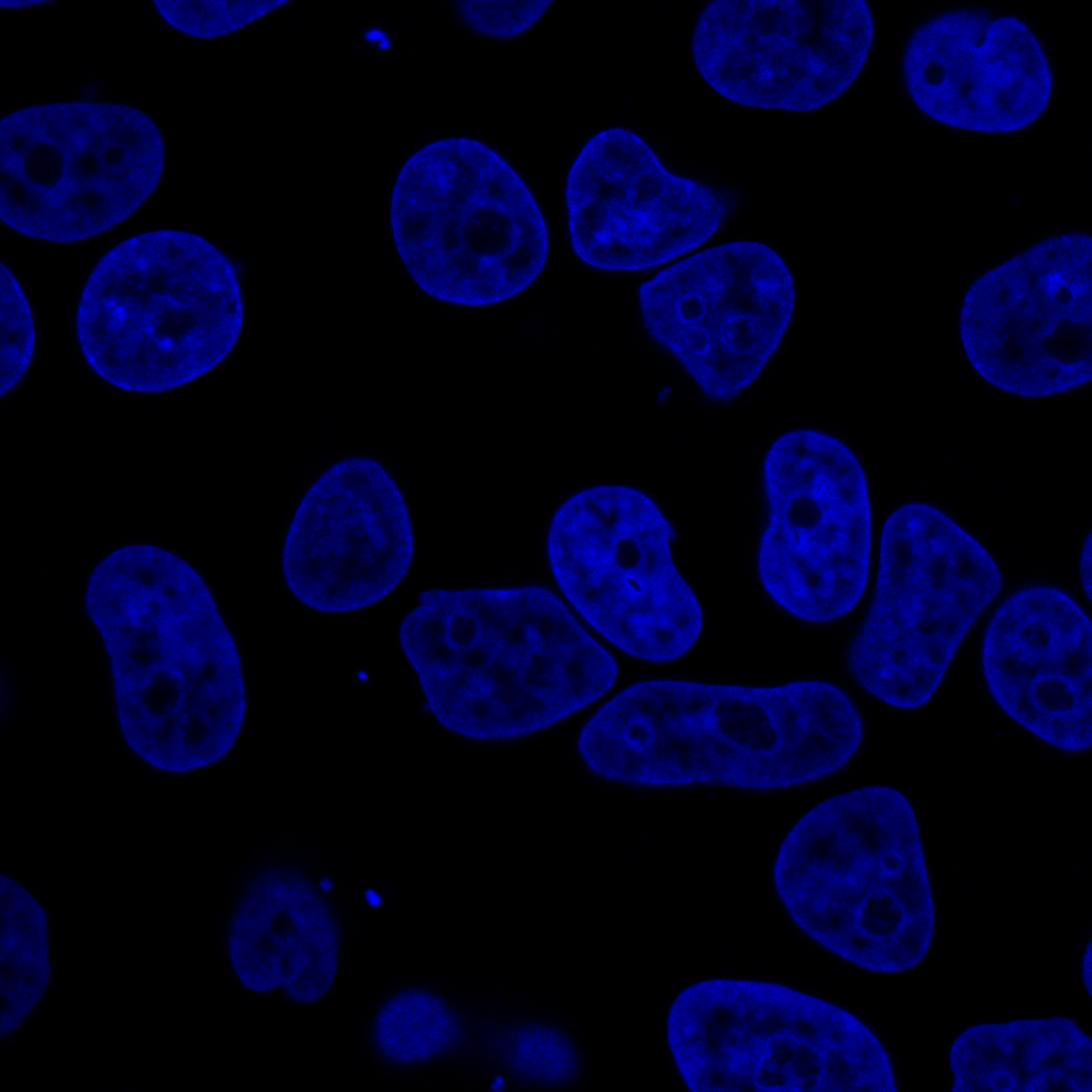

Supplement: Supplementary file 14 — Figure EV3 Source Data [file 44319_2026_815_MOESM14_ESM.zip › Figure EV3/3D/C6-flag WTAP & GFP ZC3H 100X.png]

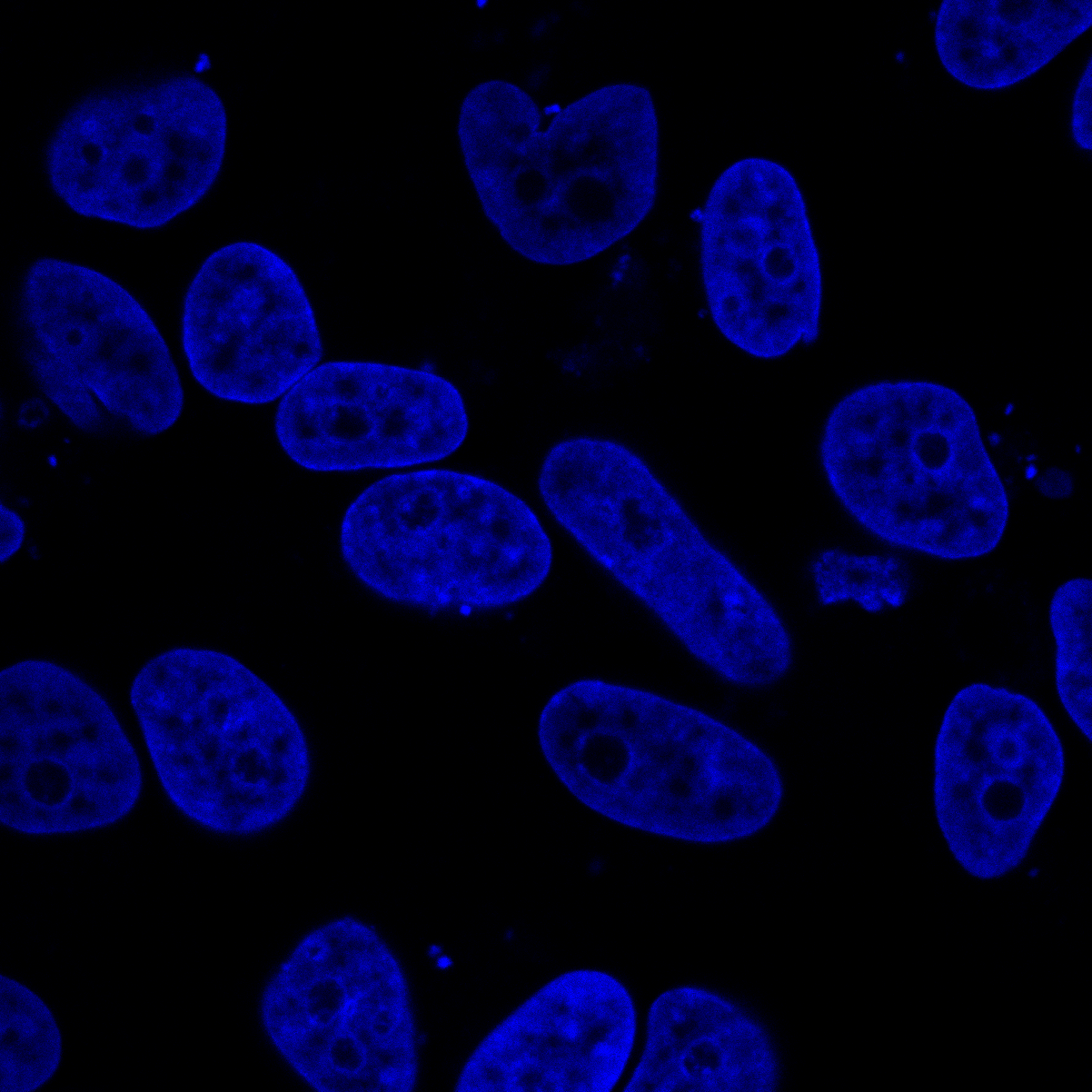

Supplement: Supplementary file 14 — Figure EV3 Source Data [file 44319_2026_815_MOESM14_ESM.zip › Figure EV3/3D/C6-flag WTAP & GFP ZC3H-C 100X-4.png]

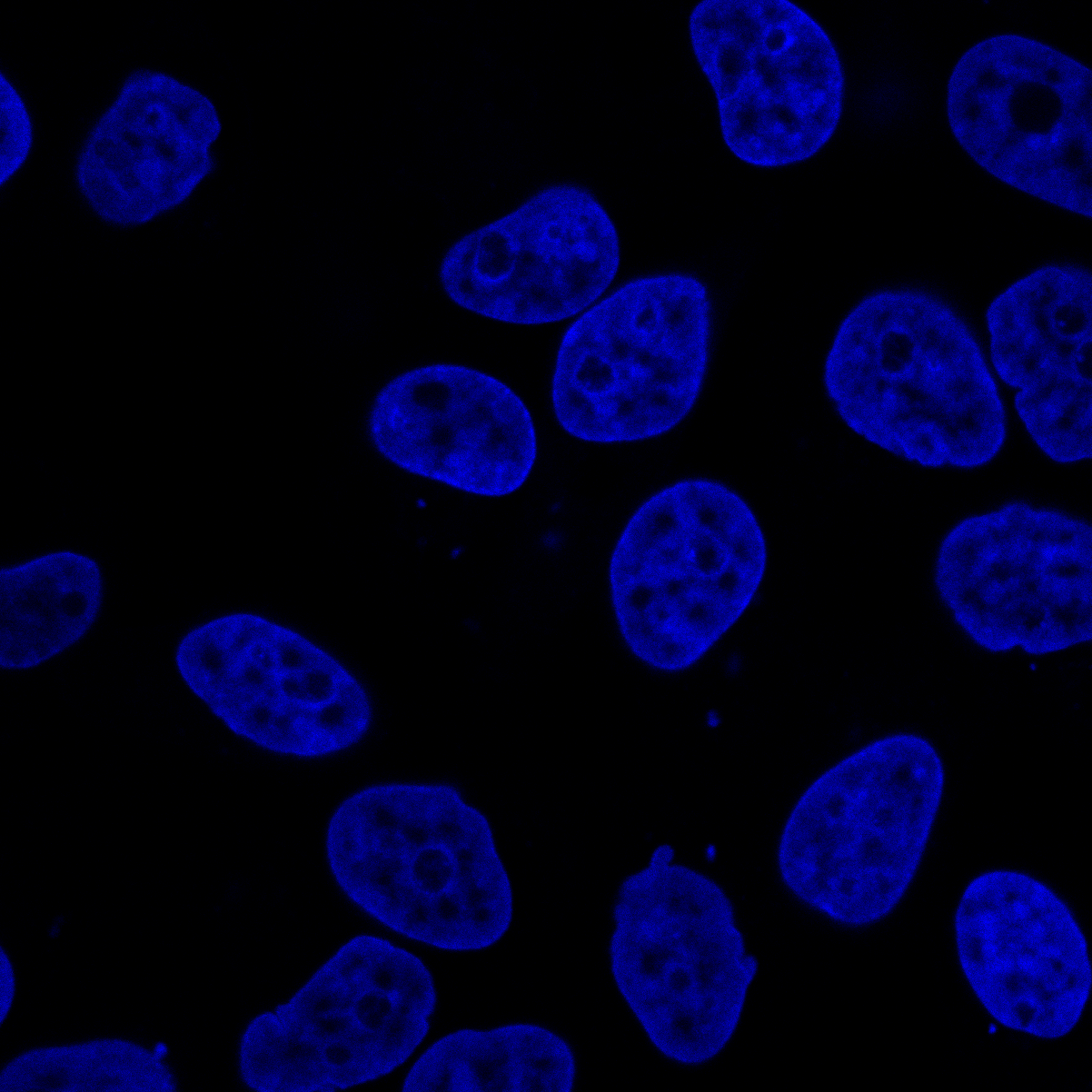

Supplement: Supplementary file 14 — Figure EV3 Source Data [file 44319_2026_815_MOESM14_ESM.zip › Figure EV3/3D/C6-flag WTAP & GFP ZC3H-C 100X-6.png]

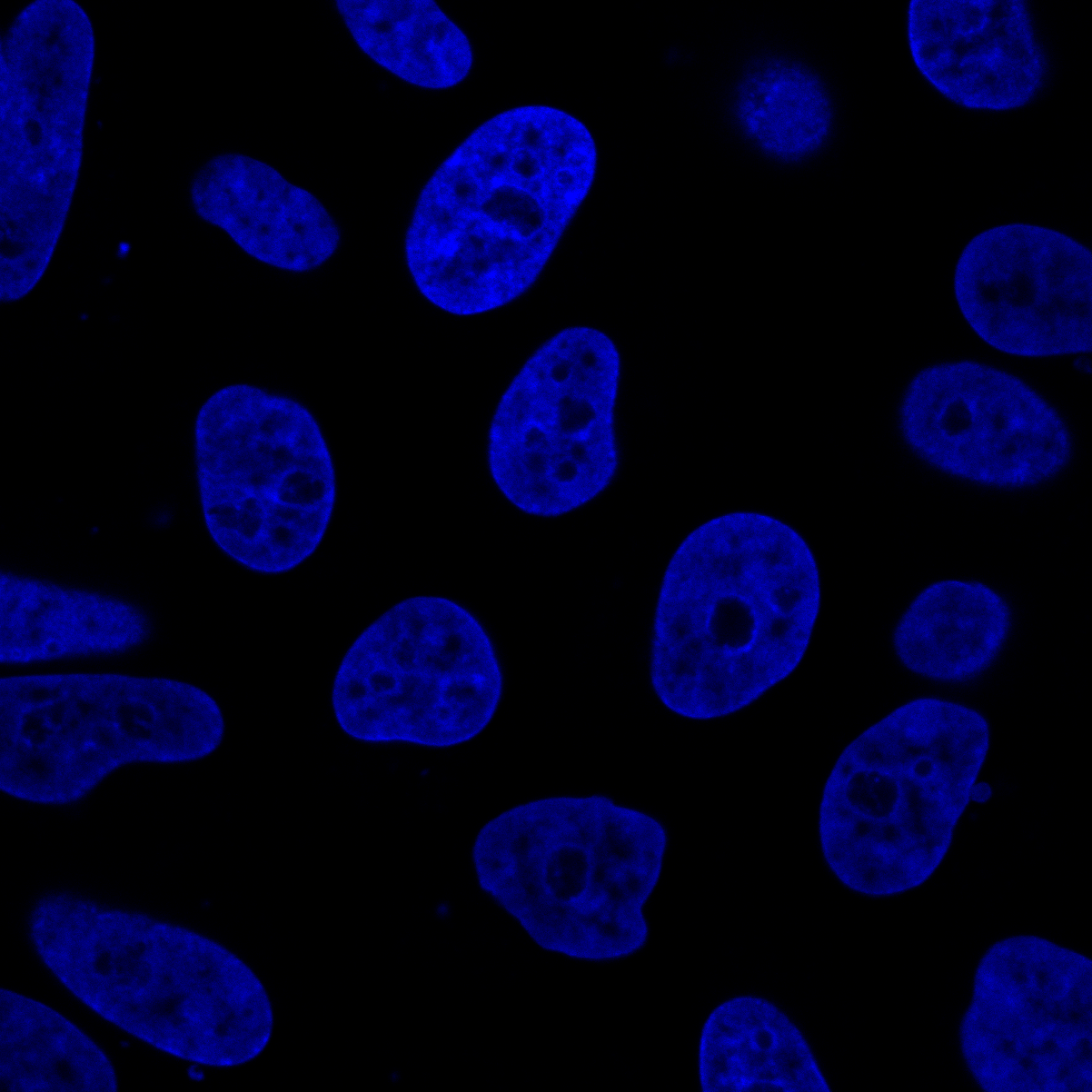

Supplement: Supplementary file 14 — Figure EV3 Source Data [file 44319_2026_815_MOESM14_ESM.zip › Figure EV3/3D/C6-flag WTAP L2E & GFP ZC3H-C 100X-2.png]

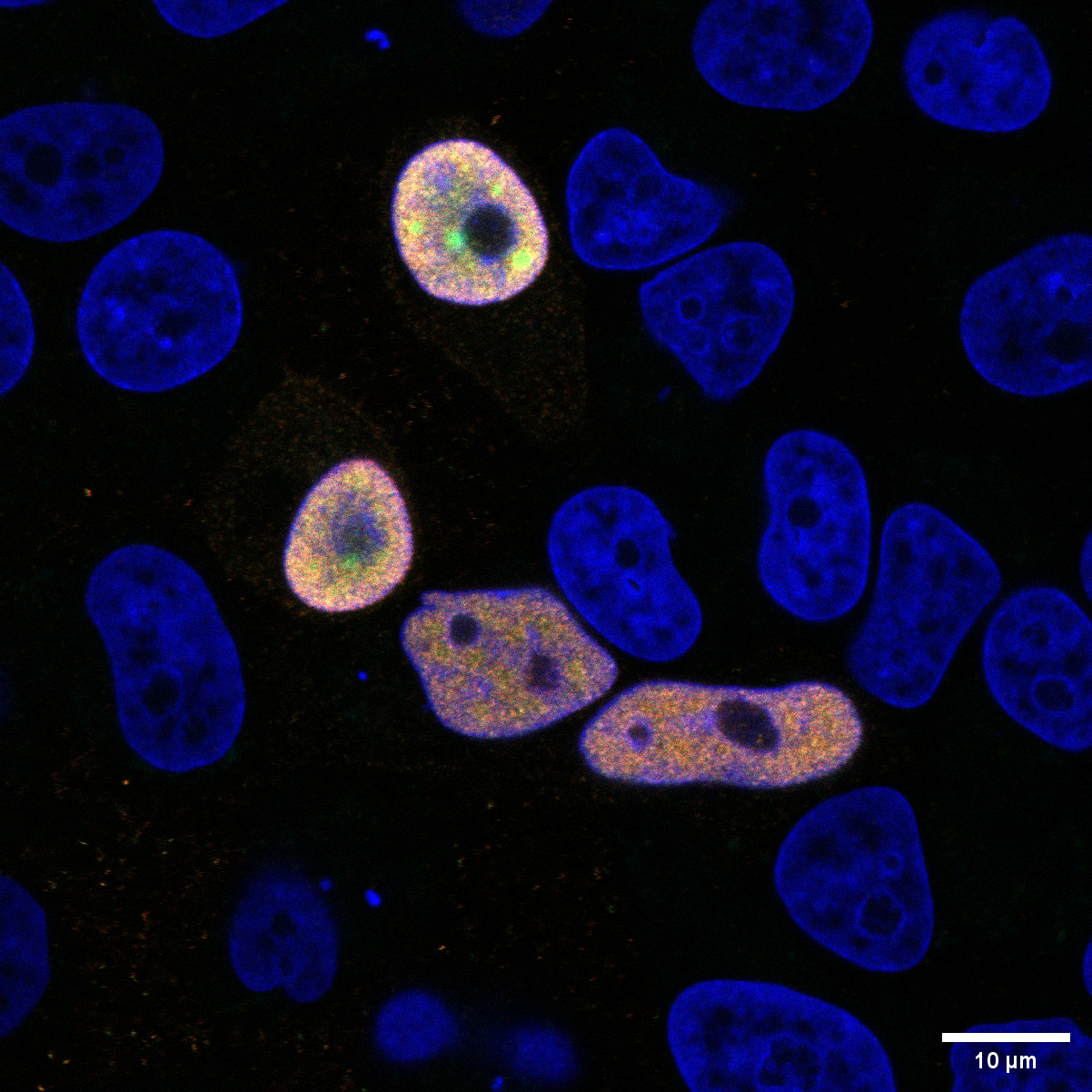

Supplement: Supplementary file 14 — Figure EV3 Source Data [file 44319_2026_815_MOESM14_ESM.zip › Figure EV3/3D/flag WTAP & GFP ZC3H 100X.png]

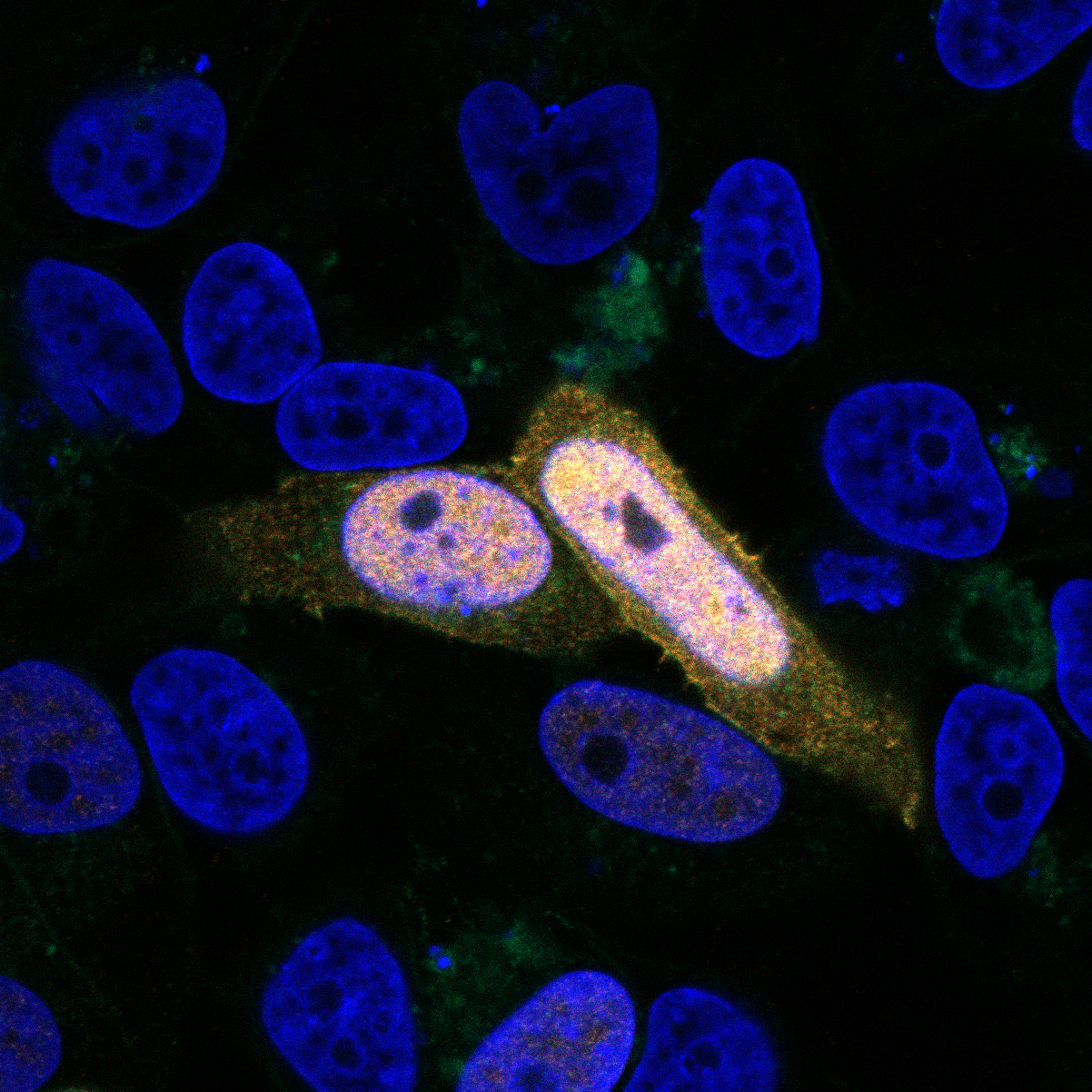

Supplement: Supplementary file 14 — Figure EV3 Source Data [file 44319_2026_815_MOESM14_ESM.zip › Figure EV3/3D/flag WTAP & GFP ZC3H-C 100X-4.png]

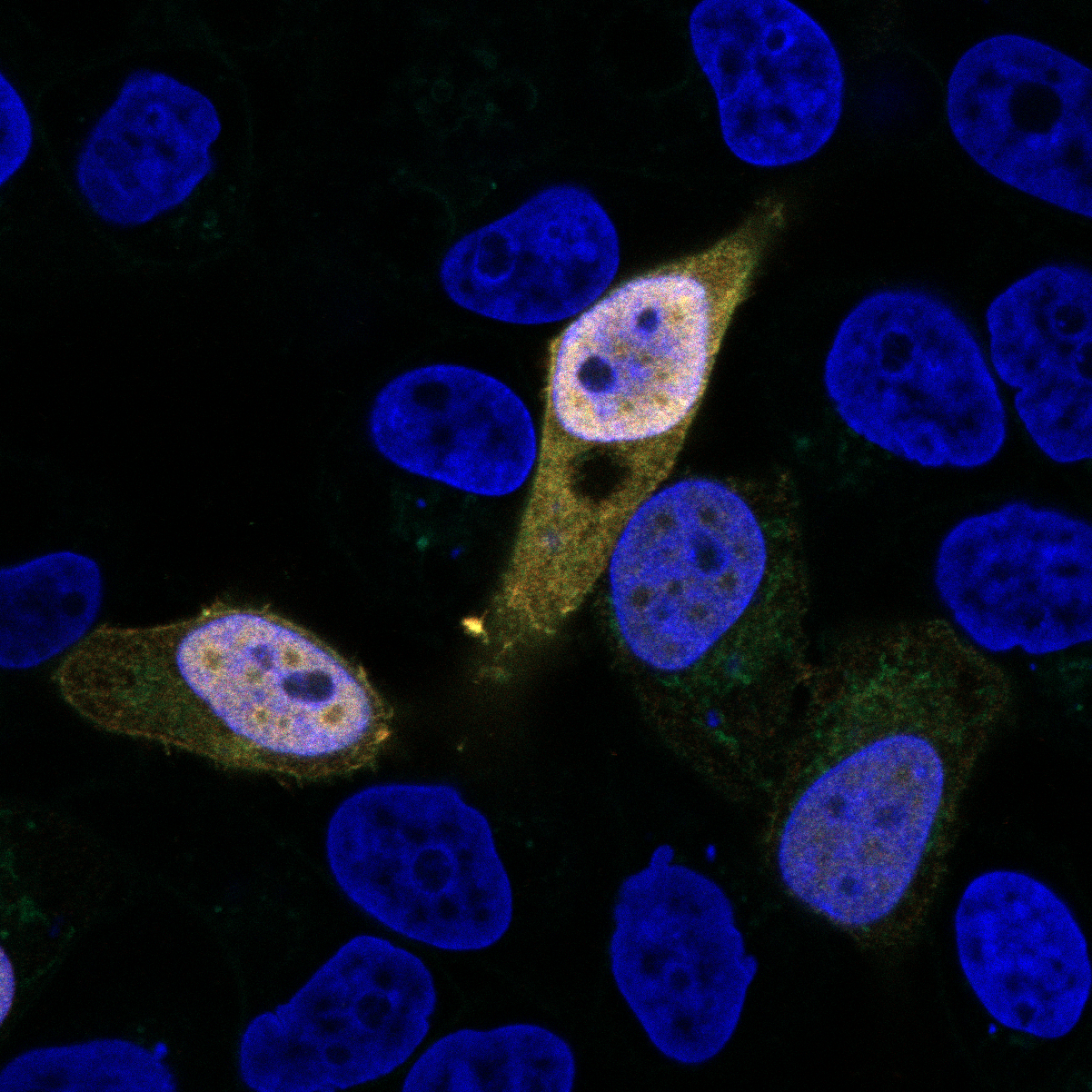

Supplement: Supplementary file 14 — Figure EV3 Source Data [file 44319_2026_815_MOESM14_ESM.zip › Figure EV3/3D/flag WTAP & GFP ZC3H-C 100X-6.png]

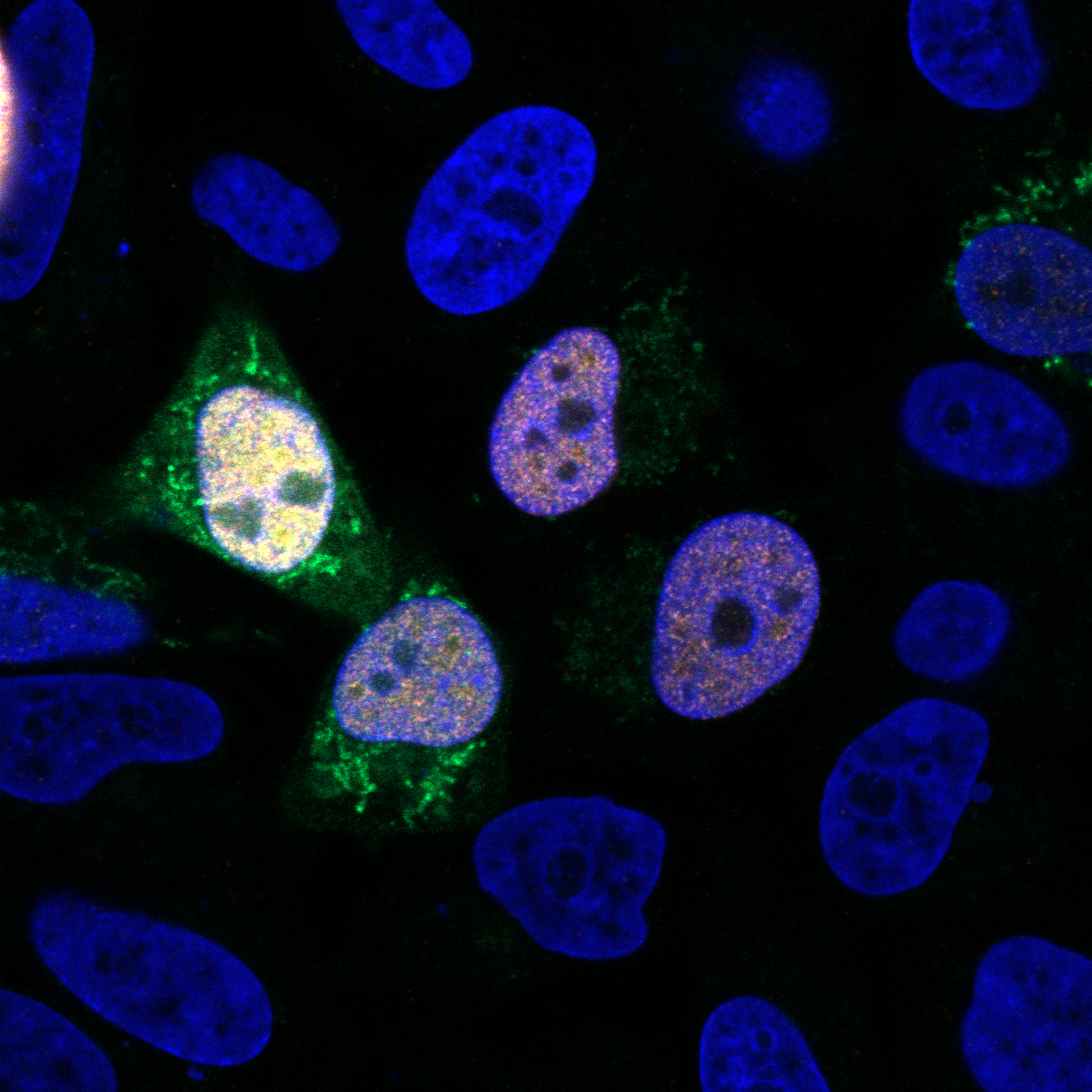

Supplement: Supplementary file 14 — Figure EV3 Source Data [file 44319_2026_815_MOESM14_ESM.zip › Figure EV3/3D/flag WTAP L2E & GFP ZC3H-C 100X-2.png]

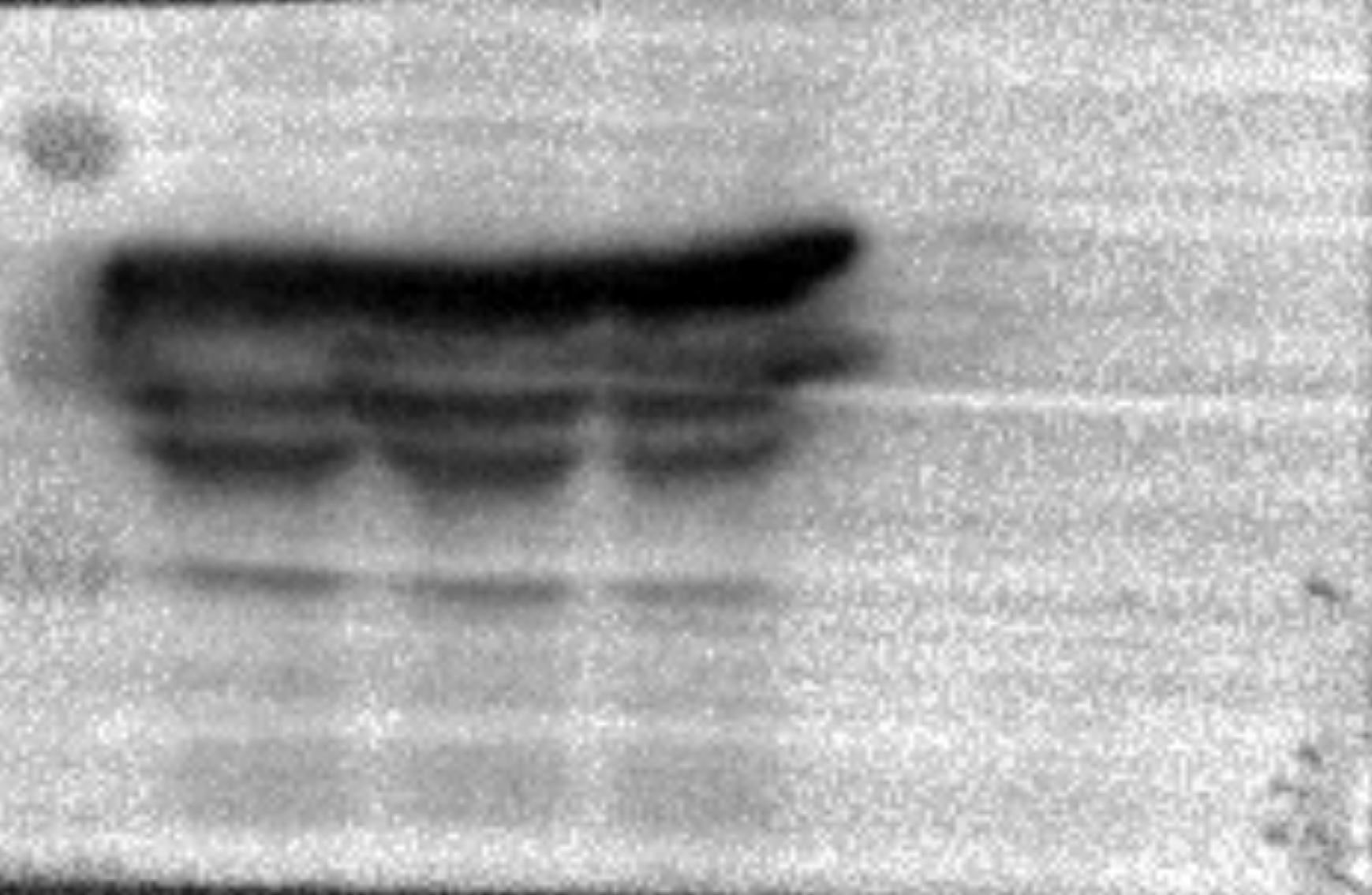

Supplement: Supplementary file 15 — Figure EV4 Source Data [file 44319_2026_815_MOESM15_ESM.zip › Figure EV4/4A/anti GAPDH.tif]

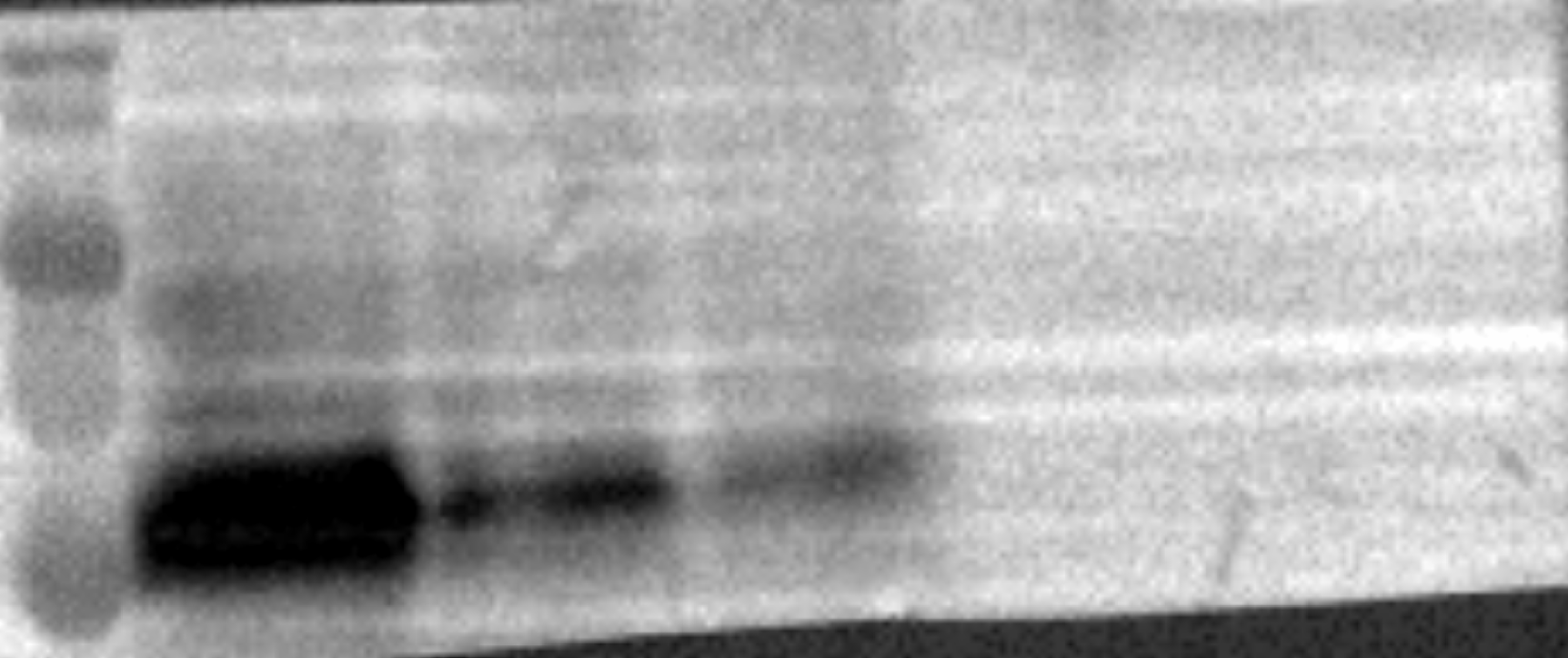

Supplement: Supplementary file 15 — Figure EV4 Source Data [file 44319_2026_815_MOESM15_ESM.zip › Figure EV4/4A/anti WTAP.tif]

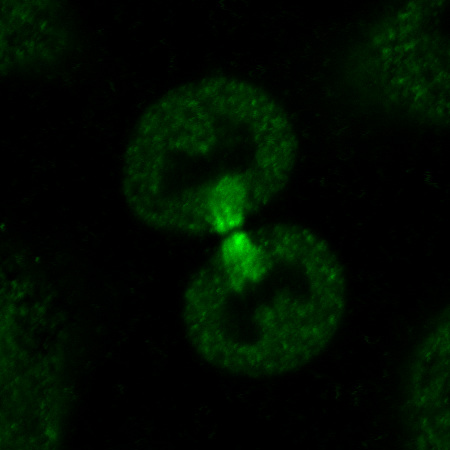

Supplement: Supplementary file 15 — Figure EV4 Source Data [file 44319_2026_815_MOESM15_ESM.zip › Figure EV4/4B/hela-WTAPandatublin-1.jpg]

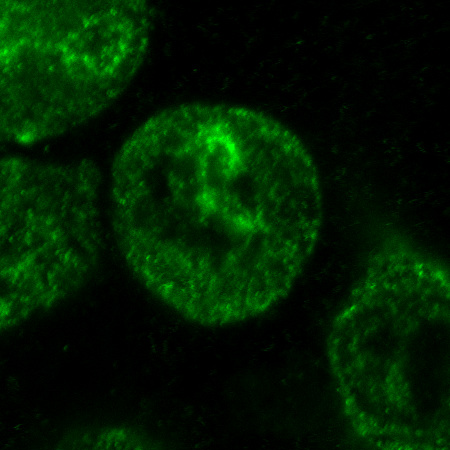

Supplement: Supplementary file 15 — Figure EV4 Source Data [file 44319_2026_815_MOESM15_ESM.zip › Figure EV4/4B/hela-WTAPandatublin-17A.jpg]

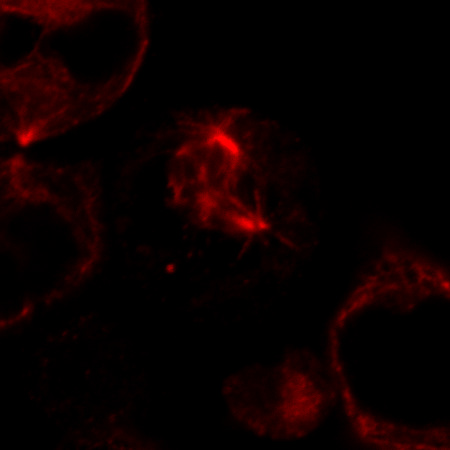

Supplement: Supplementary file 15 — Figure EV4 Source Data [file 44319_2026_815_MOESM15_ESM.zip › Figure EV4/4B/hela-WTAPandatublin-17B.jpg]

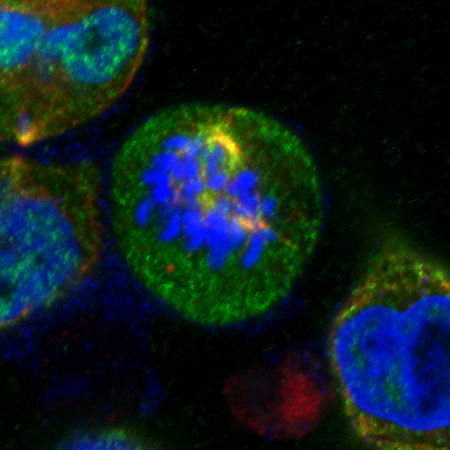

Supplement: Supplementary file 15 — Figure EV4 Source Data [file 44319_2026_815_MOESM15_ESM.zip › Figure EV4/4B/hela-WTAPandatublin-17C.jpg]

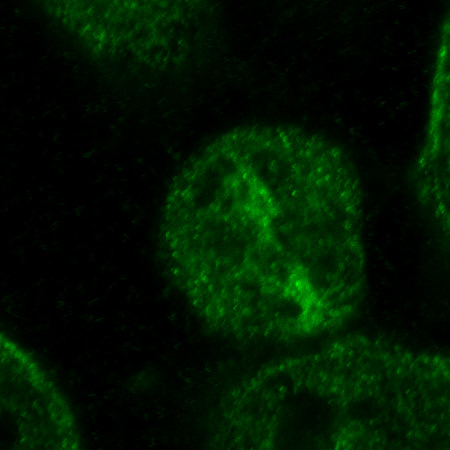

Supplement: Supplementary file 15 — Figure EV4 Source Data [file 44319_2026_815_MOESM15_ESM.zip › Figure EV4/4B/hela-WTAPandatublin-18A.jpg]

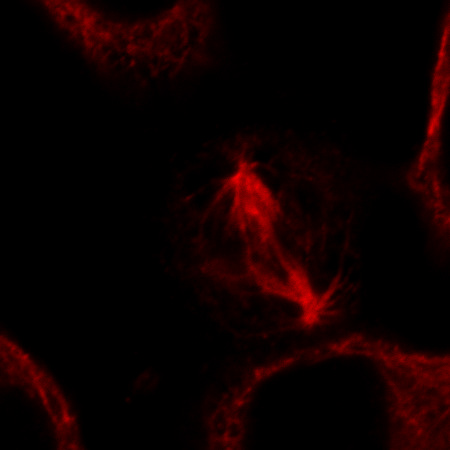

Supplement: Supplementary file 15 — Figure EV4 Source Data [file 44319_2026_815_MOESM15_ESM.zip › Figure EV4/4B/hela-WTAPandatublin-18B.jpg]

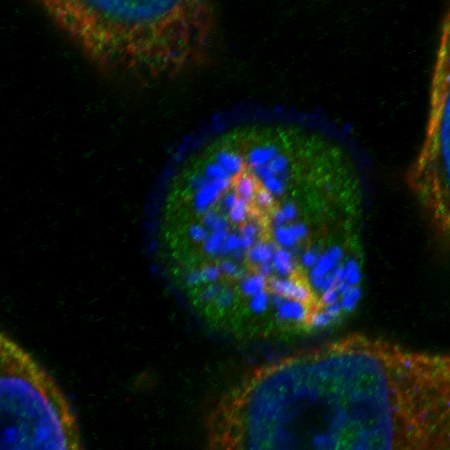

Supplement: Supplementary file 15 — Figure EV4 Source Data [file 44319_2026_815_MOESM15_ESM.zip › Figure EV4/4B/hela-WTAPandatublin-18C.jpg]

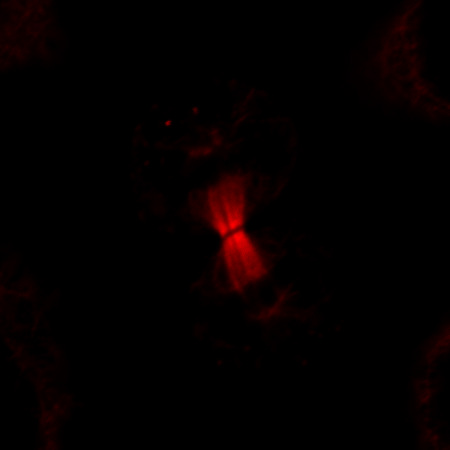

Supplement: Supplementary file 15 — Figure EV4 Source Data [file 44319_2026_815_MOESM15_ESM.zip › Figure EV4/4B/hela-WTAPandatublin-2.jpg]

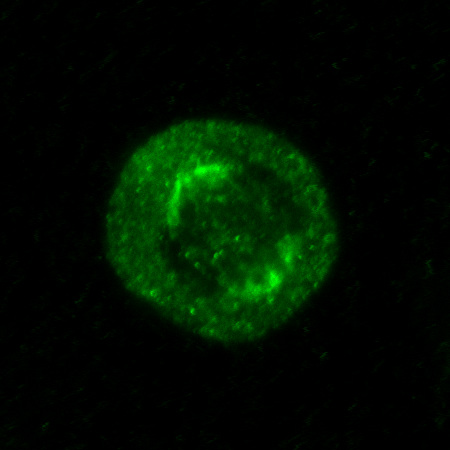

Supplement: Supplementary file 15 — Figure EV4 Source Data [file 44319_2026_815_MOESM15_ESM.zip › Figure EV4/4B/hela-WTAPandatublin-20A.jpg]

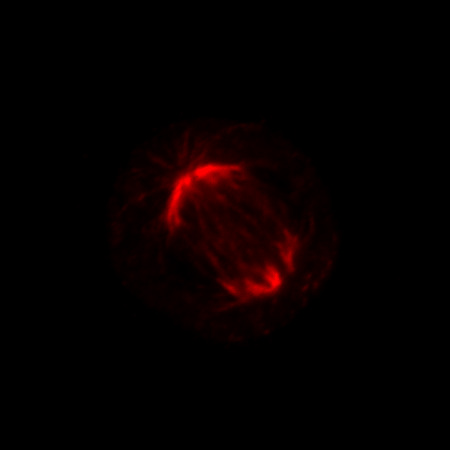

Supplement: Supplementary file 15 — Figure EV4 Source Data [file 44319_2026_815_MOESM15_ESM.zip › Figure EV4/4B/hela-WTAPandatublin-20B.jpg]

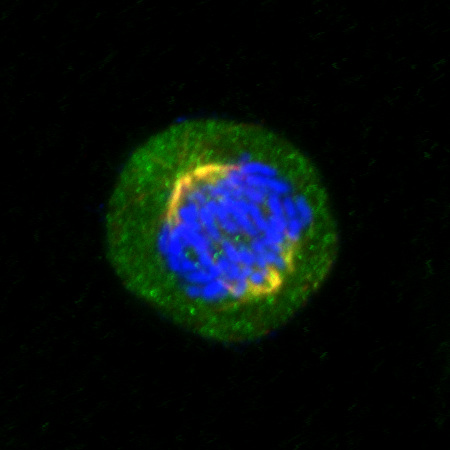

Supplement: Supplementary file 15 — Figure EV4 Source Data [file 44319_2026_815_MOESM15_ESM.zip › Figure EV4/4B/hela-WTAPandatublin-20C.jpg]

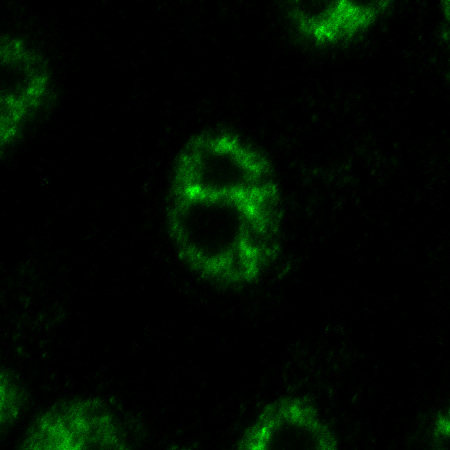

Supplement: Supplementary file 15 — Figure EV4 Source Data [file 44319_2026_815_MOESM15_ESM.zip › Figure EV4/4B/hela-WTAPandatublin-24A.jpg]

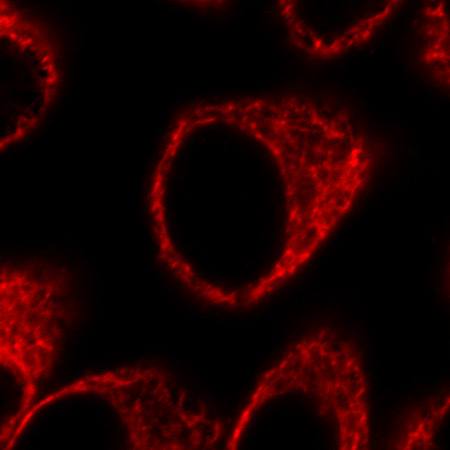

Supplement: Supplementary file 15 — Figure EV4 Source Data [file 44319_2026_815_MOESM15_ESM.zip › Figure EV4/4B/hela-WTAPandatublin-24B.jpg]

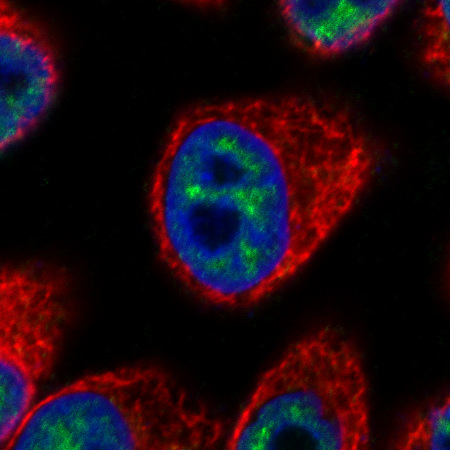

Supplement: Supplementary file 15 — Figure EV4 Source Data [file 44319_2026_815_MOESM15_ESM.zip › Figure EV4/4B/hela-WTAPandatublin-24C.jpg]

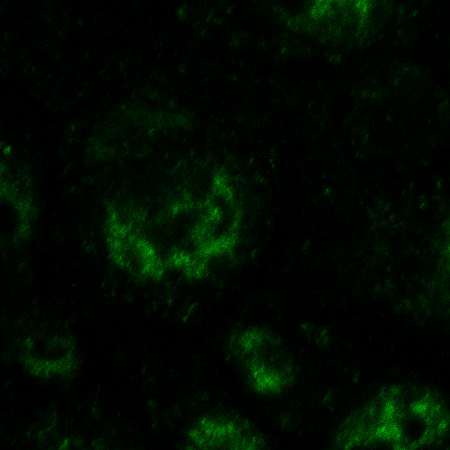

Supplement: Supplementary file 15 — Figure EV4 Source Data [file 44319_2026_815_MOESM15_ESM.zip › Figure EV4/4B/hela-WTAPandatublin-26A.jpg]

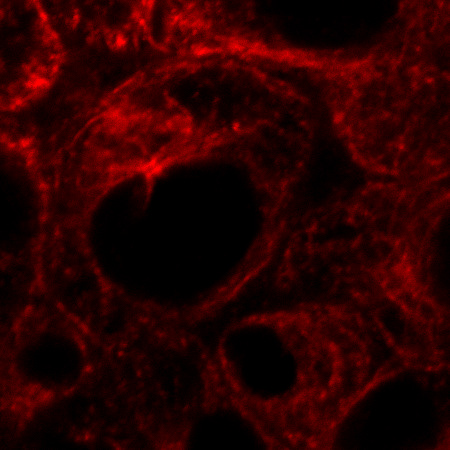

Supplement: Supplementary file 15 — Figure EV4 Source Data [file 44319_2026_815_MOESM15_ESM.zip › Figure EV4/4B/hela-WTAPandatublin-26B.jpg]

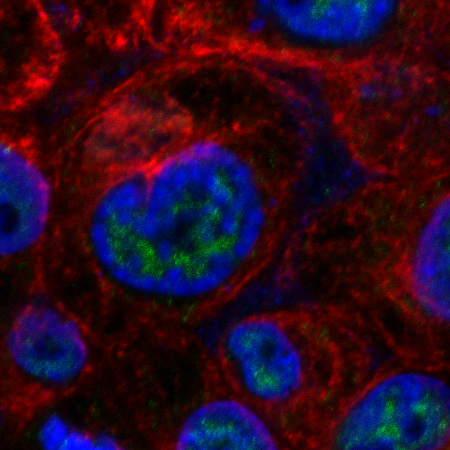

Supplement: Supplementary file 15 — Figure EV4 Source Data [file 44319_2026_815_MOESM15_ESM.zip › Figure EV4/4B/hela-WTAPandatublin-26C.jpg]

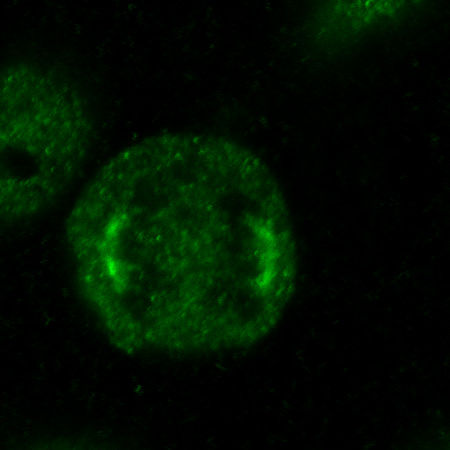

Supplement: Supplementary file 15 — Figure EV4 Source Data [file 44319_2026_815_MOESM15_ESM.zip › Figure EV4/4B/hela-WTAPandatublin-2A.jpg]

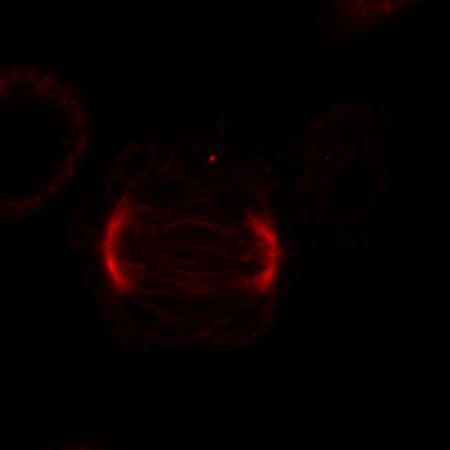

Supplement: Supplementary file 15 — Figure EV4 Source Data [file 44319_2026_815_MOESM15_ESM.zip › Figure EV4/4B/hela-WTAPandatublin-2B.jpg]

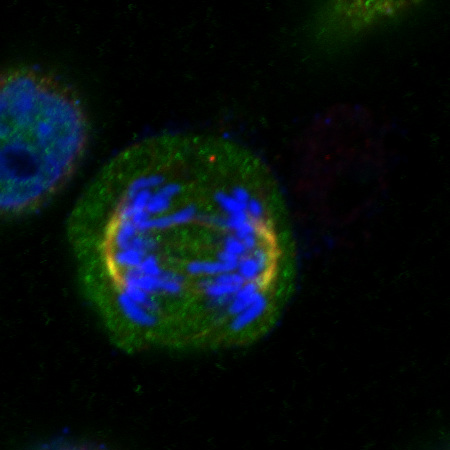

Supplement: Supplementary file 15 — Figure EV4 Source Data [file 44319_2026_815_MOESM15_ESM.zip › Figure EV4/4B/hela-WTAPandatublin-2C.jpg]
