# Supplementary figures and images for: WTAP tetramer ensures m6A writer assembly and faithful mitosis (part 4 of 4)
Source: EMBO Rep. 2026 Jun 2;27(13):3842–62. doi: 10.1038/s44319-026-00815-3 (PMC13354555; doi:10.1038/s44319-026-00815-3)

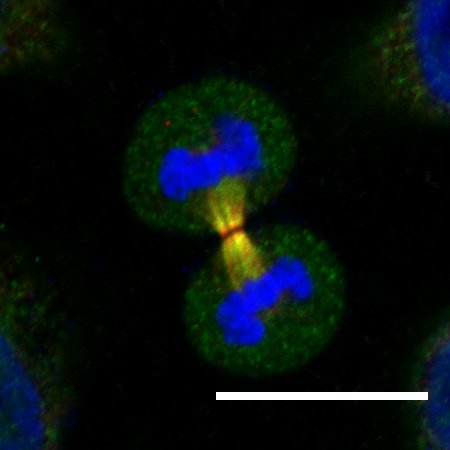

Supplement: Supplementary file 15 — Figure EV4 Source Data [file 44319_2026_815_MOESM15_ESM.zip › Figure EV4/4B/hela-WTAPandatublin-3 bar.jpg]

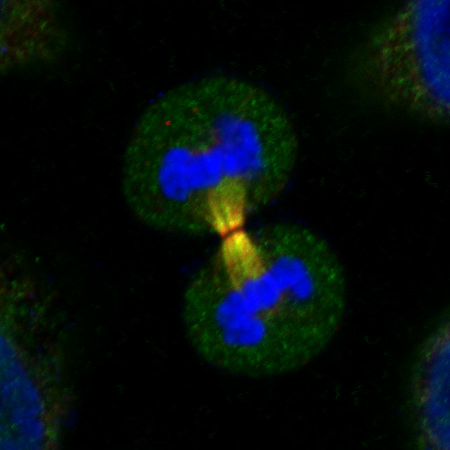

Supplement: Supplementary file 15 — Figure EV4 Source Data [file 44319_2026_815_MOESM15_ESM.zip › Figure EV4/4B/hela-WTAPandatublin-3.jpg]

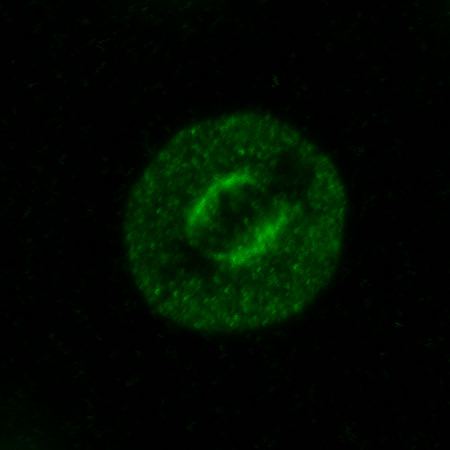

Supplement: Supplementary file 15 — Figure EV4 Source Data [file 44319_2026_815_MOESM15_ESM.zip › Figure EV4/4B/中期-1.jpg]

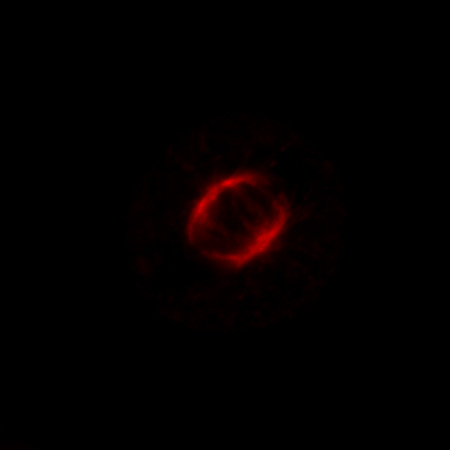

Supplement: Supplementary file 15 — Figure EV4 Source Data [file 44319_2026_815_MOESM15_ESM.zip › Figure EV4/4B/中期-2.jpg]

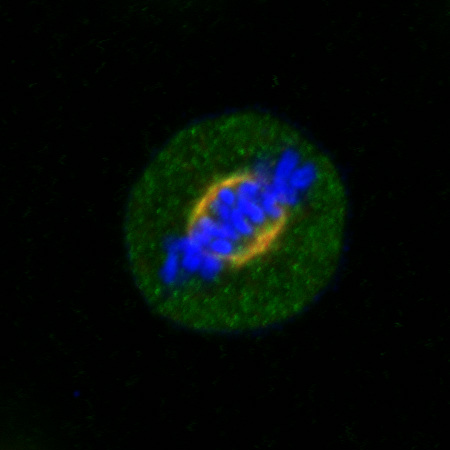

Supplement: Supplementary file 15 — Figure EV4 Source Data [file 44319_2026_815_MOESM15_ESM.zip › Figure EV4/4B/中期-3.jpg]

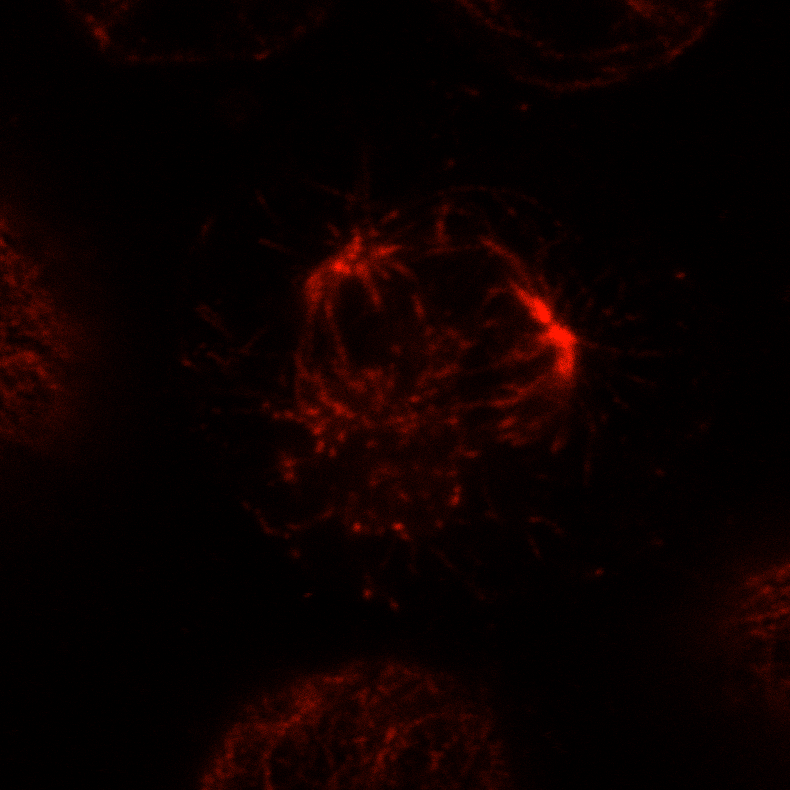

Supplement: Supplementary file 15 — Figure EV4 Source Data [file 44319_2026_815_MOESM15_ESM.zip › Figure EV4/4C/C2-PLVX L2E 100X1.7-2.png]

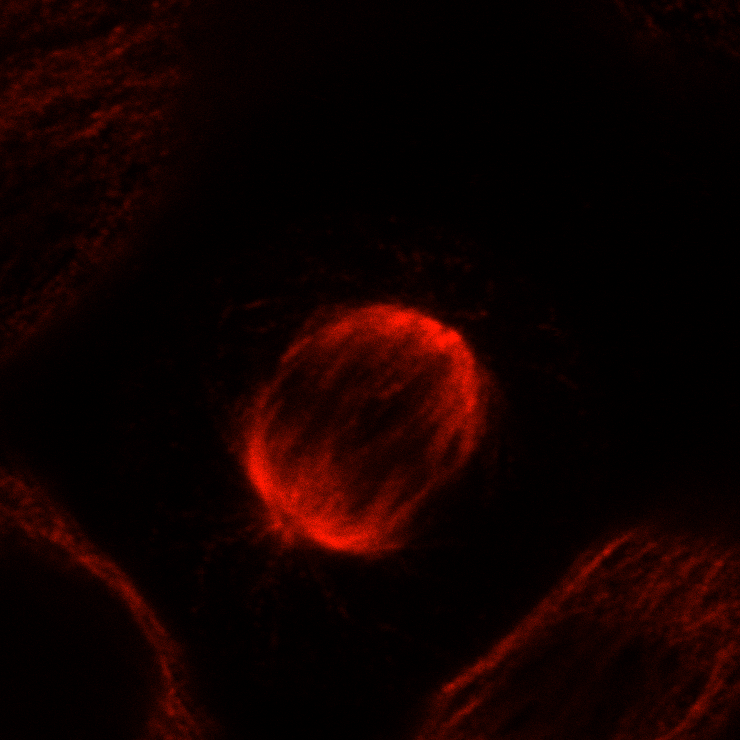

Supplement: Supplementary file 15 — Figure EV4 Source Data [file 44319_2026_815_MOESM15_ESM.zip › Figure EV4/4C/C2-PLVX L2E 100X1.7-3.png]

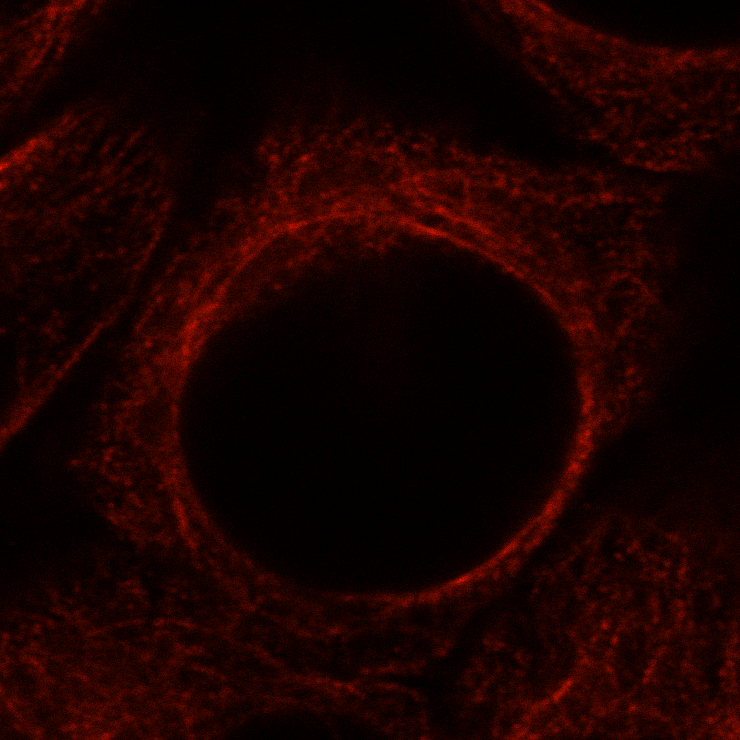

Supplement: Supplementary file 15 — Figure EV4 Source Data [file 44319_2026_815_MOESM15_ESM.zip › Figure EV4/4C/C2-PLVX L2E 100X1.7-4.png]

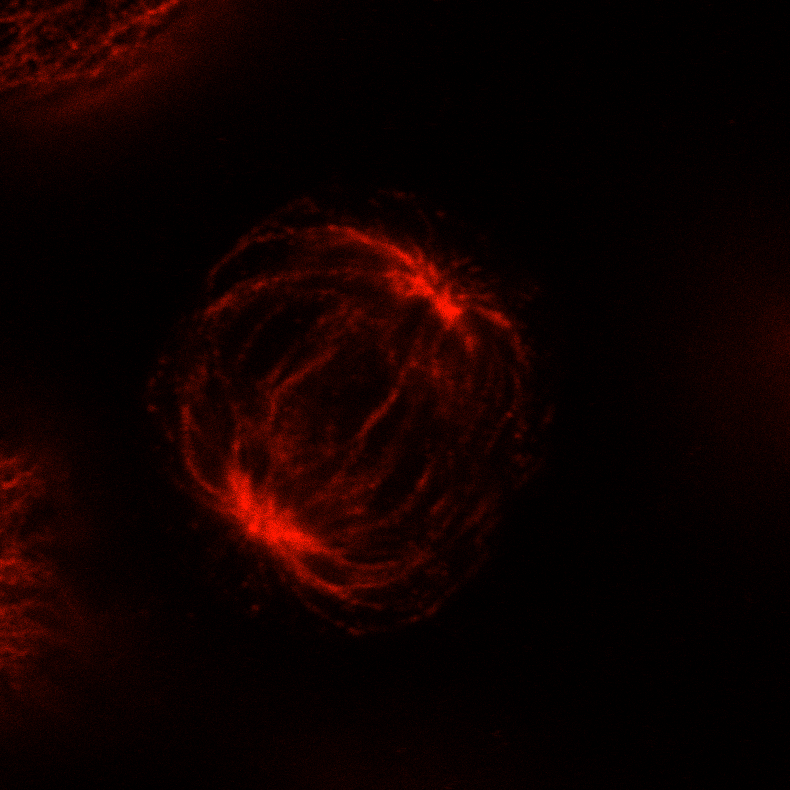

Supplement: Supplementary file 15 — Figure EV4 Source Data [file 44319_2026_815_MOESM15_ESM.zip › Figure EV4/4C/C2-PLVX L2E 100X1.7-5.png]

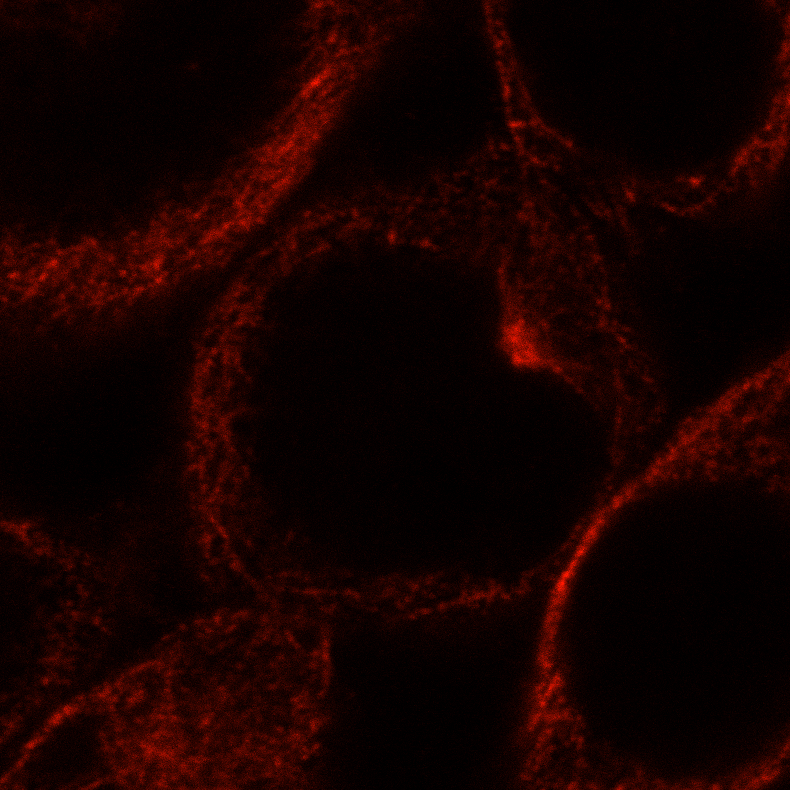

Supplement: Supplementary file 15 — Figure EV4 Source Data [file 44319_2026_815_MOESM15_ESM.zip › Figure EV4/4C/C2-PLVX L2E 100X1.7-7.png]

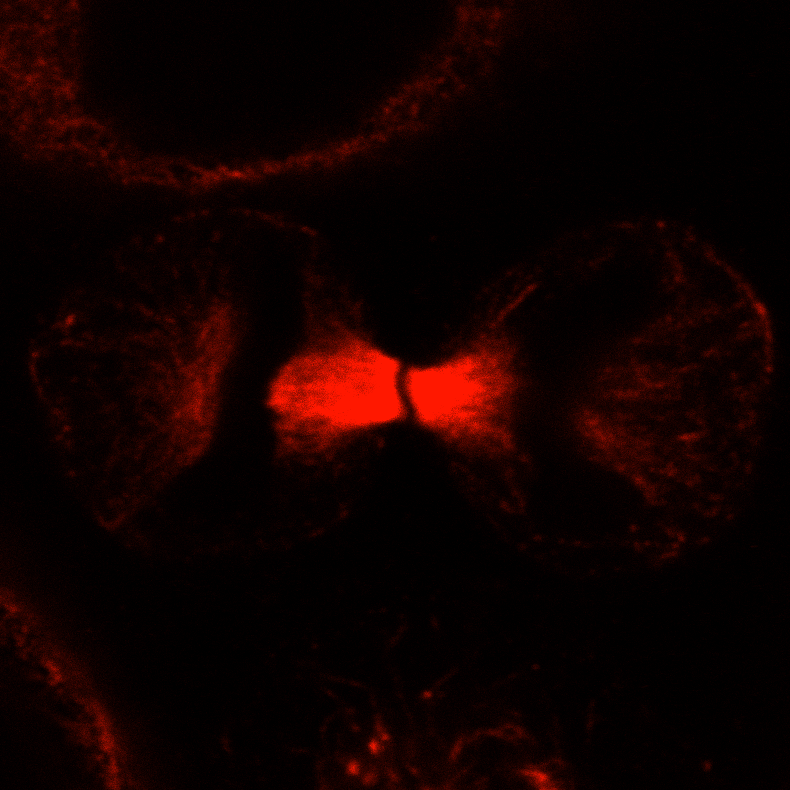

Supplement: Supplementary file 15 — Figure EV4 Source Data [file 44319_2026_815_MOESM15_ESM.zip › Figure EV4/4C/C2-PLVX L2E 100X1.7.png]

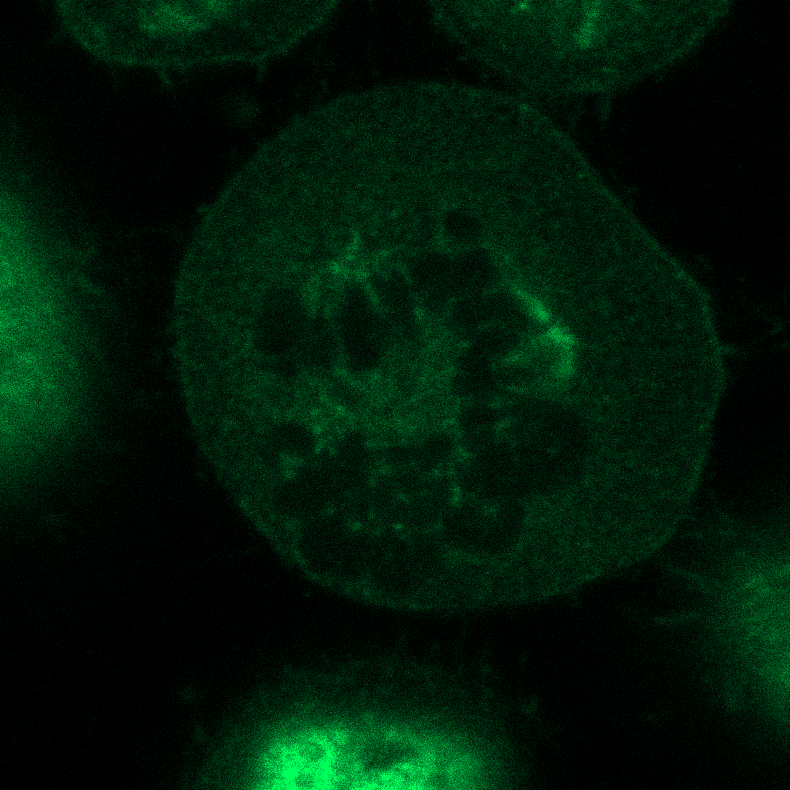

Supplement: Supplementary file 15 — Figure EV4 Source Data [file 44319_2026_815_MOESM15_ESM.zip › Figure EV4/4C/C4-PLVX L2E 100X1.7-2.png]

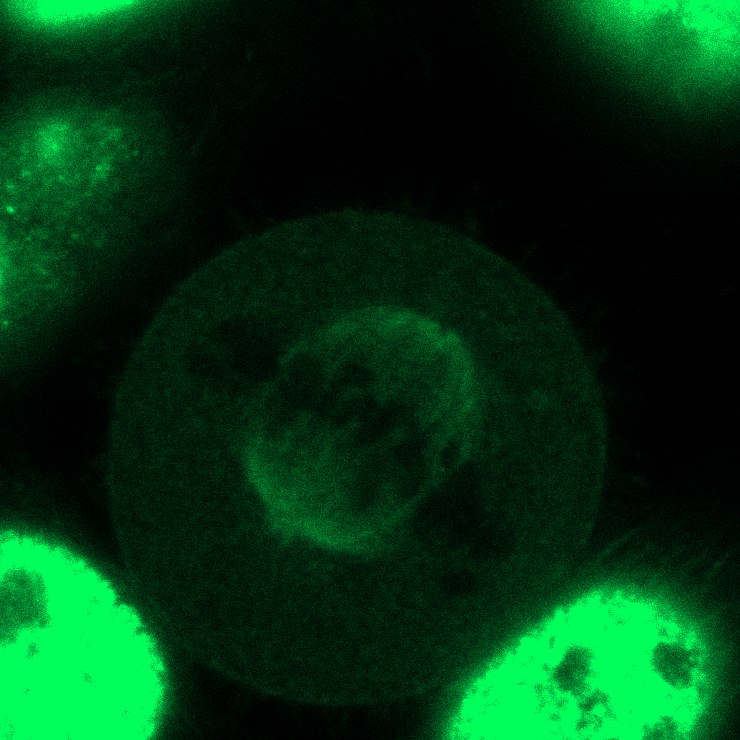

Supplement: Supplementary file 15 — Figure EV4 Source Data [file 44319_2026_815_MOESM15_ESM.zip › Figure EV4/4C/C4-PLVX L2E 100X1.7-3.png]

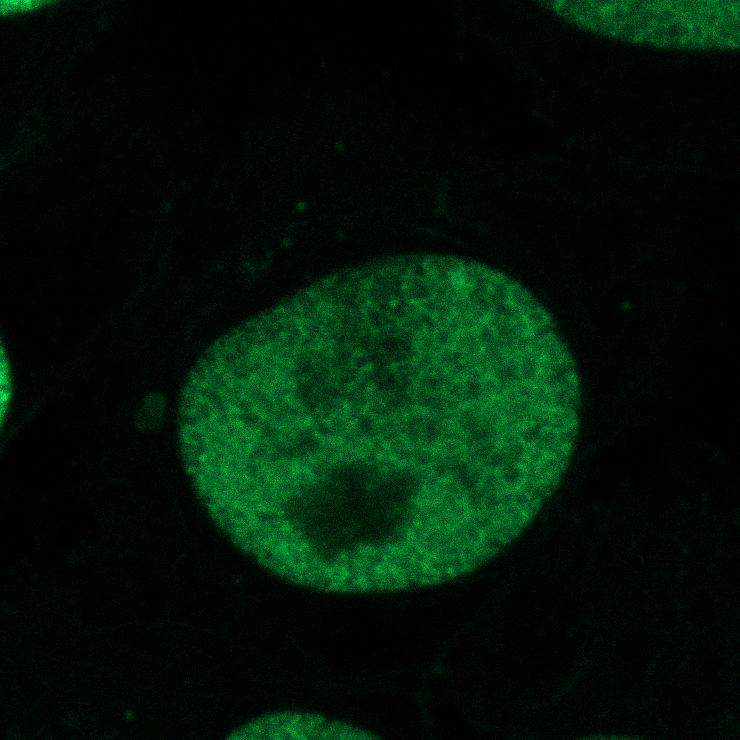

Supplement: Supplementary file 15 — Figure EV4 Source Data [file 44319_2026_815_MOESM15_ESM.zip › Figure EV4/4C/C4-PLVX L2E 100X1.7-4.png]

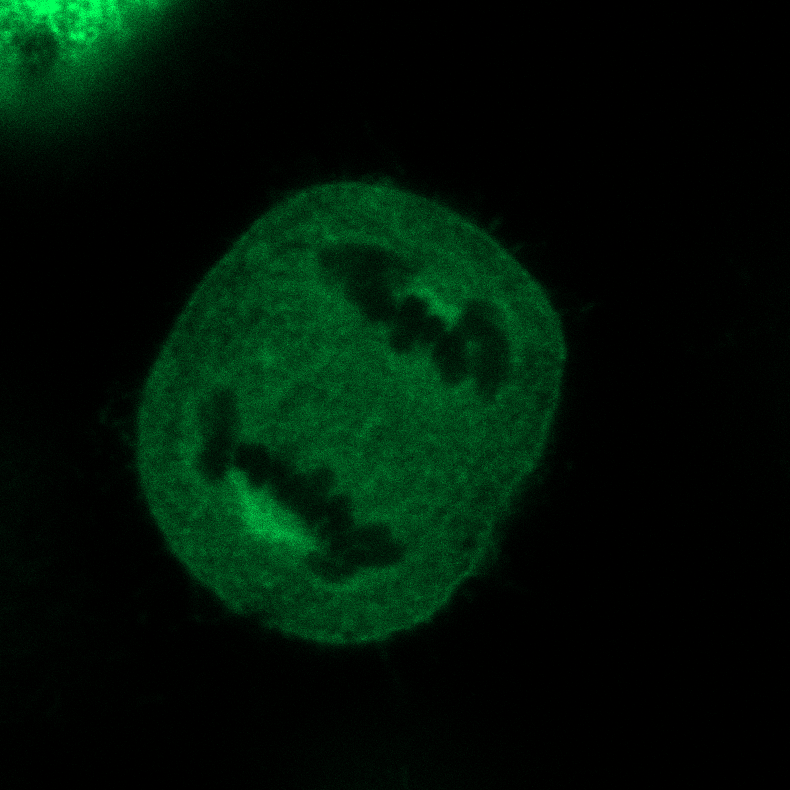

Supplement: Supplementary file 15 — Figure EV4 Source Data [file 44319_2026_815_MOESM15_ESM.zip › Figure EV4/4C/C4-PLVX L2E 100X1.7-5.png]

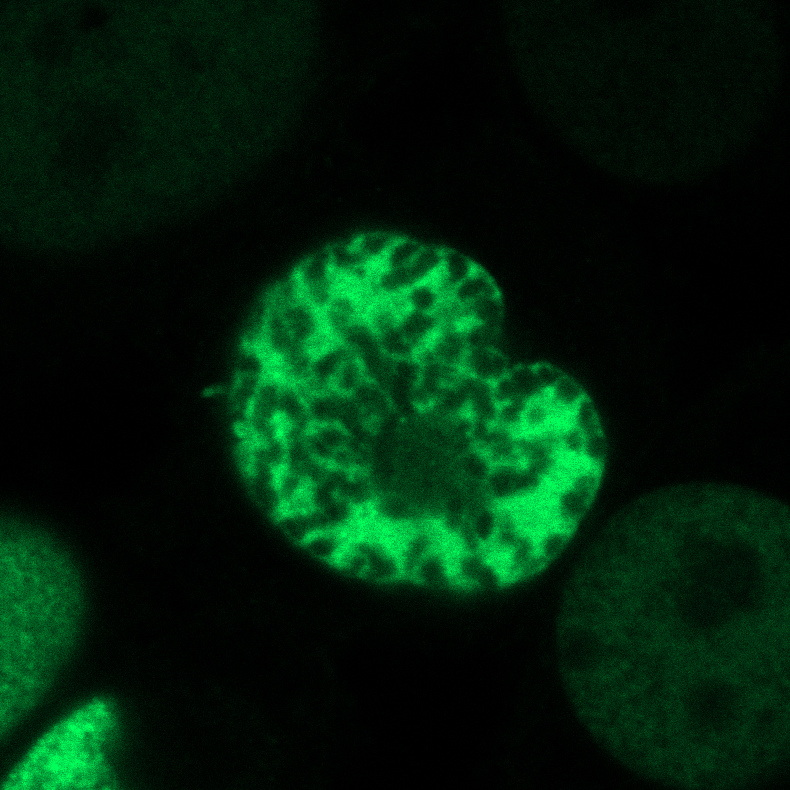

Supplement: Supplementary file 15 — Figure EV4 Source Data [file 44319_2026_815_MOESM15_ESM.zip › Figure EV4/4C/C4-PLVX L2E 100X1.7-7.png]

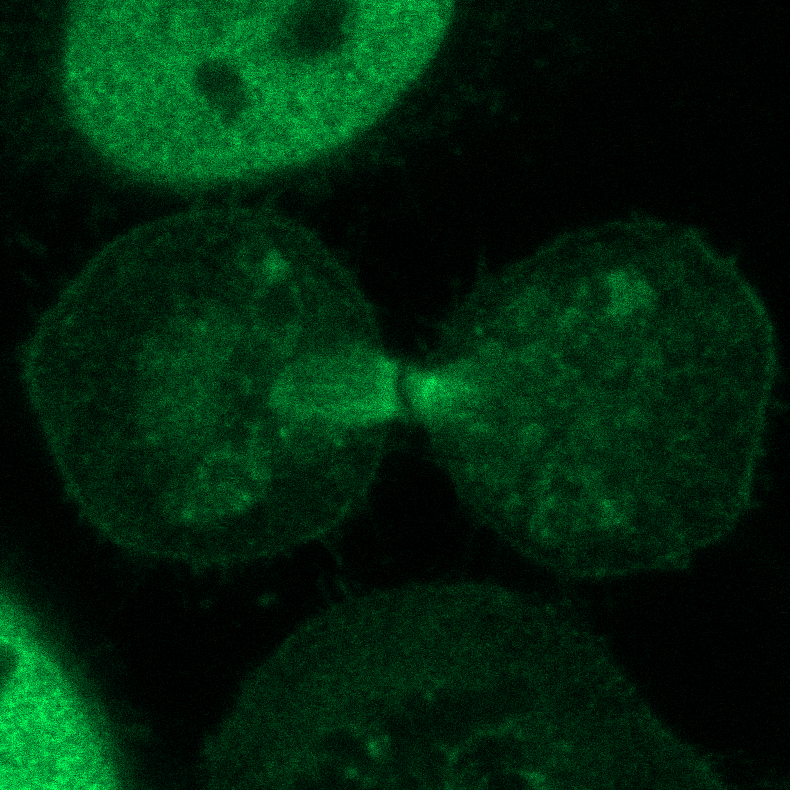

Supplement: Supplementary file 15 — Figure EV4 Source Data [file 44319_2026_815_MOESM15_ESM.zip › Figure EV4/4C/C4-PLVX L2E 100X1.7.png]

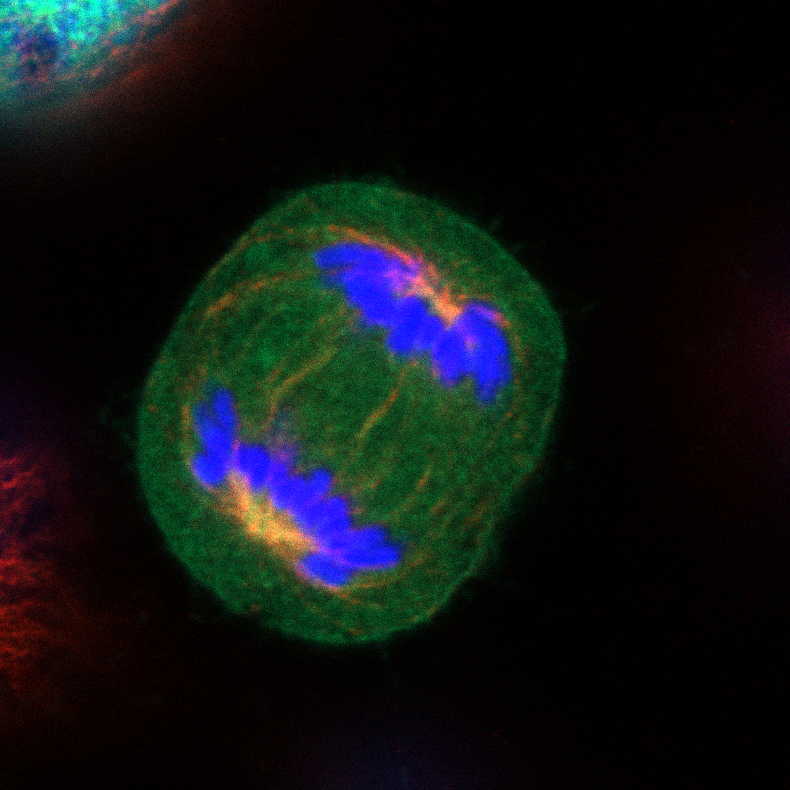

Supplement: Supplementary file 15 — Figure EV4 Source Data [file 44319_2026_815_MOESM15_ESM.zip › Figure EV4/4C/PLVX L2E-anaphase.png]

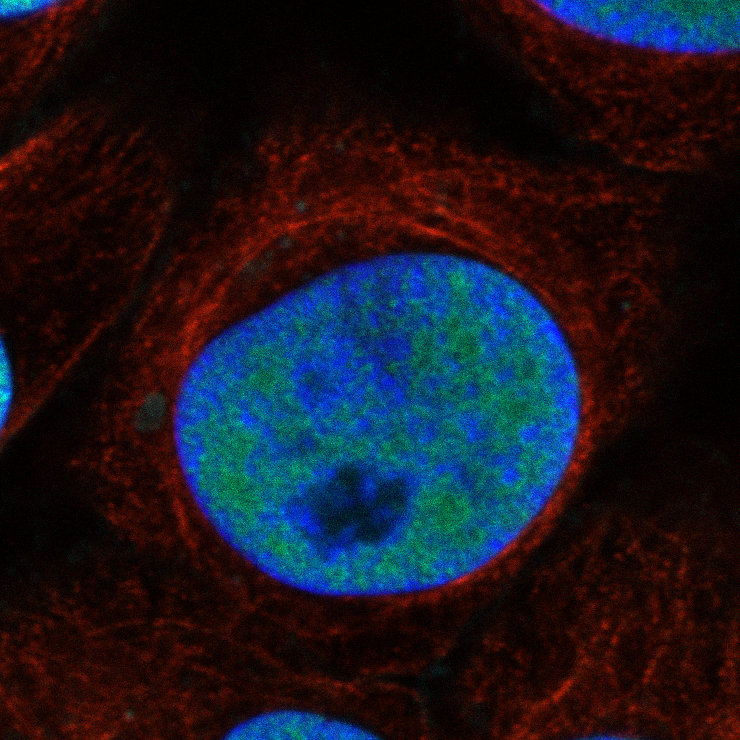

Supplement: Supplementary file 15 — Figure EV4 Source Data [file 44319_2026_815_MOESM15_ESM.zip › Figure EV4/4C/PLVX L2E-interphase.png]

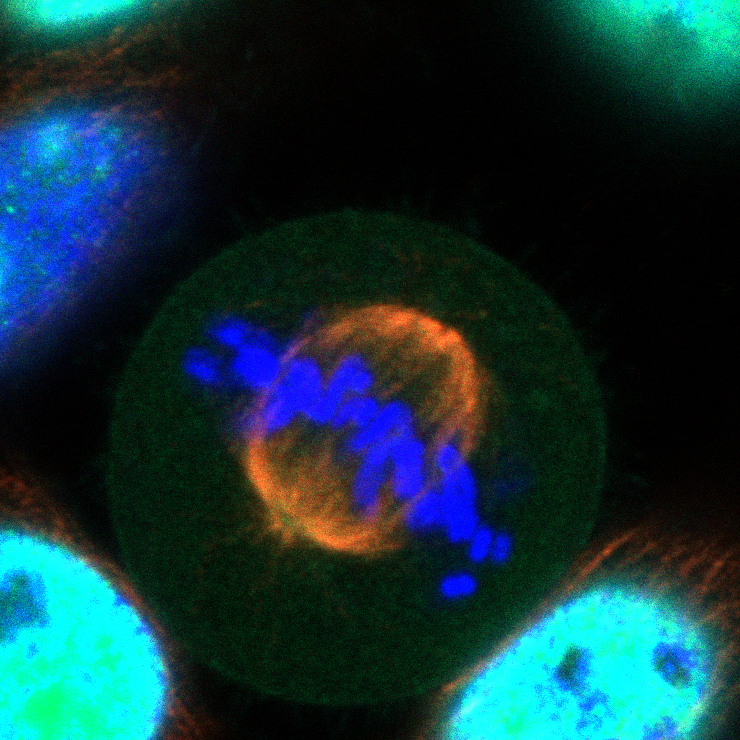

Supplement: Supplementary file 15 — Figure EV4 Source Data [file 44319_2026_815_MOESM15_ESM.zip › Figure EV4/4C/PLVX L2E-metaphase.png]

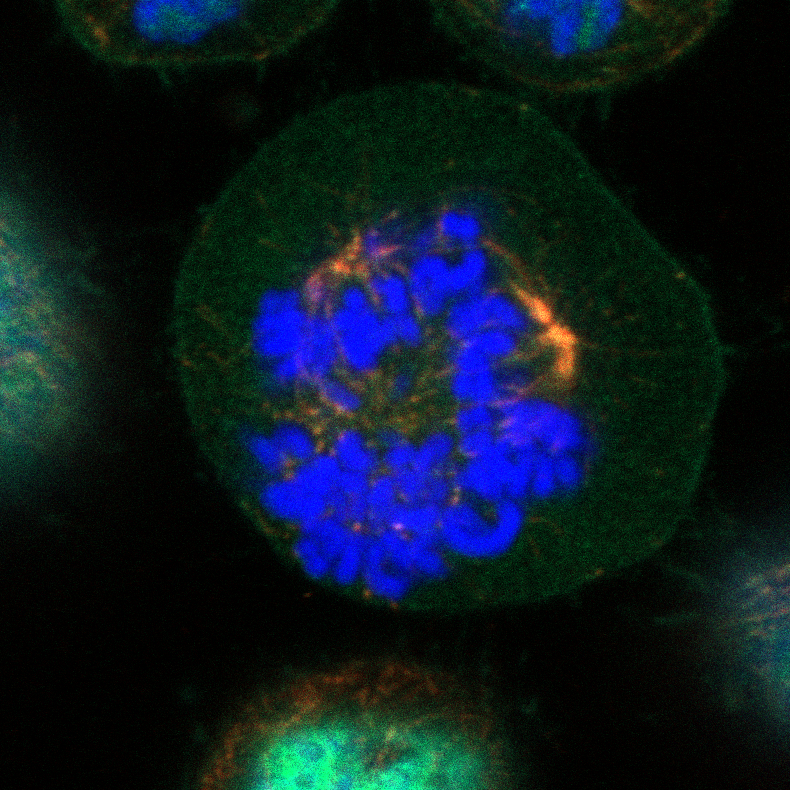

Supplement: Supplementary file 15 — Figure EV4 Source Data [file 44319_2026_815_MOESM15_ESM.zip › Figure EV4/4C/PLVX L2E-prometaphase.png]

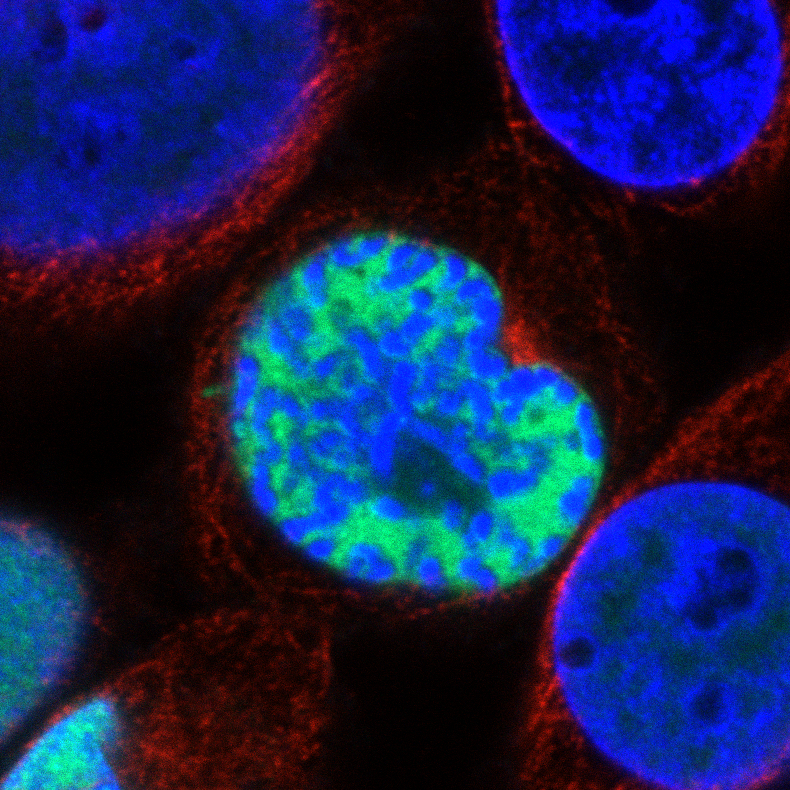

Supplement: Supplementary file 15 — Figure EV4 Source Data [file 44319_2026_815_MOESM15_ESM.zip › Figure EV4/4C/PLVX L2E-prophase.png]

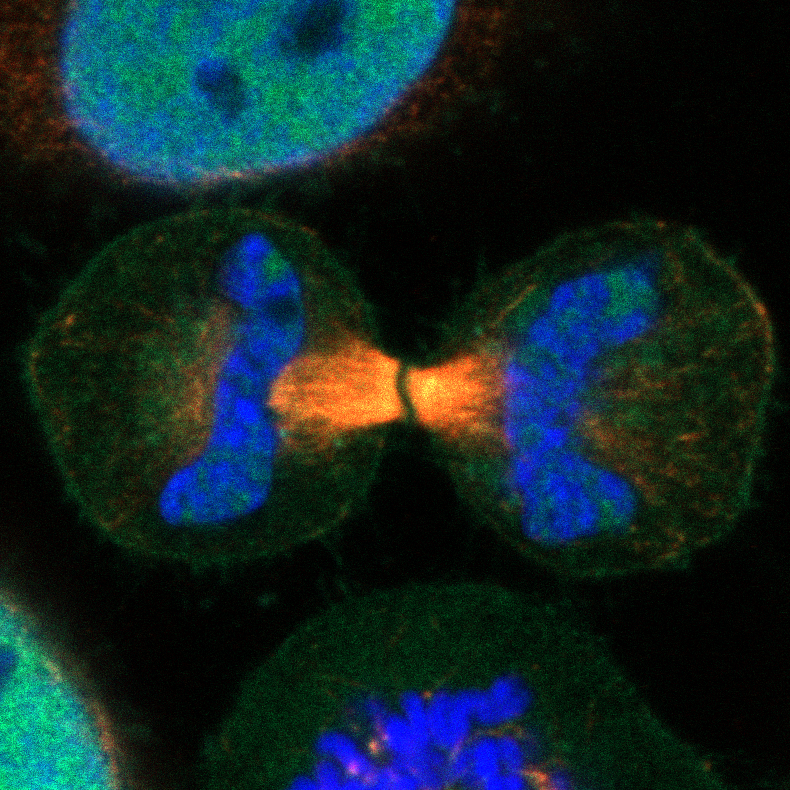

Supplement: Supplementary file 15 — Figure EV4 Source Data [file 44319_2026_815_MOESM15_ESM.zip › Figure EV4/4C/PLVX L2E-telophase.png]

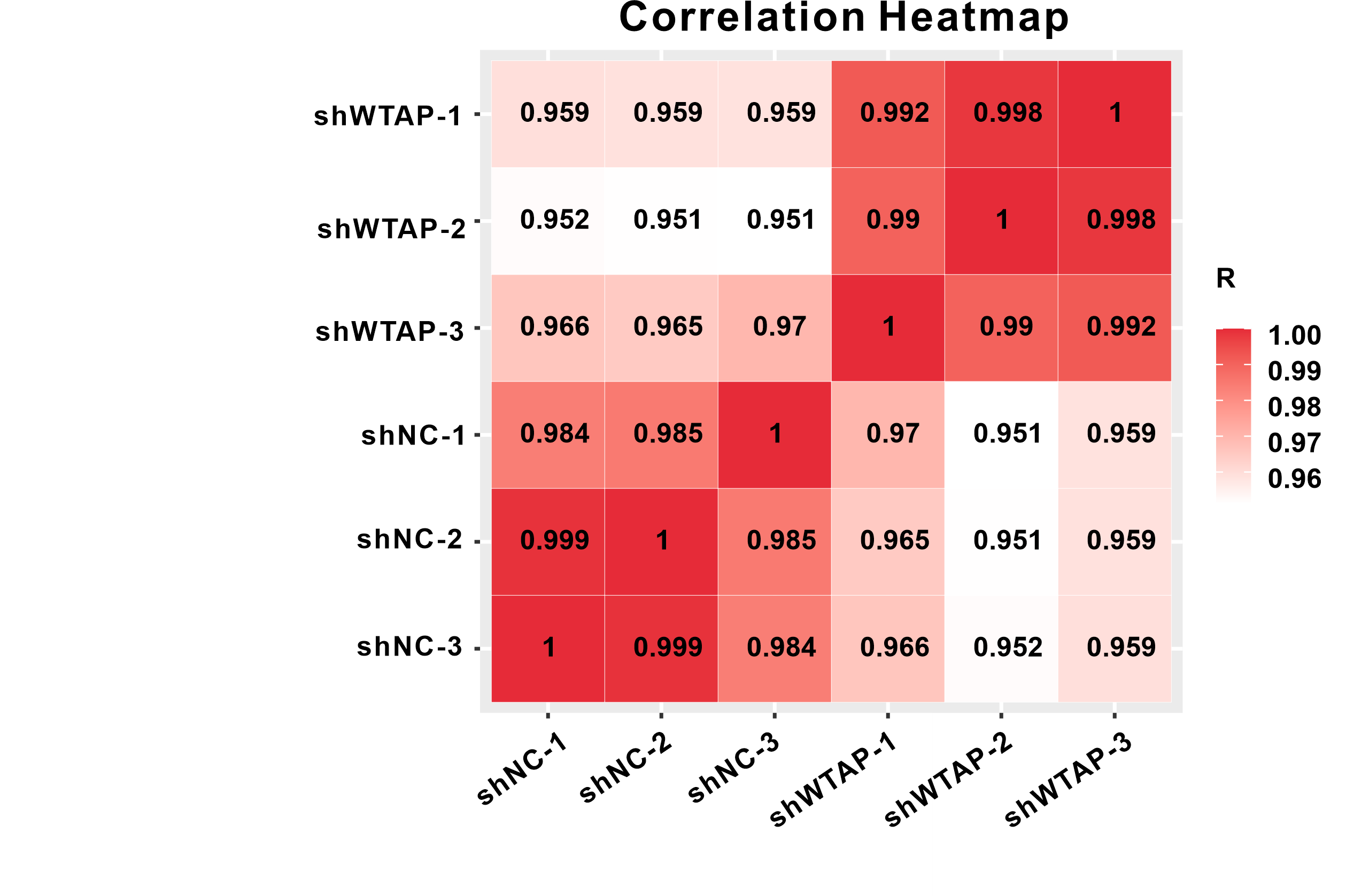

Supplement: Supplementary file 16 — Figure EV5 Source Data [file 44319_2026_815_MOESM16_ESM.zip › Figure EV5/5A/Pearson correlation coefficients.tif]

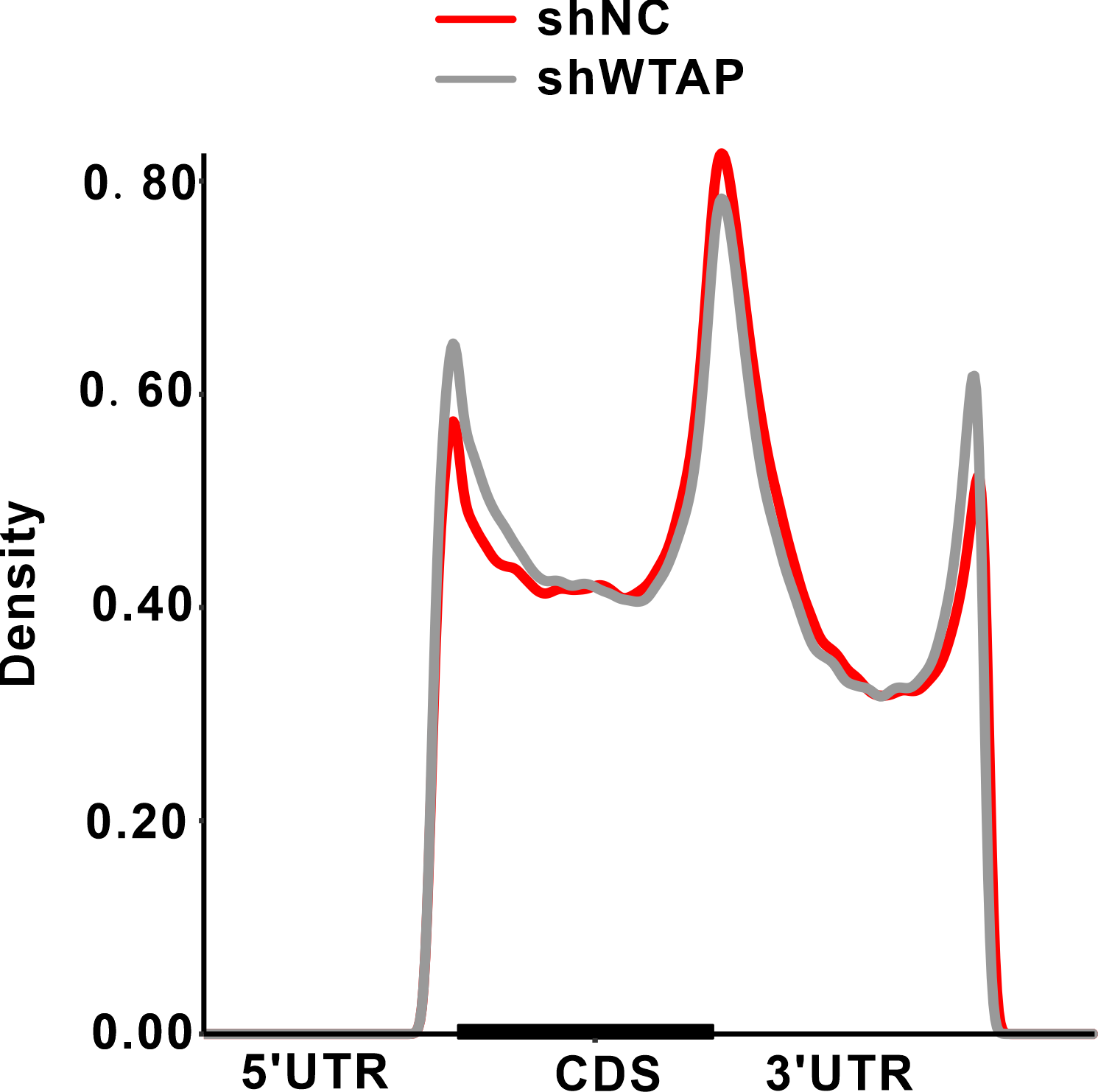

Supplement: Supplementary file 16 — Figure EV5 Source Data [file 44319_2026_815_MOESM16_ESM.zip › Figure EV5/5B/peak density.tif]

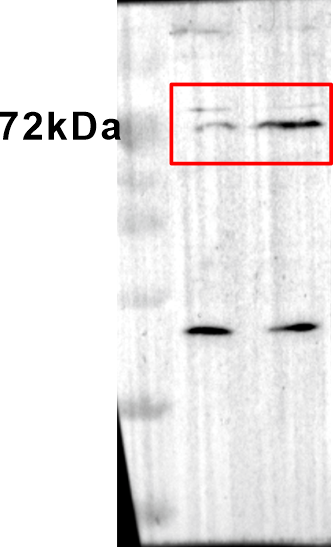

Supplement: Supplementary file 16 — Figure EV5 Source Data [file 44319_2026_815_MOESM16_ESM.zip › Figure EV5/5D/DYRK3.tif]

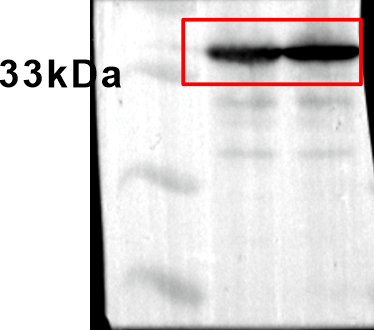

Supplement: Supplementary file 16 — Figure EV5 Source Data [file 44319_2026_815_MOESM16_ESM.zip › Figure EV5/5D/GAPDH.tif]

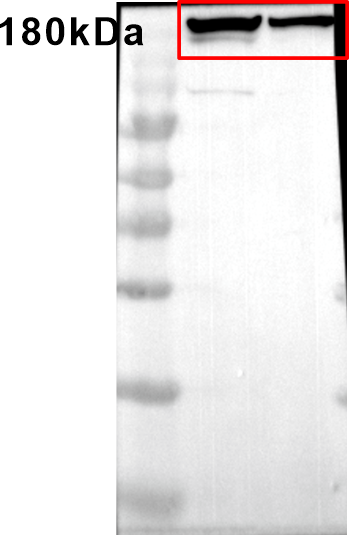

Supplement: Supplementary file 16 — Figure EV5 Source Data [file 44319_2026_815_MOESM16_ESM.zip › Figure EV5/5D/IQGAP1.tif]

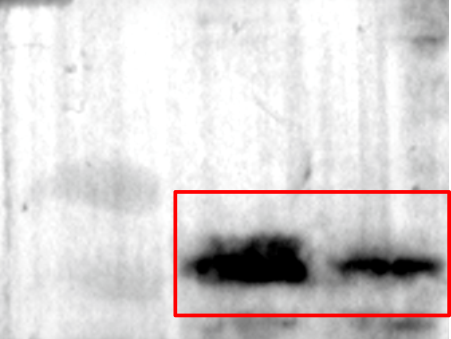

Supplement: Supplementary file 16 — Figure EV5 Source Data [file 44319_2026_815_MOESM16_ESM.zip › Figure EV5/5D/WTAP.tif]
