# Supplementary material for: WTAP tetramer ensures m6A writer assembly and faithful mitosis
Source: EMBO Rep. 2026 Jun 2;27(13):3842–62. doi: 10.1038/s44319-026-00815-3 (PMC13354555; doi:10.1038/s44319-026-00815-3)
Supplement: Supplementary file 17 — Expanded View Figures [file 44319_2026_815_MOESM17_ESM.pdf]

Expanded View Figures

Figure EV1. Biochemical analysis of full-length and truncated WTAP constructs.

(**A**) SDS-PAGE analysis of purified full-length WTAP and truncated fragments (F1-F4), followed by Coomassie blue staining. (**B**–**F**) Mass photometry analysis of purified full-length WTAP (**B**) and truncated fragments F1 (**C**), F2 (**D**), F3 (**E**), and F4 (**F**). All measurements were performed at a protein concentration of 0.2 µM. The molecular masses of the detected species are indicated. Source data are available online for this figure.

© The Author(s)

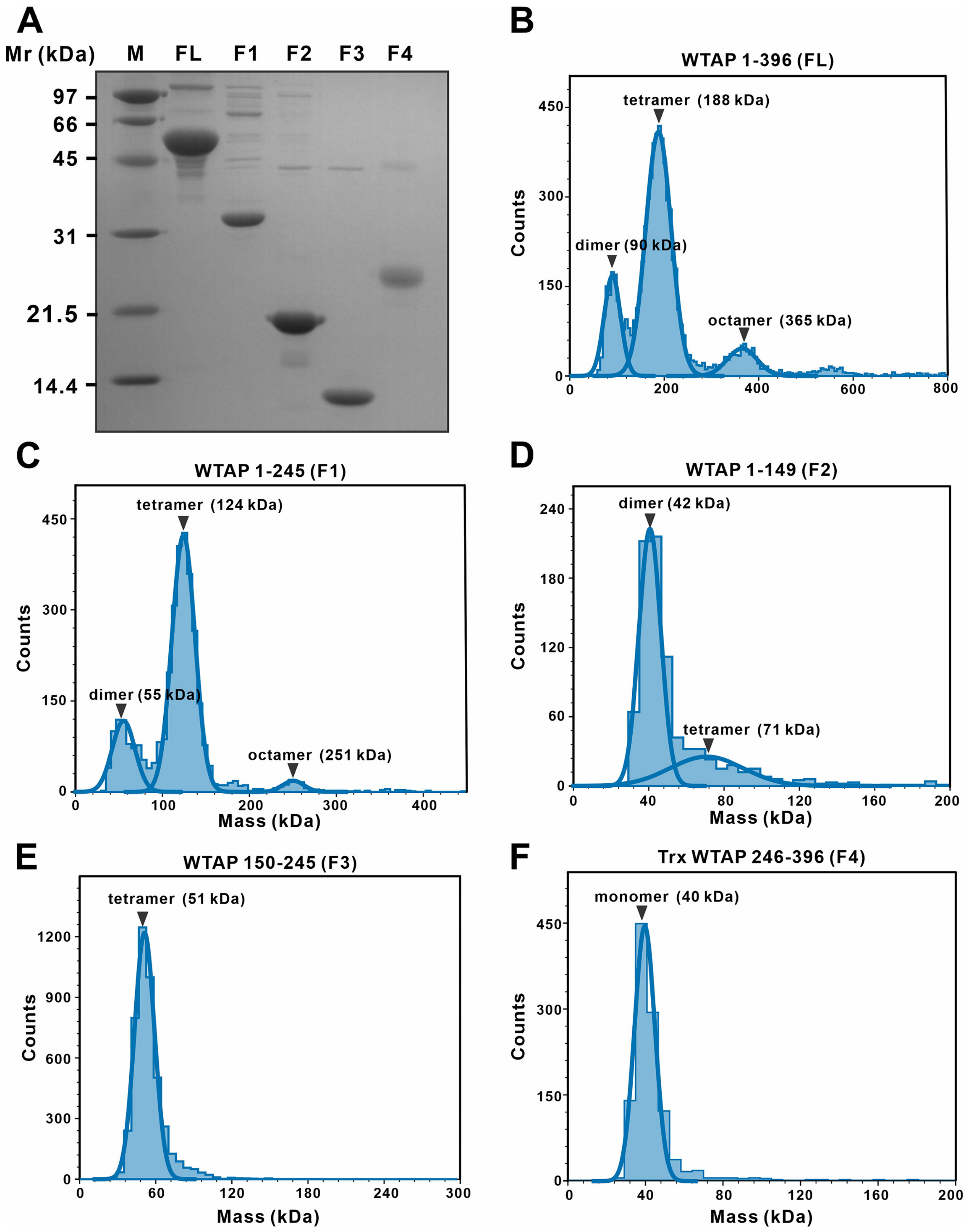
A
Mr (kDa) M FL F1 F2 F3 F4
97
66
45
31
21.5
14.4
B
WTAP 1-396 (FL)
tetramer (188 kDa)
dimer (90 kDa)
octamer (365 kDa)
Counts
0 200 400 600 800
0 150 300 450
C
WTAP 1-245 (F1)
tetramer (124 kDa)
dimer (55 kDa)
octamer (251 kDa)
Counts
Mass (kDa)
0 100 200 300 400
0 150 300 450
D
WTAP 1-149 (F2)
dimer (42 kDa)
tetramer (71 kDa)
Counts
Mass (kDa)
0 40 80 120 160 200
0 60 120 180 240
E
WTAP 150-245 (F3)
tetramer (51 kDa)
Counts
Mass (kDa)
0 60 120 180 240 300
0 300 600 900 1200
F
Trx WTAP 246-396 (F4)
monomer (40 kDa)
Counts
Mass (kDa)
0 40 80 120 160 200
0 150 300 450

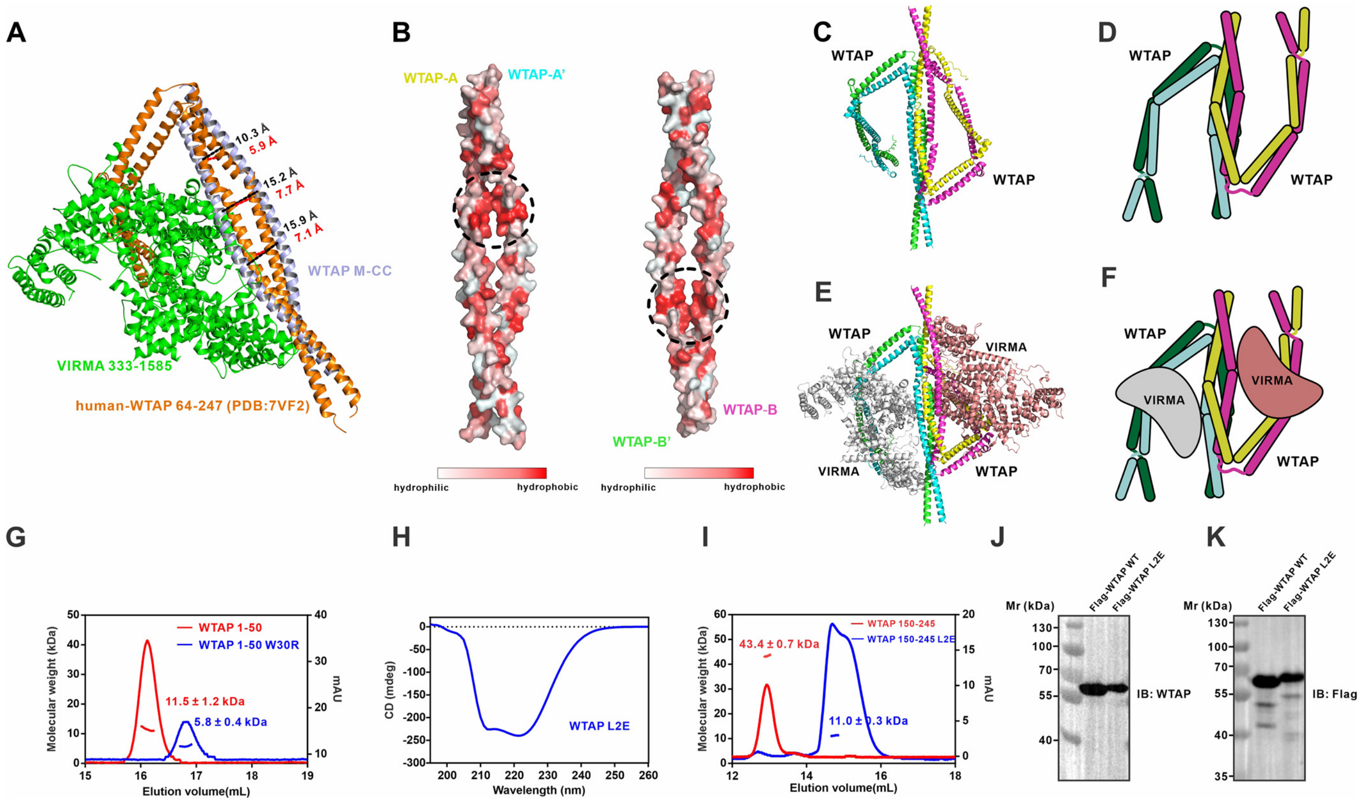

Figure EV2. Structure-guided mutagenesis of WTAP disrupts oligomeric assembly while preserving secondary structure.

(**A**) Superimposition of the WTAP M-CC crystal structure from this study (cyan) with the previously reported WTAP cryo-EM structure (PDB: 7VF2, orange) (Su et al, 2022). Distances between helices within the coiled-coil domain are indicated. (**B**) Hydrophobic interfaces between α-helices: left, WTAP-A and WTAP-A′; right, WTAP-B and WTAP-B′. Hydrophobic regions are highlighted in red. (**C**, **D**) Cartoon representation (**C**) and schematic diagram (**D**) of WTAP (residues 1–245), showing hierarchical assembly from dimer to tetramer via the central coiled-coil domain. (**E**, **F**) Cartoon representation (**E**) and schematic diagram (**F**) of WTAP (residues 1–245) in complex with VIRMA. (**G**) FPLC-MALS analysis of WTAP-N W30R mutant showing loss of dimerization, confirming disruption of the N-terminal hydrophobic interface. (**H**) Circular dichroism (CD) spectrum of the WTAP L2E mutant, showing retention of α-helical secondary structure despite loss of tetramerization. (**I**) FPLC-MALS analysis of WTAP M-CC (residues 150–245) L2E mutant, showing monomerization of the middle coiled-coil domain. (**J**, **K**) Immunoblot analysis of HEK293T cells expressing Flag-WTAP WT or L2E mutant. WTAP was detected using anti-WTAP (**J**) and anti-Flag (**K**) antibodies. Source data are available online for this figure.

 © The Author(s)

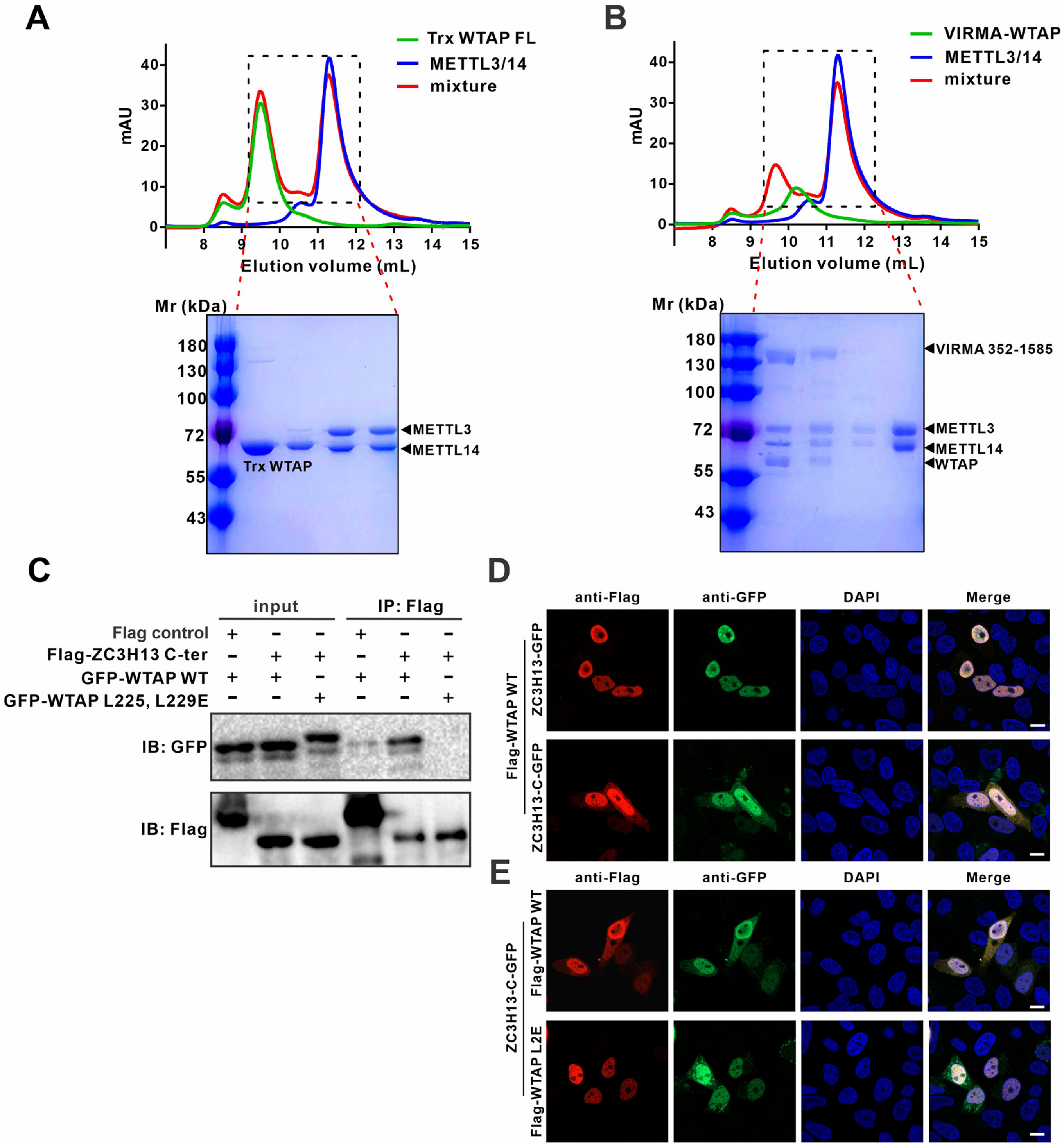

Figure EV3. Coiled-coil tetramerization of WTAP is required for interaction with ZC3H13.

(**A**) FPLC analysis (top) and SDS-PAGE analysis (bottom) of WTAP and METTL3/14. Distinct elution profiles and absence of co-elution indicate that WTAP does not directly interact with METTL3/14. (**B**) FPLC analysis (top) and SDS-PAGE analysis (bottom) of the WTAP-VIRMA complex and METTL3/14. Co-elution of WTAP, VIRMA, and METTL3/14 indicates that WTAP associates with METTL3/14 in a VIRMA-dependent manner. (**C**) Co-immunoprecipitation assays in HEK293T cells co-expressing Flag-tagged ZC3H13 C-terminal domain (residues 1460–1669) and GFP-tagged WTAP variants (WT or L2E). WTAP L2E fails to interact with ZC3H13. (**D**) Immunofluorescence imaging of HeLa cells co-expressing Flag-WTAP (red, Alexa Fluor 568) and ZC3H13-GFP (full-length or C-terminal). Full-length ZC3H13 co-localizes with WTAP in nuclear speckles, whereas the C-terminal fragment partially relocalizes WTAP to the cytoplasm. Nuclei were stained with DAPI. Scale bar: 10 µm. (**E**) HeLa cells co-expressing Flag-WTAP (WT or L2E) with ZC3H13-C-GFP. Compared to WTAP WT, the L2E mutant fails to colocalize with the ZC3H13 C-terminal fragment. Scale bar: 10 µm. Source data are available online for this figure.

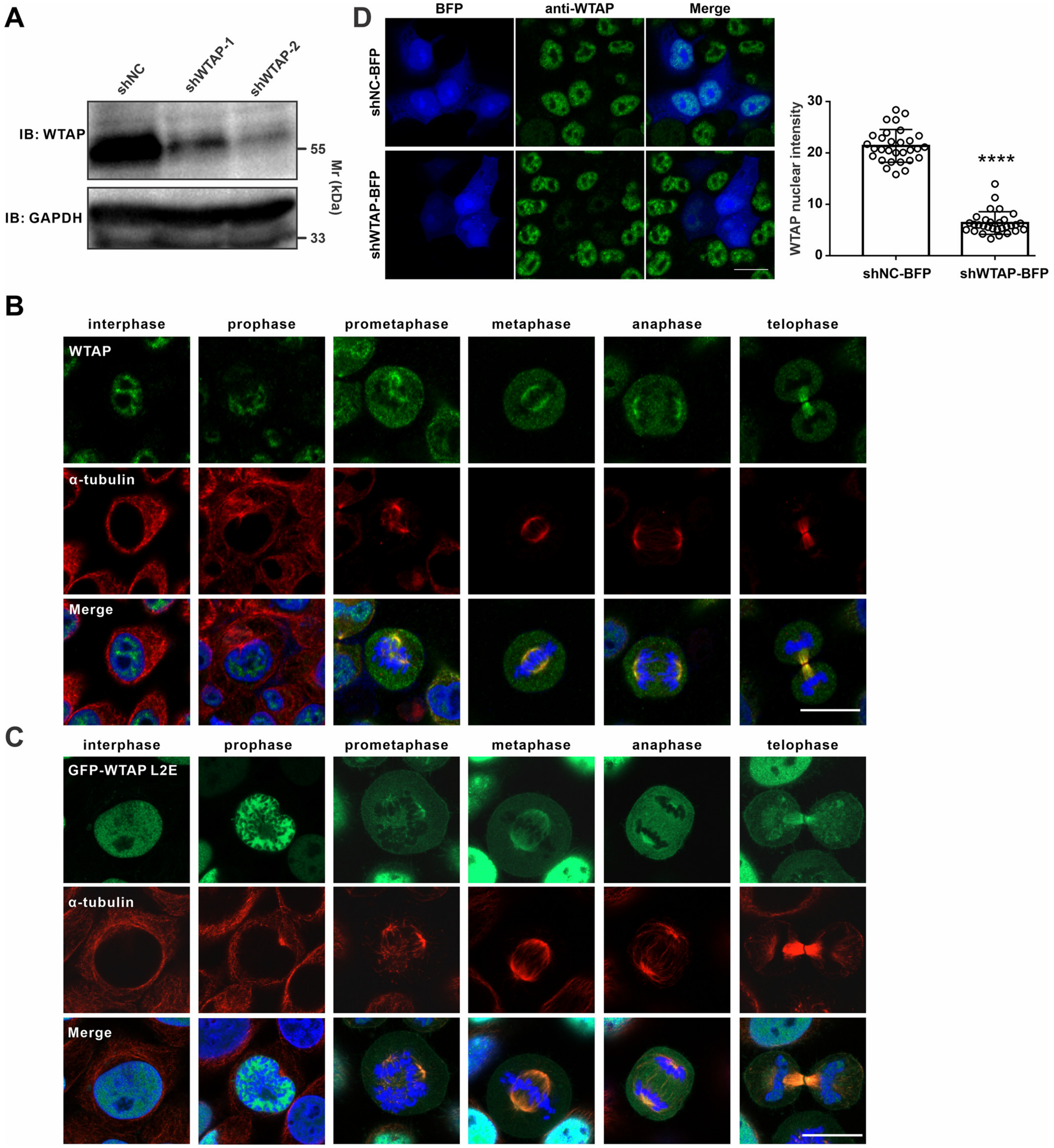

Figure EV4. WTAP knockdown efficiency and dynamic mitotic localization.

(**A**) Immunoblot analysis of WTAP knockdown in HeLa cells using two independent shRNAs. GAPDH serves as a loading control. (**B**) Immunofluorescence imaging of endogenous WTAP (green) and α-tubulin (red) across mitotic stages. WTAP redistributes from the nucleus to mitotic spindles upon mitotic entry. Scale bar: 20 μm. (**C**) Immunofluorescence imaging of HeLa cells stably expressing GFP-tagged WTAP L2E at different mitotic stages. Cells were stained with α-tubulin to visualize the spindle. Scale bar: 20 μm. (**D**) Fluorescence imaging of HeLa cells transfected with BFP-marked shWTAP or shNC (left). The right panel shows quantification of WTAP fluorescence intensity. Data are presented as mean ± SD ($n = 30$). Statistical significance was determined using a two-tailed unpaired Student's *t* test: *****P* < 0.0001. Scale bar: 20 μm. Source data are available online for this figure.

 © The Author(s)

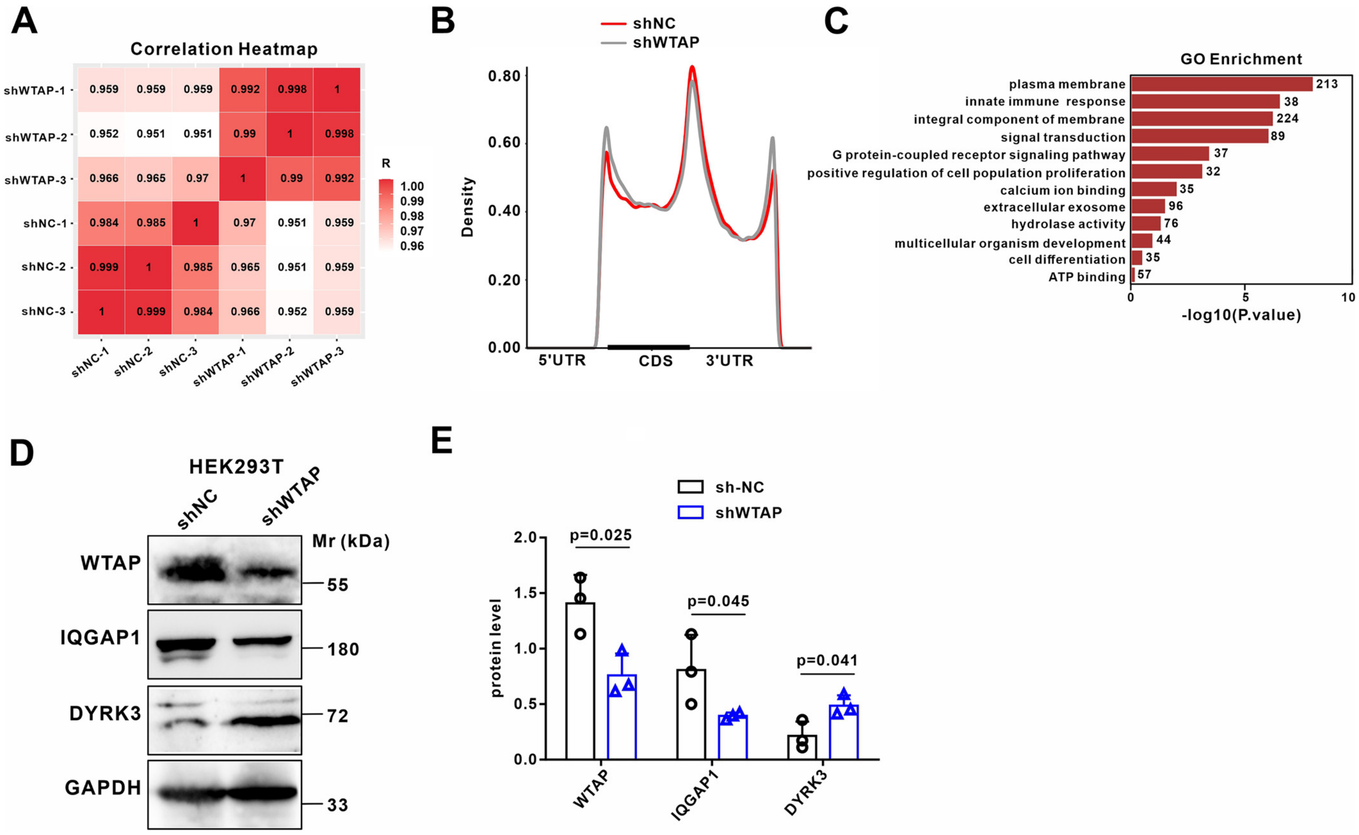

Figure EV5. WTAP-dependent m^{6}A regulation of mitotic transcripts.

(**A**) Heatmap showing Pearson correlation coefficients (R) between samples, indicating reproducibility of biological replicates. (**B**) Metagene analysis showing distribution of m^{6}A peaks across mRNA regions in WTAP knockdown (gray) and control (red) cells based on MeRIP-seq. (**C**) Gene Ontology (GO) enrichment analysis of differentially expressed genes from RNA-seq, highlighting pathways related to cell signaling and mitotic progression. Statistical significance was assessed using a one-sided hypergeometric test with Benjamini–Hochberg FDR correction. (**D**) Immunoblot validation of protein expression changes (IQGAP1and DYRK3) upon WTAP knockdown in HEK293T cells. (**E**) Quantification of protein expression changes from (**D**). Data are presented as mean ± SD from three biological replicates. Band intensities were normalized to GAPDH. Statistical significance was determined using a two-tailed unpaired Student's *t* test. Source data are available online for this figure.
