# Supplementary material for: WTAP tetramer ensures m6A writer assembly and faithful mitosis
Source: EMBO Rep. 2026 Jun 2;27(13):3842–62. doi: 10.1038/s44319-026-00815-3 (PMC13354555; doi:10.1038/s44319-026-00815-3)
Supplement: Supplementary file 4 — Peer Review File [file 44319_2026_815_MOESM4_ESM.pdf]

EMBO reports

WTAP tetramer ensures m^6A writer assembly and faithful mitosis

Shan Zhang, Jin Ye, Manjuan Zhang, Jie Cao, Yujuan Li, Ranran Hu, Da Chen, Ting Li, Jiasheng Chen, Wen Zhu, Wenli Jiang, Jianchao LI, Wei Hu, Jianzhao Liu, Xing Liu, and Chao WANG

Corresponding author(s): Chao WANG (cwangust@ustc.edu.cn) , Jianzhao Liu (liujz@zju.edu.cn), Xing Liu (xing1017@ustc.edu.cn), Wei Hu (andinghu@ustc.edu.cn)

Review Timeline:

Submission Date:	17th Nov 25
Editorial Decision:	18th Dec 25
Revision Received:	24th Mar 26
Editorial Decision:	11th May 26
Revision Received:	13th May 26
Accepted:	21st May 26

Editor: Esther Schnapp

Transaction Report:

(Note: With the exception of the correction of typographical or spelling errors that could be a source of ambiguity, letters and reports are not edited. Depending on transfer agreements, referee reports obtained elsewhere may or may not be included in this compilation. Referee reports are anonymous unless the Referee chooses to sign their reports.)

1st Editorial Decision **18th Dec 2025**

Dear Dr. WANG,

Thank you for the submission of your manuscript to EMBO reports. We have now received the enclosed comments from 2 referees and given that both reports are very good, I am making a decision now in order to save time.

As you will see, the referees acknowledge that the findings are interesting and novel. However, they also have several suggestions for how the study could be improved and strengthened, and I think all suggestions are good and should be addressed. Please let me know in case you disagree and we can discuss the exact revision requirements further, also in a video chat, if you like.

I would thus like to invite you to revise your manuscript with the understanding that the referee concerns must be fully addressed and their suggestions taken on board. Please address all referee concerns in a complete point-by-point response. Acceptance of the manuscript will depend on a positive outcome of a second round of review. It is EMBO reports policy to allow a single round of major revision only and acceptance or rejection of the manuscript will therefore depend on the completeness of your responses included in the next, final version of the manuscript.

We realize that it is difficult to revise to a specific deadline. In the interest of protecting the conceptual advance provided by the work, we recommend a revision within 3 months (20th Mar 2026). Please discuss the revision progress ahead of this time with the editor if you require more time to complete the revisions.

You can either publish the study as a short report or as a full article. For short reports, the revised manuscript should not exceed 29,000 characters (including spaces but excluding materials & methods and references) and 5 main plus 5 expanded view figures. The results and discussion sections must further be combined, which will help to shorten the manuscript text by eliminating some redundancy that is inevitable when discussing the same experiments twice. For a normal article there are no length limitations, but it should have more than 5 main figures and the results and discussion sections must be separate. In both cases, the entire materials and methods must be included in the main manuscript file.

IMPORTANT NOTE: we perform an initial quality control of all revised manuscripts before re-review. Your manuscript will FAIL this control and the handling will be DELAYED if the following APPLIES:
1) A data availability section providing access to data deposited in public databases is missing. If you have not deposited any data, please add a sentence to the data availability section that explains that.
2) Your manuscript contains statistics and error bars based on n=2. Please use scatter blots in these cases. No statistics should be calculated if n=2.

When submitting your revised manuscript, please review the submission guidelines (https://link.springer.com/journal/44319/submission-guidelines#cms-Revised-submissions) and carefully review the instructions that follow below. Failure to include requested items will delay the evaluation of your revision.

1) a .docx formatted version of the manuscript text (including legends for main figures, EV figures and tables). Please make sure that the changes are highlighted to be clearly visible.

2) individual production quality figure files as .eps, .tif, .jpg (one file per figure). See hhttps://media.springernature.com/original/springer-cms/rest/v1/content/27825798/data/v1 for more info on how to prepare your figures.

3) We replaced Supplementary Information with Expanded View (EV) Figures and Tables that are collapsible/expandable online. A maximum of 5 EV Figures can be typeset. EV Figures should be cited as 'Figure EV1, Figure EV2" etc... in the text and their respective legends should be included in the main text after the legends of regular figures.

- For the figures that you do NOT wish to display as Expanded View figures, they should be bundled together with their legends in a single PDF file called *Appendix*, which should start with a short Table of Content. Appendix figures should be referred to in the main text as: "Appendix Figure S1, Appendix Figure S2" etc. See 'Expanded View' section of the submission guidelines.

- Additional Tables/Datasets should be labeled and referred to as Table EV1, Dataset EV1, etc. Legends have to be provided in a separate tab in case of .xls files. Alternatively, the legend can be supplied as a separate text file (README) and zipped together with the Table/Dataset file.

4) a .docx formatted letter INCLUDING the reviewers' reports and your detailed point-by-point responses to their comments. As part of the EMBO Press transparent editorial process, the point-by-point response is part of the Review Process File (RPF), which will be published alongside your paper.

5) a complete author checklist, which you can download from our author guidelines. Please insert information in the checklist

that is also reflected in the manuscript. The completed author checklist will also be part of the RPF.

6) Please note that all corresponding authors are required to supply an ORCID ID for their name upon submission of a revised manuscript (<https://orcid.org/>), which can be linked to your profile in the manuscript submission system.

7) Before submitting your revision, primary datasets produced in this study need to be deposited in an appropriate public database. Please remember to provide a reviewer password if the datasets are not yet public. The accession numbers and database should be listed in a formal "Data Availability" section placed after Methods. Please note that the Data Availability Section is restricted to new primary data that are part of this study. * Note - All links should resolve to a page where the data can be accessed. *

If your study has not produced novel datasets, please mention this fact in the Data Availability Section.

8) At EMBO Press we ask authors to provide source data for the main manuscript figures. You will receive a separate email with instructions for providing source data with your revised manuscript, including how to upload and organize the files.

9) Our journal also encourages inclusion of *data citations in the reference list* to directly cite datasets that were re-used and obtained from public databases. Data citations in the article text are distinct from normal bibliographical citations and should directly link to the database records from which the data can be accessed. In the main text, data citations are formatted as follows: "Data ref: Smith et al, 2001" or "Data ref: NCBI Sequence Read Archive PRJNA342805, 2017". In the Reference list, data citations must be labeled with "[DATASET]". A data reference must provide the database name, accession number/identifiers and a resolvable link to the landing page from which the data can be accessed at the end of the reference.

10) Regarding data quantification, the following points must be specified in each figure legend:

- the name of the statistical test used to generate error bars and P values,

- the number (n) of independent experiments (please specify technical or biological replicates) underlying each data point,

- the nature of the bars and error bars (s.d., s.e.m.),

- If the data are obtained from n Program fragment delivered error ``Can't locate object method "less" via package "than" (perhaps you forgot to load "than"?) at //ejpvfs23/sites23b/embor_www/letters/embor_decision_revise_and_review.txt line 54.' 2, use scatter blots showing the individual data points.

Discussion of statistical methodology can be reported in the materials and methods section, but figure legends should contain a basic description of n, P and the test applied.

- Please also include scale bars in all microscopy images.

11) The journal requires a statement specifying whether or not authors have competing interests (defined as all potential or actual interests that could be perceived to influence the presentation or interpretation of an article). In case of competing interests, this must be specified in your disclosure statement.

12) All Materials and Methods need to be described in the main text using our 'Structured Methods' format, which is required for all research articles. According to this format, the Methods section includes a separate Reagents and Tools Table file (listing key reagents, experimental models, software and relevant equipment and including their sources and relevant identifiers) and a Methods and Protocols section describing the methods using a step-by-step protocol format. The aim is to facilitate adoption of the methodologies across labs. More information on how to adhere to this format as well as a downloadable template (.docx) for the Reagents and Tools Table can be found in our author guidelines.

We would also welcome the submission of cover suggestions, or motifs to be used by our Graphics Illustrator in designing a cover.

As part of the EMBO publication's Transparent Editorial Process, EMBO reports publishes online a Review Process File (RPF) to accompany accepted manuscripts. This File will be published in conjunction with your paper and will include the referee reports, your point-by-point response and all pertinent correspondence relating to the manuscript.

You are able to opt out of this by letting the editorial office know (emboreports@embo.org). If you do opt out, the Review Process File link will point to the following statement: "No Review Process File is available with this article, as the authors have chosen not to make the review process public in this case."

I look forward to seeing a revised form of your manuscript when it is ready.

Best regards,
Esther

Esther Schnapp, PhD
Senior Editor
EMBO reports

Referee #1:

In this study, Zhang et al. investigate the structural organization of WTAP and its role in assembling the m6A writer complex. Using a combination of circular dichroism, FPLC-MALS, and X-ray crystallography techniques, the authors demonstrate that a fragment of WTAP protein forms a tetramer through a central coiled-coil domain. Disrupting this tetramer WTAP fragment impairs the binding of WTAP with METTL3. The authors further show that tetramer-disrupting mutations decrease global m6A levels, fail to rescue proliferation defects in WTAP-depleted cells, and lead to chromosome segregation errors during mitosis. Together, these results suggest that the tetrameric WTAP scaffold is required for the writer complex assembly and expression of mitosis-related genes.

This manuscript strongly highlights that tetrameric nature of WTAP, which was not well-stated in the prior cryoEM. Also, the study makes the intruiging insight that METTL3 binding depends on acquiring the dimer-of-dimer conformation. This could be a major regulatory step. Thus, this study has important insights for how m6A could be regulated, although these conformations are not shown to be dynamically regulated in cells. Regardless, these new insights are drivers for publication in this journal.

While this study explores a novel connection between WTAP oligomerization, with writer complex assembly and m6A-dependent cell-cycle regulation, a few methodological and conceptual issues require further clarification:

1. WTAP is composed of an N-terminal region, a central coiled-coil domain, and a C-terminal segment (Schöller et al., RNA, 2018). An earlier cryo-EM study demonstrated that WTAP forms a saddle-shaped structure consisting of four a-helices (H1-H4) arranged as a homodimer stabilized by coiled-coil interactions. The H3-H4 helices of WTAP bind the METTL3/METTL14 heterodimer (Su et al., Cell Res., 2022). In the present study, Zhang et al. similarly identify four a-helices but propose that they assemble into a tetramer, with the central coiled-coil domain being essential for both tetramer formation and writer-complex assembly. While these observations are broadly consistent with prior work, there is a nomenclature discrepancy that requires clarification. Su et al. (2022) also identified four a-helices in WTAP but described the structure as a homodimer rather than a tetramer (or a dimer of dimers). In contrast, the current manuscript refers to this architecture as a tetramer. The authors should acknowledge this difference in terminologies and clearly explain how their findings refine or advance the existing role of WTAP and the structural model of the writer-complex assembly.

2. Although in this manuscript, the authors have demonstrated that a fragment of WTAP exists as tetramer, an important unresolved question remains: whether the tetrameric state of WTAP is required for the writer-complex assembly and m6A methylation. The crystallographic data do not establish whether WTAP exists as tetramers in vivo (in cells) or whether this tetrameric state regulates interactions of WTAP with other writer complex proteins. For example, although the authors show that mutating leucine residues within the coiled-coil domain disrupts WTAP's interaction with METTL3 (Figure 3), this does not directly demonstrate that METTL3 binds tetrameric WTAP.
Given that WTAP can exist in monomeric, dimeric, and tetrameric states (Figure 1), it is important to determine whether mutations in the coiled-coil domain alter the oligomeric states of WTAP and the interaction of WTAP with other proteins. For example, how shifting WTAP from a tetramer to a dimer or monomeric species, can alter the interaction of WTAP with METTL3 and other proteins of the writer complex. It would be interesting to test whether altering the oligomeric state of WTAP impacts the m6A methylation levels.
Su et al. demonstrated that the WTAP dimer is stabilized by an extensive interface that involves three salt bridges, eight hydrogen bonds, and multiple hydrophobic interactions (several of which are also highlighted in the present manuscript). The authors should align their proposed WTAP tetramer structure with the previously reported cryo-EM homodimer structure of WTAP to determine whether it overlaps with any of the established interaction sites. Moreover, the authors should integrate this prior work to clarify which aspects of their findings align with previous observations and which are novel.

3. WTAP interacts with METTL3, METTL14 and VIRMA (Su et al., 2022; Ping et al., 2014). Moreover, the C-terminal region of ZC3H13 (residues 1461-1729) interacts with WTAP, VIRMA, and HAKAI (Wen et al., 2018). However, this C-terminal region of ZC3H13 is structurally uncharacterized and is likely an intrinsically disordered region, suggesting its interactions may be flexible or transient in nature. Therefore, the authors should provide rationale for why they chose to investigate ZC3H13 as a WTAP-binding partner.

4. The authors use techniques like circular dichroism (CD) and FPLC-MALS to conclude that WTAP assembles into a tetramer.

However, both techniques have limitations, and they cannot reliably predict protein assemblies or higher order quaternary structures. For example, CD can only predict secondary structures, such as a-helices, but it cannot distinguish between different oligomeric states. Similarly, MALS can provide estimates of molecular mass but cannot inform about structural organization or protein assembly. In addition, coiled-coil fragments are well known to oligomerize non-specifically at high protein concentrations (~100 μM) used in their MALS experiments. Given these technical limitations, to substantiate their claims, the authors should analyze full-length WTAP protein and the F1-F4 fragments side-by-side using both native and SDS-PAGE to corroborate the MALS-based oligomeric observations reported in Figure 1D.

5. The authors report that reducing WTAP protein levels leads to only 50% decrease in m6A/A level compared to control (Figure 3D). However, previous work showed that an 85-90% knockdown of WTAP resulted in a corresponding 85-90% reduction in global m6A levels (Schwartz et al., 2014). This effect of WTAP knockdown on m6A levels were more pronounced than depletion of METTL3, METTL14, or VIRMA. A potential explanation for this discrepancy could be that LC/MS was performed on total RNA rather than purified mRNA. LC/MS quantifies m6A on all RNAs and cannot distinguish between m6A in mRNA and other RNAs such as rRNA and tRNA. Because rRNA and tRNA are abundant, changes detected by LC/MS may not accurately reflect mRNA-specific methylation, potentially confounding the readout of writer complex activity. Therefore, the authors perform mRNA methylation assays on purified mRNAs.

6. The authors show an effect of WTAP tetramer on chromosome segregation (Figure 4). Although WTAP knockdown has been reported to induce G2/M arrest and impair cell proliferation (Horiuchi et al., 2006), it is not known if WTAP with chromosomes or the mitotic assembly during cell division. Therefore, the authors should perform immunostaining to compare the distribution/localization of wild-type and L2E mutant WTAP during cell division. This would help explain their results in Figures 4F and 4G, where re-expression of wild-type WTAP, but not L2E mutant WTAP rescues mitotic defects.
It is unclear from Figure 4C (bottom panel), why there are two sets of time-lapse observations recorded under shWTAP-BFP. Are these from two different shRNAs? Please label all the panels.

Horiuchi, K., Umetani, M., Minami, T., Okayama, H., Takada, S., Yamamoto, M., Aburatani, H., Reid, P. C., Housman, D. E., Hamakubo, T., & Kodama, T. (2006). Wilms' tumor 1-associating protein regulates G2/M transition through stabilization of cyclin A2 mRNA. Proceedings of the National Academy of Sciences of the United States of America, 103(46), 17278-17283. https://doi.org/10.1073/pnas.0608357103

7. An earlier study showed that ZC3H13 is required for the nuclear localization of the ZC3H13-WTAP-VIRMA-HAKAI complex and that full-length ZC3H13 resides in nuclear speckles (Wen et al., 2018). In that study, while the ZC3H13 (1-1460) retained speckle localization similar to the full-length protein, the C-terminal fragment (1461-1729) remained nuclear but showed a diffused distribution.

In contrast, the present manuscript reports that although full-length ZC3H13 is nuclear, the C-terminal fragment of ZC3H13 (1460-1669) shows a clear cytoplasmic localization. The authors should discuss potential reasons for these observations, such as differences in the reporters used or the placement of reporters in the ZC3H13 expression construct.

Wen, J., Lv, R., Ma, H., Shen, H., He, C., Wang, J., Jiao, F., Liu, H., Yang, P., Tan, L., Lan, F., Shi, Y. G., He, C., Shi, Y., & Diao, J. (2018). Zc3h13 Regulates Nuclear RNA m6A Methylation and Mouse Embryonic Stem Cell Self-Renewal. Molecular cell, 69(6), 1028-1038.e6. https://doi.org/10.1016/j.molcel.2018.02.015

8. The authors were unable to crystallize full-length WTAP, but they do not discuss why. They should include a discussion point on the likely reasons, such as the extensive intrinsically disordered C-terminus, to clarify for the readers why crystallizing the full-length protein was challenging. If intrinsic disorder was indeed the limitation, crystallizing the F1 fragment, which retains most structured elements while excluding the disordered tail, would be a more appropriate approach. Without this clarification, the structural interpretation remains ambiguous, given that the N-terminal region (1-50) of WTAP forms dimers while the middle coiled-coil region (150-245) forms tetramers.

9. The main missing aspect of the study is whether WTAP exists as a stable dimer-of-dimer in the cytosol, or if it is a mixture of dimers and dimers-of-dimer, and if signals regulate this process. Information on whether METTL3 association with WTAP in cells is regulated by this process is also missing. Any additional information on these main questions would be appreciated since this would reveal if the phenomenon described in this manuscript is regulatory for m6A formation.

Referee #2:

This manuscript by Zhang et al. reports a tetrameric assembly of the m6A writer subunit WTAP that is required for interactions with other complex subunits and for proper mitosis. The authors present crystal structures of WTAP N terminal and central regions, identify amino acids required for tetramerization, and show that point mutations that disrupt tetramer formation impair binding to METTL3/METTL14 and ZC3H13 and fail to rescue mitotic defects after WTAP knockdown. Transcriptome and m6A profiling identify candidate misregulated transcripts potentially relevant to the mitotic phenotype.
The finding that WTAP can oligomerize and that oligomerization is functionally important is novel and interesting. The structural data are generally convincing; however, the connection to m6A deposition and the mechanistic link to the mitotic defects need stronger support.

Major comments

1. The m6A mapping data are underexploited and contain features that raise concern. The reported metagene profile shows an unexpected enrichment at the 5′ UTR, which is atypical for antibody-based m6A mapping and may reflect a high false-positive rate or technical bias.

Furthermore there is no clear, robust decrease of m6A levels upon WTAP knockdown, which undermines the claim that the mitotic phenotypes are mediated by altered m6A deposition. Given that WTAP has m6A independent roles (and localizes to spindles), the possibility that the mitotic defect is m6A independent must be considered and tested.

The authors should provide mapping statistics, peak-calling thresholds, and measures of reproducibility across replicates. Show representative genome browser tracks for key loci (input, IP, and KD) to illustrate signal changes and to support claims about site-specific m6A alterations. Ideally the authors could use higher-confidence, stoichiometry-aware m6A mapping methods (for example, GLORI, or direct RNA sequencing with Nanopore) and re-analyze to obtain a more reliable m6A landscape. Show a clear global or site-specific reduction in m6A upon WTAP KD. Correlate changes in m6A with transcriptome changes (differential expression/splicing) and prioritize candidates for follow-up validation.

2. The authors should test whether loss of METTL3/METTL14 phenocopies the mitotic defects observed with WTAP KD. The authors could use a catalytic METTL3 inhibitor (or catalytically dead METTL3) to probe whether methyltransferase activity is required for the mitotic phenotype. These experiments are critical to link the structural/interaction data to m6A biology.

Minor/comments

1. The crystal structures should be described in greater detail with respect to the tetramerization interfaces: better specify the interface residues, the nature of the interactions, highlight the hydrophobic surfaces, and the key side chains in the figures.

2. Discuss how the tetramer architecture is compatible with previously published structures of the methyltransferase complex. Does tetramerization sterically permit binding with respect to the previous described horse-shaped structure?

3. Figure S2 (FPLC MALS) should include the wild type proteins alongside mutants to directly compare oligomeric states.

Point-by-point responses to the comments raised by the reviewers

Before point-by-point responses to the referees' comments, we thank both reviewers for recognizing the novelty and interests of our works and their critical and constructive suggestions and guidance that help us to efficiently improve our manuscript. Our responses are shown in blue.

Referee #1:

In this study, Zhang et al. investigate the structural organization of WTAP and its role in assembling the m6A writer complex. Using a combination of circular dichroism, FPLC-MALS, and X-ray crystallography techniques, the authors demonstrate that a fragment of WTAP protein forms a tetramer through a central coiled-coil domain. Disrupting this tetramer WTAP fragment impairs the binding of WTAP with METTL3. The authors further show that tetramer-disrupting mutations decrease global m6A levels, fail to rescue proliferation defects in WTAP-depleted cells, and lead to chromosome segregation errors during mitosis. Together, these results suggest that the tetrameric WTAP scaffold is required for the writer complex assembly and expression of mitosis-related genes.
This manuscript strongly highlights that tetrameric nature of WTAP, which was not well-stated in the prior cryoEM. Also, the study makes the intruiging insight that METTL3 binding depends on acquiring the dimer-of-dimer conformation. This could be a major regulatory step. Thus, this study has important insights for how m6A could be regulated, although these conformations are not shown to be dynamically regulated in cells. Regardless, these new insights are drivers for publication in this journal.
While this study explores a novel connection between WTAP oligomerization, with writer complex assembly and m6A-dependent cell-cycle regulation, a few methodological and conceptual issues require further clarification:

We thank the reviewer for the positive and insightful assessment of our study. We appreciate the reviewer's recognition of the novelty of our findings, particularly the identification of a tetrameric architecture of WTAP and its role in supporting METTL3 binding. Below we provide point-by-point responses to the methodological and conceptual issues raised.

1. WTAP is composed of an N-terminal region, a central coiled-coil domain, and a C-terminal segment (Schöller et al., RNA, 2018). An earlier cryo-EM study demonstrated that WTAP forms a saddle-shaped structure consisting of four a-helices (H1-H4) arranged as a homodimer stabilized by coiled-coil interactions. The H3-H4 helices of WTAP bind the METTL3/METTL14 heterodimer (Su et al., Cell Res., 2022). In the present study, Zhang et al. similarly identify four a-helices but propose that they assemble into a tetramer, with the central coiled-coil domain being essential for both tetramer formation and writer-complex assembly. While these observations are broadly consistent with prior work, there is a nomenclature discrepancy that requires clarification. Su et al. (2022) also identified four a-helices in WTAP but described the structure as a homodimer rather than a tetramer (or a dimer of dimers). In contrast, the current manuscript refers to this architecture as a tetramer. The authors should acknowledge this difference in terminologies and clearly explain how their findings refine or advance the existing role of WTAP and the structural

model of the writer-complex assembly.

We thank the reviewer for raising this important point regarding the structural nomenclature of WTAP and for the opportunity to clarify the relationship between our findings and the previous study by Su et al[1]. First, we note that the structural regions analyzed differ between the two studies. Su et al. defined four α-helices across a broader region of WTAP (H1: residues 64-99, H2: residues 115-144, H3: residues 149-177, and H4: residues 179-247). In contrast, our crystallographic analysis focuses on the central coiled-coil region (residues 150-245), which encompasses helices H3 and H4 (from Su et al's study) and forms a stable oligomeric assembly suitable for structural determination. Importantly, our results are consistent with the structural model reported by Su et al. In both studies, the fundamental building block of WTAP is a dimer in which two monomers contribute α-helical elements to form the characteristic saddle-shaped structure. The key advance of our study is the demonstration that these dimeric units can further assemble into a higher-order tetramer (i.e., a dimer of dimers) through interactions within the central coiled-coil region. Our biochemical analyses further show that this region is required both for tetramer formation and for efficient assembly of the m^6A writer complex.

Thus, rather than contradicting the previous cryo-EM model, our findings extend it by revealing a hierarchical assembly mechanism in which WTAP dimers associate through their coiled-coil domains to form a tetrameric scaffold. To clarify this relationship, we have added a schematic representation illustrating the hierarchical assembly of WTAP from dimers to tetramers (Figure R1A-D) and revised the manuscript accordingly.

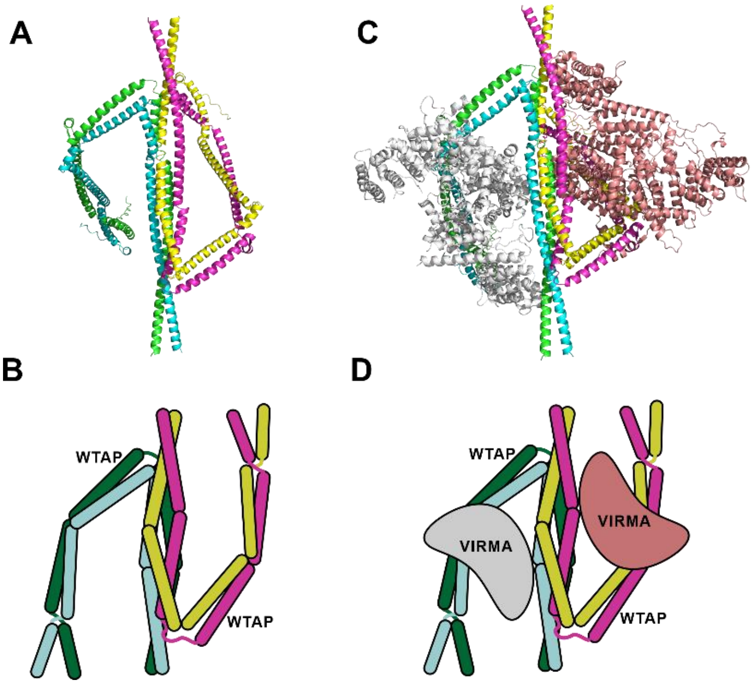

Figure R1. Structural organization of WTAP and its hierarchical assembly model.

(A) Domain architecture of the modeled WTAP 1-245 fragment used for structural analysis.

(B) Schematic model illustrating the hierarchical assembly of WTAP, in which dimeric units further associate through the central coiled-coil domain to form a tetramer.

(C) Domain organization of WTAP and its interaction partner VIRMA.

(D) Schematic representation of the WTAP-VIRMA complex architecture.

2. Although in this manuscript, the authors have demonstrated that a fragment of WTAP exists as tetramer, an important unresolved question remains: whether the tetrameric state of WTAP is required for the writer-complex assembly and m6A methylation. The crystallographic data do not establish whether WTAP exists as tetramers in vivo (in cells) or whether this tetrameric state regulates interactions of WTAP with other writer complex proteins. For example, although the authors show that mutating leucine residues within the coiled-coil domain disrupts WTAP's interaction with METTL3 (Figure 3), this does not directly demonstrate that METTL3 binds tetrameric WTAP.

Given that WTAP can exist in monomeric, dimeric, and tetrameric states (Figure 1), it is important to determine whether mutations in the coiled-coil domain alter the oligomeric states of WTAP and the interaction of WTAP with other proteins. For example, how shifting WTAP from a tetramer to a dimer or monomeric species, can alter the interaction of WTAP with METTL3 and other proteins of the writer complex. It would be interesting to test whether altering the oligomeric state of WTAP impacts the m6A methylation levels.

Su et al. demonstrated that the WTAP dimer is stabilized by an extensive interface that involves three salt bridges, eight hydrogen bonds, and multiple hydrophobic interactions (several of which are also highlighted in the present manuscript). The authors should align their proposed WTAP tetramer structure with the previously reported cryo-EM homodimer structure of WTAP to determine whether it overlaps with any of the established interaction sites. Moreover, the authors should integrate this prior work to clarify which aspects of their findings align with previous observations and which are novel.

We thank the reviewer for raising this important question regarding the oligomeric state of WTAP and its functional relevance for writer complex assembly and m^6A methylation. We have performed additional experiments and analyses to address these points as outlined below.

(1) Evidence for tetrameric WTAP in solution and in cells

In addition to the FPLC-MALS analysis presented in the original manuscript, we have now confirmed the tetrameric assembly of WTAP in solution using single-molecule mass photometry (Figure R5 and see below for details). This method allows accurate determination of protein oligomeric states at near-physiological concentrations. The results consistently support a tetrameric species of WTAP in solution. We further performed native PAGE analysis using cell lysates expressing WTAP and revealed a higher-order WTAP complex in cells. Together, these additional data support the presence of tetrameric WTAP under physiological conditions.

(2) Functional consequences of disrupting the tetrameric interface

To test whether the tetrameric state is functionally important for writer complex assembly, we introduced a structure-guided mutation within the coiled-coil interface (L2E) designed to disrupt tetramer formation. FPLC-MALS analysis confirmed that this mutation shifts WTAP from a tetramer to a dimeric species. Co-immunoprecipitation experiments show that the L2E mutant exhibits markedly reduced binding to METTL3 and METTL14 (Revised Figure 3), indicating that tetramer formation promotes efficient interaction with the methyltransferase complex.

Furthermore, in WTAP knockdown-rescue experiments, the L2E mutant fails to restore global m^6A levels compared with wild-type WTAP (Revised Figure 3D). These results support the conclusion that tetramerization of WTAP is required for efficient writer complex assembly and m^6A methylation activity.

(3) Structural comparison with the previously reported WTAP cryo-EM structure

We have now performed a detailed comparison between our tetrameric WTAP structure and the previously reported cryo-EM structure of WTAP bound to VIRMA (PDB: 7VF2)[1]. Structural superposition reveals a notable difference in the spacing between the helices within the coiled-coil region. In the VIRMA-bound structure, the inter-helical distances are 5.9 Å, 7.7 Å, and 7.1 Å, whereas in our WTAP M-CC structure the corresponding distances are significantly larger (10.3 Å, 15.2 Å, and 15.9 Å) (Figure R2). These observations suggest that VIRMA binding may induce a conformational rearrangement that brings the helices closer together and stabilizes the complex.

In addition, our study provides new structural information for the WTAP N-terminal region (residues 1-50), which was not resolved in the previous cryo-EM structure. By integrating this N-terminal crystal structure with the cryo-EM structure of the WTAP core (residues 64-247), we constructed a structural model of full-length coiled-coil region of WTAP (residues 1-247) (Figure R3). This integrated model provides a more complete view of WTAP architecture and its potential assembly within the writer complex. We have revised the manuscript accordingly to clarify the relationship between our findings and the previous structural model.

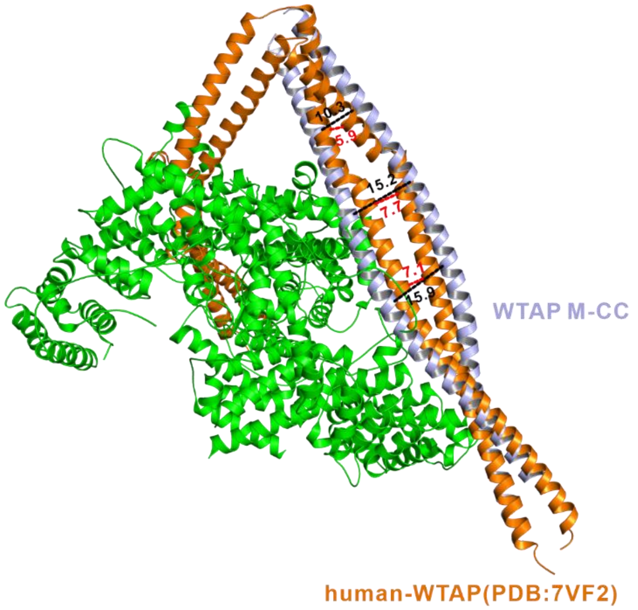

Figure R2. Structural comparison of WTAP M-CC with the previously reported WTAP structure. Superposition of the WTAP M-CC crystal structure determined in this study (cyan) with the previously reported WTAP cryo-EM structure bound to VIRMA (PDB: 7VF2; orange) from Su et al. Distances between the two helices within the coiled-coil region are indicated, highlighting the increased spacing observed in the absence of VIRMA.

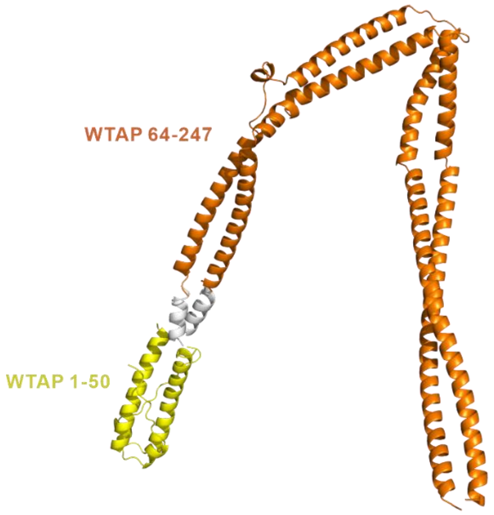

Figure R3. Structural model of full-length coiled-coil region of WTAP.
Structural model of WTAP (residues 1-247) generated by integrating the N-terminal crystal structure determined in this study (yellow) with the previously reported cryo-EM structure of the core region (residues 64-247; orange). The linker region (residues 51-63; grey) was modeled using AlphaFold prediction.

3. WTAP interacts with METTL3, METTL14 and VIRMA (Su et al., 2022; Ping et al., 2014). Moreover, the C-terminal region of ZC3H13 (residues 1461-1729) interacts with WTAP, VIRMA, and HAKAI (Wen et al., 2018). However, this C-terminal region of ZC3H13 is structurally uncharacterized and is likely an intrinsically disordered region, suggesting its interactions may be flexible or transient in nature. Therefore, the authors should provide rationale for why they chose to investigate ZC3H13 as a WTAP-binding partner.

We thank the reviewer for this important question regarding the rationale for investigating ZC3H13 as a WTAP-binding partner. ZC3H13 is a key regulatory component of the m^6A writer complex that links WTAP to additional regulatory subunits and plays an essential role in stabilizing the nuclear writer complex. Previous studies have shown that the C-terminal region of ZC3H13 interacts with WTAP, VIRMA, and HAKAI, thereby bridging multiple components of the writer complex[2]. Although this C-terminal region is predicted to be intrinsically disordered and has not yet been structurally characterized, biochemical and cellular studies indicate that it is essential for maintaining the integrity and nuclear localization of the writer complex. In addition, ZC3H13 has also been reported to interact with RBM15[3], another key component of the writer complex that recruits the methyltransferase machinery to specific RNA substrates. These observations suggest that ZC3H13 serves as an important organizational hub within the writer complex, coordinating multiple protein–protein interactions involved in m^6A deposition.

Consistent with this view, in a parallel study currently under submission, we have investigated the molecular interaction between RBM15 and ZC3H13 and identified a direct binding interface between these two proteins. Although this work focuses on a different aspect of writer complex assembly, it further supports the role of ZC3H13 as a central interaction platform within the m^6A

regulatory network. Because WTAP functions as a key scaffold protein within the m^{6}A writer complex, understanding how WTAP engages with regulatory partners such as ZC3H13 is important for elucidating the higher-order organization of the complex. Therefore, despite the predicted intrinsic disorder of the ZC3H13 C-terminal region, investigating its interaction with WTAP provides important mechanistic insight into how the WTAP scaffold coordinates assembly of the writer complex. We have clarified this rationale in the revised manuscript and added the relevant references.

4. The authors use techniques like circular dichroism (CD) and FPLC-MALS to conclude that WTAP assembles into a tetramer. However, both techniques have limitations, and they cannot reliably predict protein assemblies or higher order quaternary structures. For example, CD can only predict secondary structures, such as a-helices, but it cannot distinguish between different oligomeric states. Similarly, MALS can provide estimates of molecular mass but cannot inform about structural organization or protein assembly. In addition, coiled-coil fragments are well known to oligomerize non-specifically at high protein concentrations (~100 μM) used in their MALS experiments. Given these technical limitations, to substantiate their claims, the authors should analyze full-length WTAP protein and the F1-F4 fragments side-by-side using both native and SDS-PAGE to corroborate the MALS-based oligomeric observations reported in Figure 1D.

We thank the reviewer for this thoughtful comment regarding the limitations of CD and MALS in determining protein oligomeric states. To further substantiate the oligomeric organization of WTAP, we performed additional biochemical analyses as suggested.

(1) SDS-PAGE and native PAGE analysis of WTAP fragments

We expressed and purified full-length WTAP together with the F1-F4 fragments and analyzed them side-by-side by SDS-PAGE and native PAGE. SDS-PAGE confirmed the purity of all proteins and their expected monomeric molecular weights (Figure R4A). We then examined these proteins using native PAGE (both 4-20% gradient gels and 8% fixed-percentage gels). However, all WTAP constructs displayed markedly anomalous migration patterns, appearing substantially larger than their theoretical molecular masses (Figure R4B, C). This behavior is likely due to the elongated coiled-coil architecture of WTAP fragments, which can lead to atypical electrophoretic mobility in native gels. Similar anomalous migration has been reported for other extended coiled-coil proteins. Therefore, native PAGE did not provide a reliable estimate of molecular mass for this protein system.

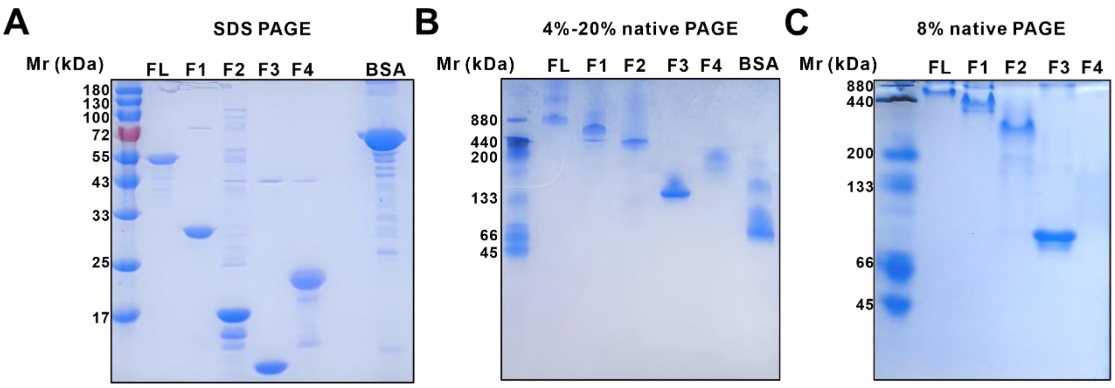

Figure R4. Electrophoretic analysis of WTAP full-length protein and truncation fragments.
(A) SDS-PAGE analysis of purified full-length WTAP and truncation fragments (F1-F4). Proteins were visualized by Coomassie blue staining. BSA was included as a control.
(B, C) Native PAGE analysis of the same protein samples using a 4-20% gradient gel (B) and an 8% gel (C). Proteins were visualized by Coomassie blue staining. All WTAP constructs exhibit anomalous migration behavior, likely due to their elongated coiled-coil architecture.

(2) Single-molecule mass photometry at dilute protein concentrations

To further address the reviewer's concern that coiled-coil fragments may oligomerize non-specifically at high concentrations used in MALS experiments (~100 μM), we performed single-molecule mass photometry to determine the oligomeric states of WTAP constructs under dilute conditions. These measurements were carried out at a protein concentration of 0.2 μM, well below the range where non-specific coiled-coil aggregation is typically observed.

The results consistently support a tetrameric assembly of WTAP. Full-length WTAP displayed a predominant species of approximately 188 kDa, which corresponds closely to a tetramer based on the theoretical monomer mass (Figure R5A). Similarly, the F1 fragment showed a dominant peak at ~124 kDa, consistent with a tetramer (Figure R5B). The F2 fragment predominantly formed a dimer (~42 kDa) (Figure R5C), whereas the F3 fragment formed a tetramer (~51 kDa) (Figure R5D). In contrast, the F4 fragment behaved as a monomer (~40 kDa) (Figure R5E). Due to the lower detection limit of mass photometry (~30 kDa), the F4 construct was analyzed with its fusion tag (Trx) retained. Together, these single-molecule measurements performed at low protein concentrations provide independent evidence supporting the tetrameric assembly of WTAP and are fully consistent with the MALS analysis presented in the original manuscript. These new data have been incorporated into the revised manuscript.

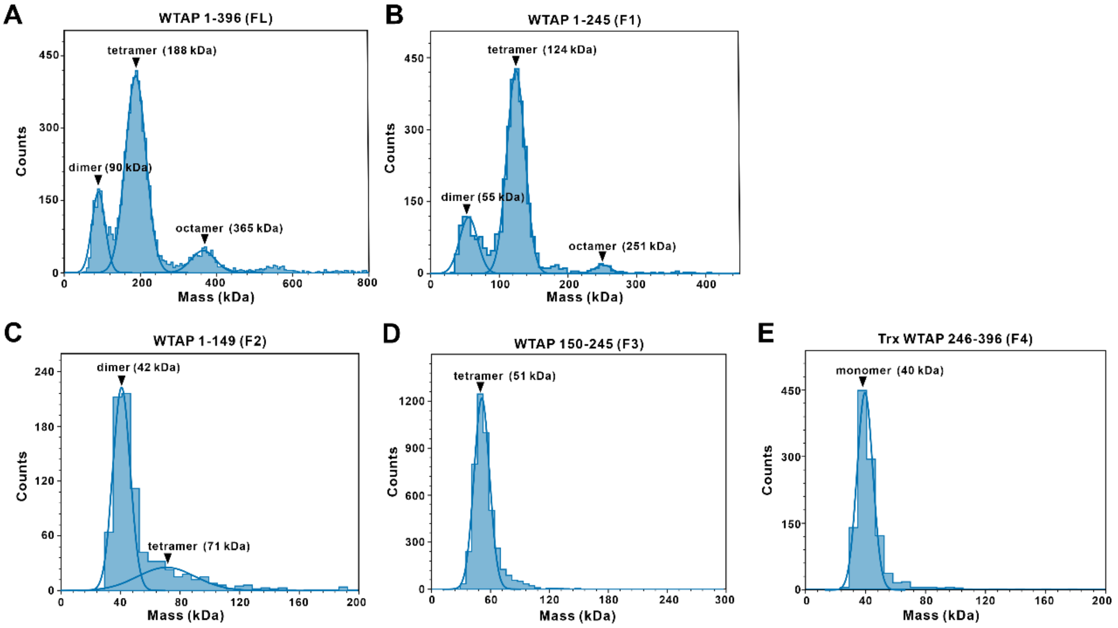

Figure R5. Mass photometry analysis of WTAP oligomeric states.
(A-E) Mass photometry measurements of purified full-length WTAP (A) and truncation fragments F1 (B), F2 (C), F3 (D), and F4 (E). Experiments were performed at a protein concentration of 0.2

μM. The measured molecular masses of the dominant species are indicated.

5. The authors report that reducing WTAP protein levels leads to only 50% decrease in m6A/A level compared to control (Figure 3D). However, previous work showed that an 85-90% knockdown of WTAP resulted in a corresponding 85-90% reduction in global m6A levels (Schwartz et al., 2014). This effect of WTAP knockdown on m6A levels were more pronounced than depletion of METTL3, METTL14, or VIRMA. A potential explanation for this discrepancy could be that LC/MS was performed on total RNA rather than purified mRNA. LC/MS quantifies m6A on all RNAs and cannot distinguish between m6A in mRNA and other RNAs such as rRNA and tRNA. Because rRNA and tRNA are abundant, changes detected by LC/MS may not accurately reflect mRNA-specific methylation, potentially confounding the readout of writer complex activity. Therefore, the authors perform mRNA methylation assays on purified mRNAs.

We thank the reviewer for raising this important point regarding the interpretation of the LC/MS measurements of m^6A levels.

First, we would like to clarify that our LC/MS analysis was performed on purified polyadenylated mRNA rather than total RNA. As described in the Methods section, poly(A) RNA was isolated using oligo(dT) beads prior to LC-MS/MS quantification. Therefore, our measurements specifically reflect m^6A modifications on mRNA and are not confounded by the abundant rRNA or tRNA species present in total RNA. Second, we acknowledge the study by Schwartz et al. (2014), which reported an ~85-90% reduction in global m^6A levels upon WTAP knockdown. Notably, those experiments were performed in A549 cells, whereas our measurements were conducted in HeLa cells. Differences in cell type, expression levels of writer complex components, or regulatory context may contribute to quantitative differences in the observed reduction of m^6A levels. Third, previous studies performed in HeLa cells report reductions similar to what we observe here. For example, Liu et al. (2014) quantified m^6A levels in polyadenylated RNA from HeLa cells and found that WTAP knockdown reduced m^6A levels by approximately 50%[5], which is consistent with our results (Figure R6). Together, these observations indicate that the magnitude of m^6A reduction upon WTAP depletion can vary depending on cellular context, and our results are consistent with previously reported measurements in HeLa cells. These results further support that partial loss of WTAP is sufficient to significantly impair m6A deposition while still maintaining residual methylation activity.

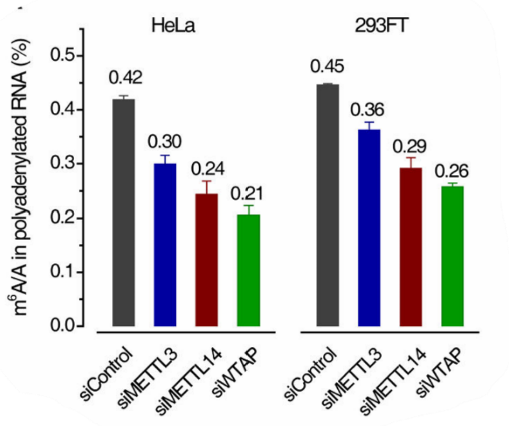

Figure R6. Previously reported effects of METTL3, METTL14, and WTAP knockdown on m^6A levels in HeLa cells.
LC-MS/MS quantification of the m^6A/A ratio in polyadenylated RNA isolated from HeLa and 293FT cells following knockdown of METTL3, METTL14, or WTAP (adapted from Liu et al., 2014[5]). In HeLa cells, WTAP depletion results in an approximately 50% reduction in m^6A levels, consistent with the observations reported in this study.

6. The authors show an effect of WTAP tetramer on chromosome segregation (Figure 4). Although WTAP knockdown has been reported to induce G2/M arrest and impair cell proliferation (Horiuchi et al., 2006), it is not known if WTAP with chromosomes or the mitotic assembly during cell division. Therefore, the authors should perform immunostaining to compare the distribution/localization of wild-type and L2E mutant WTAP during cell division. This would help explain their results in Figures 4F and 4G, where re-expression of wild-type WTAP, but not L2E mutant WTAP rescues mitotic defects.

It is unclear from Figure 4C (bottom panel), why there are two sets of time-lapse observations recorded under shWTAP-BFP. Are these from two different shRNAs? Please label all the panels.

Horiuchi, K., Umetani, M., Minami, T., Okayama, H., Takada, S., Yamamoto, M., Aburatani, H., Reid, P. C., Housman, D. E., Hamakubo, T., & Kodama, T. (2006). Wilms' tumor 1-associating protein regulates G2/M transition through stabilization of cyclin A2 mRNA. Proceedings of the National Academy of Sciences of the United States of America, 103(46), 17278-17283. https://doi.org/10.1073/pnas.0608357103

We thank the reviewer for these helpful suggestions and we have performed new experiments as suggested by the reviewer shown as below.

(1) Localization of WTAP during mitosis
To compare the localization of wild-type WTAP and the tetramer-disrupting L2E mutant during cell division, we generated HeLa cell lines stably expressing GFP-tagged WTAP WT or WTAP L2E. Cells were synchronized by thymidine block and released into mitosis, followed by immunofluorescence analysis at different mitotic stages. As shown in Figure R7, both WTAP WT and the L2E mutant display similar localization patterns during mitosis. Upon mitotic entry, WTAP redistributes from the nucleus and shows clear co-localization with α-tubulin-labeled spindle structures throughout mitosis. Importantly, the L2E mutation does not alter the mitotic localization of WTAP. These results indicate that the chromosome segregation defects observed upon expression of the L2E mutant are unlikely to arise from altered subcellular localization of WTAP. Instead, we propose that disruption of WTAP tetramer formation impairs assembly of the m^6A writer complex and consequently affects the expression of genes involved in chromosome segregation, consistent with our observations on m^6A levels and downstream cellular phenotypes presented in the manuscript.

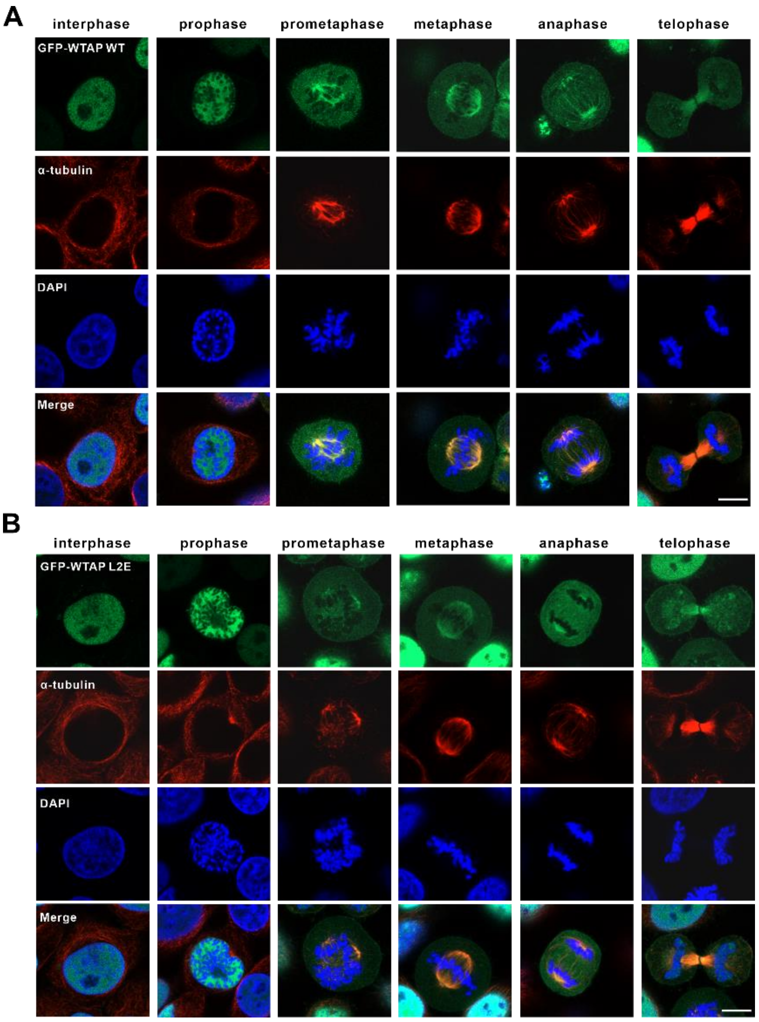

Figure R7. Localization of WTAP WT and L2E variant during mitosis.
Immunofluorescence images of HeLa cells stably expressing GFP-tagged wild-type WTAP (A) or the tetramer-disrupting L2E mutant (B) at different stages of mitosis. Cells were stained with α-tubulin to visualize the mitotic spindle. Both WTAP WT and WTAP L2E display similar localization patterns and co-localize with spindle structures during mitosis. Scale bar: 10 μm.

(2) Clarification of Figure 4C labeling

We apologize for the lack of clarity in the labeling of Figure 4C in the original manuscript. The two sets of time-lapse observations shown under the *shWTAP-BFP* condition do not correspond to two different shRNAs. Rather, they represent two distinct categories of chromosome segregation errors observed under the same experimental condition. The figure has now been revised to clearly label these phenotypes directly on the panels in the updated manuscript.

7. An earlier study showed that ZC3H13 is required for the nuclear localization of the ZC3H13-WTAP-VIRMA-HAKAI complex and that full-length ZC3H13 resides in nuclear speckles (Wen et al., 2018). In that study, while the ZC3H13 (1-1460) retained speckle localization similar to the full-length protein, the C-terminal fragment (1461-1729) remained nuclear but showed a diffused distribution.

In contrast, the present manuscript reports that although full-length ZC3H13 is nuclear, the C-terminal fragment of ZC3H13 (1460-1669) shows a clear cytoplasmic localization. The authors should discuss potential reasons for these observations, such as differences in the reporters used or the placement of reporters in the ZC3H13 expression construct.

Wen, J., Lv, R., Ma, H., Shen, H., He, C., Wang, J., Jiao, F., Liu, H., Yang, P., Tan, L., Lan, F., Shi, Y. G., He, C., Shi, Y., & Diao, J. (2018). Zc3h13 Regulates Nuclear RNA m6A Methylation and Mouse Embryonic Stem Cell Self-Renewal. Molecular cell, 69(6), 1028-1038.e6. https://doi.org/10.1016/j.molcel.2018.02.015

We thank the reviewer for pointing out the apparent discrepancy between our observations and those reported by Wen et al. (2018) regarding the localization of the ZC3H13 C-terminal region.

First, we note that the C-terminal region of ZC3H13 used in our study corresponds closely to the region analyzed by Wen et al. (2018). Sequence alignment between mouse ZC3H13 (used in Wen et al.) and human ZC3H13 (used in our study) shows a very high degree of conservation within this region, suggesting that species differences are unlikely to account for the observed localization patterns.

To address this issue, we repeated the localization experiments in HeLa cells expressing GFP-tagged ZC3H13 C-terminal fragment (residues 1460-1669). In these repeated experiments, we observed that ZC3H13-C displays both nuclear and cytoplasmic distribution, with a clear and predominant nuclear signal, which is consistent with the diffuse nuclear localization described by Wen et al. (2018). Upon re-examining the images presented in our original submission, we realized that the example shown in the manuscript emphasized the cytoplasmic signal and did not fully represent the predominant nuclear localization observed in most cells. We have therefore repeated the experiment and replaced the original images with representative data that more accurately reflect the observed localization pattern. We further examined the interaction between WTAP and the ZC3H13 C-terminal fragment by co-expressing Flag-tagged WTAP (WT or L2E mutant) together with GFP-ZC3H13-C. In these experiments, wild-type WTAP co-localizes with ZC3H13-C in both nuclear and cytoplasmic compartments (Figure R8A). In contrast, the tetramer-disrupting WTAP L2E mutant fails to be efficiently recruited by ZC3H13-C and remains predominantly nuclear (Figure R8B). These results indicate that the localization pattern of the ZC3H13 C-terminal fragment is largely consistent with previous reports. The relevant figures and text have been revised accordingly in the manuscript.

We thank the reviewer for the careful examination of our data and apologize for the oversight in the original manuscript. The revised experiments more accurately represent the localization pattern of ZC3H13-C and further support our main conclusion that WTAP tetramerization contributes to its interaction with ZC3H13-containing complexes.

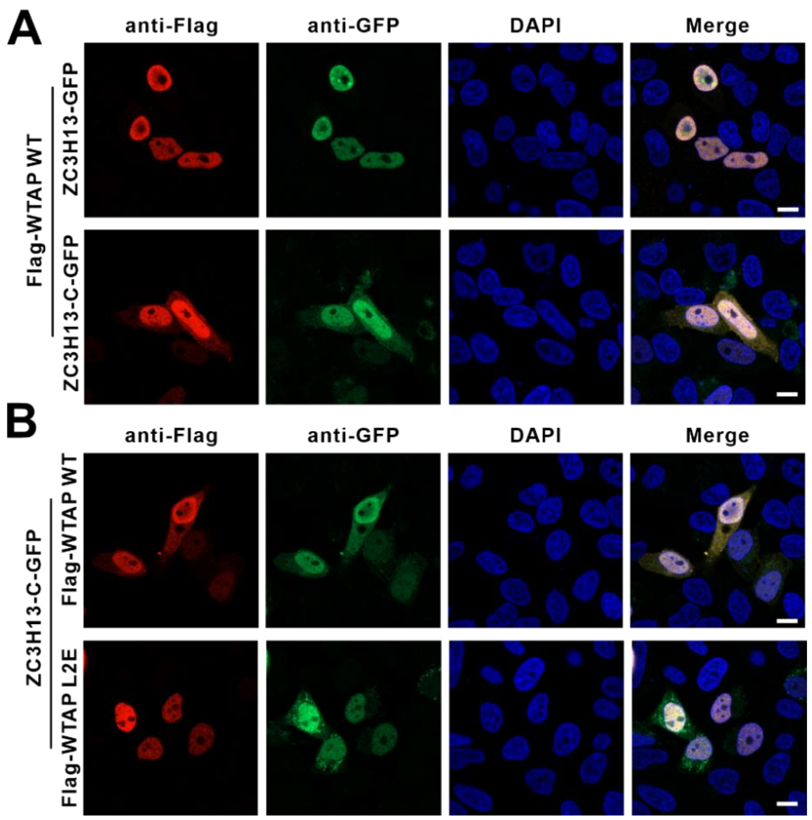

Figure R8. Co-localization analysis of WTAP and the ZC3H13 C-terminal fragment in HeLa cells. (A) Immunofluorescence images of HeLa cells co-expressing Flag-tagged WTAP (red, Alexa Fluor 568) and GFP-tagged ZC3H13 (full-length or C-terminal fragment). Full-length ZC3H13 co-localizes with WTAP in nuclear speckles. In contrast, the ZC3H13 C-terminal fragment displays both nuclear and cytoplasmic distribution and co-localizes with WTAP in these compartments. Nuclei were stained with DAPI. Scale bar: 10 μm.

(B) HeLa cells co-expressing Flag-tagged WTAP WT or the tetramer-disrupting mutant WTAP L2E together with GFP-ZC3H13-C. WTAP WT shows clear co-localization with ZC3H13-C both in the nuclear and cytoplasmic region, whereas the L2E mutant remains predominantly nuclear and shows reduced co-localization with ZC3H13-C. Scale bar: 10 μm.

8. The authors were unable to crystallize full-length WTAP, but they do not discuss why. They should include a discussion point on the likely reasons, such as the extensive intrinsically disordered C-terminus, to clarify for the readers why crystallizing the full-length protein was challenging. If intrinsic disorder was indeed the limitation, crystallizing the F1 fragment, which retains most structured elements while excluding the disordered tail, would be a more appropriate approach. Without this clarification, the structural interpretation remains ambiguous, given that the N-terminal region (1-50) of WTAP forms dimers while the middle coiled-coil region (150-245) forms tetramers.

We thank the reviewer for this helpful suggestion regarding the crystallization of full-length WTAP. As the reviewer correctly noted, the most likely reason that full-length WTAP could not be crystallized is the presence of an extensive intrinsically disordered C-terminal region. Such intrinsically disordered regions introduce conformational heterogeneity and flexibility, which

often interfere with the formation of well-ordered crystal lattices and therefore pose a major obstacle for crystallization. To address this limitation, we focused on structurally ordered regions of WTAP. We initially attempted to crystallize the WTAP F1 fragment (residues 1-245), which retains most of the predicted structured elements while excluding the disordered C-terminal tail. Although crystals were obtained, they diffracted poorly and did not yield data of sufficient resolution for structure determination.

As an alternative strategy, we determined high-resolution structures of the two individual structured regions of WTAP: the N-terminal dimerization domain (residues 1-50) and the central coiled-coil tetramerization domain (residues 150-245). These structures reveal that the N-terminal region forms a stable dimer, whereas the central coiled-coil region mediates tetramer formation. Based on these observations, we propose a hierarchical assembly model in which WTAP forms a dimeric building block through its N-terminal domain, while tetramerization is mediated by the central coiled-coil domain, resulting in a dimer-of-dimers organization (Figure R1A, B). This model reconciles the distinct oligomerization behaviors of the two regions and provides a structural framework for understanding WTAP assembly. We have now added a discussion of these points in the revised manuscript as guided by the reviewer.

9. The main missing aspect of the study is whether WTAP exists as a stable dimer-of-dimer in the cytosol, or if it is a mixture of dimers and dimers-of-dimer, and if signals regulate this process. Information on whether METTL3 association with WTAP in cells is regulated by this process is also missing. Any additional information on these main questions would be appreciated since this would reveal if the phenomenon described in this manuscript is regulatory for m6A formation.

We thank the reviewer for raising these important questions regarding the oligomeric state of WTAP in cells and its potential regulatory role in m^6A formation. To examine the oligomeric behavior of WTAP in a cellular context, we analyzed WTAP under native conditions using cell lysates expressing endogenous or exogenous WTAP. Native PAGE analysis revealed two major species with distinct electrophoretic mobility. However, we note that native PAGE migration does not always accurately reflect the molecular mass of elongated coiled-coil proteins, and therefore this method alone cannot reliably define the oligomeric state of WTAP. To more accurately determine the oligomeric state of WTAP, we performed single-molecule mass photometry analysis. These measurements show that full-length WTAP predominantly exists as a tetramer in solution, with a smaller fraction of dimeric species detected under dilute conditions (Figure R5A). These results support a model in which WTAP mainly forms a tetrameric assembly while retaining the capacity to exist in equilibrium with a dimeric state.

Importantly, our functional data suggest that WTAP tetramerization contributes to writer complex assembly. Specifically, the tetramer-disrupting mutation L2E significantly weakens the association between WTAP and METTL3, and this mutation also reduces global m^6A levels in cells. These observations indicate that tetramer formation facilitates productive interaction between WTAP and the METTL3/METTL14 methyltransferase complex.

We agree with the reviewer that it will be important in future work to determine whether cellular

signals regulate the equilibrium between different WTAP oligomeric states and thereby modulate m^6A deposition. While the current study establishes the structural basis and functional relevance of WTAP tetramerization, understanding potential regulatory mechanisms will require further investigation and is now discussed in the revised manuscript.

Referee #2:

This manuscript by Zhang et al. reports a tetrameric assembly of the m6A writer subunit WTAP that is required for interactions with other complex subunits and for proper mitosis. The authors present crystal structures of WTAP N terminal and central regions, identify amino acids required for tetramerization, and show that point mutations that disrupt tetramer formation impair binding to METTL3/METTL14 and ZC3H13 and fail to rescue mitotic defects after WTAP knockdown. Transcriptome and m6A profiling identify candidate misregulated transcripts potentially relevant to the mitotic phenotype.
The finding that WTAP can oligomerize and that oligomerization is functionally important is novel and interesting. The structural data are generally convincing; however, the connection to m6A deposition and the mechanistic link to the mitotic defects need stronger support.

We thank the reviewer for the positive evaluation of our study and for the constructive suggestions to strengthen the mechanistic connections between WTAP tetramerization, m^6A deposition, and the observed mitotic phenotypes. In the revised manuscript, we have expanded the analyses of our m^6A mapping datasets, added additional controls/quantifications where appropriate, and clarified how disruption of the tetrameric scaffold impacts writer-complex assembly and downstream gene regulation. Below, we provide a point-by-point response to each concern.

Major comments

1. The m6A mapping data are underexploited and contain features that raise concern. The reported metagene profile shows an unexpected enrichment at the 5' UTR, which is atypical for antibody-based m6A mapping and may reflect a high false-positive rate or technical bias.

Furthermore there is no clear, robust decrease of m6A levels upon WTAP knockdown, which undermines the claim that the mitotic phenotypes are mediated by altered m6A deposition. Given that WTAP has m6A independent roles (and localizes to spindles), the possibility that the mitotic defect is m6A independent must be considered and tested.

The authors should provide mapping statistics, peak-calling thresholds, and measures of reproducibility across replicates. Show representative genome browser tracks for key loci (input, IP, and KD) to illustrate signal changes and to support claims about site-specific m6A alterations. Ideally the authors could use higher-confidence, stoichiometry-aware m6A mapping methods (for example, GLORI, or direct RNA sequencing with Nanopore) and re-analyze to obtain a more reliable m6A landscape. Show a clear global or site-specific reduction in m6A upon WTAP KD. Correlate changes in m6A with transcriptome changes (differential expression/splicing) and prioritize candidates for follow-up validation.

We thank the reviewer for this important comment regarding the interpretation and robustness of our m^6A mapping data. We agree that careful analysis of the m^6A landscape is essential for establishing the mechanistic connection between WTAP tetramerization, m^6A deposition, and the observed mitotic phenotypes. In response to these concerns, we have re-analyzed the dataset and

added several additional analyses and controls, as described below.

(1) Mapping statistics, peak-calling thresholds, and reproducibility

We acknowledge that the enrichment of peaks in the 5′ UTR observed in the initial metagene plot is atypical for antibody-based m^{6}A mapping. Such patterns have occasionally been reported in MeRIP-seq datasets and may arise from technical background signals or biases inherent to antibody-based enrichment methods. To address this concern, we have re-analyzed the dataset with more stringent parameters. First, we now provide detailed sequencing and mapping statistics in Table EV2 in the revised manuscript, including total reads, mapping rates, and uniquely mapped reads, which confirm the high quality of the sequencing libraries. Second, m^{6}A peaks were re-called using exomePeak2 (v1.16.2) with stringent parameters (p-value < 0.05 and fold enrichment > 2). This re-analysis shows that the majority of high-confidence peaks remain enriched in coding sequences (CDS) and 3′ UTR regions, which is consistent with the canonical distribution of m^{6}A modifications. A residual signal in the 5′ UTR is still detectable but represents a minor fraction of the total peaks. To assess reproducibility across replicates, we additionally performed correlation analysis between biological replicates. As shown in Figure R9, pairwise Pearson correlation coefficients between samples are high ($R > 0.9$), indicating strong reproducibility of the MeRIP-seq datasets.

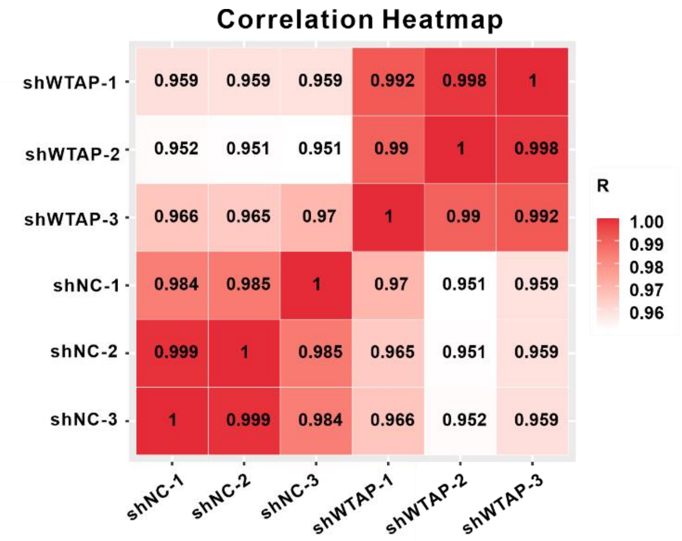

Figure R9. Reproducibility analysis of MeRIP-seq datasets.

Pearson correlation heatmap showing the pairwise correlation coefficients among biological replicates from shNC and shWTAP samples. The high correlation values ($R > 0.9$) indicate strong reproducibility of the MeRIP-seq datasets.

(2) Evidence for reduced m^{6}A levels upon WTAP knockdown

To evaluate global changes in m^{6}A modification upon WTAP depletion, we analyzed m^{6}A peak enrichment using cumulative distribution function (CDF) plots and violin plots based on normalized peak intensities from MeRIP-seq data. The CDF curve of shWTAP samples is consistently shifted to the left compared with shNC samples, indicating a systematic reduction in m^{6}A enrichment across the transcriptome (Figure R10). This trend is further supported by the

distribution of $\log_2$ peak intensities, which shows a significantly lower median value in WTAP knockdown samples (****$p < 0.0001$) (Figure R10). Importantly, m^{6}A enrichment values were normalized to input RNA signals, ensuring that the observed differences reflect changes in methylation enrichment rather than sequencing depth or immunoprecipitation efficiency. Together, these analyses support a global reduction of m^{6}A enrichment following WTAP knockdown.

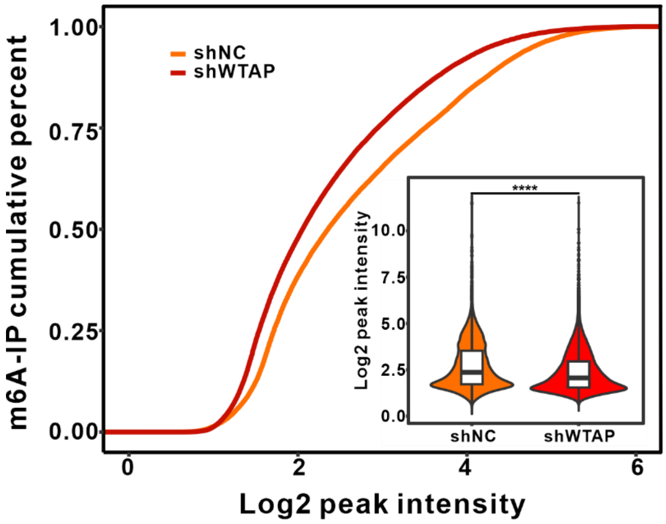

Figure R10. Global reduction of m^{6}A enrichment upon WTAP knockdown.
Cumulative distribution function (CDF) plot of normalized m^{6}A peak intensities in shNC and shWTAP cells. m^{6}A enrichment was calculated by normalizing immunoprecipitation signal to input. The leftward shift of the shWTAP curve indicates a global reduction in m^{6}A enrichment upon WTAP depletion. Statistical significance was assessed using a two-sided Wilcoxon test.

(3) Representative genome browser tracks for m^{6}A-regulated loci

To illustrate site-specific changes in m^{6}A modification, we generated genome browser tracks for representative transcripts identified in our analysis as guided by the reviewer. We identified a subset of high-confidence m^{6}A peaks that were consistently and significantly reduced upon WTAP depletion. As shown in Figure R11, IGV tracks for *KIF20A* and *DYRK3* display clear m^{6}A enrichment peaks in control cells that are markedly reduced in WTAP knockdown samples across biological replicates. These data demonstrate locus-specific loss of m^{6}A enrichment upon WTAP depletion.

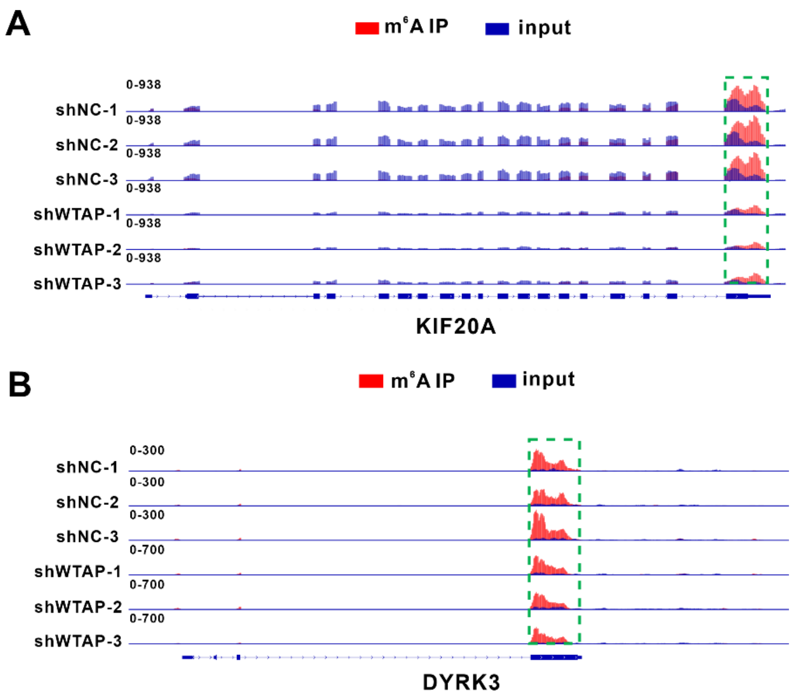

Figure R11. Representative genome browser tracks showing WTAP-dependent m^{6}A peaks. Genome browser views of m^{6}A-IP and input sequencing reads at the KIF20A (A) and DYRK3 (B) loci. For each gene, tracks are shown for input control, three shNC replicates, and three shWTAP replicates. m^{6}A peaks detected in control cells are markedly reduced upon WTAP knockdown, indicating site-specific loss of m^{6}A enrichment.

(4) Linking WTAP-dependent m^{6}A deposition to mitotic regulation

We appreciate the reviewer's important point that WTAP has previously reported m^{6}A-independent functions, including localization to spindle structures during mitosis. Therefore, establishing a connection between m^{6}A regulation and the observed mitotic phenotypes is essential. To further address this issue, we examined m^{6}A enrichment on KIF20A mRNA, a gene identified in our combined transcriptome and m^{6}A analyses. MeRIP-qPCR analysis confirms that WTAP knockdown leads to a significant reduction in m^{6}A enrichment on KIF20A transcripts. Importantly, this reduction in m^{6}A modification correlates with decreased KIF20A protein levels in WTAP-depleted cells. KIF20A is known to play an essential role in chromosome segregation and cytokinesis[7,8], and its dysregulation has been linked to mitotic defects. These observations support a model in which WTAP-dependent m^{6}A modification contributes to mitotic fidelity through regulation of KIF20A expression. Finally, we note that while our data strongly support an m^{6}A-mediated contribution to the observed mitotic phenotypes, we cannot fully exclude the possibility that WTAP may also influence mitosis through additional m6A-independent mechanisms, such as its reported association with spindle structures. We have clarified this point in the revised manuscript.

2. The authors should test whether loss of METTL3/METTL14 phenocopies the mitotic defects observed with WTAP KD. The authors could use a catalytic METTL3 inhibitor (or catalytically dead METTL3) to probe whether methyltransferase activity is required for the mitotic phenotype. These experiments are critical to link the structural/interaction data to m6A biology.

We thank the reviewer for this insightful suggestion. To test whether the mitotic defects are linked to loss of m^{6}A methyltransferase activity rather than solely to non-catalytic functions of WTAP, we performed pharmacological inhibition of METTL3 using the specific catalytic inhibitor STM2457[9], as guided by the reviewer. HeLa cells were synchronized by thymidine block and treated with either DMSO or 5 µM STM2457 for 24 h, followed by release into mitosis (Figure R12A). Mitotic progression was then monitored by live-cell imaging, and chromosome segregation defects were quantified. Time-lapse microscopy revealed that acute inhibition of METTL3 catalytic activity caused a significant increase in mitotic abnormalities, including lagging chromosomes and chromosome mis-segregation, thereby phenocopying the defects observed upon WTAP depletion (Figure R12B-D). These results support the conclusion that m^{6}A methyltransferase activity contributes to proper mitotic progression and strengthen the mechanistic link between WTAP-dependent writer complex assembly and mitotic fidelity.

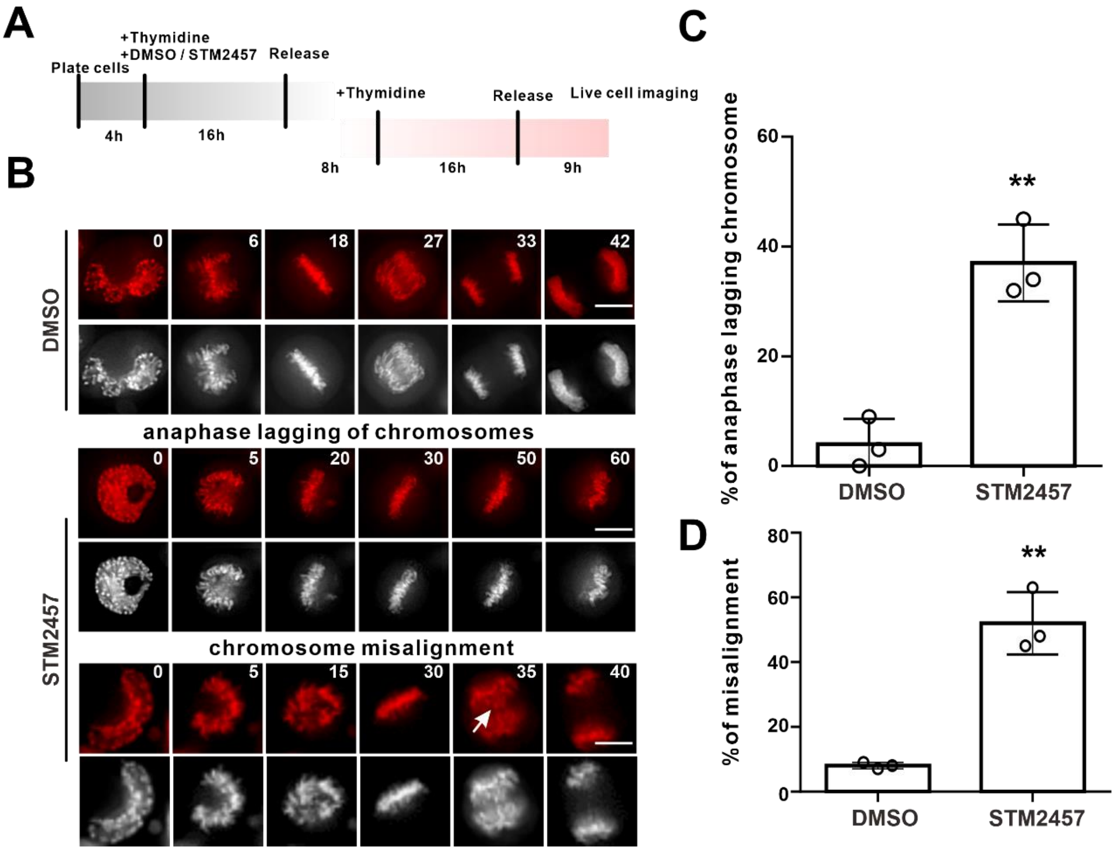

Figure R12. Pharmacological inhibition of METTL3 catalytic activity induces mitotic defects.

(A) Schematic diagram of the experimental design. HeLa cells were synchronized by thymidine block, treated with DMSO or STM2457, and released for live-cell imaging analysis.

(B) Representative time-lapse images of DMSO-treated and STM2457-treated HeLa cells expressing mCherry-H2B. Numbers indicate elapsed time in minutes. Arrow indicates a chromosome mis-segregation event. Scale bar: 10 µm.

(C) Quantification of anaphase cells exhibiting lagging chromosomes.

(D) Quantification of cells showing chromosome mis-segregation.

Data in (C) and (D) are presented as mean ± SEM from three independent experiments. Statistical significance was assessed using an unpaired two-tailed Student's *t*-test; **$p < 0.01$.

Minor/comments

1. The crystal structures should be described in greater detail with respect to the tetramerization interfaces: better specify the interface residues, the nature of the interactions, highlight the hydrophobic surfaces, and the key side chains in the figures.

We thank the reviewer for this helpful suggestion to provide a more detailed description of the tetramerization interface. In response, we performed an expanded structural analysis of the WTAP tetramer interface and revised the manuscript accordingly.

Our analysis shows that the interface is primarily stabilized by hydrophobic interactions between α-helices from adjacent monomers. Key residues contributing to this hydrophobic core include M158, I165, L204, L211, L225, L229, and L236, which form a layered hydrophobic packing at the tetramerization interface (Figure R13A). In addition to hydrophobic packing, we identified a salt bridge between R179 and E186, which may further contribute to the stabilization and specificity of the interface (Figure R13A, Layer 6). Furthermore, we analyzed the hydrophobic surface properties of the interacting helices. Prominent hydrophobic patches are observed at the interfaces between WTAP protomers (WTAP-A/A' and WTAP-B/B') (Figure R13B, C).These surfaces are largely formed by residues M158, I165, L204, and L211, which create a continuous hydrophobic patch that stabilizes the tetrameric assembly. These structural features are now highlighted in the revised figures and described in the text.

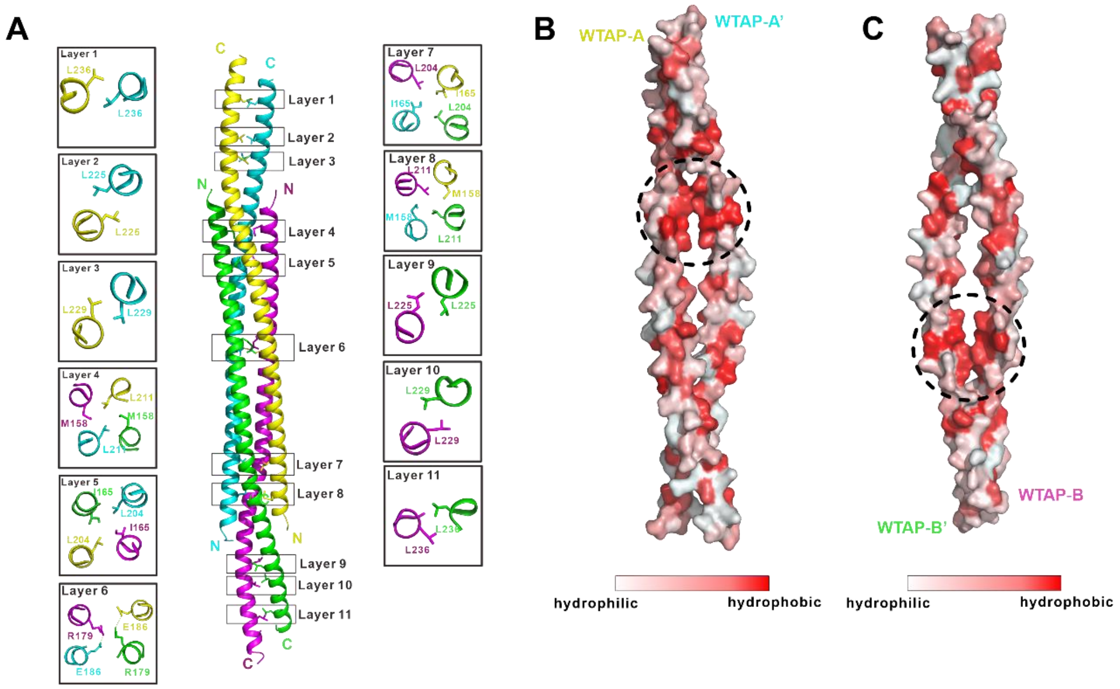

Figure R13. Structural features of the WTAP tetramerization interface.

(A) Schematic representation of the layered hydrophobic packing that stabilizes the WTAP tetramer interface. Key hydrophobic residues involved in the interface include M158, I165, L204, L211, L225, L229, and L236, which form a stacked hydrophobic core between adjacent helices.

(B, C) Surface representations of the tetramerization interface between WTAP protomers. Panel B shows the interface between WTAP-A and WTAP-A', and panel C shows the interface between WTAP-B and WTAP-B'. Hydrophobic surfaces are highlighted in red, illustrating the continuous hydrophobic patch that stabilizes the tetrameric assembly.

2. Discuss how the tetramer architecture is compatible with previously published structures of the methyltransferase complex. Does tetramerization sterically permit binding with respect to the previous described horse-shaped structure?

We thank the reviewer for raising this important structural question. The compatibility between the WTAP tetramer identified in our study and the previously reported architecture of the m^{6}A methyltransferase complex, often described as a "horse-shaped" structure, is indeed important for understanding how WTAP oligomerization integrates into the holocomplex assembly. As shown in Figure R1 and Figure R2 in our response to Reviewer #1, structural analysis indicates that the tetramerization interface and the VIRMA-binding interface are located on spatially distinct surfaces of WTAP. We generated a structural model by positioning our WTAP tetramer relative to the previously reported WTAP-VIRMA module. In this model, the tetramerization interface is located on the outer surface of the WTAP coiled-coil assembly, whereas the inner surface mediates binding to VIRMA. As a result, tetramer formation does not sterically occlude the VIRMA-binding region or the interfaces required for association with other components of the methyltransferase complex. This structural arrangement suggests that WTAP tetramerization is compatible with the previously described architecture of the m^{6}A writer complex and may provide multiple interaction surfaces within the holocomplex.

3. Figure S2 (FPLC MALS) should include the wild type proteins alongside mutants to directly compare oligomeric states.

We thank the reviewer for this helpful suggestion. In response, we have updated the figure to include FPLC-MALS analyses of both wild-type WTAP and the corresponding mutants, allowing direct comparison of their oligomeric states. As shown in Figure R14A, wild-type WTAP N-terminal fragment (residues 1-50) predominantly forms a dimer, with an experimentally determined molecular weight consistent with a dimeric assembly. In contrast, the W30R mutant shows a substantially reduced molecular weight corresponding to a monomer, indicating that the W30R mutation disrupts the N-terminal dimer interface. Similarly, as shown in Figure R14B, the WTAP middle coiled-coil fragment (residues 150-245) assembles into a tetramer as determined by FPLC-MALS analysis. In contrast, the L2E mutant abolishes tetramer formation and produces a monomeric species, demonstrating that this residue is critical for stabilizing the tetrameric assembly.

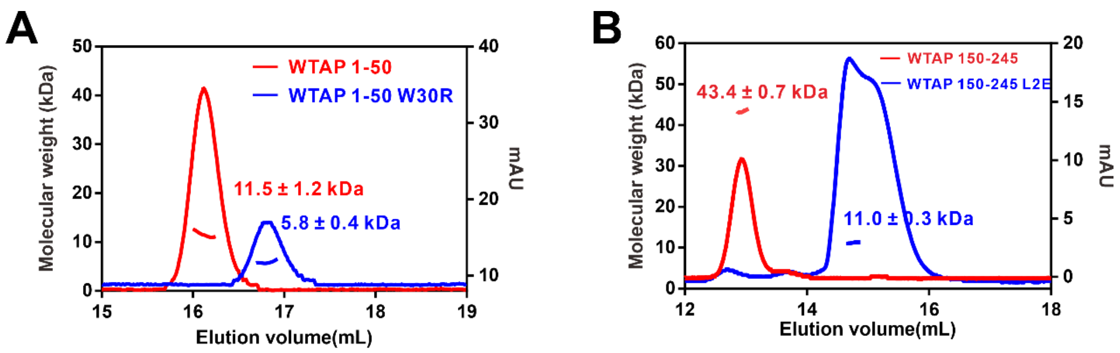

Figure R14. FPLC-MALS analysis of WTAP wild-type and oligomerization mutants.

(A) FPLC-MALS analysis of WTAP N-terminal fragment (residues 1-50). Wild-type protein forms a dimer, whereas the W30R mutant displays a reduced molecular weight corresponding to a monomer, indicating disruption of the N-terminal dimerization interface.

(B) FPLC-MALS analysis of the WTAP middle coiled-coil domain (residues 150-245). Wild-type WTAP forms a tetramer, while the L2E mutant results in a monomeric species, demonstrating that this residue is essential for tetramer stabilization.

Reference

1. Su, S. et al. Cryo-EM structures of human m6A writer complexes. Cell Res. 32, 982–994 (2022).
2. Wen, J. et al. Zc3h13 Regulates Nuclear RNA m6A Methylation and Mouse Embryonic Stem Cell Self-Renewal. Mol. Cell 69, 1028-1038.e6 (2018).
3. Knuckles, P. et al. Zc3h13/Flacc is required for adenosine methylation by bridging the mRNA-binding factor Rbm15/Spenito to the m 6 A machinery component Wtap/Fl(2)d. Genes Dev. 32, 415–429 (2018).
4. Schwartz, S. et al. Perturbation of m6A Writers Reveals Two Distinct Classes of mRNA Methylation at Internal and 5′ Sites. Cell Rep. 8, 284–296 (2014).
5. Liu, J. et al. A METTL3–METTL14 complex mediates mammalian nuclear RNA N6-adenosine methylation. Nat. Chem. Biol. 10, 93–95 (2014).
6. Horiuchi, K. et al. Wilms’ tumor 1-associating protein regulates G2/M transition through stabilization of cyclin A2 mRNA. Proc Natl Acad Sci U S A 103, 17278-17283 (2006).
7. Wang, M. et al. Downregulation of KIF20A induces cell cycle arrest and apoptosis by suppressing PI3K/AKT in human glioblastoma. Int J Clin Exp Med 10, 16133-16143 (2017).
8. Wu, W.-D., Yu, K.-W., Zhong, N., Xiao, Y. & She, Z.-Y. Roles and mechanisms of Kinesin-6 KIF20A in spindle organization during cell division. Eur. J. Cell Biol. 98, 74–80 (2019).
9. Yankova, E. et al. Small-molecule inhibition of METTL3 as a strategy against myeloid leukaemia. Nature 593, 597–601 (2021).

1st Revision - Editorial Decision 11th May 2026

Dear Dr. WANG,

Thank you for the submission of your revised manuscript. We have now received the enclosed reports from the referees and I am happy to say that both support its publication now. Only a few editorial requests will need to be addressed before we can proceed with the official acceptance of your manuscript:

- The author credits need to be removed from the ms file. All credits need to be entered during online ms submission.

- The following FUNDING INFO is missing in our online system: the Fundamental and Interdisciplinary Disciplines Breakthrough Plan of the Ministry of Education of China (JYB2025XDXM505), the Center for Advanced Interdisciplinary Science and Biomedicine of IHM (QYPY20220014), and the Strategic Priority Research Program of the Chinese Academy of Sciences (XDB0490000). These need to be added with the final ms submission.

- Appendix Table S1 is called out in the ms file but is missing. Supplementary information is not a correct callout. Please correct.

- Table EV1 should be removed from the ms and the separate file should not have a title page, just the page with the legend and the table; Table EV2 and Table EV3 look like datasets, so these should be corrected to Dataset EV1 and Dataset EV2 in all places (source file names, legends, titles in the system, ms and Reagents table callouts), then Table EV4 should be Table EV2. If sheet 2 can be removed from Table EV2, it can remain an EV table. Table EV4 could be part of the Reagents & Tools table and in this case deleted.

- Please note that the specific URLs for the 9XOE and 9XOF datasets need to be provided in the data availability statement.

* Figure Legends - Comments *

- Please define the annotated p values ****/***/**/* as well as provide the exact p-values for the same in the legend of figure 6B as appropriate and reasonable.
- Please note that the exact p values are not provided in the legends of figures 3D, 4B, F, G; 5B, C. Please provide exact values as reasonable.
- Please indicate the statistical test used for data analysis in the legends of figures 6A, C; EV5 C
- Please note that the box plots need to be defined in terms of minima, maxima, centre, bounds of box and whiskers, and percentile in the legend of figure 6B
- Please note that information related to n is missing in the legends of figures 4B, 6B
- Please note that the error bars are not defined in the legends of figure EV4 D

EMBO press papers are accompanied online by A) a short (1-2 sentences) summary of the findings and their significance, B) 2-3 bullet points highlighting key results and C) a synopsis image that is exactly 550 pixels wide and 300 pixels high. The synopsis image should provide a sketch of the major findings, like a graphical abstract. Please note that text needs to be readable at the final size. Please send us this information along with the final manuscript.

I look forward to seeing a final version of your manuscript as soon as possible.

Best regards,
Esther

Esther Schnapp, PhD
Senior Editor
EMBO reports

Referee #1:

Overall, the authors have addressed the concerns in a satisfactory manner

Referee #2:

The authors satisfactorily addressed my comments, in particular the link between the tetramer conformation and m6 as well as with the spindle phenotype is not more solid. I congratulate the authors for their interesting work.

All minor editorial requests have been addressed by the authors.

2nd Revision - Editorial Decision 21st May 2026

Dr. Chao WANG
University of Science and Technology of China
Division of Life Sciences and Medicine
No. 443 Huangshan Rd
Hefei, Anhui 230027
China

Dear Dr. WANG,

I am very pleased to accept your manuscript for publication in the next available issue of EMBO reports. Thank you for your contribution to our journal.

Your manuscript will be processed for publication by EMBO Press. It will be copy edited and you will receive page proofs prior to publication. Please note that you will be contacted by Springer Nature Author Services to complete licensing and payment information.

You may qualify for financial assistance for your publication charges - either via a Springer Nature fully open access agreement or an EMBO initiative. Check your eligibility: https://link.springer.com/journal/44319/how-to-publish-with-us

Should you be planning a Press Release on your article, please notify the production team via this form in order to coordinate publication and release dates: https://forms.cloud.microsoft/e/eH4A8JVP6g

If you have any questions, please do not hesitate to contact the Editorial Office. Thank you for your contribution to EMBO Reports.

Best regards,
Esther

Esther Schnapp, PhD
Senior Editor
EMBO reports

>>> Please note that it is EMBO Reports policy for the transcript of the editorial process (containing referee reports and your response letter) to be published as an online supplement to each paper. If you do NOT want this, you will need to inform the Editorial Office via email immediately. More information is available here: https://link.springer.com/partners/embo-press/editorial-policies#Peer%20review

EMBO Press Author Checklist

Corresponding Author Name: Chao WANG	
Journal Submitted to: EMBO Reports	
Manuscript Number: EMBOR-2025-63208V2	

USEFUL LINKS FOR COMPLETING THIS FORM
The EMBO Journal - Author Guidelines
EMBO Reports - Author Guidelines
Molecular Systems Biology - Author Guidelines
EMBO Molecular Medicine - Author Guidelines

Reporting Checklist for Life Science Articles (updated January
This checklist is adapted from Materials Design Analysis Reporting (MDAR) Checklist for Authors. MDAR establishes a minimum set of requirements in transparent reporting in the life sciences (see Statement of Task: 10.31222/osf.io/9sm4x). Please follow the journal's guidelines in preparing your
Please note that a copy of this checklist will be published alongside your article.

Abridged guidelines for figures
1. Data
The data shown in figures should satisfy the following conditions:
- the data were obtained and processed according to the field's best practice and are presented to reflect the results of the experiments in an accurate and unbiased manner.
- ideally, figure panels should include only measurements that are directly comparable to each other and obtained with the same assay.
- plots include clearly labeled error bars for independent experiments and sample sizes. Unless justified, error bars should not be shown for technical
- if n<5, the individual data points from each experiment should be plotted. Any statistical test employed should be justified.
- Source Data should be included to report the data underlying figures according to the guidelines set out in the authorship guidelines on Data

2. Captions
Each figure caption should contain the following information, for each panel where they are relevant:
- a specification of the experimental system investigated (eg cell line, species name).
- the assay(s) and method(s) used to carry out the reported observations and measurements.
- an explicit mention of the biological and chemical entity(ies) that are being measured.
- an explicit mention of the biological and chemical entity(ies) that are altered/varied/perturbed in a controlled manner.
- the exact sample size (n) for each experimental group/condition, given as a number, not a range;
- a description of the sample collection allowing the reader to understand whether the samples represent technical or biological replicates (including how many animals, litters, cultures, etc.).
- a statement of how many times the experiment shown was independently replicated in the laboratory.
- definitions of statistical methods and measures:
  - common tests, such as t-test (please specify whether paired vs. unpaired), simple χ2 tests, Wilcoxon and Mann-Whitney tests, can be unambiguously identified by name only, but more complex techniques should be described in the methods section;
  - are tests one-sided or two-sided?
  - are there adjustments for multiple comparisons?
  - exact statistical test results, e.g., P values = x but not P values < x;
  - definition of 'center values' as median or average;
  - definition of error bars as s.d. or s.e.m.

Please complete ALL of the questions below.
Select "Not Applicable" only when the requested information is not relevant for your study.

Materials

Newly Created Materials	Information included in the manuscript?	In which section is the information available? (Reagents and Tools Table, Materials and Methods, Figures, Data Availability Section)
New materials and reagents need to be available; do any restrictions apply?	Not Applicable	

Antibodies	Information included in the manuscript?	In which section is the information available? (Reagents and Tools Table, Materials and Methods, Figures, Data Availability Section)
For **antibodies** provide the following information:<br>- Commercial antibodies: RRID (if possible) or supplier name, catalogue number and or/clone number<br>- Non-commercial: RRID or citation	Yes	Reagents and Tools Table

DNA and RNA sequences	Information included in the manuscript?	In which section is the information available? (Reagents and Tools Table, Materials and Methods, Figures, Data Availability Section)
**Short novel DNA or RNA including primers, probes**: provide the sequences.	Yes	Materials and Methods, Data Availability Section (Table EV3)

Cell materials	Information included in the manuscript?	In which section is the information available? (Reagents and Tools Table, Materials and Methods, Figures, Data Availability Section)
**Cell lines:** Provide species information, strain. Provide accession number in repository **OR** supplier name, catalog number, clone number, and/**OR** RRID.	Yes	Reagents and Tools Table, Materials and Methods
**Primary cultures:** Provide species, strain, sex of origin, genetic modification status.	Yes	Reagents and Tools Table, Materials and Methods
Report if the cell lines were recently **authenticated** (e.g., by STR profiling) and tested for mycoplasma contamination.	Not Applicable	

Experimental animals	Information included in the manuscript?	In which section is the information available? (Reagents and Tools Table, Materials and Methods, Figures, Data Availability Section)
**Laboratory animals or Model organisms:** Provide species, strain, sex, age, genetic modification status. Provide accession number in repository **OR** supplier name, catalog number, clone number, **OR** RRID.	Not Applicable	Not applicable
**Animal observed in or captured from the field:** Provide species, sex, and age where possible.	Not Applicable	Not applicable
Please detail **housing and husbandry conditions**.	Not Applicable	Not applicable

Plants and microbes	Information included in the manuscript?	In which section is the information available? (Reagents and Tools Table, Materials and Methods, Figures, Data Availability Section)
**Plants:** provide species and strain, ecotype and cultivar where relevant, unique accession number if available, and source (including location for collected wild specimens).	Not Applicable	Not applicable
**Microbes:** provide species and strain, unique accession number if available, and source.	Yes	Materials and Methods

Human research participants	Information included in the manuscript?	In which section is the information available? (Reagents and Tools Table, Materials and Methods, Figures, Data Availability Section)
If collected and within the bounds of privacy constraints report on age, sex and gender or ethnicity for all study participants.	Not Applicable	Not applicable

Core facilities	Information included in the manuscript?	In which section is the information available? (Reagents and Tools Table, Materials and Methods, Figures, Data Availability Section)
If your work benefited from core facilities, was their service mentioned in the acknowledgments section?	Yes	Data Availability Section (Acknowledgments)

Design

Study protocol	Information included in the manuscript?	In which section is the information available? (Reagents and Tools Table, Materials and Methods, Figures, Data Availability Section)
If study protocol has been **pre-registered, provide DOI in the manuscript**. For clinical trials, provide the trial registration number **OR** cite DOI.	Not Applicable	Not applicable
Report the **clinical trial registration number** (at ClinicalTrials.gov or equivalent), where applicable.	Not Applicable	Not applicable

Laboratory protocol	Information included in the manuscript?	In which section is the information available? (Reagents and Tools Table, Materials and Methods, Figures, Data Availability Section)
Provide DOI OR other citation details if **external detailed step-by-step protocols** are available.	Not Applicable	Not applicable

Experimental study design and statistics	Information included in the manuscript?	In which section is the information available? (Reagents and Tools Table, Materials and Methods, Figures, Data Availability Section)
Include a statement about **sample size** estimate even if no statistical methods were used.	Not Applicable	Not applicable
Were any steps taken to minimize the effects of subjective bias when allocating animals/samples to treatment (e.g. **randomization procedure**)? If yes, have they been described?	Yes	Materials and Methods
Include a statement about **blinding** even if no blinding was done.	Not Applicable	Not applicable
Describe **inclusion/exclusion criteria** if samples or animals were excluded from the analysis. Were the criteria pre-established?<br><br>If sample or data points were omitted from analysis, report if this was due to attrition or intentional exclusion and provide justification.	Not Applicable	Not applicable
For every figure, are **statistical tests** justified as appropriate? Do the data meet the assumptions of the tests (e.g., normal distribution)? Describe any methods used to assess it. Is there an estimate of variation within each group of data? Is the variance similar between the groups that are being statistically compared?	Yes	Materials and Methods, Figures

Sample definition and in-laboratory replication	Information included in the manuscript?	In which section is the information available? (Reagents and Tools Table, Materials and Methods, Figures, Data Availability Section)
In the figure legends: state number of times the experiment was **replicated** in laboratory.	Yes	Figures
In the figure legends: define whether data describe **technical or biological replicates**.	Yes	Figures

Ethics

Ethics	Information included in the manuscript?	In which section is the information available? (Reagents and Tools Table, Materials and Methods, Figures, Data Availability Section)
Studies involving **human participants**: State details of **authority granting ethics approval** (IRB or equivalent committee(s), provide reference number for approval.	Not Applicable	Not applicable
Studies involving **human participants**: Include a statement confirming that **informed consent** was obtained from all subjects and that the experiments conformed to the principles set out in the WMA Declaration of Helsinki and the Department of Health and Human Services Belmont Report.	Not Applicable	Not applicable
Studies involving **human participants:** For publication of **patient photos**, include a statement confirming that consent to publish was obtained.	Not Applicable	Not applicable
Studies involving experimental **animals**: State details of **authority granting ethics approval** (IRB or equivalent committee(s), provide reference number for approval. Include a statement of compliance with ethical regulations.	Not Applicable	Not applicable
Studies involving **specimen and field samples:** State if relevant **permits** obtained, provide details of authority approving study; if none were required, explain why.	Not Applicable	Not applicable

Dual Use Research of Concern (DURC)	Information included in the manuscript?	In which section is the information available? (Reagents and Tools Table, Materials and Methods, Figures, Data Availability Section)
Could your study fall under dual use research restrictions? Please check biosecurity documents and list of **select agents and toxins** (CDC): https://www.selectagents.gov/sat/list.htm	Not Applicable	Not applicable
If you used a select agent, is the security level of the lab appropriate and reported in the manuscript?	Not Applicable	Not applicable
If a study is subject to dual use research of concern regulations, is the name of the **authority granting approval and reference number** for the regulatory approval provided in the manuscript?	Not Applicable	Not applicable

Reporting

The MDAR framework recommends adoption of discipline-specific guidelines, established and endorsed through community initiatives. Journals have their own policy about requiring specific guidelines and recommendations to complement MDAR.

Adherence to community standards	Information included in the manuscript?	In which section is the information available? (Reagents and Tools Table, Materials and Methods, Figures, Data Availability Section)
State if relevant guidelines or checklists (e.g., **ICMJE, MIBBI, ARRIVE, PRISMA**) have been followed or provided.	Not Applicable	Not applicable
For **tumor marker prognostic studies,** we recommend that you follow the **REMARK** reporting guidelines (see link list at top right). See author guidelines, under 'Reporting Guidelines'. Please confirm you have followed these guidelines.	Not Applicable	Not applicable
For **phase II and III randomized controlled trials**, please refer to the **CONSORT** flow diagram (see link list at top right) and submit the CONSORT checklist (see link list at top right) with your submission. See author guidelines, under 'Reporting Guidelines'. Please confirm you have submitted this list.	Not Applicable	Not applicable

Data Availability

Data availability	Information included in the manuscript?	In which section is the information available? (Reagents and Tools Table, Materials and Methods, Figures, Data Availability Section)
Have **primary datasets** been deposited according to the journal's guidelines (see 'Data Deposition' section) and the respective accession numbers provided in the Data Availability Section?	Yes	Data Availability Section
Were **human clinical and genomic datasets** deposited in a public access-controlled repository in accordance to ethical obligations to the patients and to the applicable consent agreement?	Not Applicable	Not applicable
Are **computational models** that are central and integral to a study available without restrictions in a machine-readable form? Were the relevant accession numbers or links provided?	Not Applicable	Not applicable
If publicly available data were reused, provide the respective **data citations in the reference list**.	Not Applicable	Not applicable
